# COMBImage: a modular parallel processing framework for pairwise drug combination analysis that quantifies temporal changes in label-free video microscopy movies

**DOI:** 10.1186/s12859-018-2458-x

**Published:** 2018-11-26

**Authors:** Efthymia Chantzi, Malin Jarvius, Mia Niklasson, Anna Segerman, Mats G. Gustafsson

**Affiliations:** 1 Department of Medical Sciences, Cancer Pharmacology and Computational Medicine, Uppsala University, Uppsala, Sweden; 2 SciLifeLab Drug Discovery and Development, In Vitro Systems Pharmacology Facility, Uppsala University, Uppsala, Sweden; 3 Department of Immunology, Genetics and Pathology, Rudbeck Laboratory, Uppsala University, Uppsala, Sweden

**Keywords:** Time-lapse video microscopy, Label-free, Drug combination analysis, Therapeutic synergy, MapReduce, Parallel image processing, Systematic parameter optimization, Glioblastoma multiforme

## Abstract

**Background:**

Large-scale pairwise drug combination analysis has lately gained momentum in drug discovery and development projects, mainly due to the employment of advanced experimental-computational pipelines. This is fortunate as drug combinations are often required for successful treatment of complex diseases. Furthermore, most new drugs cannot totally replace the current standard-of-care medication, but rather have to enter clinical use as add-on treatment. However, there is a clear deficiency of computational tools for label-free and temporal image-based drug combination analysis that go beyond the conventional but relatively uninformative end point measurements.

**Results:**

COMBImage is a fast, modular and instrument independent computational framework for in vitro pairwise drug combination analysis that quantifies temporal changes in label-free video microscopy movies. Jointly with automated analyses of temporal changes in cell morphology and confluence, it performs and displays conventional cell viability and synergy end point analyses. The image processing algorithms are parallelized using Google’s MapReduce programming model and optimized with respect to method-specific tuning parameters. COMBImage is shown to process time-lapse microscopy movies from 384-well plates within minutes on a single quad core personal computer.

This framework was employed in the context of an ongoing drug discovery and development project focused on glioblastoma multiforme; the most deadly form of brain cancer. Interesting add-on effects of two investigational cytotoxic compounds when combined with vorinostat were revealed on recently established clonal cultures of glioma-initiating cells from patient tumor samples. Therapeutic synergies, when normal astrocytes were used as a toxicity cell model, reinforced the pharmacological interest regarding their potential clinical use.

**Conclusions:**

COMBImage enables, for the first time, fast and optimized pairwise drug combination analyses of temporal changes in label-free video microscopy movies. Providing this jointly with conventional cell viability based end point analyses, it could help accelerating and guiding any drug discovery and development project, without use of cell labeling and the need to employ a particular live cell imaging instrument.

**Electronic supplementary material:**

The online version of this article (10.1186/s12859-018-2458-x) contains supplementary material, which is available to authorized users.

## Background

Large-scale drug combination analysis (CA) using quantitative label-free time-lapse video microscopy (TLVM) imaging constitutes a yet unconventional method that could offer increased in vitro drug testing sensitivity and efficacy, compared to conventional end point methods [1]. Although well established, such assays may be uninformative; either because of misalignment with respect to the cell cycle duration time or due to chemically induced changes not being associated with altered end point readouts. Moreover, they provide neither temporal information about when the chemical perturbations are taking effect, nor dynamical information about how the effects evolve as a function of time. Fluorescent labeling, although undoubtedly emerging and powerful [2], especially when combined with advanced and robust data analytics [3, 4], requires an extra step of adding reagents. This may result in undesirable interferences either with the drugs or natural cellular functions, as well as cellular perturbations due to repeated UV light exposure [5]. Thus, despite lacking specific molecular changes, label-free measurements are overall very attractive, since they are less costly, labor intensive and error prone [5].

The strong potential of label-free quantitative TLVM imaging in the form of automated quantification of differences in time evolving morphologies (AQDTEM) using the pixel histogram and hierarchy comparison (PHHC) algorithm has already been demonstrated in the context of in vitro cancer pharmacology studies. It was used to identify morphology modulating drugs as a complement/alternative to cell viability assays [6, 7], establish cell line identity control procedures [7, 8], as well as to detect differential drug activity in iso-genic cell line pairs [7, 8]. However, this suite of computational tools for extracting general morphological differences in in vitro growing cell cultures as a function of time, has neither been applied to nor generalized for drug CA. Moreover, method-specific optimized parameter tuning has not been an option due to long running times, and has therefore resulted in the employment of ad hoc parameter settings. Furthermore, these label-free algorithmic methods have not been robustified against outliers, which often cause great variability in the image quality between different time points and/or experimental wells and thereby, falsify the interpretation of the obtained results. Thus, there is an apparent need for improving, refining, speeding up and incorporating these algorithms into a generic and modular computational infrastructure that can be easily employed to a wide variety of similar applications, including more sophisticated phenotypic drug discovery and development (DDD) [3, 9].

Unlike the limited use of label-free temporal imaging, drug CA based on single end point readouts, such as cell viability, is a commonly used practice supported by commercial tools [10–12] and large industrial efforts [13]. Lately, open source packages that enable large-scale pairwise drug CA have been developed, including Combenefit [14], COMBIA [15] and SynergyFinder [16, 17] (see Table 1). At the same time, it has been shown that this type of conventional synergy analysis, focused merely on target cells and defined as any positive deviation from trivial cases, may be completely misleading when it comes to the detection of large in vitro therapeutic windows, which is of pre-clinical and pharmacological interest [18–20]. The strength of therapeutic synergy (TS) analysis [18, 19], and its rare use compared to target cell focused (conventional) synergy analysis, are motivations for performing automated quantification of TS in drug CA studies.

**Table 1:** Comparison of the four latest and free softwares for in vitro pairwise drug combination analysis developed during the period 2016−2018

Features		COMBImage	COMBIA	SynergyFinder	Combenefit
Cell viability	Synergy/antagonism models	B, RB, T, RT	B, L, T	B, L, HSA, ZIP	B, L, HSA, SANE
	Non-parametric statistics	+	+	−	−
	Graphics	+	+	+	+
	Result tables	+	+	+	+
TLVM	Morphological changes	+	−	−	−
	Confluence changes	+	−	−	−
	MapReduce	+	−	−	−
	Parameter optimization	+	−	−	−
	Non-parametric statistics	+	−	−	−
	Graphics	+	−	−	−
	Result tables	+	−	−	−
Other	OS	Windows	all	all	all
	Available as	Standalone	R-package	Web application	Standalone, M-package

They are listed in descending chronological order from the newest (left) to the oldest (right). The compared features (rows) are organized in two main algorithmic categories based on the employed readouts: (**1**) Cell Viability ; (**2**) Time-lapse video microscopy (TLVM) and a third one: (**3**) Other, which provides functional and technical details. Explanation of the abbreviations used follows. B: Bliss, RB: Refined Bliss, T: Therapeutic, RT: Refined Therapeutic, L: Loewe, HSA: Highest Single Agent, ZIP: Zero Interaction Potency, SANE: Synergy Antagonism or Neutrality Estimation, M-package: MATLAB package

High throughput screening (HTS) employing TLVM generates large datasets since the recording time may range from days to weeks resulting in hundreds of gigabytes (GB) or even terabytes (TB) of data. The produced data volume might either not fit into memory or require long running times by using conventional data analytics. Therefore, storage and processing time are two main challenges for building corresponding computationally efficient tools [4]. State-of-the-art distributed computing technologies, like Hadoop MapReduce [21–23], have already shown strong potential in predicting effective drug combinations from integrating the gene expression data of the combined drugs [24], as well as accelerating the detection of adverse drug effects for pharmacovigilance [25]. However, the use of the MapReduce paradigm in biomedical applications is still quite limited and its employment to AQDTEM using TLVM imaging could pave the way for more sophisticated, scalable and fault tolerant DDD platforms.

In this study, we designed and developed COMBImage (see Table 1); a fast, modular and instrument independent computational framework for large-scale pairwise drug CA and visualization, incorporating MapReduce-based and optimized AQDTEM. It consists of three toolboxes compatible with custom experimental layouts in a checkerboard format (i.e., all combinations of two drugs at *n* doses each): (a) COMBO-V offers conventional cell viability and subsequently Bliss [26] and TS analyses. Both these types of synergy analysis are refined by means of a weighting step with the aim to provide a practically more useful ranking of the combination effects observed; (b) COMBO-M offers robustified, parallelized and method-specific optimized analyses of the changes in morphology over time; (c) COMBO-C offers robustified, parallelized and automated quantification of changes in confluence (AQC). The user gets access to high quality global checkerboard style screens and custom text files with all results. Although MapReduce is well suited for processing of vast datasets across hundreds or thousands of servers in a Hadoop cluster, our results suggest that already employing this technique on multi core computers, for smaller datasets, offers unprecedented performance. In particular, TLVM movies from 384-well plates were processed within a few minutes in a single quad core personal computer. The currently described version of COMBImage is distributed as a package of three standalone applications for Windows with appropriate documentation via the figshare repository [27–29].

### Case study

The prospect of COMBImage was demonstrated in the context of an ongoing DDD project, focused on the development of new multicompound therapies for glioblastoma multiforme (GBM); a highly aggressive and the most common primary brain tumor in adults. It exhibits extremely poor prognosis that has been attributed to not only genetic, but also intratumoral heterogeneity that is thought to be linked to therapy resistance and disease relapse [30, 31]. A promising approach towards combating GBM, as well as any other complex disease, is the discovery of drug coktails, which in contrast to monotherapy, could overcome resistance, achieve better efficacy and reduce the risk of adverse reactions [18]. However, such an early DDD phase requires in vitro drug testing and evaluation. COMBImage was employed in this context as a novel image-based drug CA tool that goes beyond the single end point cell viability measurements. In particular, it was used to evaluate the add-on effects of two investigational cytotoxic drug candidates along with the standard-of-care temozolomide (TMZ), and vorinostat (SAHA) that has shown effect and high tolerance in patients with recurrent GBM [32].

**Fig. 1:**
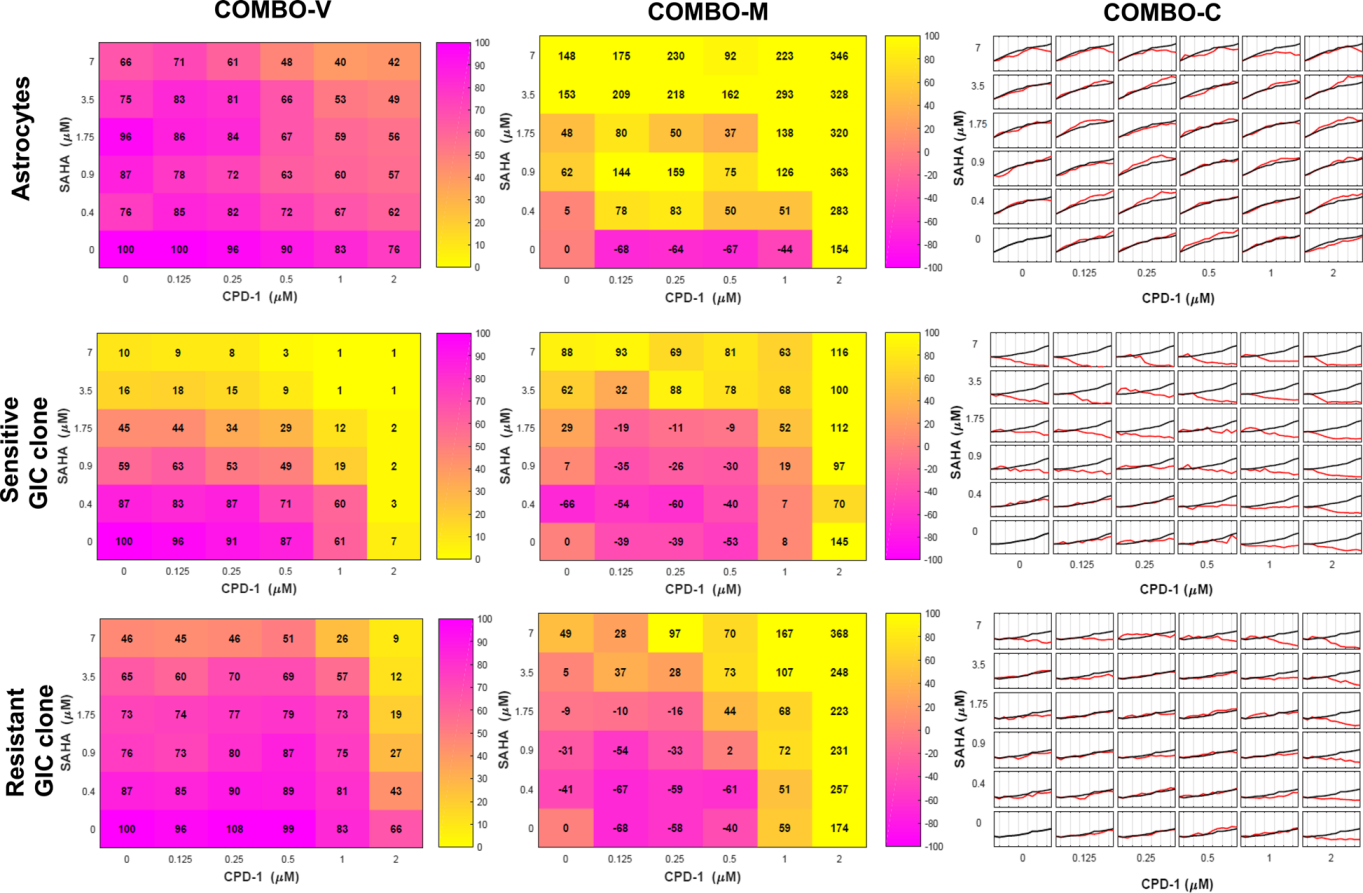
COMBImage Case Study. Selected results in checkerboard style screens produced by **COMBO-V**, **COMBO-M** and **COMBO-C**, for normal astrocytes (first row), sensitive GIC clone (second row) and resistant GIC clone (third row), when SAHA was combined with CPD-1, an investigational cytotoxic compound. The information shown for a particular combination concentration patch is as follows. **COMBO-V:** cell viability, from purple showing full cell survival (100%) to yellow showing zero cell survival (0%). **COMBO-M:** relative difference from the top 5% of corresponding natural/untreated morphological effects, from purple being −100*%* to yellow being 100%. **COMBO-C:** growth curves of treated (red) and untreated (black) GIC clones. All growth curves are expressed with respect to the first time point

Notably, the drug combination effects were evaluated on two recently established clonal cultures of glioma-initiating cells (GICs) from patient tumor samples [31]; one drug sensitive and one multidrug resistant. This is attractive in a pharmacological context, as the GIC clones have stem cell properties, reflect intratumoral heterogeneity and are close to the original patient cells due to their low passage number. TSs were identified for both investigational cytotoxic compounds, when combined with SAHA. The TSs were apparent as the viability of the reference (toxicity) cell model in the form of normal astrocytes (ACS) was mainly unaffected, compared to the GBM clones where cell death was induced. However, these combinations started to substantially modulate the morphologies of the GBM cells and ACS in different directions. Finally, quantification of changes in cell confluence confirmed the aforementioned therapeutic synergies, while revealing delayed but equal effects on the resistant compared to the sensitive GIC clone. These results are summarized in Fig. 1.

## Results

Results from COMBImage are provided in the context of the current case study. Since all the four different drug pairs were duplicated in each 384-well plate (see “Methods” section), only the merged values are shown. This is a user-defined option during the initiation of all three toolboxes, when there are intra-plate replicates. However, global visualization is also supported, meaning that all drug pairs on a single plate are visualized separately. In both cases, all generated graphics are based on a checkerboard format as heatmaps and growth curves.

### COMBO-V

**Fig. 2:**
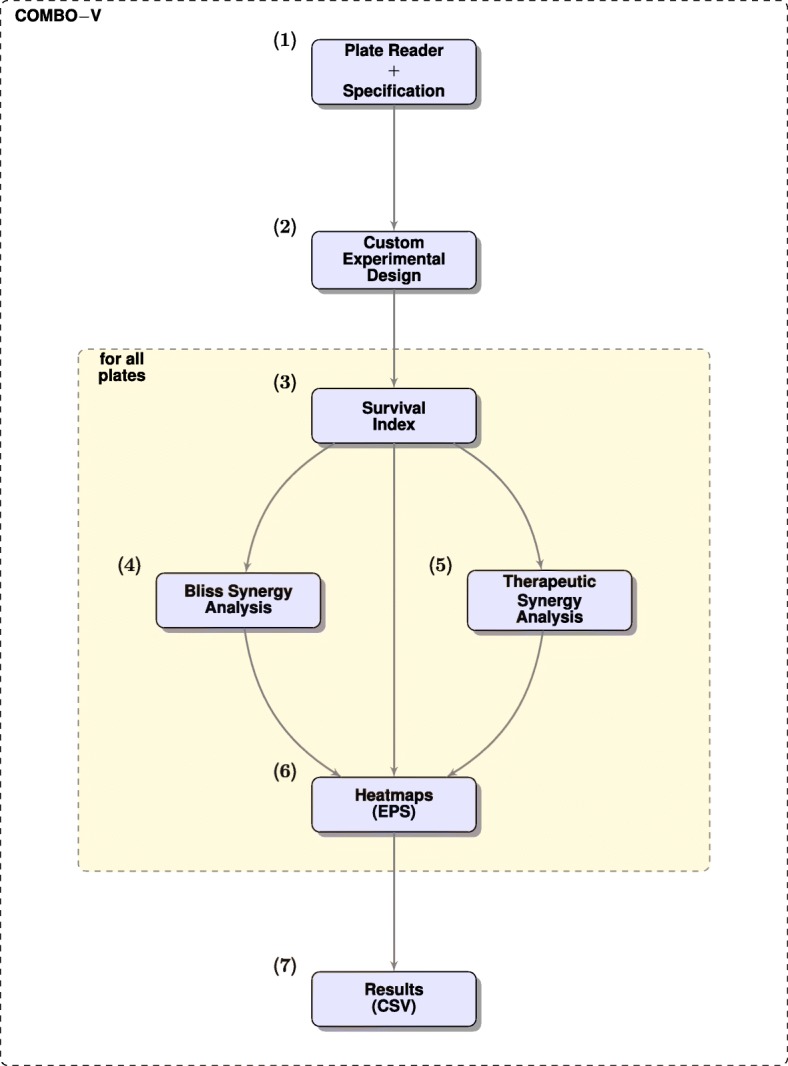
COMBO-V Flowchart. (**1**) Microplate reader and specification files are selected by the user; (**2**) Deployment of the custom experimental format; (**3**) FMCA-based cell viability analysis; (**4**) Conventional and scaled Bliss synergy analysis; (**5**) Conventional and refined therapeutic synergy analysis, if reference cell model system exists; (**6**) Global checkerboard style screens as heatmaps in EPS file format; (**7**) Extraction of results in CSV file format

COMBO-V (Fig. 2), as part of COMBImage, offers conventional cell viability and subsequently Bliss and TS analyses (see “Methods” section and Table 1). The latter two are further refined by means of a weighting step for ranking of the observed combination effects. The running time for analyzing, generating and storing the results for all three 384-well plates of the current case study was approximately 1 *min*. Notably, non-parametric statistics are also provided in the presence of inter-plate replicates, as extensively described and shown by us in a previous work [33].

In case of intra-plate replicate wells, only survival index values with standard deviation smaller than 30% were kept and merged. Notably, the aforementioned cut-off threshold for the standard deviation is a user-defined parameter during the initiation of COMBO-V. White patches annotated with “X” in the generated graphics indicate excluded values from the analyses due to this criterion.

#### Cell viability analysis and visualization

All results from cell viability analyses and associated comments are provided as additional files (“Cell cultures” section and Additional file 8: Figures S1-S3). Only partial results are shown in the first column of Fig. 1.

#### Bliss synergy analysis and visualization

All results from conventional and scaled/refined Bliss synergy analyses and associated comments are provided as additional files (“Chemical compounds” section and Additional file 8: Figures S4-S6).

#### TS analysis and visualization

All results from conventional TS analysis and associated comments are provided as additional files (“Experimental format” section and Additional file 8: Figure S7). The main focus is given to the refined TS analysis, which offered less misleading results due to ranking/sorting (see “Methods” section).

**Fig. 3:**
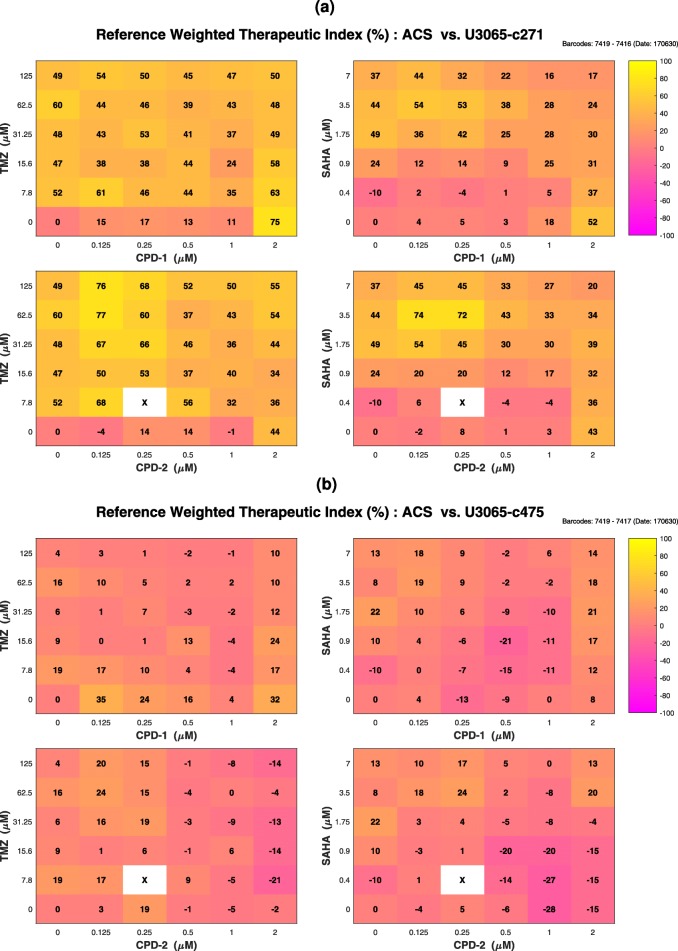
COMBO-V Checkerboard Style Screens. Refined therapeutic synergy analysis: **a** astrocytes (ACS) vs. sensitive GIC clone (*U*3065−*c*271); **b** astrocytes (ACS) vs. resistant GIC clone (*U*3065−*c*475). The color of each combination concentration patch represents the reference weighted therapeutic index, *T*_*RW*_ (%), from purple showing maximal therapeutic antagonism (−100*%*) to yellow showing maximal therapeutic synergy (100%). White patches annotated with “X” are related to survival index values with more than 30% standard deviation between the intra-plate replicates, which have been subsequently excluded

Overall, therapeutic windows were observed between astrocytes and the sensitive GIC clone *U*3065−*c*271 (Fig. 3a), for all four different drug pairs, especially when either CPD-1 or CPD-2 was combined with TMZ. Regarding astrocytes and the resistant GIC clone *U*3065−*c*475 (Fig. 3b), therapeutic windows were mainly observed, when CPD-2 and especially CPD-1, both at the highest concentration (2*μ*M), were combined with SAHA.

The strength of employing TS analysis over target cell focused Bliss synergy analysis was clearly shown when comparing the Bliss index values *B*_*S*_ (Additional file 8: Figure S5b) with the therapeutic index values *T*_*RW*_ (Fig. 3a) for the combination concentrations (CPD-2, SAHA) = (0.25*μ*M, 3.5*μ*M) and (CPD-2, SAHA) = (0.5*μ*M, 3.5*μ*M). Although the corresponding *B*_*S*_ values were −1*%*, in both cases, indicating Bliss antagonism, the *T*_*RW*_ values were 74% and 72% respectively, showing substantial TS.

### COMBO-M

**Fig. 4:**
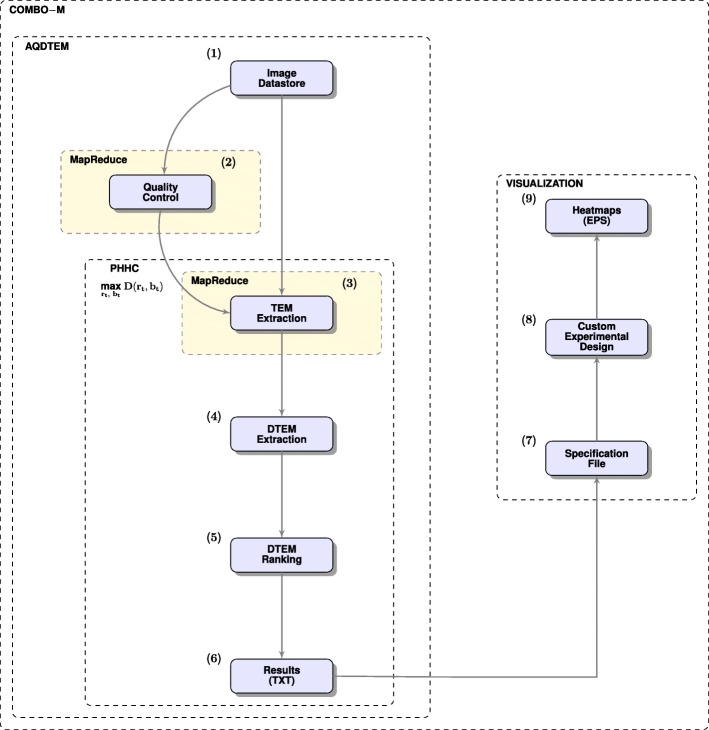
COMBO-M Flowchart. (**1**) Image datastore is selected by the user; (**2**) Image quality control, back up and foreground segmentation; (**3**) MapReduce TEM extraction; (**4**) DTEM extraction; (**5**) DTEM ranking based on null hypothesis testing; (**6**) Extraction of raw results in TXT file format; (**7**) Specification file is selected by the user; (**8**) Deployment of the custom experimental format; (**9**) Global checkerboard style screens as heatmaps in EPS file format. Modules (**3**) - (**6**) are executed for all parameter pairs (*r*,*b*) per decreasing time interval *t*, starting with all time points and ending up to only the last time point. The optimum pair for each decreasing time interval *t*, is the one that maximizes the number of detections

COMBO-M (Fig. 4), as part of COMBImage, offers robustified, MapReduce-based and method-specific optimized AQDTEM via the PHHC algorithm (see “Methods” section and Table 1). The running time for this algorithm on the time-lapse microscopy movies per 384-well plate of the current case study was approximately 5 *min*, including parameter optimization.

#### Image quality control

**Fig. 5:**
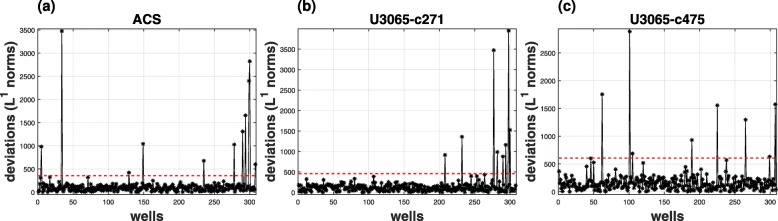
Image Quality Control. Detected and subsequently excluded outliers from all image processing steps: **a** astrocytes (ACS); **b** sensitive GIC clone (*U*3065−*c*271); **c** resistant GIC clone (*U*3065−*c*475). Each data point representing a particular experimental well, was detected as an outlier, if its corresponding *L*^1^-norm was equal to or greater than the detection threshold shown by the red dotted line

The first part of the AQDTEM module of COMBO-M is image quality control. Outliers are detected and subsequently excluded from all subsequent image processing steps (see “Methods” section). Figure 5 shows the distribution of all experimental wells per plate of the current case study as occurring from the image quality control step (see Additional file 7 for an example), where treatment effects are not taken into account.

#### Systematic PHHC parameter optimization

Benefiting from the very fast running times, the changes in morphology via the PHHC algorithm are quantified for the whole parameter grid, using a sequence of decreasing time intervals. This is in order to investigate how different parameter settings affect the analyses and optimize the subsequent results. Furthermore, in this way, the temporal behavior of the detected morphological changes as well as the contribution of different time points are also monitored.

**Fig. 6:**
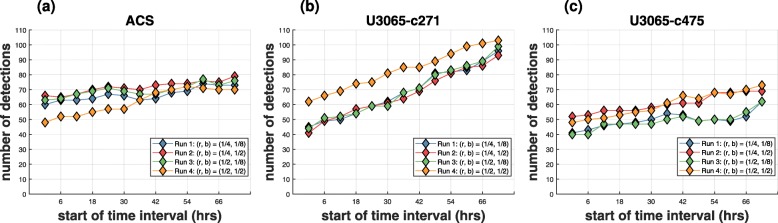
Optimized PHHC Analyses. Number of detections (interesting morphological changes compared to untreated controls) using 13 decreasing time intervals for the 4 runs of the currently employed parameter grid: **a** astrocytes (ACS); **b** sensitive GIC clone (*U*3065−*c*271); **c** resistant GIC clone (*U*3065−*c*475). The values on the x-axis correspond to the first time point for a particular time interval (e.g., 0*h* indicates the time interval [0*h*,72*h*], 6*h* indicates the time interval [6*h*,72*h*], etc.)

In terms of the current case study, the PHHC algorithm for each parameter pair (see “Methods” section and Table 2), was employed 13 times in total; firstly, using all available 13 time points, secondly, excluding the first and including all the remaining 12 time points and so on, until only the last time point was used (see Fig. 6, Additional file 8: Figure S8).

**Table 2:** Parameter grid

(r, b)	Run
$\left (\frac {1}{4},\frac {1}{8} \right)$	#1
$\left (\frac {1}{4},\frac {1}{2} \right)$	#2
$\left (\frac {1}{2},\frac {1}{8} \right)$	#3
$\left (\frac {1}{2},\frac {1}{2} \right)$	#4

Currently employed parameter grid for MapReduce PHHC algorithm, where *r* and *b* are the scale reductions factors of resolution and number of bins respectively, at each hierarchical level

#### Optimized PHHC results

Given that early time points seemed to be less informative for all three cell model systems (Fig. 6), checkerboard style screens were generated only for later time points using the corresponding suggested optimum parameter settings (see “Methods” section). The illustrated values in Fig. 6 show the distance in % from the “null” threshold (see “Methods” section), which in this case corresponds to the top 5% of untreated morphological effects. Thus, all positive values are statistically significant.

**Fig. 7:**
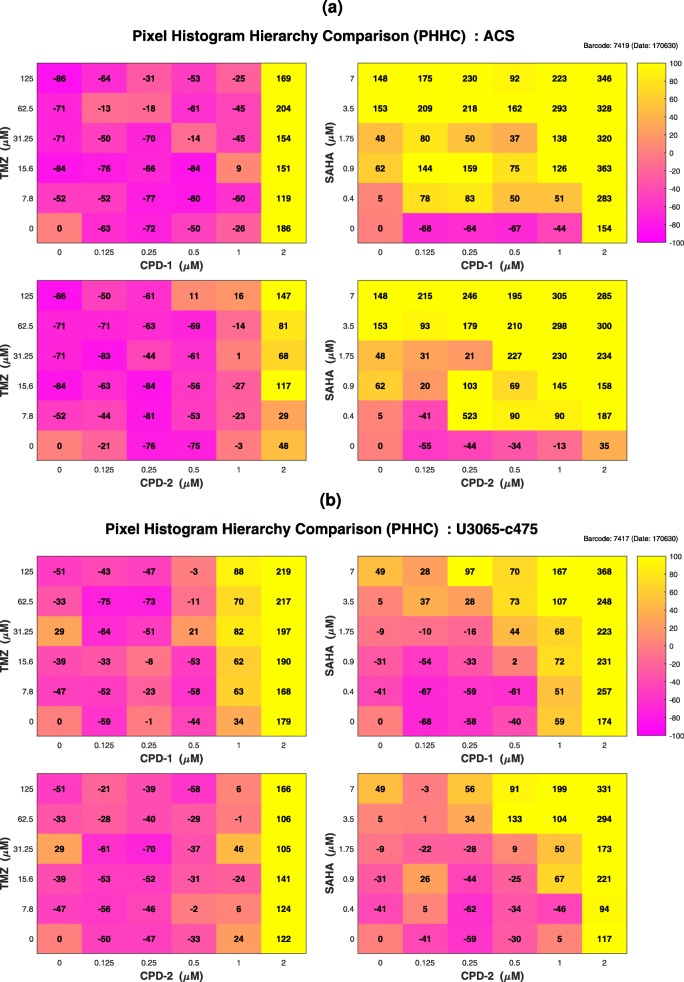
COMBO-M Checkerboard Style Screens. PHHC analyses: **a** Astrocytes (ACS) using the optimum parameter pair $(r^{*}, b^{*}) = \big (\frac {1}{2}, \frac {1}{8}\big)$ for the time interval [60*h*,72*h*]; **b** resistant GIC clone (*U*3065−*c*475) using the optimum parameter pair $(r^{*}, b^{*}) = \big (\frac {1}{2}, \frac {1}{2}\big)$ for the time interval [54*h*,72*h*]. The color of each combination concentration patch represents the relative difference (%) from the top 5% of the corresponding natural/untreated morphological effects, from purple being −100*%* to yellow being 100%

**Fig. 8:**
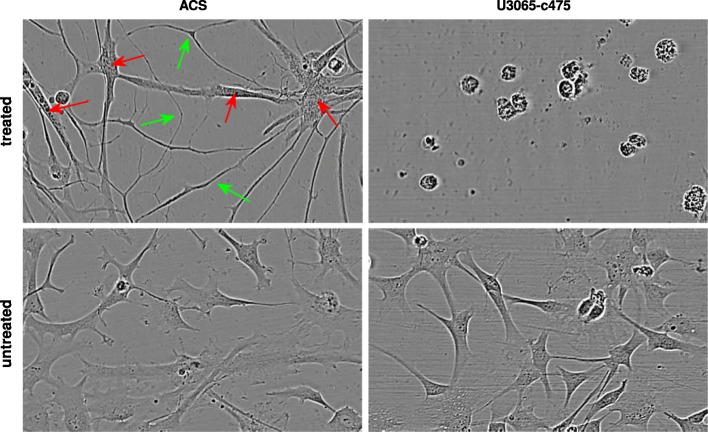
Interesting Morphological Detections by COMBO-M. **(first column)** Astrocytes (ACS) treated with (CPD-1, SAHA) = (2*μ*M, 7*μ*M) for 72*h* vs. untreated; **(second column)** resistant GIC clone (*U*3065−*c*475) treated with (CPD-1, SAHA) = (2*μ*M, 7*μ*M) for 72*h* vs. untreated. Red arrows show examples of increased dense formation of intracellular particles, while green arrows illustrate examples of long cellular protrusions

Starting with the normal astrocytes (Fig. 7a), interesting morphological cellular changes were detected when either CPD-1 or CPD-2 was combined with SAHA across almost the whole concentration grid (also with TMZ but only when the highest concentration of CPD-1/CPD-2 was used). The results suggest that interesting morphological changes were induced by the single drugs, which were clearly reinforced by their combination, showing increasing tendency with larger changes at higher doses. Based on visual inspection, the morphological changes originating from SAHA and CPD-1/CPD-2 alone, include mainly what could be described as long cellular protrusions (see Additional file 1) and increased formation of dense intracellular particles (see Additional file 2), respectively. An example showing the combination of these morphological effects after 72 *h* of treatment is shown in the left part of Fig. 8 (see also Additional file 3 for the whole movie).

The morphology of the resistant GIC clone *U*3065−*c*475 (Fig. 7b) was substantially affected when either CPD-1 or CPD-2 was combined with SAHA (also with TMZ but only when the highest concentrations of CPD-1/CPD-2 were used). The results suggest that interesting morphological changes were induced by CPD-1/CPD-2 alone in the highest dose, which were clearly reinforced by the combination with SAHA, showing an increasing gradient towards higher concentrations. Based on visual inspection, the morphological changes originating from CPD-1/CPD-2 alone, include mainly what could be described (similarly to before) as increased formation of dense intracellular particles (see Additional file 4), followed by cell death at late time points after the combination with SAHA (right part of Fig. 8, Additional file 5 for the whole movie).

The changes in morphology of the sensitive GIC clone *U*3065−*c*271 (Additional file 8: Figure S9) resemble those of the resistant GIC clone *U*3065−*c*475 mentioned above, but with the difference that increased cell death seems to be induced much earlier.

### COMBO-C

**Fig. 9:**
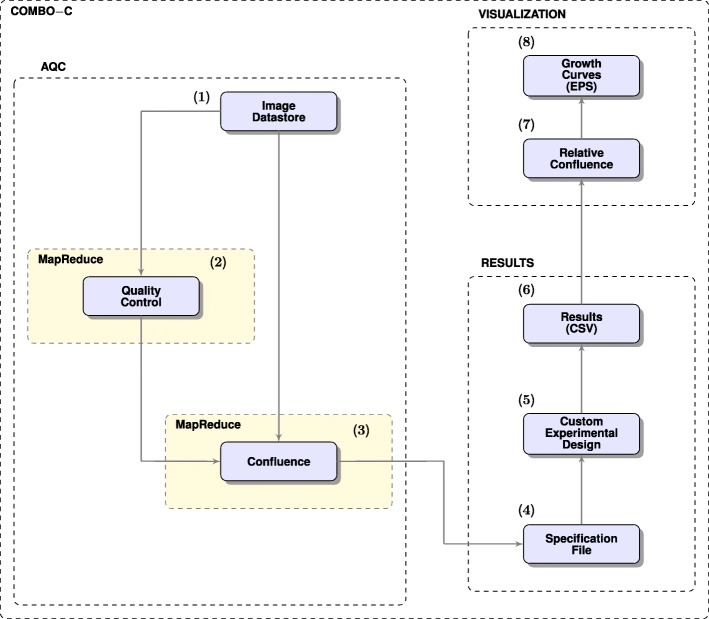
COMBO-C Flowchart. (**1**) Image datastore is selected by the user; (**2**) Image quality control, back up and foreground segmentation; (**3**) MapReduce confluence quantification; (**4**) Specification file is selected by the user; (**5**) Deployment of the custom experimental format; (**6**) Extraction of raw confluence values in CSV file format; (**7**) Quantification of changes in confluence with respect to the first time point; (**8**) Global checkerboard style screens as growth curves in EPS file format

COMBO-C (Fig. 9), as part of COMBImage offers robustified and MapReduce-based quantification of changes in confluence (see “Methods” section and Table 1), which are visualized as checkerboard style screens. The y-axis of the resulting growth curves corresponds to the change in confluence with respect to the first time point, displayed in the range [−50*%*,120*%*]. Thus, the lowest displayed value corresponds to 50% decrease in confluence, while the highest value corresponds to 120% increase in confluence, compared to the first time point. The running time for performing AQC of the time-lapse microscopy movies per 384-well plate of the current case study was approximately 2*min*.

#### Growth curves

**Fig. 10:**
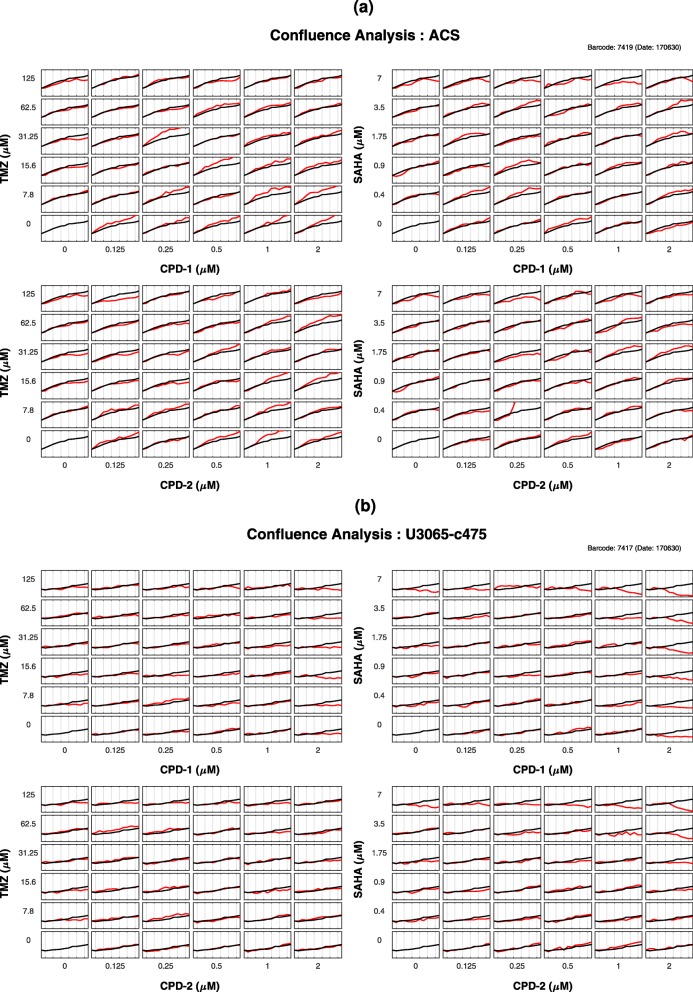
COMBO-C Checkerboard Style Screens. Quantification of changes in confluence: **a** Astrocytes (ACS); **b** resistant GIC clone (*U*3065−*c*475). The median growth curve of all untreated wells (black) is shown alone in the lower left subplot of each drug pair, as well as together with the growth curves of treated (red) cells. All growth curves are expressed with respect to the first time point

Treated astrocytes showed overall increases in confluence over time compared to untreated astrocytes (Fig. 10a). However, when SAHA was used in the highest concentration (7*μ*M), decreasing trends were observed at late time points. This was in alignment with the corresponding results from the cell viability analysis (Additional file 8: Figure S1).

As for the resistant GIC clone *U*3065−*c*475 (Fig. 10b), the confluence dropped as much as for the sensitive GIC clone *U*3065−*c*271 (Additional file 8: Figure S10), but at later time points and to a much more limited extent across the whole concentration grid. In particular, the results suggest decreases in cell growth when either SAHA or CPD-1 are used alone in the highest concentrations (not for CPD-2 though). However, a substantial downward trend was observed, when SAHA was combined with either CPD-1 or CPD-2 at the highest concentrations, indicating synergistic combination effects.

Notably, the confluence curve of (CPD-2, SAHA) = (0.25*μ*M, 0.4*μ*M) (Fig. 10a) was the only curve with a substantially upward trend, starting from *t*_6_=30*h*, which based on visual inspection, is consistent with a bacterial infection (see Additional file 6). Similarly, COMBO-M quantified the aforementioned combination concentration to have the highest value of 523% (Fig. 7a). Although of limited biological interest in the context of the current case study, this well could be perceived as a positive control example, showing the proper functionality of COMBO-M and COMBO-C.

## Discussion

The present study introduces COMBImage; a fast, modular and instrument independent computational framework for pairwise image-based drug CA and visualization. It currently consists of three different toolboxes; COMBO-V, COMBO-M and COMBO-C that all together could guide and accelerate any DDD project, especially in early phases. The potential of COMBImage was particularly demonstrated in the context of an ongoing DDD project, where two investigational cytotoxic compounds were evaluated for potential add-on treatment of GBM. Different aspects related to the importance of COMBImage as a DDD tool, are discussed in smaller distinct sections below.

### Pharmacological aspects

Drug combinations with great potency as GBM treatment were discovered, after evaluating their effects in vitro on a sensitive and a multidrug resistant patient derived GIC clones, as well as on a reference (toxicity) cell model in the form of normal astrocytes. Given the available cell viability data, we conclude that the two investigational cytotoxic drug candidates CPD-1 and CPD-2 in combination with SAHA at the highest concentrations induced cell death on the GBM cells (Additional file 8: Figures S2-S3). Notably, promising therapeutic synergies were found as they seemed to affect relatively little the cell viability of astrocytes. This result was further supported by cell confluence analysis (Fig. 10b and Additional file 8: Figure S10), which additionally revealed a delay of approximately 12−18 h for the confluence of the resistant GIC clone to drop as much as for the sensitive GIC clone. Moreover, these drug pairs seemed to induce interesting morphological changes both on normal astrocytes and the GBM cells where cell death was induced as well (Fig. 8). Regarding the biological effect of the investigational compounds alone, given the cell viability data, one can only conclude that CPD-1 induced cell death on the sensitive GBM cells (Additional file 8: Figures S2-S3). Further studies to elucidate the exact mechanism of cell death are performed independently as part of another project and thus, are not shown here.

### Computational aspects

State-of-the-art distributed computing techniques, like MapReduce, facilitate fast and reliable algorithmic implementations even on multi core computers that can be used in most labs nowadays, without necessitating cloud infrastructures that may require additional expertise related, for example, to maintenance and security configurations. Although vast datasets (multiterabytes) necessitate the deployment of large clusters of commodity hardware for scalable and fault tolerant processing, as well as for storage, there are datasets that may fit in memory and still benefit from very short running times provided by parallel processing.

Speed increases capacity, quality and sophistication. Here, systematic parameter optimization was enabled, showing the importance of generic, powerful and sensitive tools that are not restricted to arbitrary parameter settings. Different parameters were associated with the optimal results for the three cell models studied. Decreasing gradually the time interval of the compared video microscopy movies revealed that excluding earlier time points with limited information, increased the sensitivity of the analyses by generating much more consistent results.

In our previous work related to AQDTEM via the PHHC algorithm, the pixel histograms were extracted based on all pixels in the images [6, 8]. As a consequence, those histogram features were not only dependent on the actual changes in cell morphology but partially also on the confluence, which may be regarded as another type of global morphological feature. There are several advantages associated with extracting the morphological features exclusively based on the foreground pixels as by COMBO-M. Firstly, the features are dependent exclusively on changes in morphology and not confluence of the cells, which is in turn separately extracted and visualized by COMBO-C. Secondly, we achieve robustness against variability in the cell seeding, temporal fluctuations in the confluence present in the particular subpart of the well where the microscopy images are collected, as well as artifacts such as wave patterns, dust particles, and scratches that quite often are visible in the cell free background.

### Statistical aspects

Our results suggest that the sensitivity of the AQDTEM is greatly influenced by the resolution of the microscope objective. A 20 × objective, like the one used here, often results in very few cells per image and thus, unreliable detections. Another related statistical problem with such cases, is that the background experimental noise also contribute to unreliable detections. Although using a 10 × objective will decrease the resolution of individual cells, it will provide images with a much larger part of the cell population studied, which is crucial for tools that rely on comparison of general statistical properties over time.

### Image quality control and visualization

Image quality control steps are necessary for the development of robustified computational methods against outliers, which often cause great variability in the image quality and therefore, may falsify the interpretation of the obtained results. Such outliers should always be removed, especially from growth control wells that are examples of expected/natural morphological effects under no treatment. Finally, global checkerboard style screens illustrating the whole experimental plate are very convenient and helpful for getting a general grasp of the results, especially in large-scale experiments.

### Limitations

Although this work demonstrates that DDD can be guided and substantially accelerated by tools that offer label-free image-based drug CA, there is still great potential to extend and refine the current functionality. Scaling up the framework by using larger datasets, which are more well suited for the MapReduce programming model, could be a first step forward. An obvious limitation is the employment of AQDTEM using only the PHHC algorithm. Thus, the development of alternative feature extraction methods are required, in order to determine which approaches offer the best balance between sensitivity and robustness. Furthermore, the currently detected morphological changes over time do not have any particular direction, as they can deviate from natural morphological effects in many different ways. Therefore, a preprocessing step for enhancing and quantifying specific morphological features of interest would be attractive. Finally, given the relatively unexplored potential of high-order drug combinations [34] as well as the necessity of multidrug therapies for combating complex disorders [18], the pairwise drug CA offered currently by COMBImage should definitely be expanded.

## Conclusions

In brief, the main contributions of this study are:

- A demonstration of how the challenges associated with long running times may be successfully addressed by employing modern parallel data analysis methods, such as Google’s MapReduce programming model. Although the current implementation can be scaled up to run on large computer clusters, our results suggest unprecedented performance already when employing it for smaller datasets on multi core computers. This opens for fast image processing and thus, systematic parameter optimization on local machines, providing convenience and possibilities to keep precious data locally, especially for early phases in DDD.
- COMBImage; an instrument independent, integrated and modular computational framework for pairwise drug CA, which performs and displays cell viability and synergy analyses jointly with parallelized and optimized quantification of changes in morphology and confluence in label- free video microscopy movies.
- A small illustrative case study not only showing how COMBImage can be used to accelerate pairwise drug CA, but also revealing combination effects of outstanding pharmacological interest in patient derived tumor initiating clones from GBM; the most deadly form of brain cancer [30, 31].
- New examples of drug CA results where conventional Bliss synergy analyses of tumor initiating cell clones are misleading compared to a TS analysis, which compares the effects on the clonal cells with reference cells; normal astrocytes in this case.

## Methods

### Cell cultures

The GBM clonal cell cultures, *U*3065−*c*271 and *U*3065−*c*475 [31], were cultured in neural stem cell media (1:1 mix of DMEM-F12 GlutaMAX medium and Neurobasal medium (Life Technologies/GIBCO-Invitrogen) containing 1% penicillin G/streptomycin sulfate (Sigma-Aldrich, St. Louis, MO), supplemented with B-27 without vitamin A (1:50; Invitrogen), N2 supplement (1:100; Invitrogen), 10 ng/mL EGF and 10 ng/mL FGF-2 (PeproTech, Rocky Hill, NJ). Human cerebral cortex astrocytes (*#*1800, ScienCell, Carlsbad, CA) were cultured in astrocyte medium (containing basal medium, 2% fetal bovine serum, 1% penicillin G/streptomycin sulfate and astrocyte growth supplement (*#*1801, ScienCell)). Cells were seeded in poly-L-ornithine (P4957, Sigma-Aldrich) and laminin (L2020, Sigma-Aldrich) coated 384-well plates (164688, Thermo Fisher Scientific) at a density of 1000 cells/well using a BioMek 4000 (Beckman Coulter). All cells were seeded 24 h prior to treatment with compounds.

### Chemical compounds

Four different chemical compounds were used in this study; two investigational cytotoxic compounds, denoted as CPD-1 and CPD-2, the HDAC inhibitor vorinostat (SAHA) and the alkylating agent temozolomide (TMZ). CPD-1 and CPD-2 were combined with SAHA and TMZ, resulting in four different drug pairs, as duplicates per 384-well plate (see Table 3 for doses).

**Table 3:** Concentrations of chemical compounds

Drug	Concentration c (*μM*)				
	*c*_1_	*c*_2_	*c*_3_	*c*_4_	*c*_5_
CPD-1	0.125	0.25	0.5	1	2
CPD-2	0.125	0.25	0.5	1	2
SAHA	0.4	0.9	1.75	3.5	7
TMZ	7.8	15.6	31.25	62.5	125

Non-zero concentration range of the four chemical compounds used in the case study, which were combined in a checkerboard format; all four drugs in all possible concentrations. The concentrations were selected based on an initial dose-response analysis

### Experimental format

All currently compatible experimental layouts, developed in-house for different DDD projects, are included in Table 4. Here, the first one was used. Larger experimental formats (e.g., 1536-well plates) can be also integrated to the current modular computational framework.

**Table 4:** Experimental Layouts

Well plate	*#* Drug pairs	Concentration grid (*n*×*n*)
384	8	6×6
	15	4×4
	20/21	

Currently compatible 384-well experimental layouts, where the drugs are combined in a checkerboard format; all combinations of two drugs at *n* doses each. The values *n* include the zero concentration of the single drugs

### Assay for determination of survival index

Cell survival was calculated by means of the Fluorometric Cytotoxicity Assay [35, 36] (FMCA). Cell survival for a combination concentration (*c*_1_,*c*_2_) of drugs 1 and 2 respectively, known as the survival index and denoted here as *S*, is calculated by means of Eq. (1):

1 $$ S\left(c_{1}, c_{2}\right) = \frac{f\left(c_{1}, c_{2}\right) - \tilde{f}_{blank}}{\tilde{f}_{control} - \tilde{f}_{blank}}   $$

Here, *f*(*c*_1_,*c*_2_) corresponds to the fluorescence signal from the experimental well of the combination concentration (*c*_1_,*c*_2_), while $\tilde {f}_{blank}$ and $\tilde {f}_{control}$ denote the median fluorescence signals from the blank and growth control wells, respectively. For drugs causing growth inhibition and/or cell killing, the range of *S*(*c*_1_,*c*_2_) spans from 0 to 1 indicating minimal and maximal cell survival, respectively, compared to untreated controls.

### Synergy analysis

Pairwise combination effects are assessed using conventional target cell focused Bliss [26] synergy, as well as reference cell focused TS analyses. Both types of synergy analysis are further refined by an extra weighting step with the aim to sort/rank the observed combination effects. *B* and *T* denote the conventional Bliss and therapeutic indices respectively, while *B*_*S*_ and *T*_*RW*_ denote the corresponding indices after sorting/ranking (Additional file 8: “Conventional and Scaled Bliss Synergy Analysis” and “Conventional and Refined Therapeutic Synergy Analysis” sections).

### Label-free video microscopy screening

Phase-contrast time-lapse microscopy images were acquired using the IncuCyte FLR (Essen BioScience Inc.) located inside the incubator. The microscope had a 20× objective with the ability to capture high quality phase-contrast microscopy images, 1024×1280 pixels each. 15 frames/images per experimental well were acquired, one every 6*h* (the first two without treatment). The total size of image data per 384-well plate was 6.14 GB.

### Time evolving morphologies (TEM)

Time evolving morphologies (TEM) are extracted as pixel histograms at multiple consecutively decreasing resolution levels from all experimental wells. For simplicity, the extraction procedure is described for a particular experimental well *w* and hence, one time-lapse movie with *n* time points/frames in total. Starting with the first time frame *t*_1_, a pixel histogram is extracted in the original, as well as in consecutively decreasing resolutions as long as the number of bins is greater or at least equal to 2 (Additional file 8: Table ST1), resulting in *m* different resolutions. Since each pixel histogram is one-dimensional (1-D), it is translated into a feature column vector $\mathbf {h}_{w,r_{1}}(t_{1})$ with length equal to the number of bins, for the original resolution *r*_1_ and first time point *t*_1_. By merging together sequentially the obtained feature vectors $\mathbf {h}_{w,r_{j}}(t_{1})$ with *j*={1,2,⋯,*m*}, a larger feature column vector **h**_*w*_(*t*_1_), with length equal to the cumulative number of bins from all hierarchical levels, is generated. By repeating this extraction procedure for all the *n* time frames for well *w*, the following feature matrix **H**_*w*_, which contains the TEM for well *w*, is obtained:

2 $$\begin{array}{@{}rcl@{}} \mathbf{H}_{w} &=&\begin{bmatrix} \mathbf{h}_{w}\left(t_{1}\right) & {} & \cdots & {} & \mathbf{h}_{w}\left(t_{n}\right) \end{bmatrix}\\ &=&\begin{bmatrix} \mathbf{h}_{w,r_{1}}\left(t_{1}\right) & \cdots & \mathbf{h}_{w,r_{1}}\left(t_{n}\right)\\ \mathbf{h}_{w,r_{2}}\left(t_{1}\right) & \cdots & \mathbf{h}_{w,r_{2}}\left(t_{n}\right)\\ \vdots & \ddots & \vdots\\ \mathbf{h}_{w,r_{m}}\left(t_{1}\right) & \cdots & \mathbf{h}_{w,r_{m}}\left(t_{n}\right)\\ \end{bmatrix}  \end{array} $$

### MapReduce TEM extraction

The *MapReduce* programming model, as provided by MATLAB R2017b [37], is used for the TEM extraction in the form of hierarchical pixel histograms. The *Map* function extracts the hierarchical pixel histograms of single frames, while the *Reduce* function merges the hierarchical pixel histograms of all frames per experimental well. By default, the MapReduce TEM extraction is executed on a local parallel pool by deploying all available cores of the computer used. Here, 4 cores were deployed.

### Image quality control

The image quality control step requires the number of untreated frames as a user input during the initiation of the framework. The main idea is that all experimental wells (should) look similar, as long as there are no ongoing treatment effects. This quality control step is based on MapReduce TEM extraction as described by Eq. (2), where all pixel values are used for each hierarchical level. For each row in the matrix *H*_*w*_, an average feature value over the whole untreated time interval is calculated resulting in $\mathbf {\overline {h}_{w}}$, which is an individual average feature column vector under no treatment. Then, a global average feature vector, denoted here as $\mathbf {\overline {h}}$, is obtained by averaging all $\mathbf {\overline {h}_{w}}$. Finally, the deviations between individual and global averages are calculated as:

3 $$ \mathbf{e}_{w} = \mathbf{\overline{h}_{w}} - \mathbf{\overline{{h}}}   $$

The *L*^1^-norms of all individual difference vectors **e**_*w*_ are obtained as follows and create a statistical pool *P*_*e*_:

4 $$ P_{e} = \left\{\left\|\mathbf{e}_{1}\right\|_{1}, \hspace{5mm} \cdots\cdots, \hspace{5mm} \left\|\mathbf{e}_{n}\right\|_{1}\right\}, \hspace{6mm} {w: 1 \leq w \leq n}   $$

Here, *n* denotes the total number of wells per experimental plate. As outliers are characterized and subsequently excluded, all the wells *w*, whose *L*^1^-norm is greater than or equal to 1.5 times the 95^*th*^ percentile of the statistical pool *P*_*e*_, as expressed by (4).

The parameter settings used for the MapReduce TEM extraction on this step are 128 bins for the original resolution, a scale reduction factor $r = \frac {1}{4}$ in resolution and subsequently, a scale reduction factor $b = \frac {1}{8}$ in the number of bins.

### Foreground segmentation

Global threshold based segmentation is used in order to divide the images into foreground and background pixels. This step aims at extracting only the foreground pixels for the main analyses. Given that *f*(*x*,*y*) and *g*(*x*,*y*) denote the original and new pixel values at position (*x*,*y*), respectively, the employed thresholding operation to define the foreground as pixel values being equal to one, can be defined as:

5 $$ g(x, y) = \begin{cases} 1 & \quad \text{if}\ \ \ f \,(x, y) \notin \tau\\ 0 & \quad \text{otherwise}\\ \end{cases}   $$

Here, *τ* is a pre-defined intensity interval. It is adaptively selected based on the first time point(s) of all experimental wells, where the background is assumed to dominate. More precisely, the median pixel value *μ*_*w*_ of the first frame(s) for each well *w* is calculated. Then, all these values *μ*_*w*_ are merged to create a statistical pool *P*_*b*_ of background intensities across the whole image library. Finally, the interval *τ* is selected based on the median value of the samples in the pool *P*_*b*_, reflecting a global background intensity, expressed as:

6 $$ P_{b} = \left\{\mu_{1}, \hspace{5mm} \cdots\cdots, \hspace{5mm}\mu_{n}\right\}, \hspace{6mm} \{w: 1 \leq w \leq n\}   $$

where *n* denotes the total number of experimental wells. Then, the associated global background intensity estimate is:

7 $$ \mu_{b} = median\left\{P_{b}\right\}   $$

In the current analysis, *μ*_*b*_ was multiplied by 0.95 and 1.05, in order to allow for ±5*%* deviations, respectively, resulting in the following background intensity interval *τ*.

8 $$ \tau \!\in \left[\tau_{l}, \tau_{h}\right], \quad\text{where} \hspace{1mm} \!\left\{\tau_{l}, \tau_{h}: \hspace{1mm} \!\tau_{l} \,=\, 0.95\cdot \mu_{b}, \hspace{1mm} \tau_{h} \,=\, 1.05\cdot \mu_{b}\right\}   $$

Notably, the aforementioned absolute deviation is a user-defined parameter in the general framework.

### Extraction of differences in TEM (DTEM extraction)

The MapReduce TEM extraction for the main analyses is based exclusively on the pixels that belong to the foreground and thus, satisfy (5) and (8). Each hierarchical pixel histogram extracted on the foreground is also normalized (area underneath equal to 1), so as the corresponding features are not dependent on cell confluence but merely on cell morphology. The number of bins for the first hierarchical level (original resolution of images) is set to 16. Differences in TEM (DTEM) are calculated, in order to assess the deviation of the chemically induced morphological changes from natural morphological effects observed without treatment. The DTEM of the control wells are evaluated by employing the following leave-one-out procedure to the *N*_*c*_ control wells, where each control well *c*, on a plate *p*, is considered as a treated well without effect:

9 $$ \mathbf{\Delta H}(c, p) = \mathbf{H}(c, p) - \frac{1}{N_{c}-1}\sum\limits_{\substack {k = 1 \\ k \neq c}}^{N_{c}} \mathbf{H}(k, p), \hspace{6mm} c,k \in I_{control}   $$

Here, *I*_*control*_ denotes the set of well indices corresponding to the *N*_*c*_ growth control wells only. This procedure is employed iteratively for all growth control wells that belong to a particular plate *p*. Regarding treated wells, the corresponding DTEM are calculated as the difference of the individual TEM from the average control TEM. In particular, for a treated well *d* on plate *p*, this is achieved by means of the following equation:

10 $$ \mathbf{\Delta H}(d, p) = \mathbf{H}(d, p) - \frac{1}{N_{c}}\sum\limits_{c = 1}^{N_{c}} \mathbf{H}(c, p), \hspace{6mm} d \in I_{treated}   $$

Here, *I*_*treated*_ denotes the set of well indices corresponding to treated wells only.

### DTEM comparison

The DTEM of all control and treated wells are compressed into a scalar by calculating the corresponding *L*^1^-norms as follows, assuming that the DTEM matrices *ΔH* have dimensions *K*×*T*, where *K* and *T* are the total number of features and time points, respectively:

11 $$ \left\|\mathbf{\Delta H}(c, p)\right\|_{1} = \sum\limits_{t = 1}^{T} \sum\limits_{k = 1}^{K} \left|\mathbf{\Delta H}_{kt}(c, p)\right|, \hspace{6mm} c \in I_{control}   $$

12 $$ \left\|\mathbf{\Delta H}(d, p)\right\|_{1} = \sum\limits_{t = 1}^{T} \sum\limits_{k = 1}^{K} \left|\mathbf{\Delta H}_{kt}(d, p)\right|, \hspace{6mm} d \in I_{treated}   $$

*I*_*control*_ and *I*_*treated*_ denote the sets of well indices corresponding to control and treated wells, respectively. All values calculated by (11) are pooled together forming a statistical pool, *Δ*_*c*_, of magnitudes under the null hypothesis, where all the acquired differences are naturally observed without treatment and thus, they are not considered interesting. The “null” distribution *Δ*_*c*_ is expressed as:

13 $$ \begin{aligned} \Delta_{c} =& \left\{\left\|\mathbf{\Delta H}(c_{1}, p)\right\|_{1}, \hspace{5mm} \cdots\cdots, \hspace{5mm} \left\|\mathbf{\Delta H}(c_{n}, p)\right\|_{1}\right\},\\ \hspace{4mm} &c_{i} \in I_{control}  \end{aligned}  $$

The 95^*th*^ percentile *τ*_95_ of this distribution was used here, as a threshold above which, the null hypothesis was rejected, meaning that the observed differences were detected as interesting. Notably, this threshold, also referred to as the “null” threshold, is a user-defined parameter in the general framework. Finally, the relative difference between the calculated magnitudes and *τ*_95_ is calculated, denoted as $\widetilde {d}$ for a particular well *w* and defined as:

14 $$ \begin{aligned} \widetilde{d}(w) =& \frac{\left\|\mathbf{\Delta H}(w, p)\right\|_{1} - \tau_{95}}{\tau_{95}} = \frac{\left\|\mathbf{\Delta H}(w, p)\right\|_{1}}{\tau_{95}} - 1,\\ \hspace{3.5mm} &w \in \left\{I_{control}, I_{treated}\right\}  \end{aligned}  $$

This gives the value zero at *τ*_95_ and subsequently, negative and positive values below and above *τ*_95_, respectively. In our framework, morphological changes for a particular well *w* are detected as interesting and thus, called detections *D*, when $\widetilde {d}(w) > 0$, simply meaning that the null hypothesis is rejected. Accordingly, morphological changes are considered uninteresting when $\widetilde {d}(w) < 0$, simply meaning that the null hypothesis is not rejected. Thus, summing the number of experimental wells for which the null hypothesis is rejected, yields the total number of detections *N*_*D*_:

15 $$ N_{D} = \sum\limits_{\{w: \hspace{2.5mm} \widetilde{d}(w) > 0\}}\widetilde{d}(w)   $$

Notably, as a consequence of using *τ*_95_, the probability of false alarm equals 5% because this is the fraction of untreated wells being detected as interesting.

### Parameter optimization

Two tuning parameters are optimized for the PHHC algorithm. These are the scale reduction factor *r* in the resolution at each hierarchical level and the corresponding scale reduction factor *b* in the number of bins. The number of bins is reduced when reducing the resolution of an image, since the intensity information is decreased as well (Additional file 8: Table ST1). Currently, the pipeline runs through a 2×2 parameter grid, as shown in Table 2. The parameter pair (*r*^∗^,*b*^∗^) that maximizes the number of detections *N*_*D*_ is suggested as the optimal and the corresponding optimization problem can be expressed as:

16 $$ \begin{aligned} & \underset{{r,\ b}}{\text{max}}\ {N_{D}(r, b)} \end{aligned}  $$

### PHHC algorithm

Using the title names of the aforementioned sections, algorithm 1 describes the functionality of the PHHC:


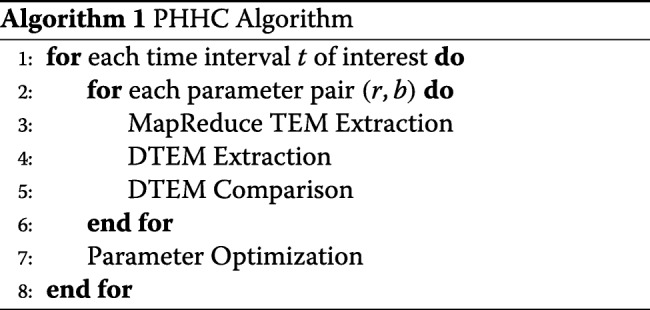


### Confluence

For each frame/time point *t* per experimental well, the cell confluence *c* is calculated as the fraction of foreground pixels, as given by summing all pixels satisfying (5) and then dividing by the total number of pixels *N*:

17 $$ c(t) = \frac{1}{N}\cdot \sum\limits_{\{(x, y):g(x,y,t)=1\}}g(x, y, t)   $$

Here *N*=1310720, since there were 1024×1280 pixels per frame.

### Relative confluence

In order to quantify and express changes in confluence between consecutive time points, we introduce an alternative measure of confluence, which is expressed relative to the first time point *t*_1_, when the treatment is just added. For a particular experimental well *w* and time point *t*_*i*_, the relative confluence $\widetilde {c}_{w}(t_{i})$ is defined as:

18 $$ \widetilde{c}_{w}(t_{i}) = \frac{c_{w}(t_{i})-c_{w}(t_{1})}{c_{w}(t_{1})} = \frac{c_{w}(t_{i})}{c_{w}(t_{1})} - 1   $$

This gives the value zero at the first time point, $\widetilde {c}_{w}(t_{1})=0$, and subsequently negative or positive values at later time points corresponding to decreases or increases in confluence with respect to *t*_1_, respectively. The main advantages of this relative measure is that it establishes a reference value $\widetilde {c}_{w}(t_{1}) = 0$ and compensates for differences in cell seeding that make the results between different experimental wells hard to compare using the conventional confluence measure.

### MapReduce confluence quantification

The *MapReduce* programming model, as provided by MATLAB R2017b [37], is used for confluence quantification. The *Map* function performs foreground segmentation as defined by (5) and (8), while the *Reduce* function calculates the fraction of foreground pixels as expressed by (17), per frame. By default, the MapReduce confluence quantification is executed on a local parallel pool by deploying all available cores of the computer used. Here, 4 cores were deployed.

### Programming environment and operating system

COMBImage was developed in MATLAB R2017b [37] under Windows 10. The data analyses were performed on a single personal computer with an Intel Core i7-6700HQ CPU, quad-core 2.6GHz, 32GB RAM, 128GB M.2 SSD and 1TB 7200rpm HDD. For scaling up the MapReduce implementation in a Hadoop cluster, the MATLAB Distributed Computing Server [38] is required.

## Additional files

Additional file 1 Video microscopy movie 1. 13 frames of the time-lapse microscopy movie (.tif format) corresponding to normal astrocytes when treated with 3.5*μ*M of SAHA alone. (ZIP 18,177 kb)

Additional file 2 Video microscopy movie 2. 13 frames of the time-lapse microscopy movie (.tif format) corresponding to normal astrocytes when treated with 2*μ*M of CPD-1 alone. (ZIP 20,242 kb)

Additional file 3 Video microscopy movie 3. 13 frames of the time-lapse microscopy movie (.tif format) corresponding to normal astrocytes when treated with the combination concentration (CPD-1, SAHA) = (2*μ*M, 7*μ*M). (ZIP 18,617 kb)

Additional file 4 Video microscopy movie 4. 13 frames of the time-lapse microscopy movie (.tif format) corresponding to the resistant GIC clone *U*3065−*c*475 when treated with 2*μ*M of CPD-2 alone. (ZIP 20,155 kb)

Additional file 5 Video microscopy movie 5. 13 frames of the time-lapse microscopy movie (.tif format) corresponding to the resistant GIC clone *U*3065−*c*475 when treated with the combination concentration (CPD-1, SAHA) = (2*μ*M, 7*μ*M). (ZIP 17,362 kb)

Additional file 6 Video microscopy movie 6. Time-lapse microscopy movie (.tif format) showing effects consistent with a bacterial contamination from time point 6 onwards. (ZIP 23,423 kb)

Additional file 7 Video microscopy movie 7. Time-lapse microscopy movie (.tif format) detected as an outlier and subsequently removed from all processing steps, during image quality control. (ZIP 18,130 kb)

Additional file 8 Supplementary Information. Single document (.pdf format) with extensive text, additional figures and tables. (ZIP 1466 kb)

## Abbreviations

ACS: Astrocytes
AQC: Automated quantification of confluence
AQDTEM: Automated quantification of differences in time evolving morphologies
CA: Combination analysis
DDD: Drug discovery and development
DTEM: Differences in time evolving morphologies
FMCA: Fluorometric cytotoxicity assay
GB: Gigabytes
GBM: Glioblastoma multiforme
GIC: Glioma-initiating cell
HTS: High-throughput screening
PHHC: Pixel histogram hierarchy comparison
SAHA: Vorinostat
TEM: Time evolving morphologies
TLVM: Time-lapse video microscopy
TMZ: Temozolomide
TS: Therapeutic synergy
*U*3065−*c*271: Sensitive GIC clone
*U*3065−*c*475: Resistant GIC clone
