# Supplementary figures and images for: COMBImage: a modular parallel processing framework for pairwise drug combination analysis that quantifies temporal changes in label-free video microscopy movies
Source: BMC Bioinformatics. 2018 Nov 26;19:453. doi: 10.1186/s12859-018-2458-x (PMC6257977; doi:10.1186/s12859-018-2458-x)

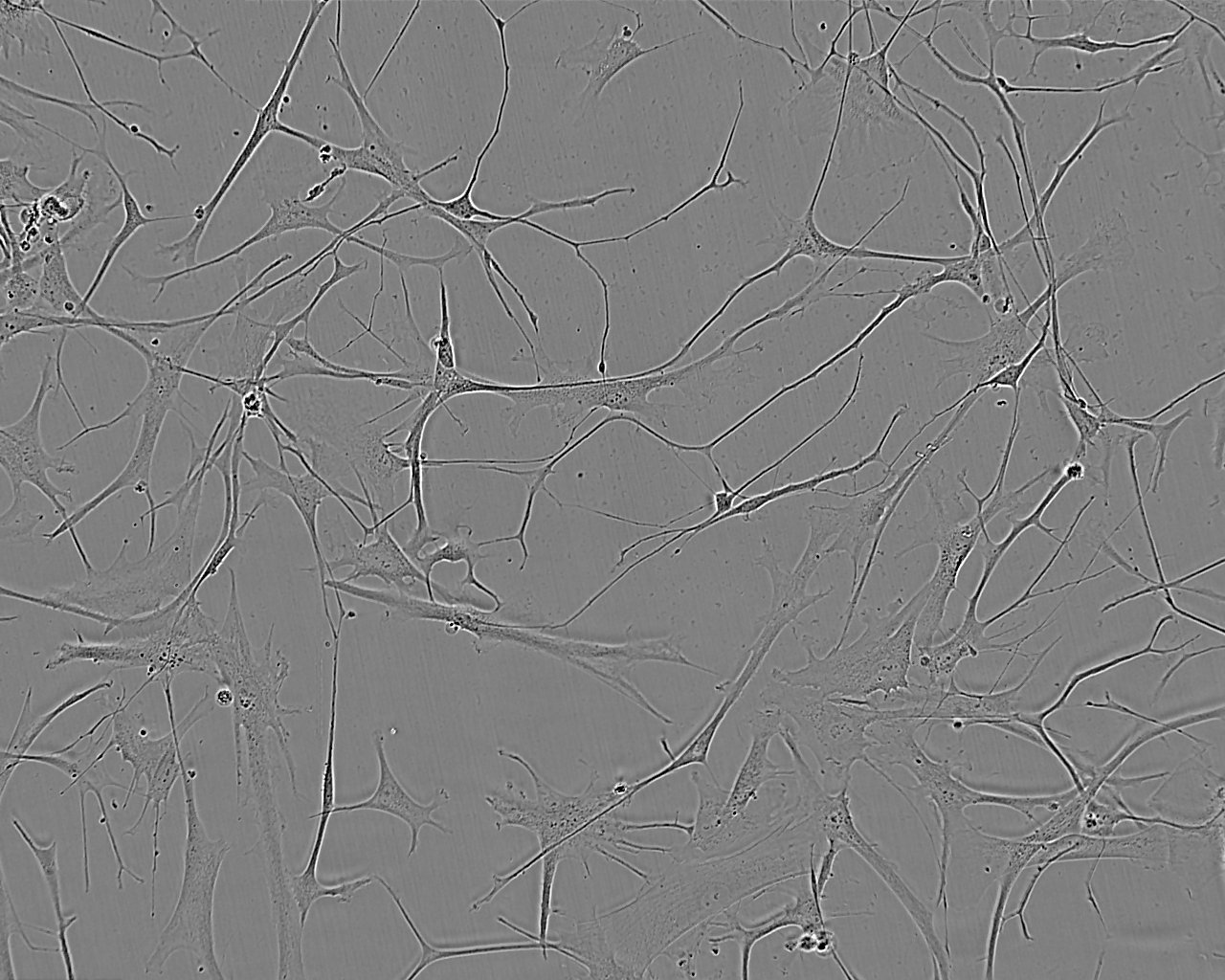

Supplement: Supplementary file 1 — Video microscopy movie 1. 13 frames of the time-lapse microscopy movie (.tif format) corresponding to normal astrocytes when treated with 3.5μM of SAHA alone. (ZIP 18,177 kb) [file 12859_2018_2458_MOESM1_ESM.zip › 1_J2_1_2017y06m30d_07h00m.tif]

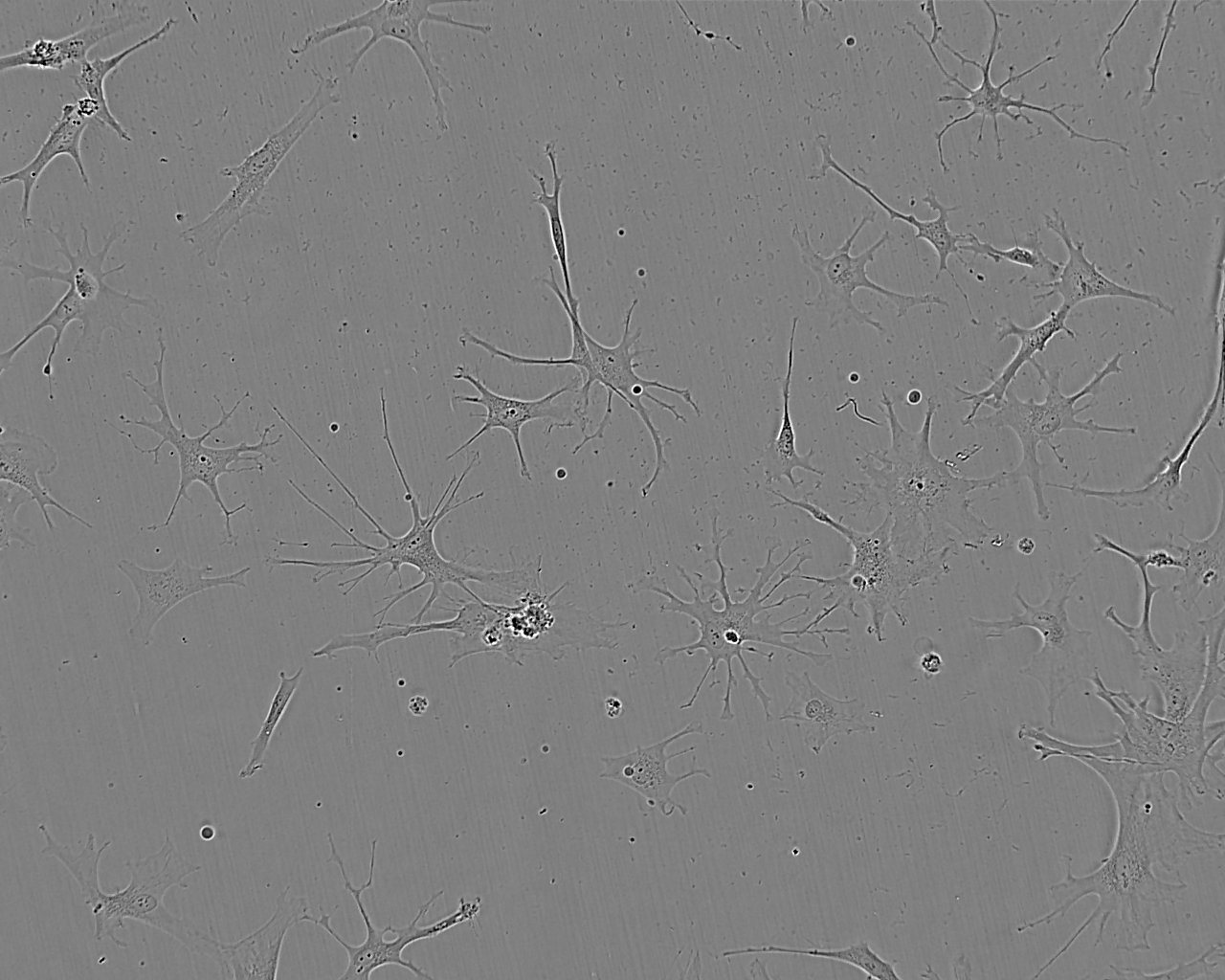

Supplement: Supplementary file 1 — Video microscopy movie 1. 13 frames of the time-lapse microscopy movie (.tif format) corresponding to normal astrocytes when treated with 3.5μM of SAHA alone. (ZIP 18,177 kb) [file 12859_2018_2458_MOESM1_ESM.zip › 1_J2_1_2017y06m27d_08h00m.tif]

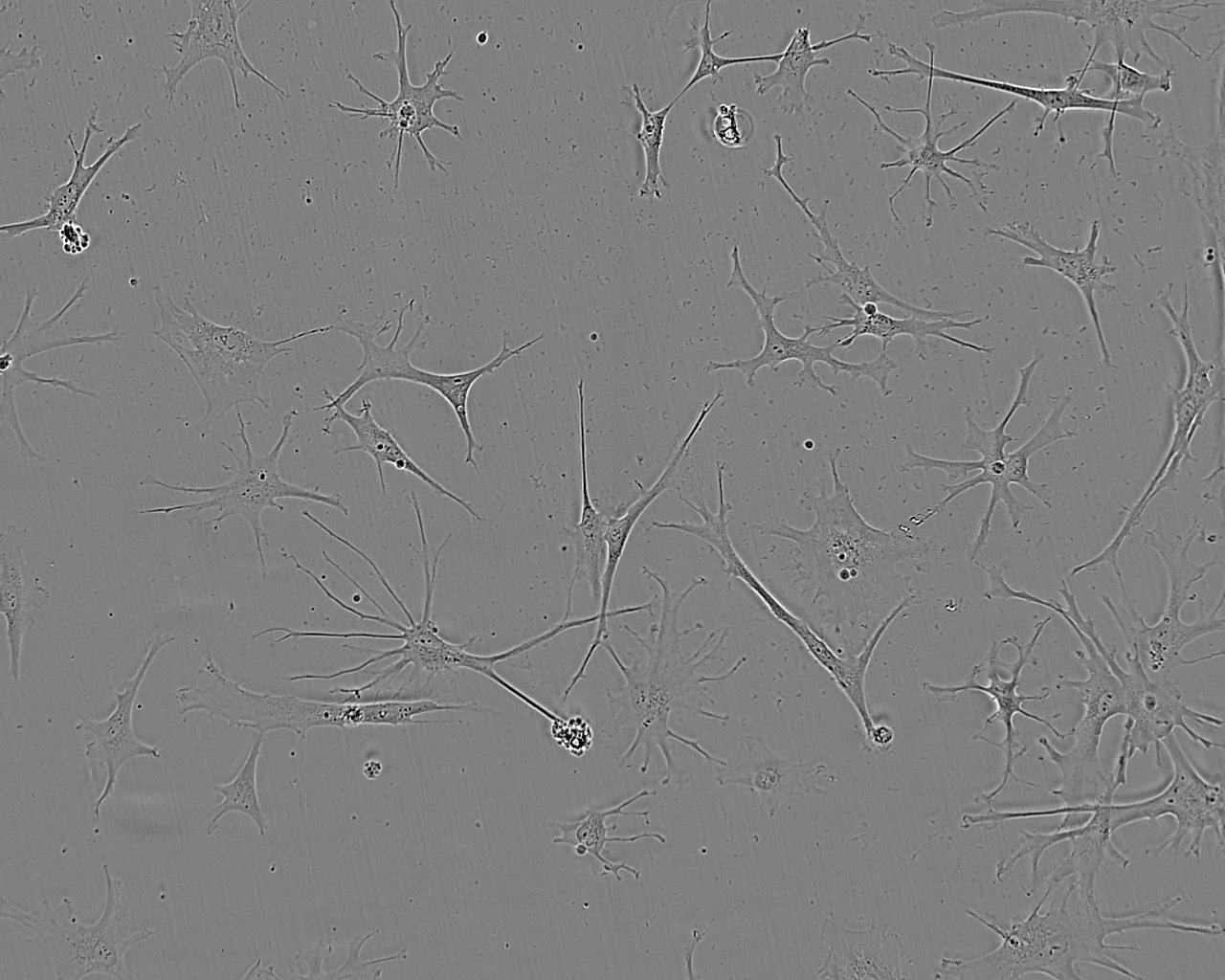

Supplement: Supplementary file 1 — Video microscopy movie 1. 13 frames of the time-lapse microscopy movie (.tif format) corresponding to normal astrocytes when treated with 3.5μM of SAHA alone. (ZIP 18,177 kb) [file 12859_2018_2458_MOESM1_ESM.zip › 1_J2_1_2017y06m27d_14h00m.tif]

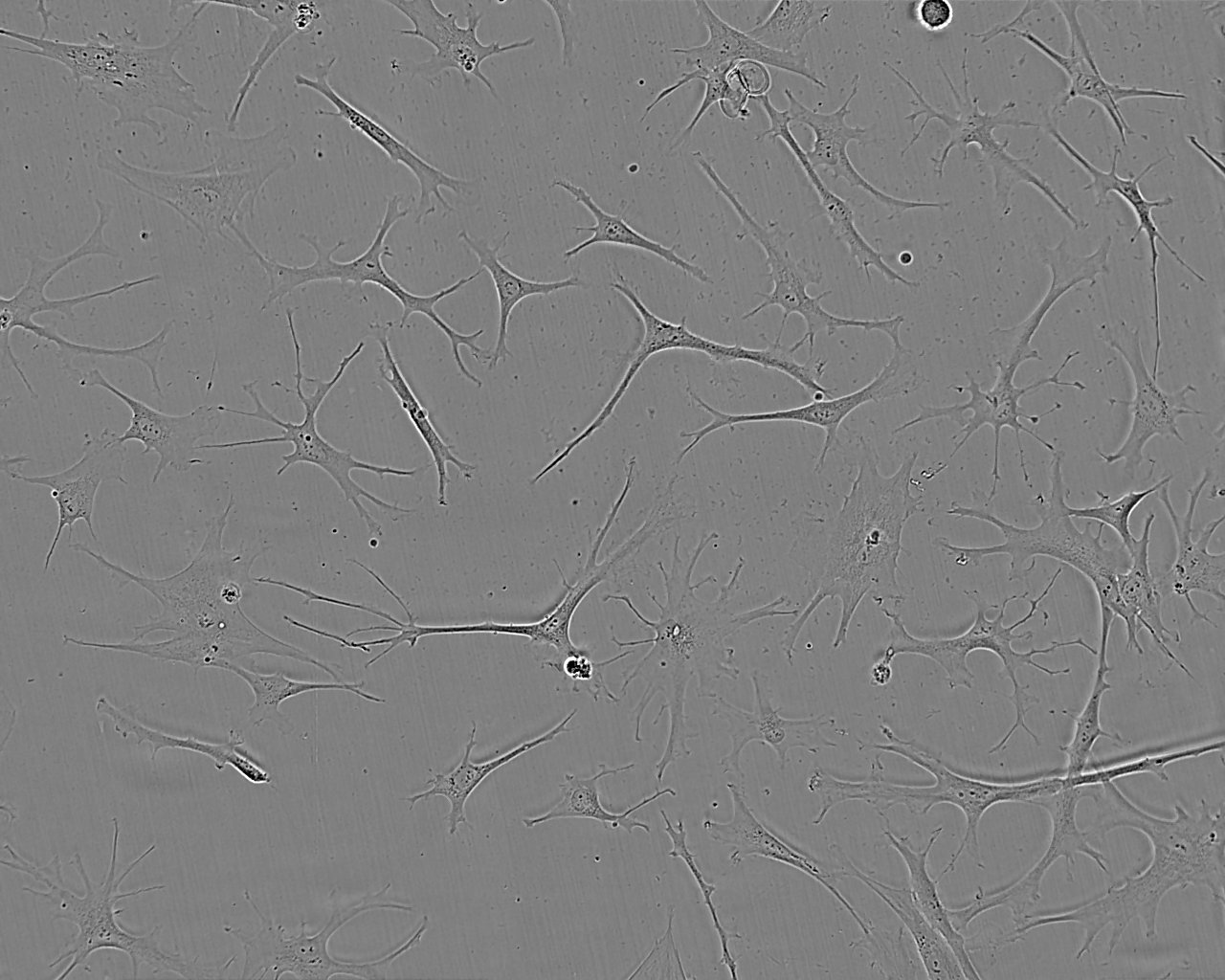

Supplement: Supplementary file 1 — Video microscopy movie 1. 13 frames of the time-lapse microscopy movie (.tif format) corresponding to normal astrocytes when treated with 3.5μM of SAHA alone. (ZIP 18,177 kb) [file 12859_2018_2458_MOESM1_ESM.zip › 1_J2_1_2017y06m27d_20h00m.tif]

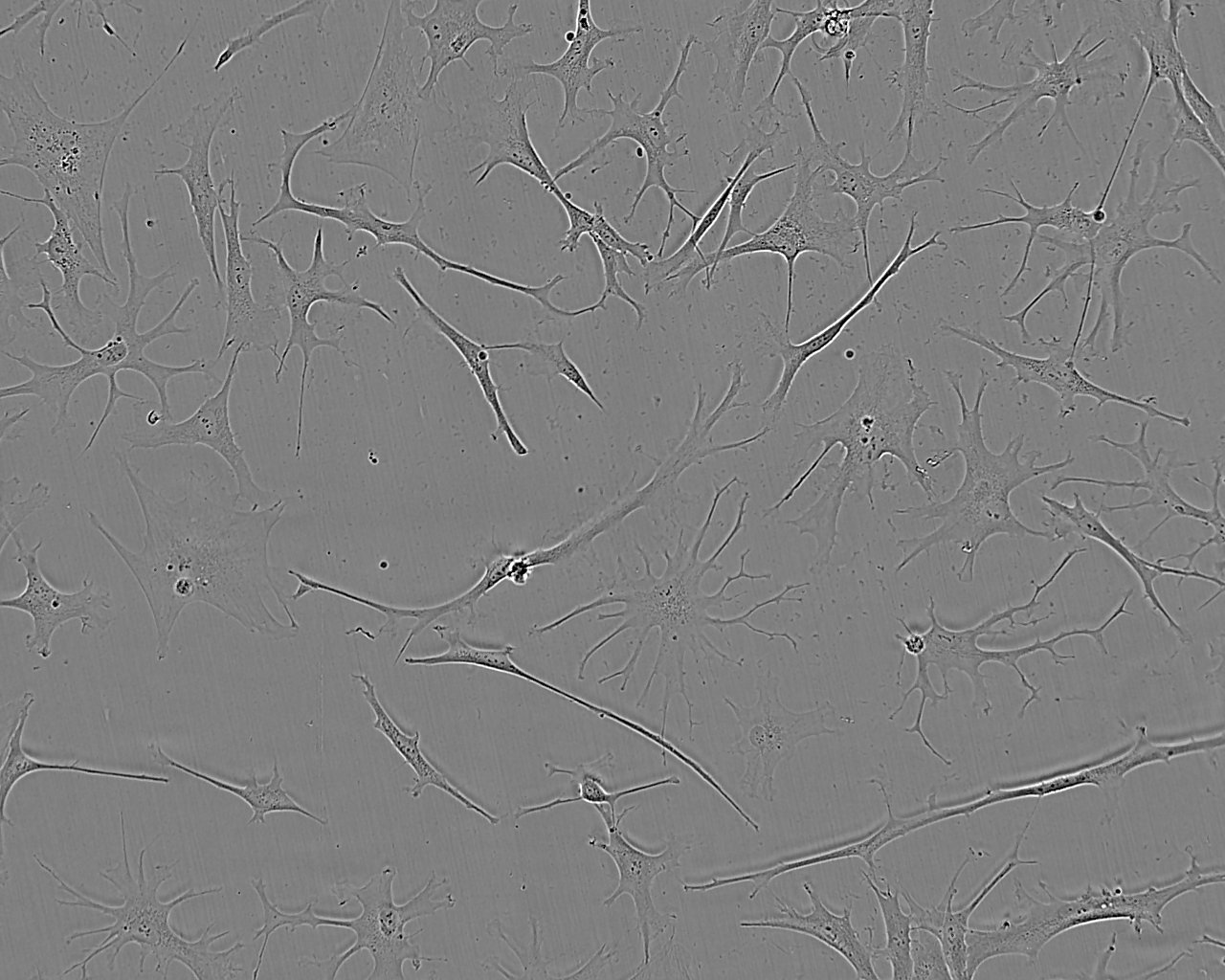

Supplement: Supplementary file 1 — Video microscopy movie 1. 13 frames of the time-lapse microscopy movie (.tif format) corresponding to normal astrocytes when treated with 3.5μM of SAHA alone. (ZIP 18,177 kb) [file 12859_2018_2458_MOESM1_ESM.zip › 1_J2_1_2017y06m28d_02h00m.tif]

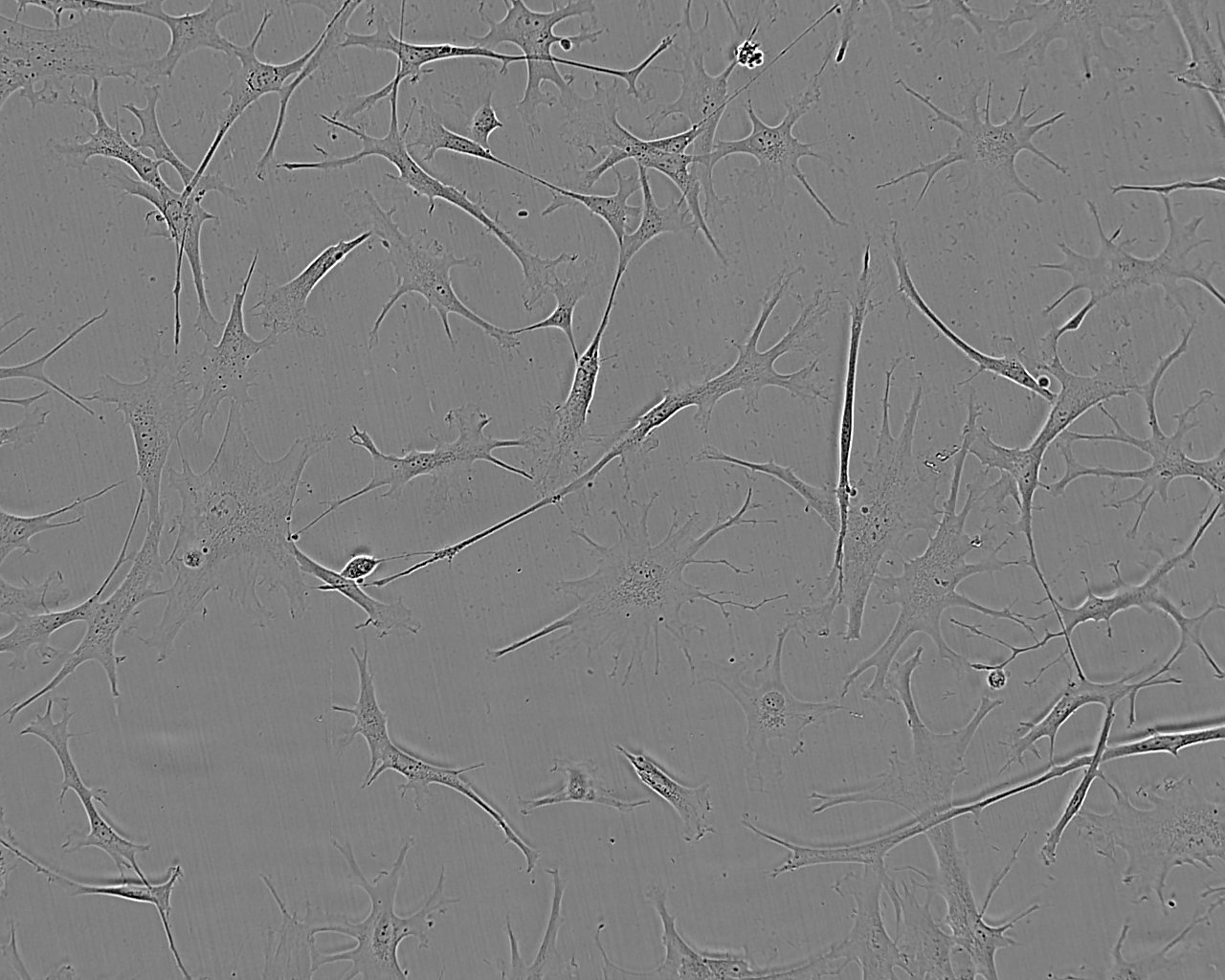

Supplement: Supplementary file 1 — Video microscopy movie 1. 13 frames of the time-lapse microscopy movie (.tif format) corresponding to normal astrocytes when treated with 3.5μM of SAHA alone. (ZIP 18,177 kb) [file 12859_2018_2458_MOESM1_ESM.zip › 1_J2_1_2017y06m28d_08h00m.tif]

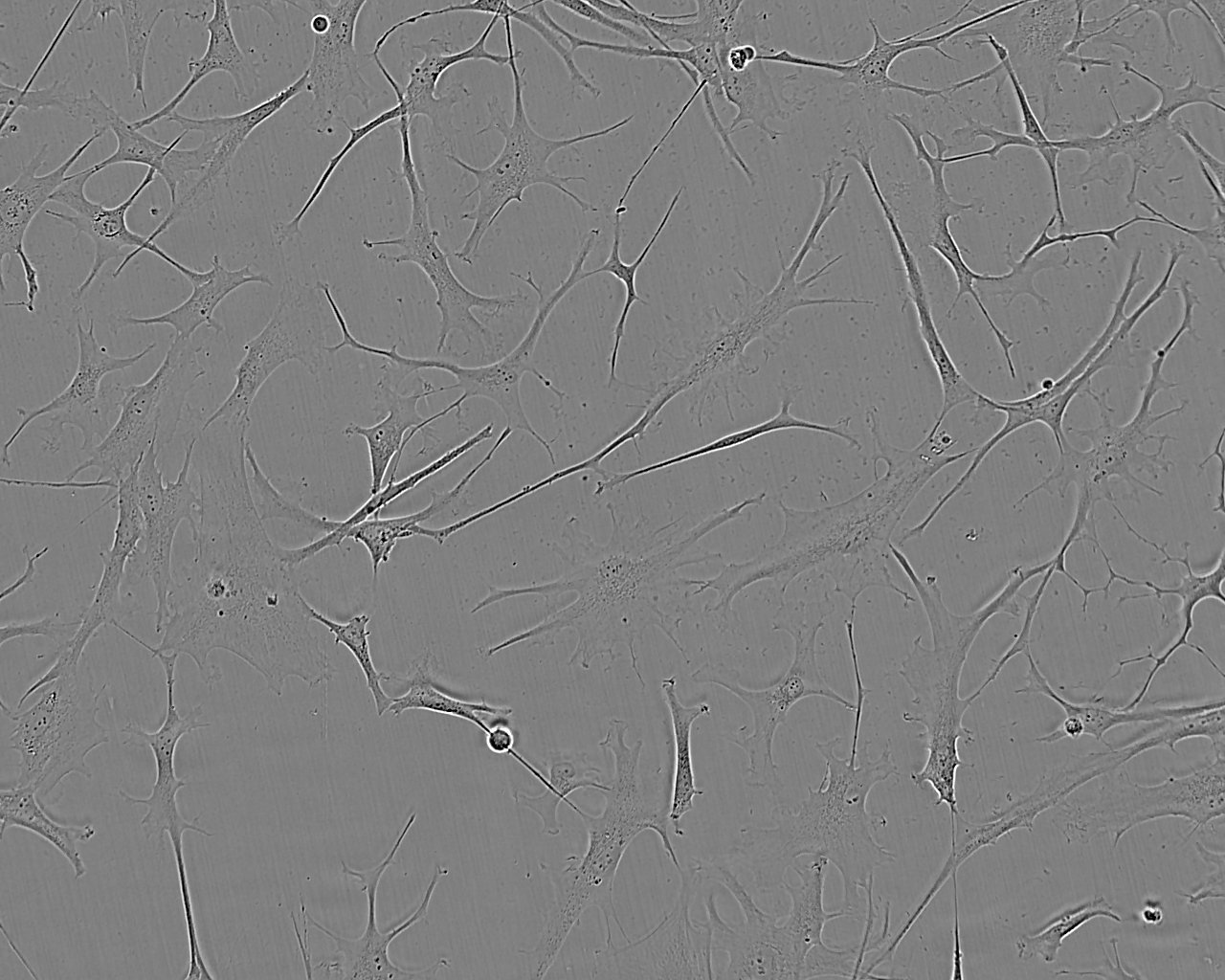

Supplement: Supplementary file 1 — Video microscopy movie 1. 13 frames of the time-lapse microscopy movie (.tif format) corresponding to normal astrocytes when treated with 3.5μM of SAHA alone. (ZIP 18,177 kb) [file 12859_2018_2458_MOESM1_ESM.zip › 1_J2_1_2017y06m28d_14h00m.tif]

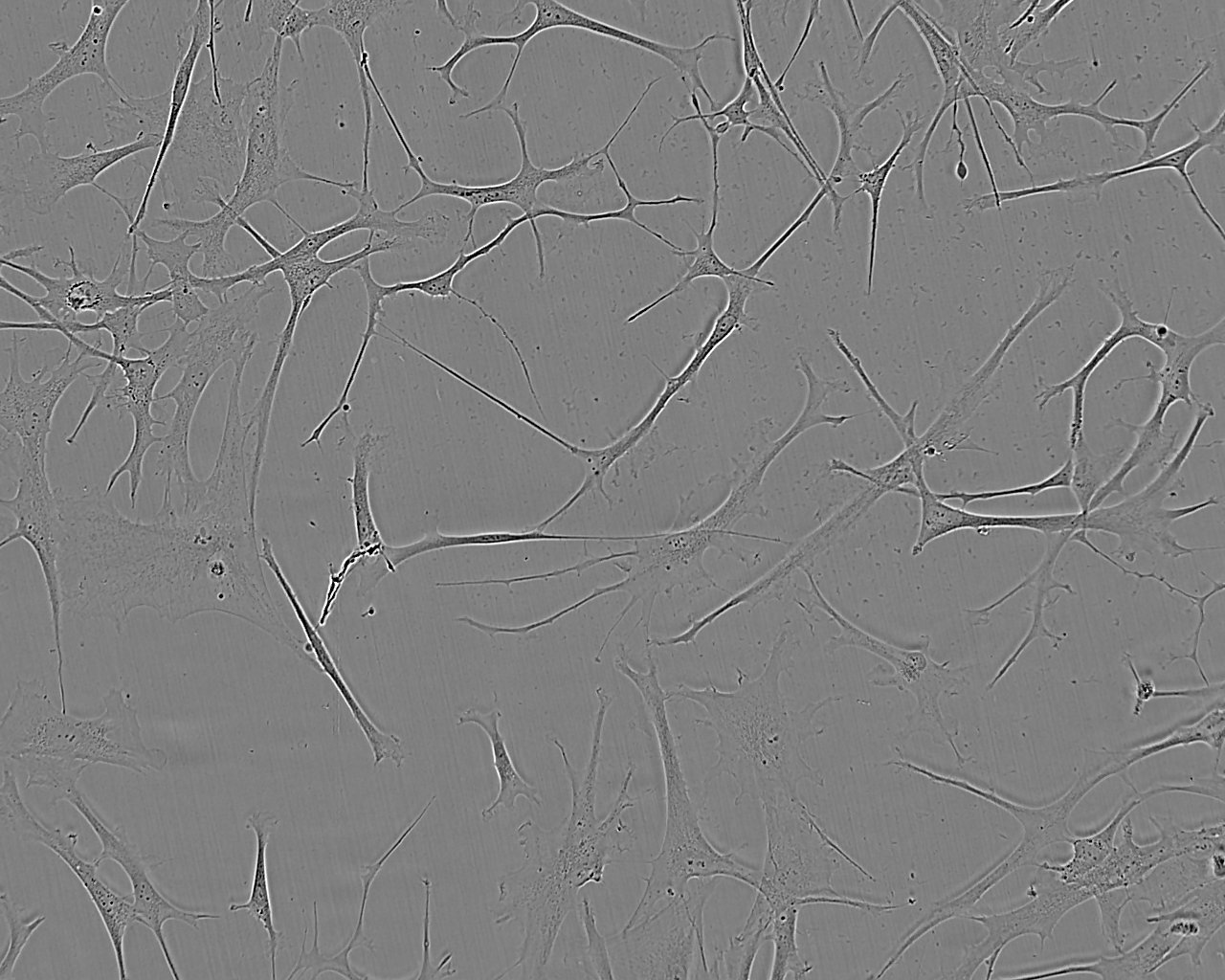

Supplement: Supplementary file 1 — Video microscopy movie 1. 13 frames of the time-lapse microscopy movie (.tif format) corresponding to normal astrocytes when treated with 3.5μM of SAHA alone. (ZIP 18,177 kb) [file 12859_2018_2458_MOESM1_ESM.zip › 1_J2_1_2017y06m28d_20h00m.tif]

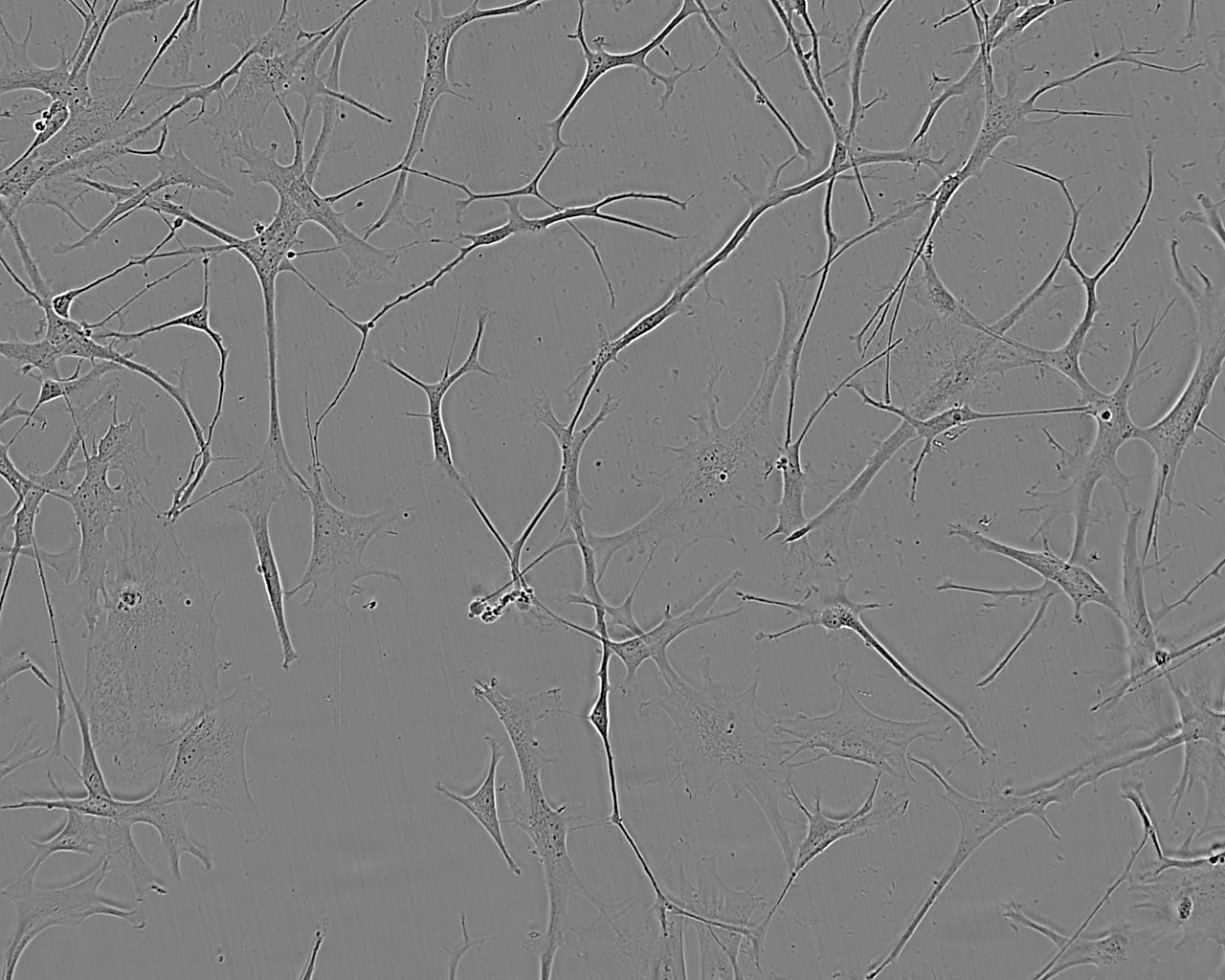

Supplement: Supplementary file 1 — Video microscopy movie 1. 13 frames of the time-lapse microscopy movie (.tif format) corresponding to normal astrocytes when treated with 3.5μM of SAHA alone. (ZIP 18,177 kb) [file 12859_2018_2458_MOESM1_ESM.zip › 1_J2_1_2017y06m29d_02h00m.tif]

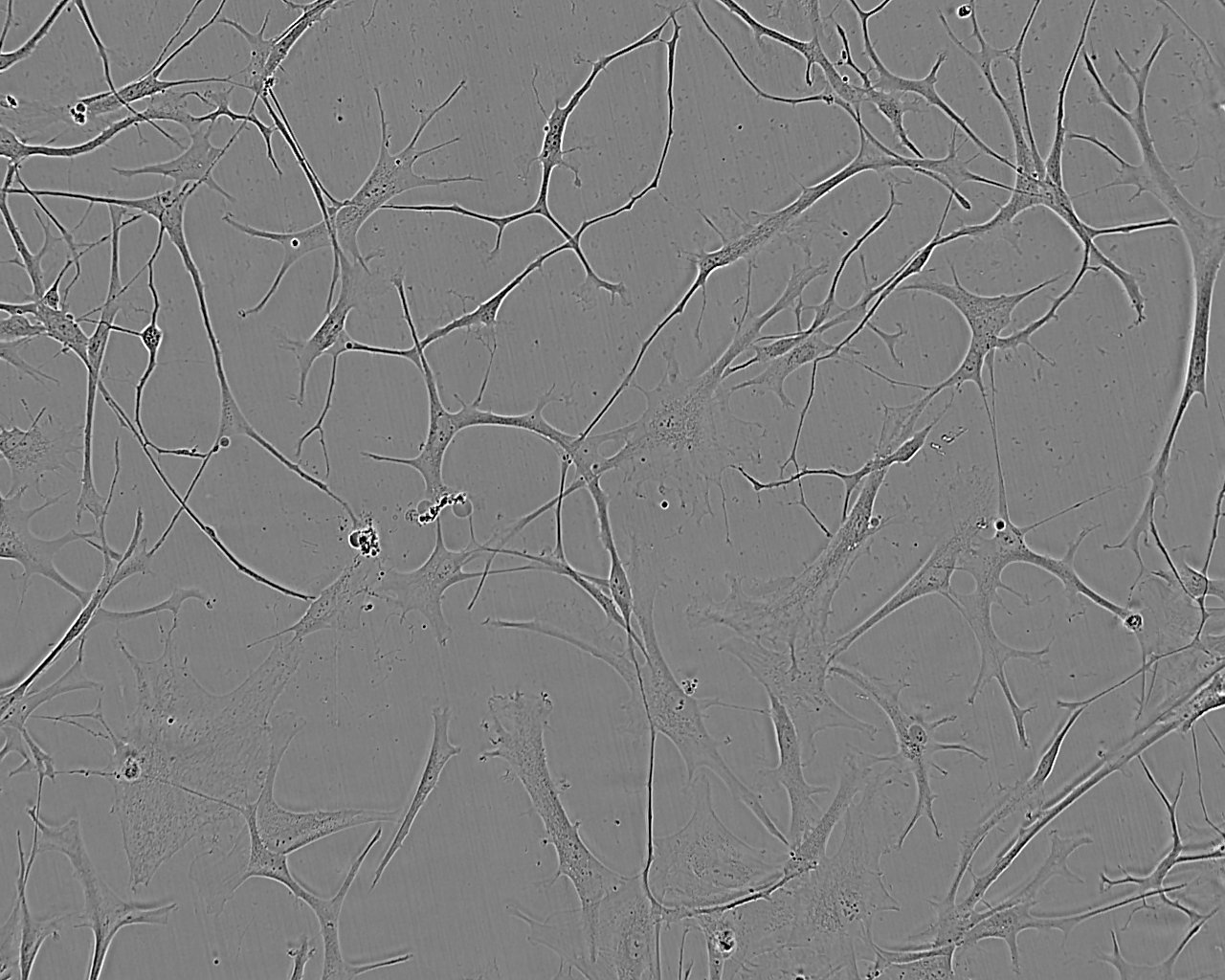

Supplement: Supplementary file 1 — Video microscopy movie 1. 13 frames of the time-lapse microscopy movie (.tif format) corresponding to normal astrocytes when treated with 3.5μM of SAHA alone. (ZIP 18,177 kb) [file 12859_2018_2458_MOESM1_ESM.zip › 1_J2_1_2017y06m29d_08h00m.tif]

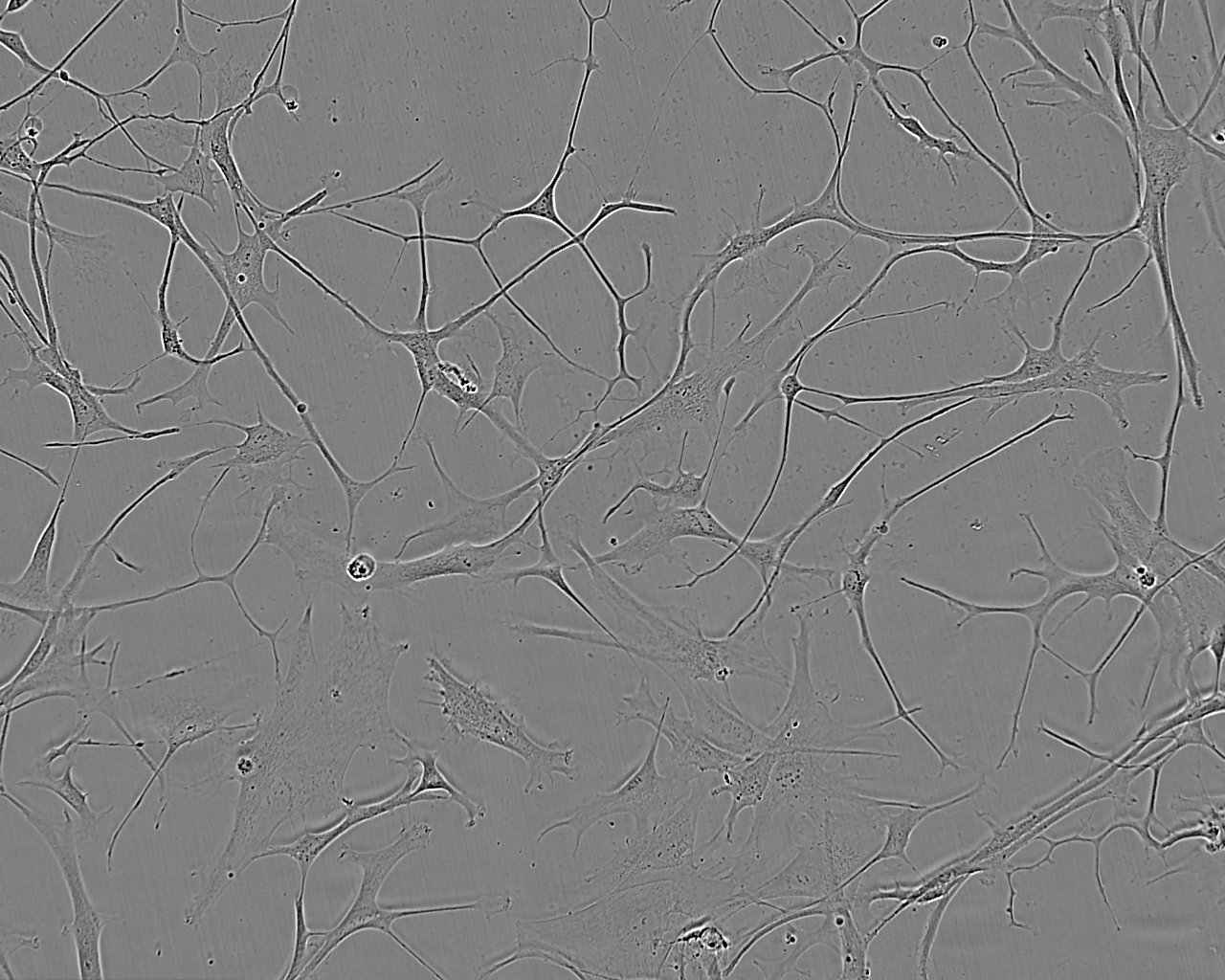

Supplement: Supplementary file 1 — Video microscopy movie 1. 13 frames of the time-lapse microscopy movie (.tif format) corresponding to normal astrocytes when treated with 3.5μM of SAHA alone. (ZIP 18,177 kb) [file 12859_2018_2458_MOESM1_ESM.zip › 1_J2_1_2017y06m29d_14h00m.tif]

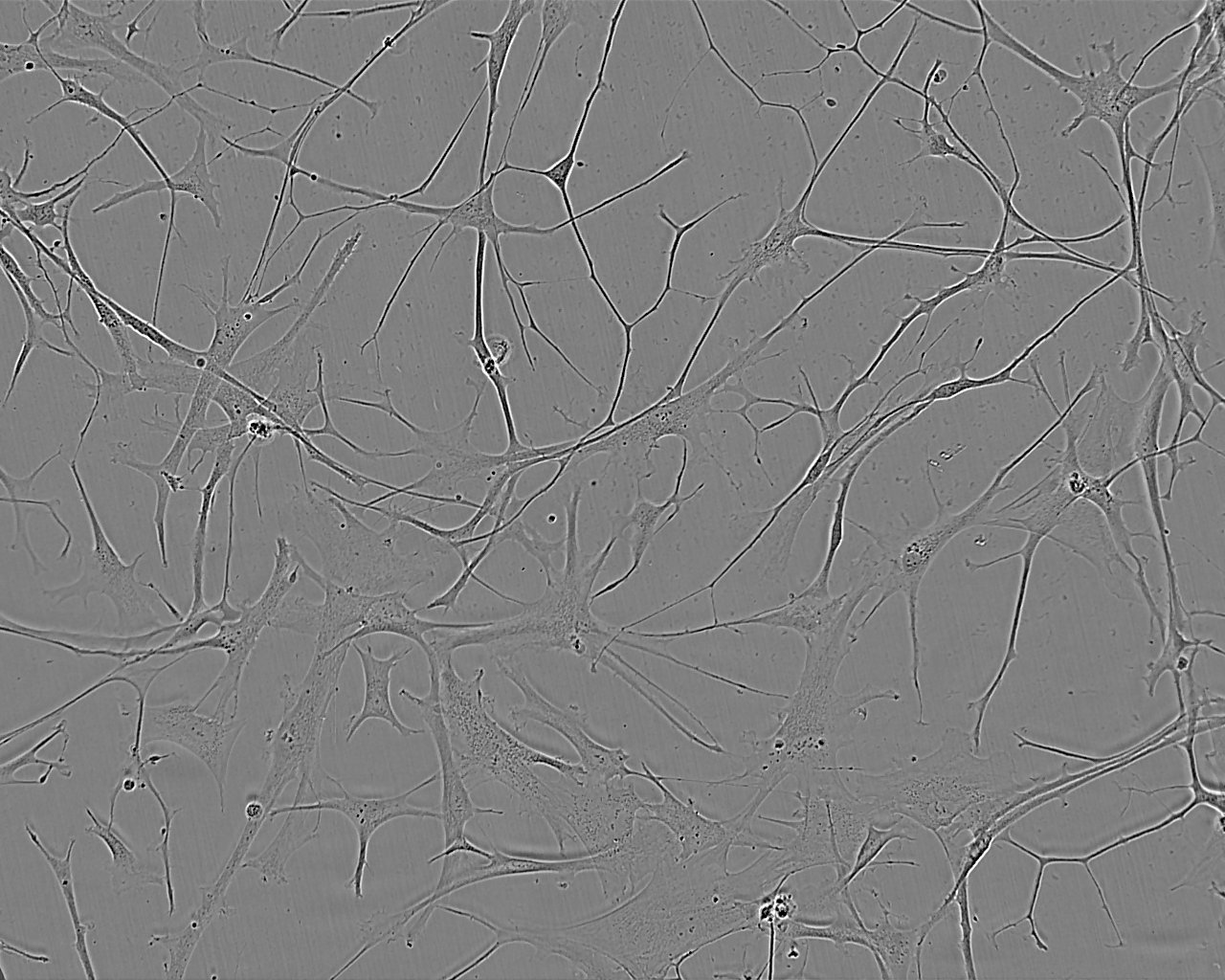

Supplement: Supplementary file 1 — Video microscopy movie 1. 13 frames of the time-lapse microscopy movie (.tif format) corresponding to normal astrocytes when treated with 3.5μM of SAHA alone. (ZIP 18,177 kb) [file 12859_2018_2458_MOESM1_ESM.zip › 1_J2_1_2017y06m29d_20h00m.tif]

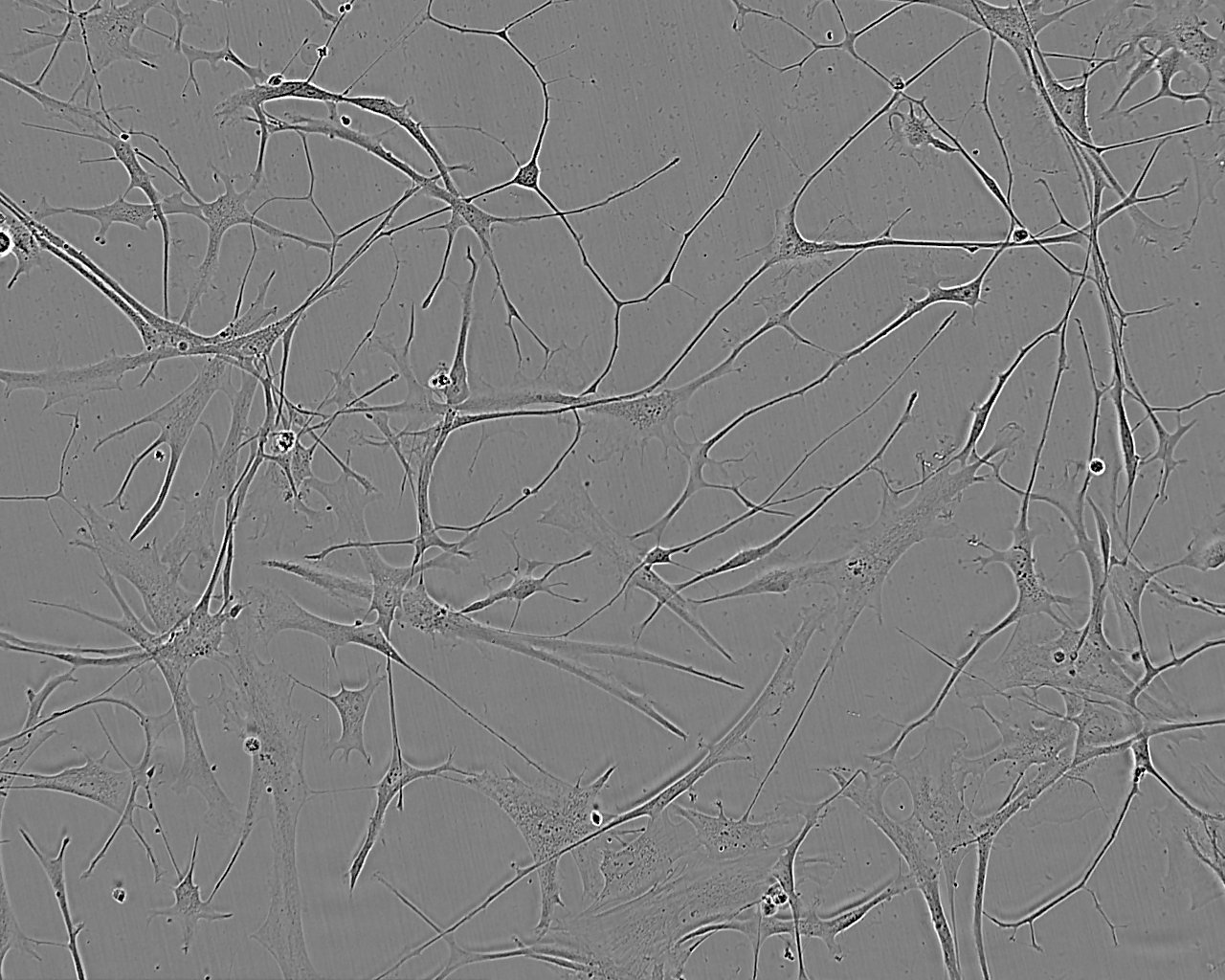

Supplement: Supplementary file 1 — Video microscopy movie 1. 13 frames of the time-lapse microscopy movie (.tif format) corresponding to normal astrocytes when treated with 3.5μM of SAHA alone. (ZIP 18,177 kb) [file 12859_2018_2458_MOESM1_ESM.zip › 1_J2_1_2017y06m30d_02h00m.tif]

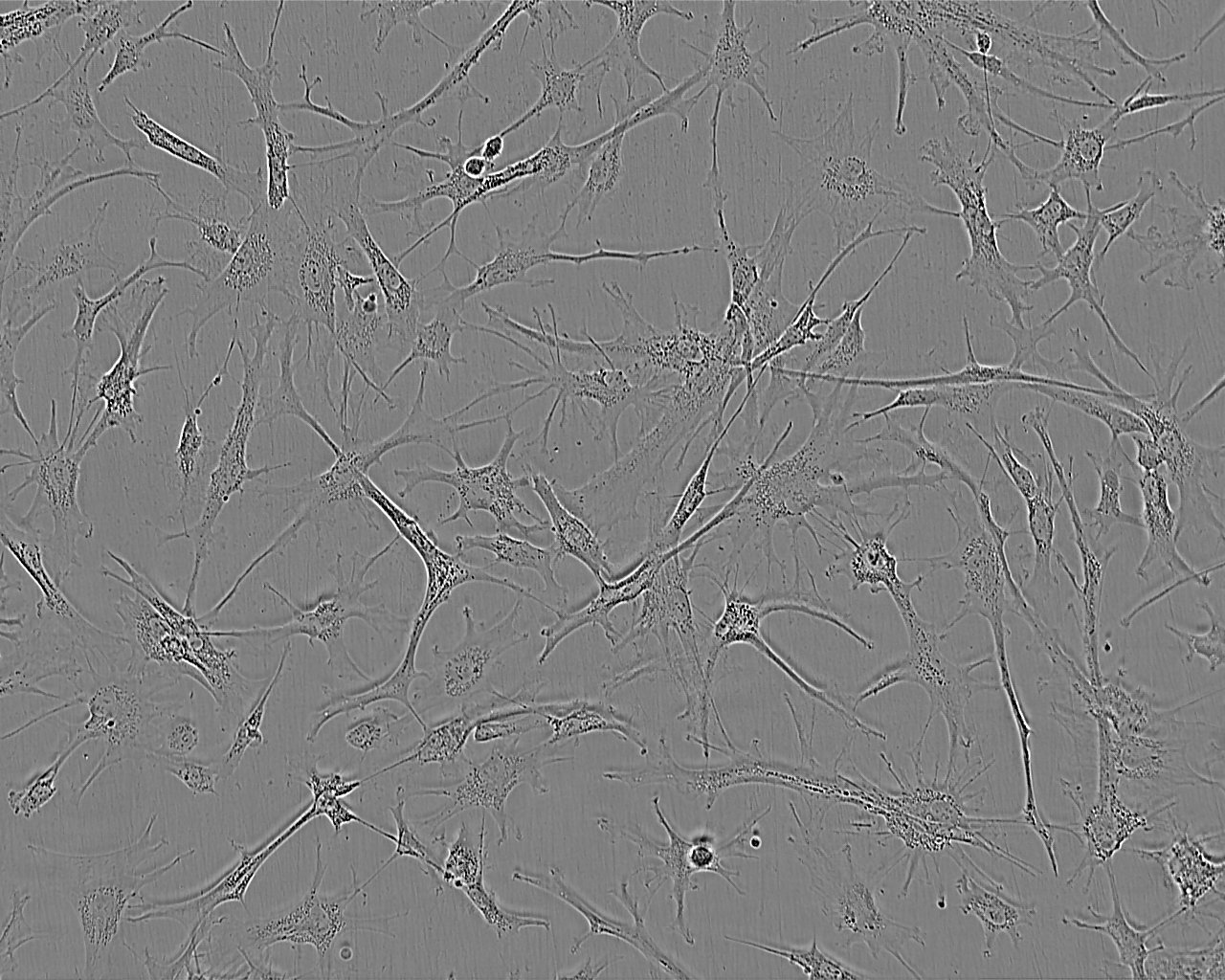

Supplement: Supplementary file 2 — Video microscopy movie 2. 13 frames of the time-lapse microscopy movie (.tif format) corresponding to normal astrocytes when treated with 2μM of CPD-1 alone. (ZIP 20,242 kb) [file 12859_2018_2458_MOESM2_ESM.zip › 1_N18_1_2017y06m30d_07h00m.tif]

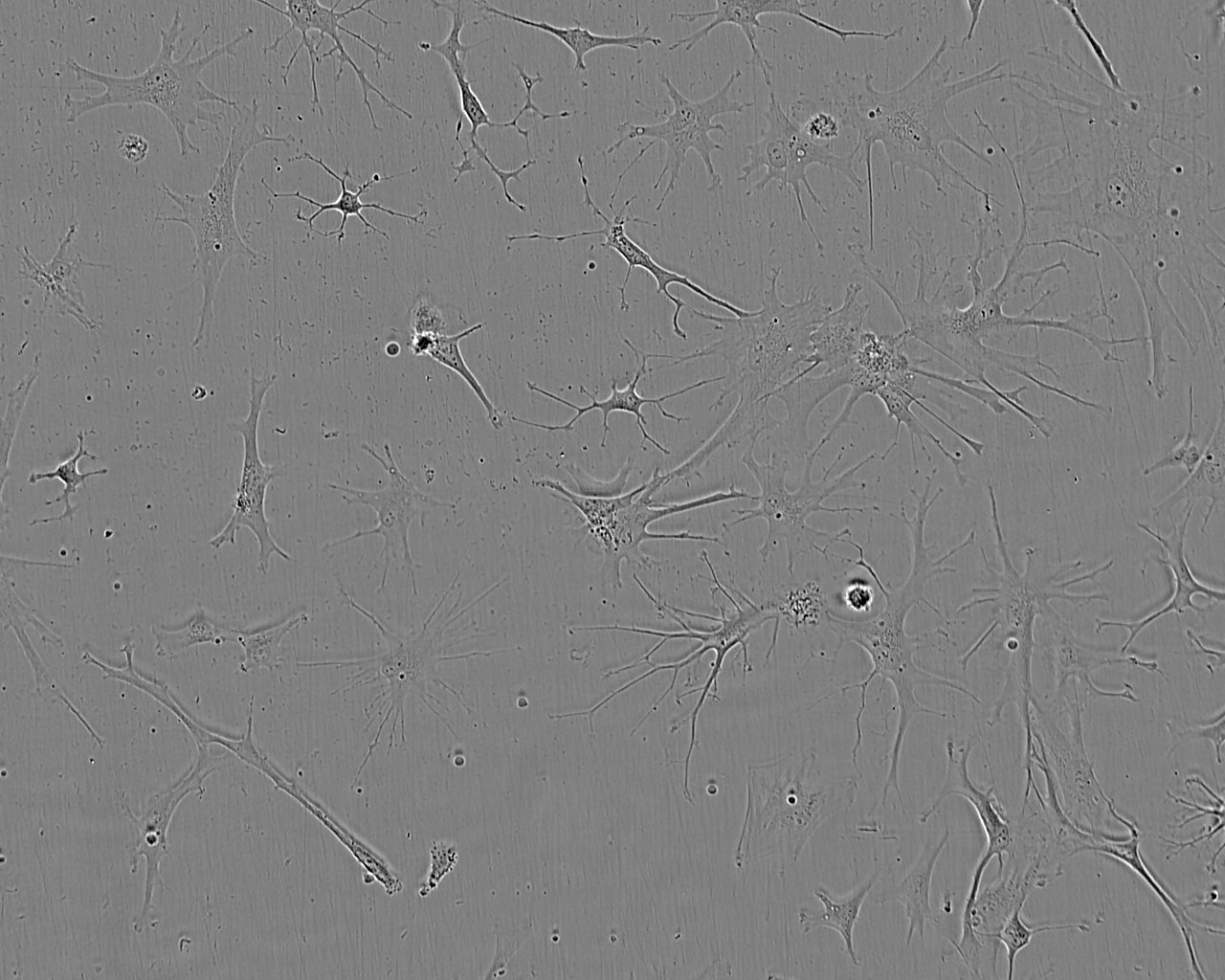

Supplement: Supplementary file 2 — Video microscopy movie 2. 13 frames of the time-lapse microscopy movie (.tif format) corresponding to normal astrocytes when treated with 2μM of CPD-1 alone. (ZIP 20,242 kb) [file 12859_2018_2458_MOESM2_ESM.zip › 1_N18_1_2017y06m27d_08h00m.tif]

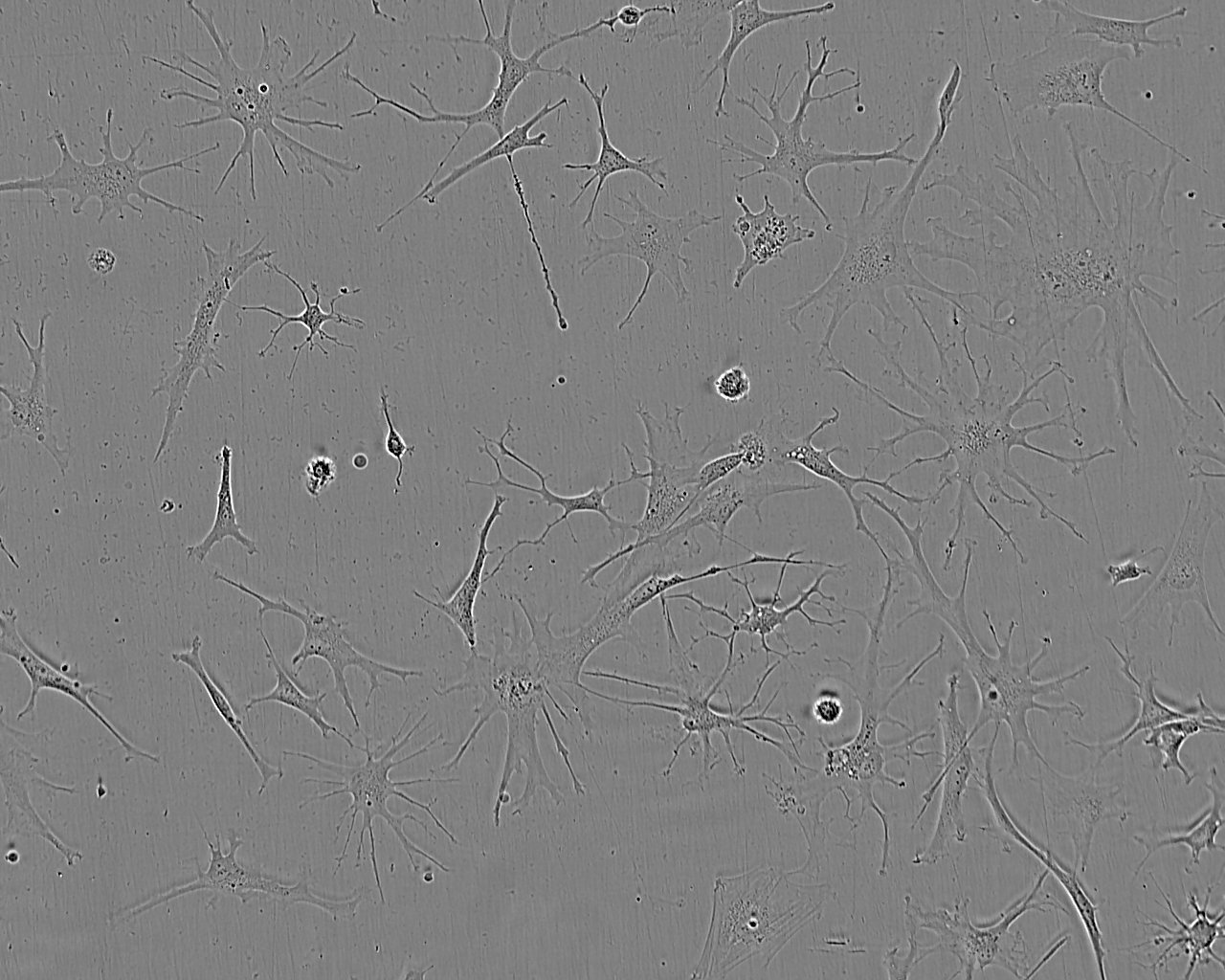

Supplement: Supplementary file 2 — Video microscopy movie 2. 13 frames of the time-lapse microscopy movie (.tif format) corresponding to normal astrocytes when treated with 2μM of CPD-1 alone. (ZIP 20,242 kb) [file 12859_2018_2458_MOESM2_ESM.zip › 1_N18_1_2017y06m27d_14h00m.tif]

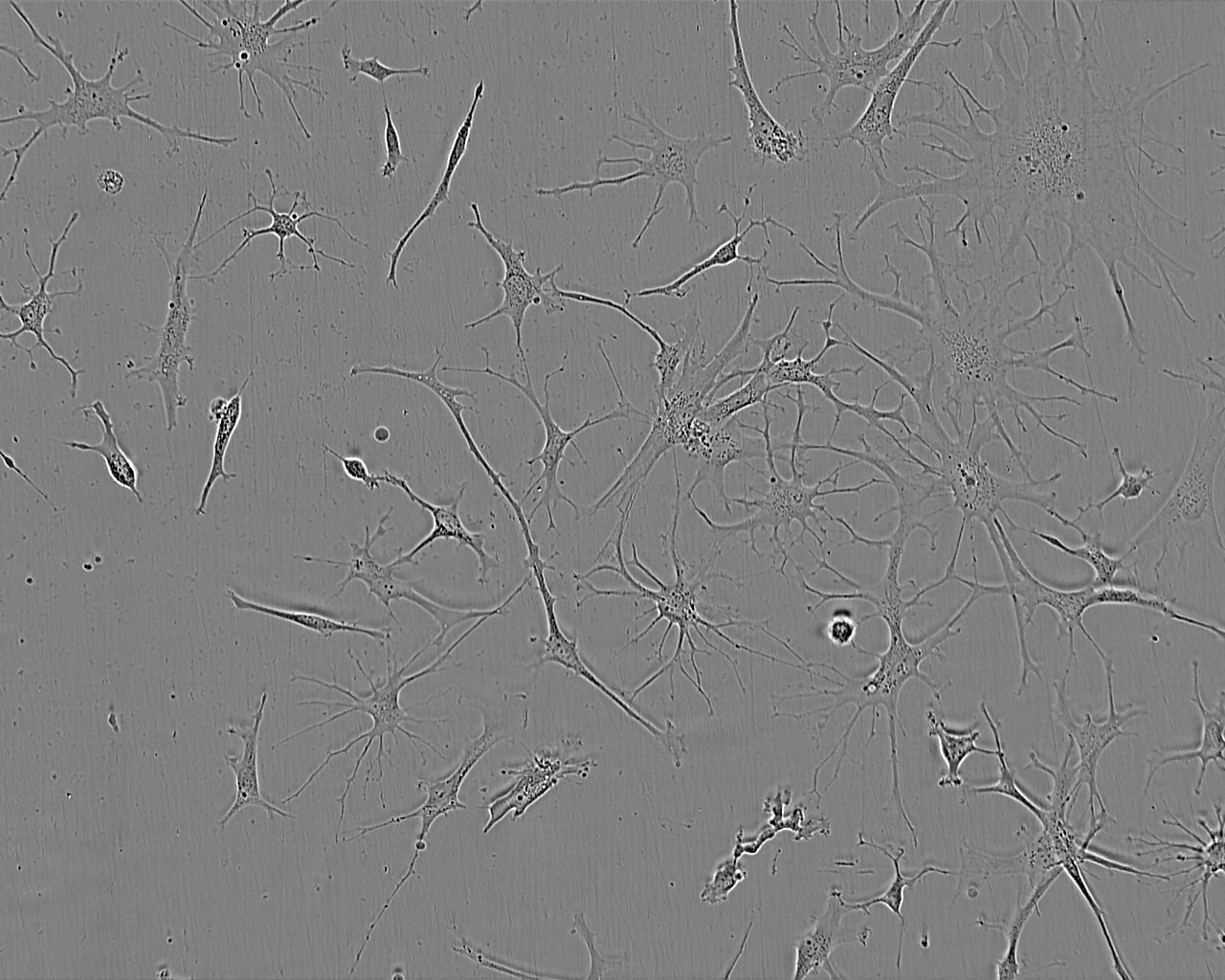

Supplement: Supplementary file 2 — Video microscopy movie 2. 13 frames of the time-lapse microscopy movie (.tif format) corresponding to normal astrocytes when treated with 2μM of CPD-1 alone. (ZIP 20,242 kb) [file 12859_2018_2458_MOESM2_ESM.zip › 1_N18_1_2017y06m27d_20h00m.tif]

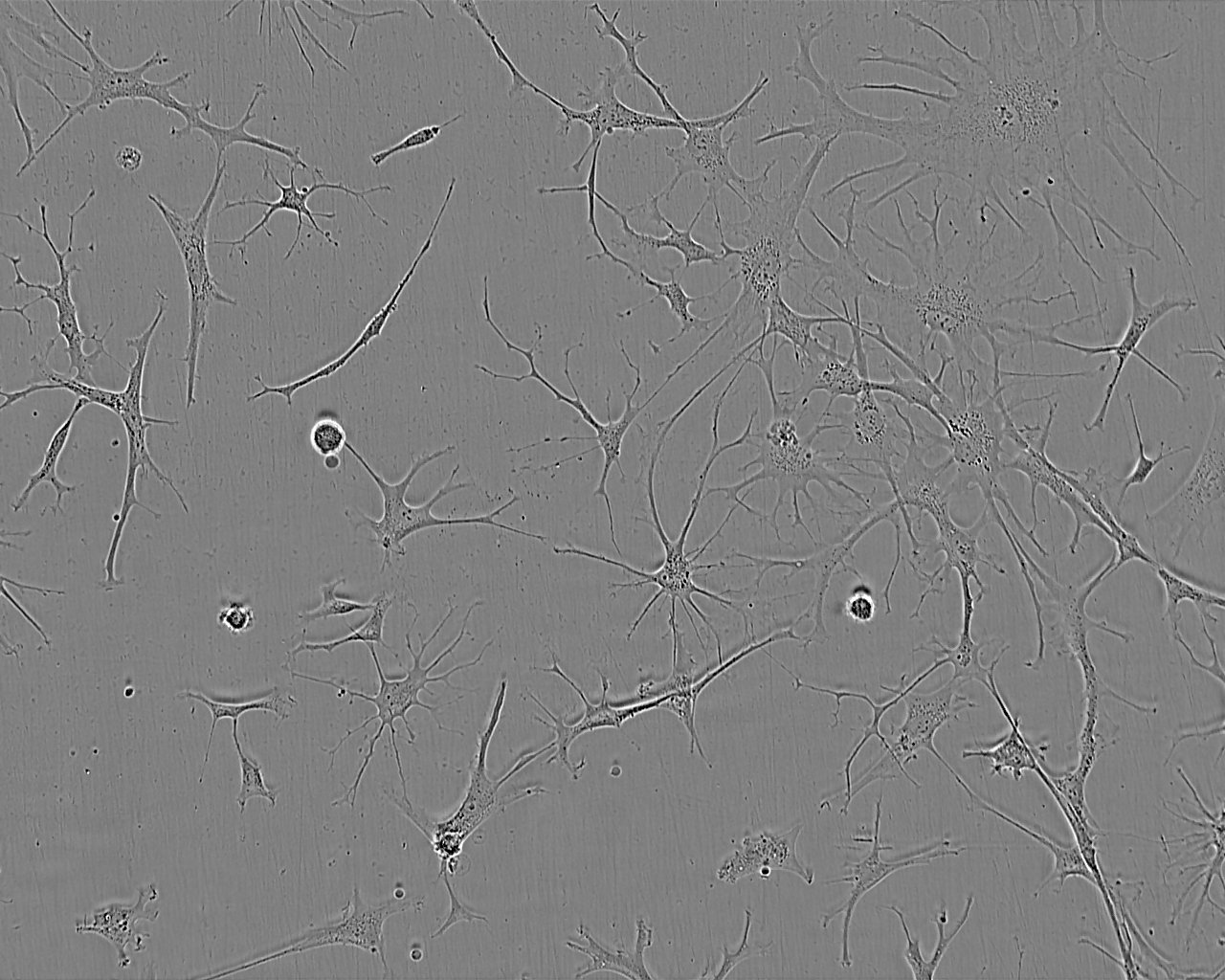

Supplement: Supplementary file 2 — Video microscopy movie 2. 13 frames of the time-lapse microscopy movie (.tif format) corresponding to normal astrocytes when treated with 2μM of CPD-1 alone. (ZIP 20,242 kb) [file 12859_2018_2458_MOESM2_ESM.zip › 1_N18_1_2017y06m28d_02h00m.tif]

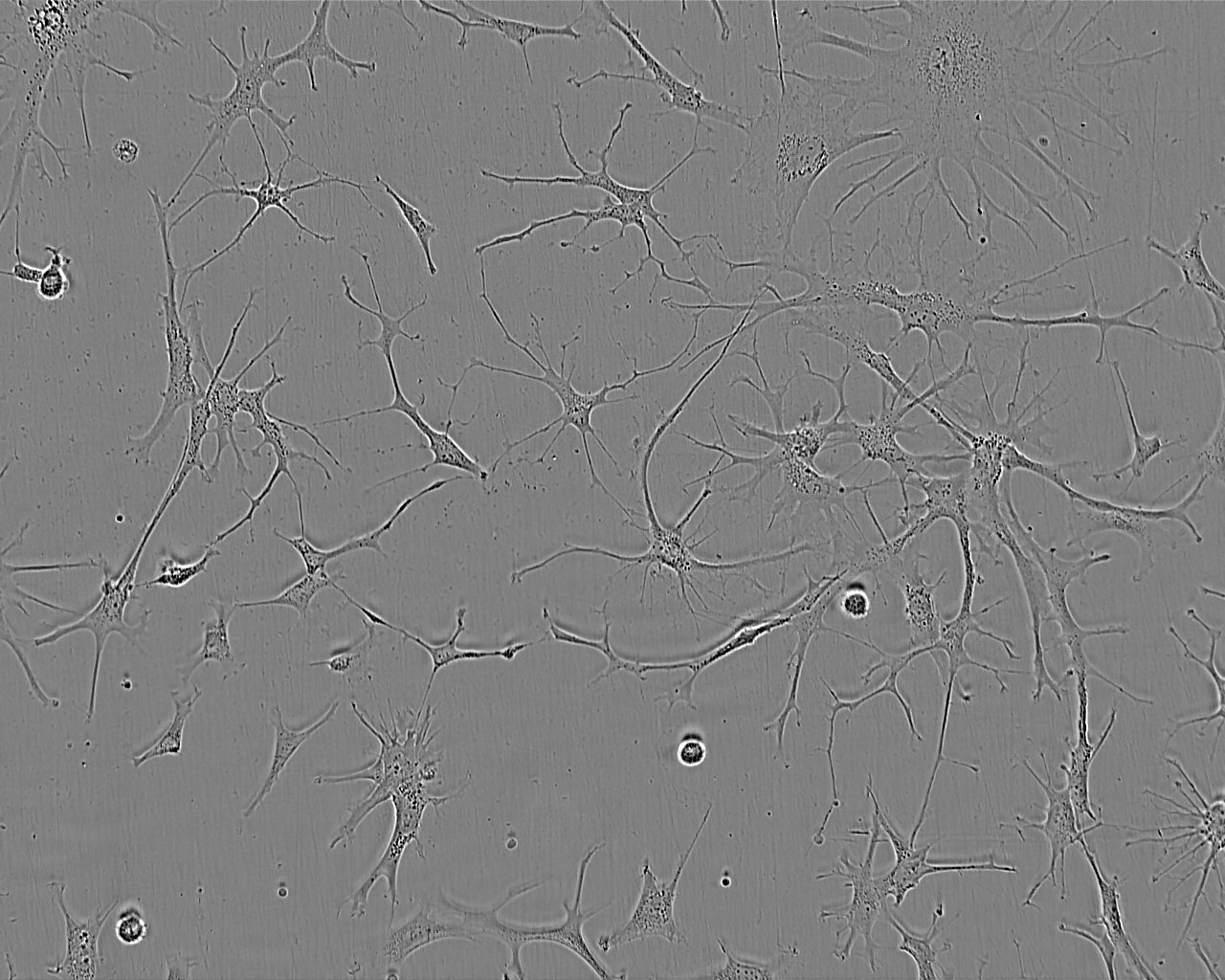

Supplement: Supplementary file 2 — Video microscopy movie 2. 13 frames of the time-lapse microscopy movie (.tif format) corresponding to normal astrocytes when treated with 2μM of CPD-1 alone. (ZIP 20,242 kb) [file 12859_2018_2458_MOESM2_ESM.zip › 1_N18_1_2017y06m28d_08h00m.tif]

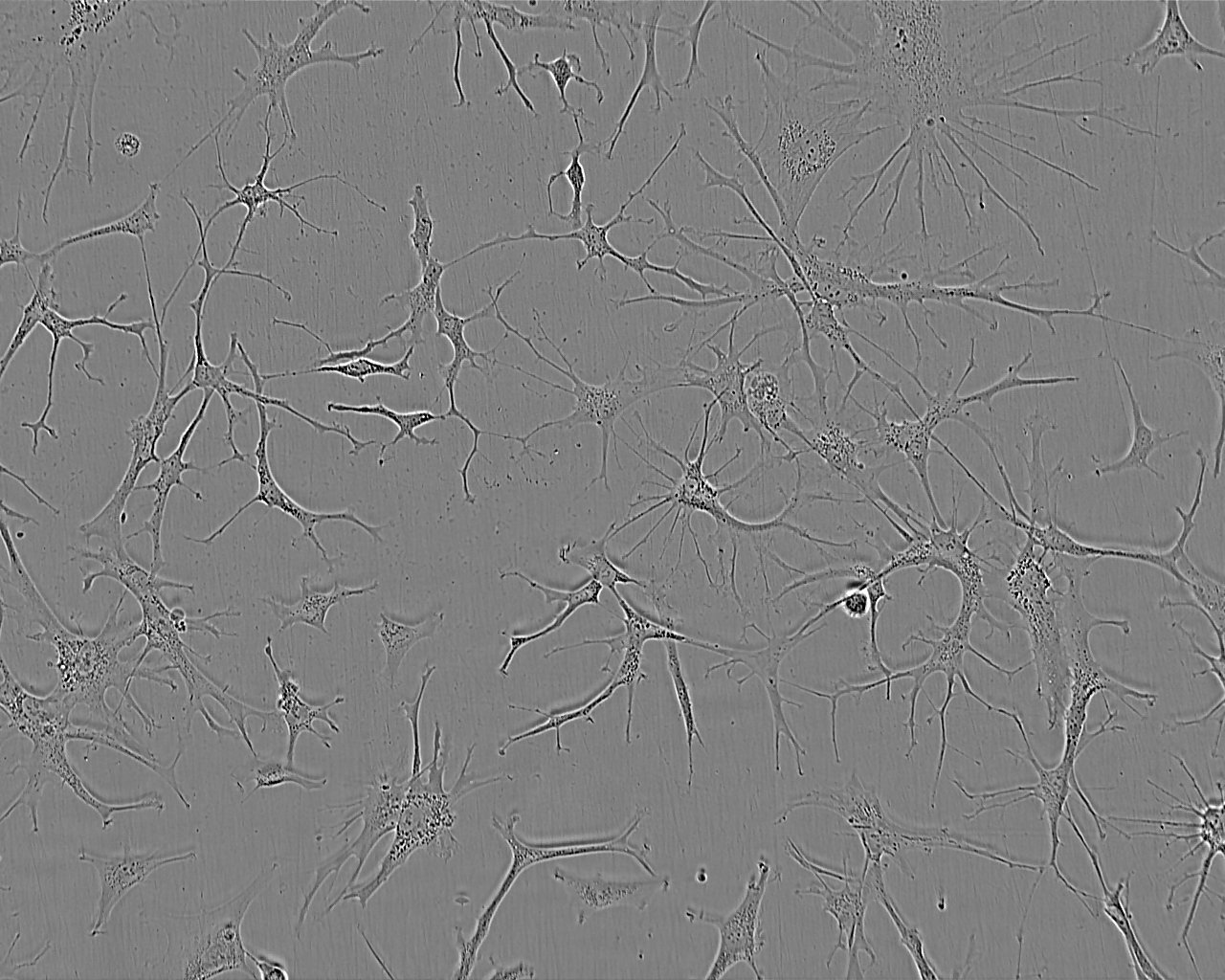

Supplement: Supplementary file 2 — Video microscopy movie 2. 13 frames of the time-lapse microscopy movie (.tif format) corresponding to normal astrocytes when treated with 2μM of CPD-1 alone. (ZIP 20,242 kb) [file 12859_2018_2458_MOESM2_ESM.zip › 1_N18_1_2017y06m28d_14h00m.tif]

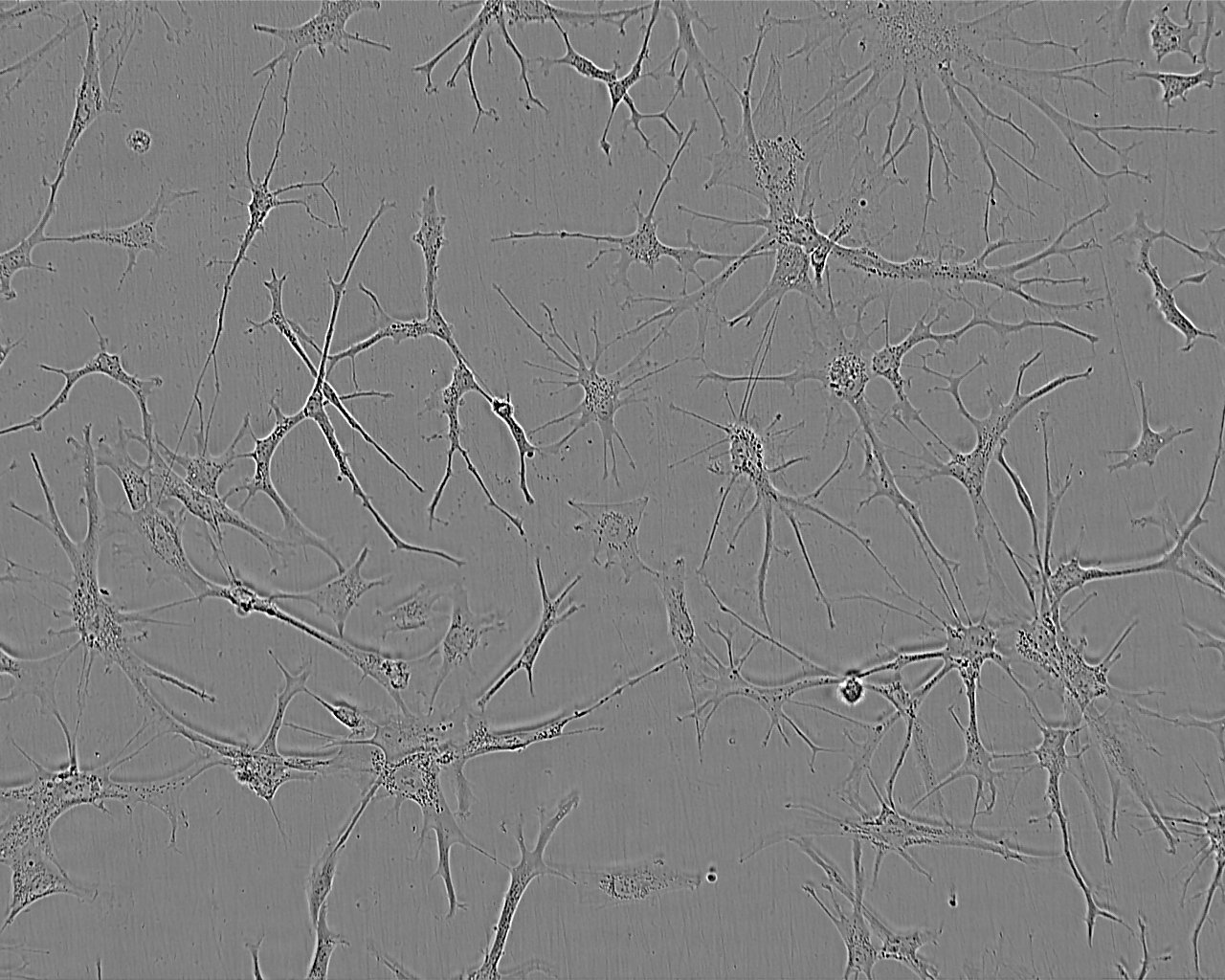

Supplement: Supplementary file 2 — Video microscopy movie 2. 13 frames of the time-lapse microscopy movie (.tif format) corresponding to normal astrocytes when treated with 2μM of CPD-1 alone. (ZIP 20,242 kb) [file 12859_2018_2458_MOESM2_ESM.zip › 1_N18_1_2017y06m28d_20h00m.tif]

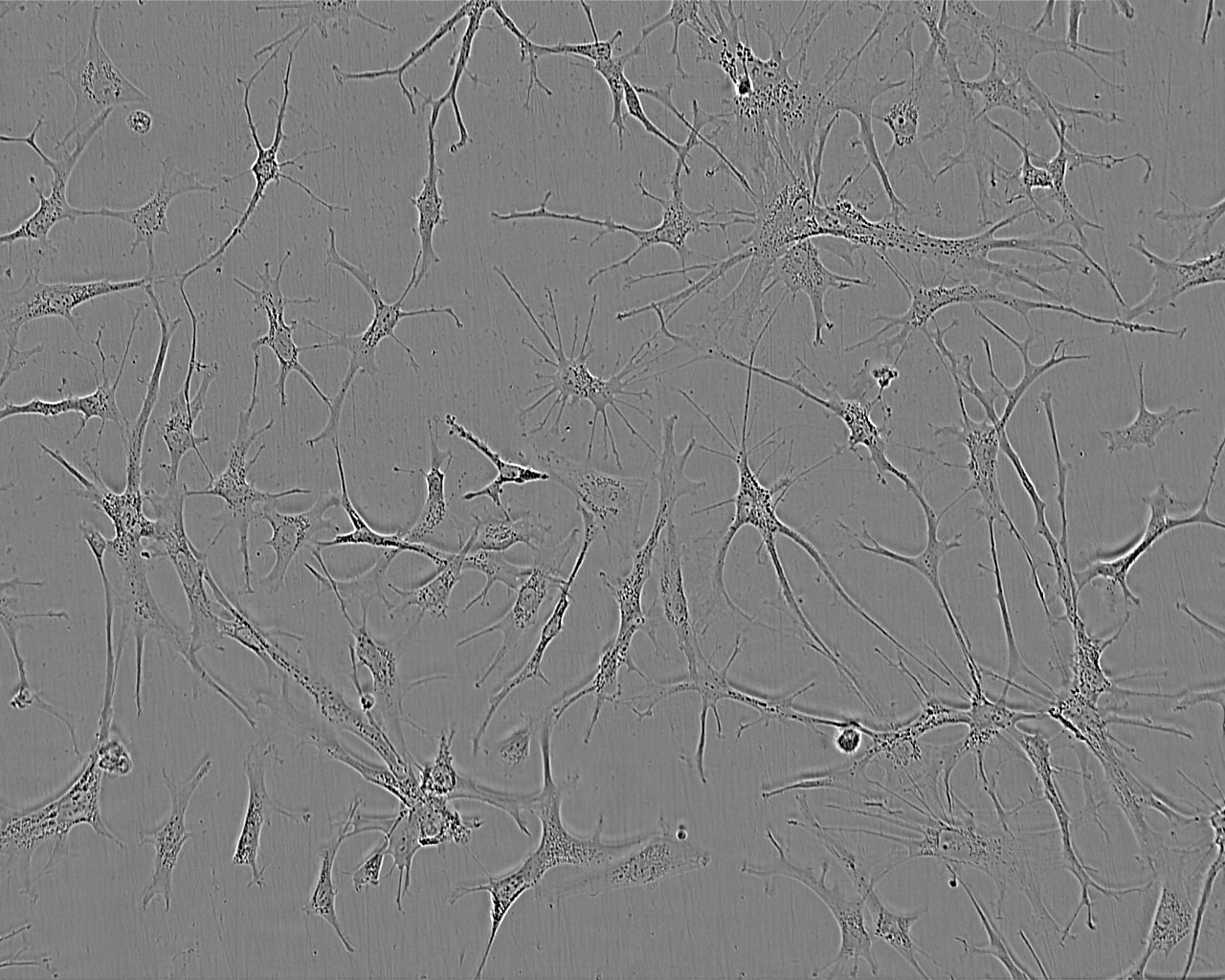

Supplement: Supplementary file 2 — Video microscopy movie 2. 13 frames of the time-lapse microscopy movie (.tif format) corresponding to normal astrocytes when treated with 2μM of CPD-1 alone. (ZIP 20,242 kb) [file 12859_2018_2458_MOESM2_ESM.zip › 1_N18_1_2017y06m29d_02h00m.tif]

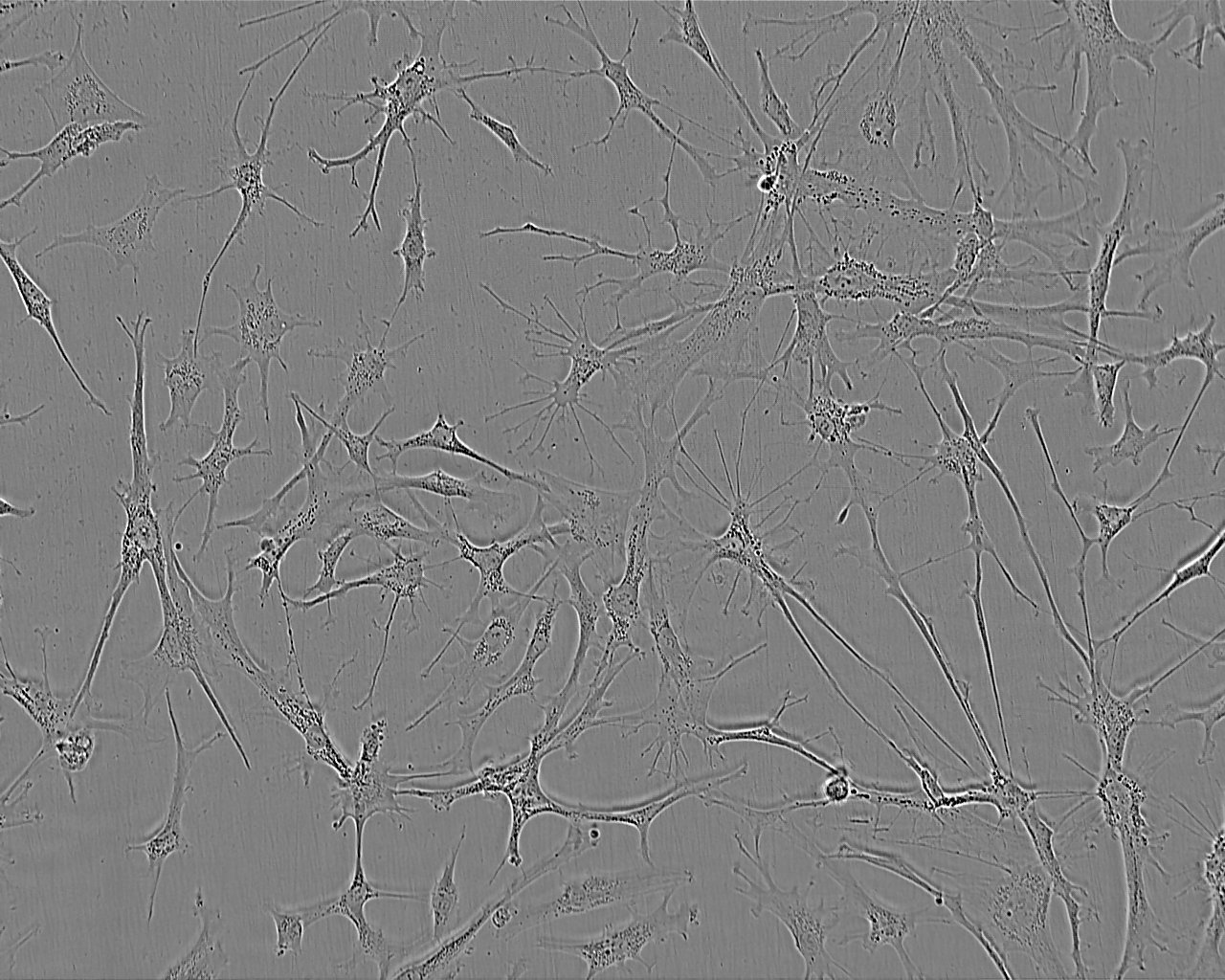

Supplement: Supplementary file 2 — Video microscopy movie 2. 13 frames of the time-lapse microscopy movie (.tif format) corresponding to normal astrocytes when treated with 2μM of CPD-1 alone. (ZIP 20,242 kb) [file 12859_2018_2458_MOESM2_ESM.zip › 1_N18_1_2017y06m29d_08h00m.tif]

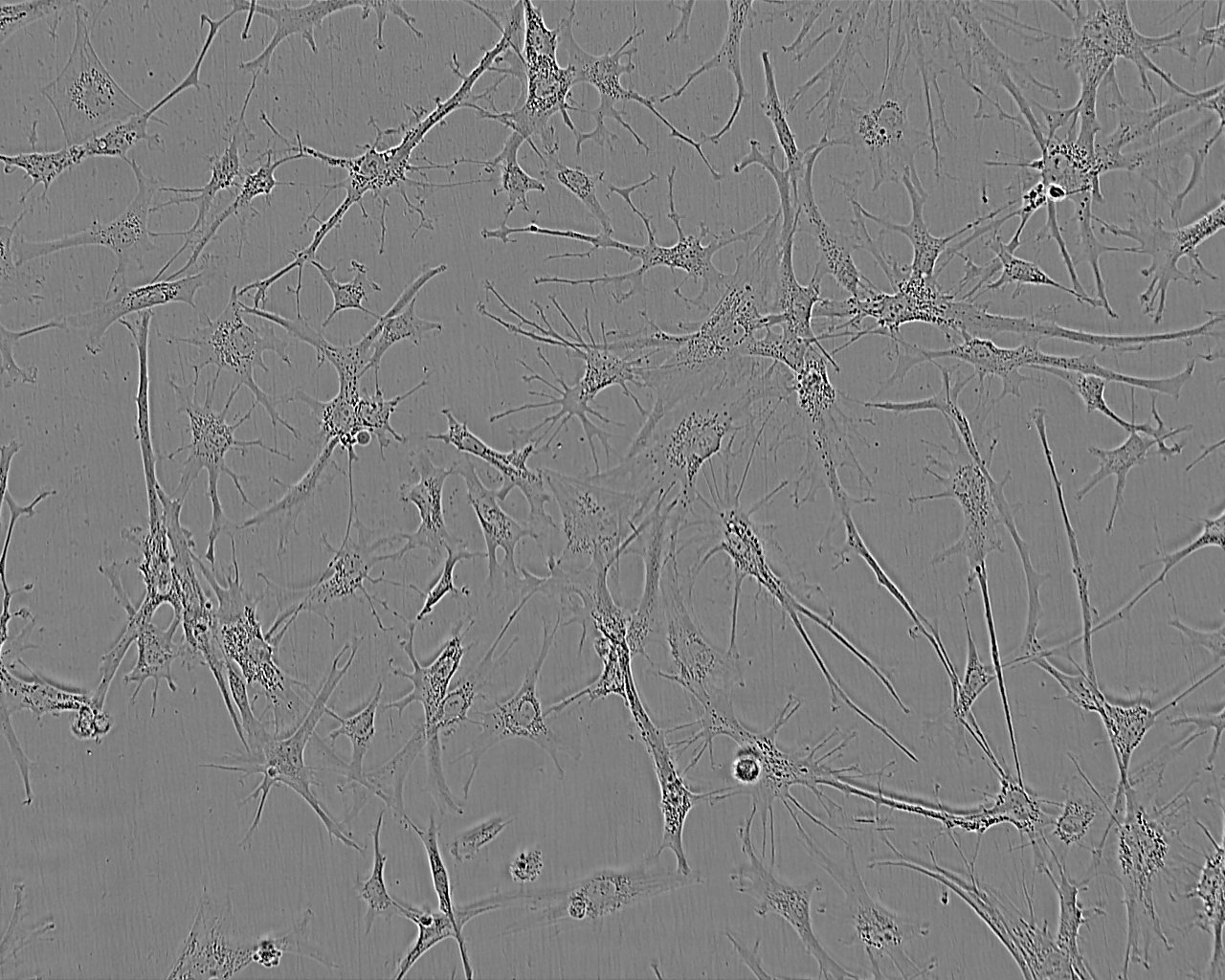

Supplement: Supplementary file 2 — Video microscopy movie 2. 13 frames of the time-lapse microscopy movie (.tif format) corresponding to normal astrocytes when treated with 2μM of CPD-1 alone. (ZIP 20,242 kb) [file 12859_2018_2458_MOESM2_ESM.zip › 1_N18_1_2017y06m29d_14h00m.tif]

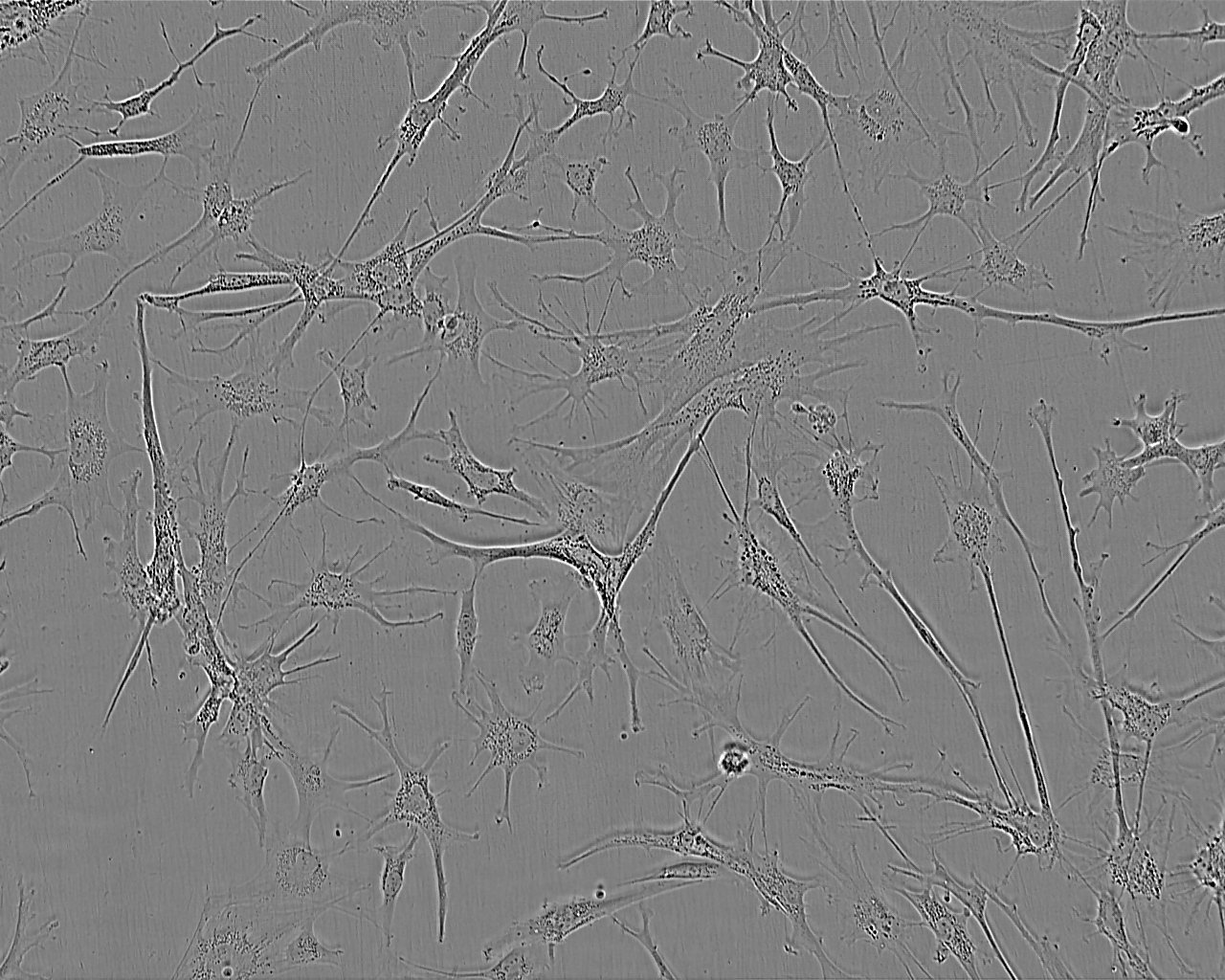

Supplement: Supplementary file 2 — Video microscopy movie 2. 13 frames of the time-lapse microscopy movie (.tif format) corresponding to normal astrocytes when treated with 2μM of CPD-1 alone. (ZIP 20,242 kb) [file 12859_2018_2458_MOESM2_ESM.zip › 1_N18_1_2017y06m29d_20h00m.tif]

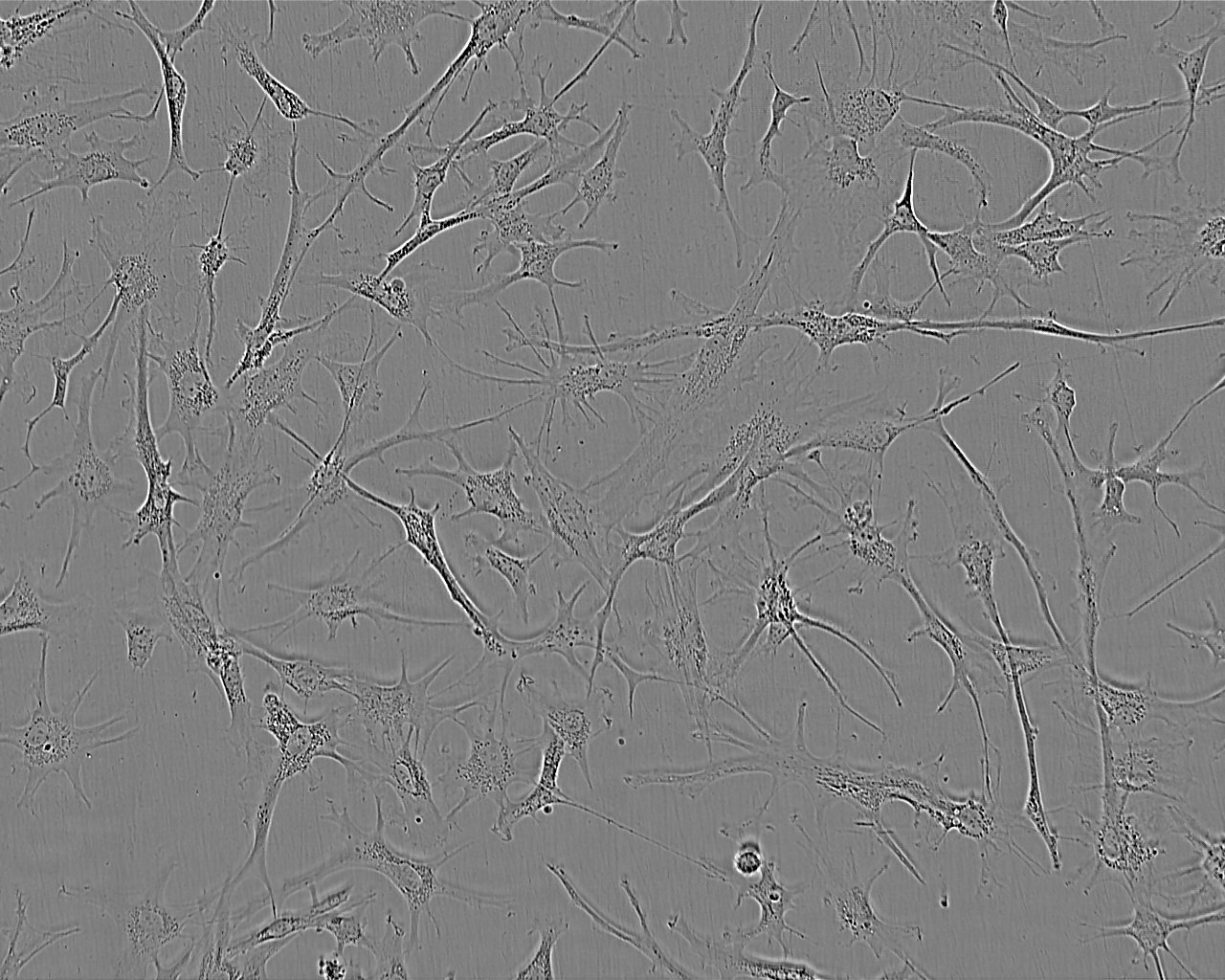

Supplement: Supplementary file 2 — Video microscopy movie 2. 13 frames of the time-lapse microscopy movie (.tif format) corresponding to normal astrocytes when treated with 2μM of CPD-1 alone. (ZIP 20,242 kb) [file 12859_2018_2458_MOESM2_ESM.zip › 1_N18_1_2017y06m30d_02h00m.tif]

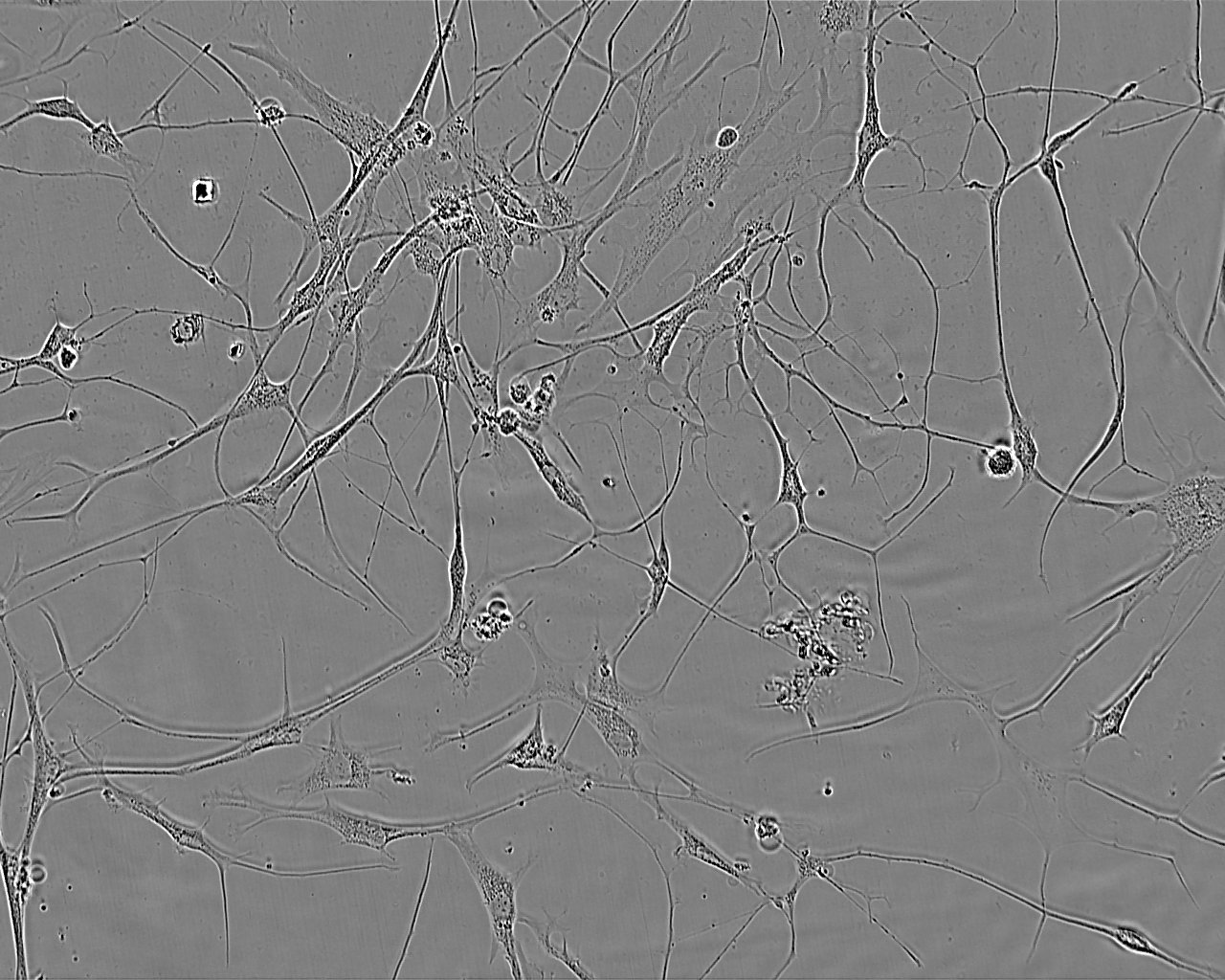

Supplement: Supplementary file 3 — Video microscopy movie 3. 13 frames of the time-lapse microscopy movie (.tif format) corresponding to normal astrocytes when treated with the combination concentration (CPD-1, SAHA) = (2μM, 7μM). (ZIP 18,617 kb) [file 12859_2018_2458_MOESM3_ESM.zip › 1_I18_1_2017y06m30d_07h00m.tif]

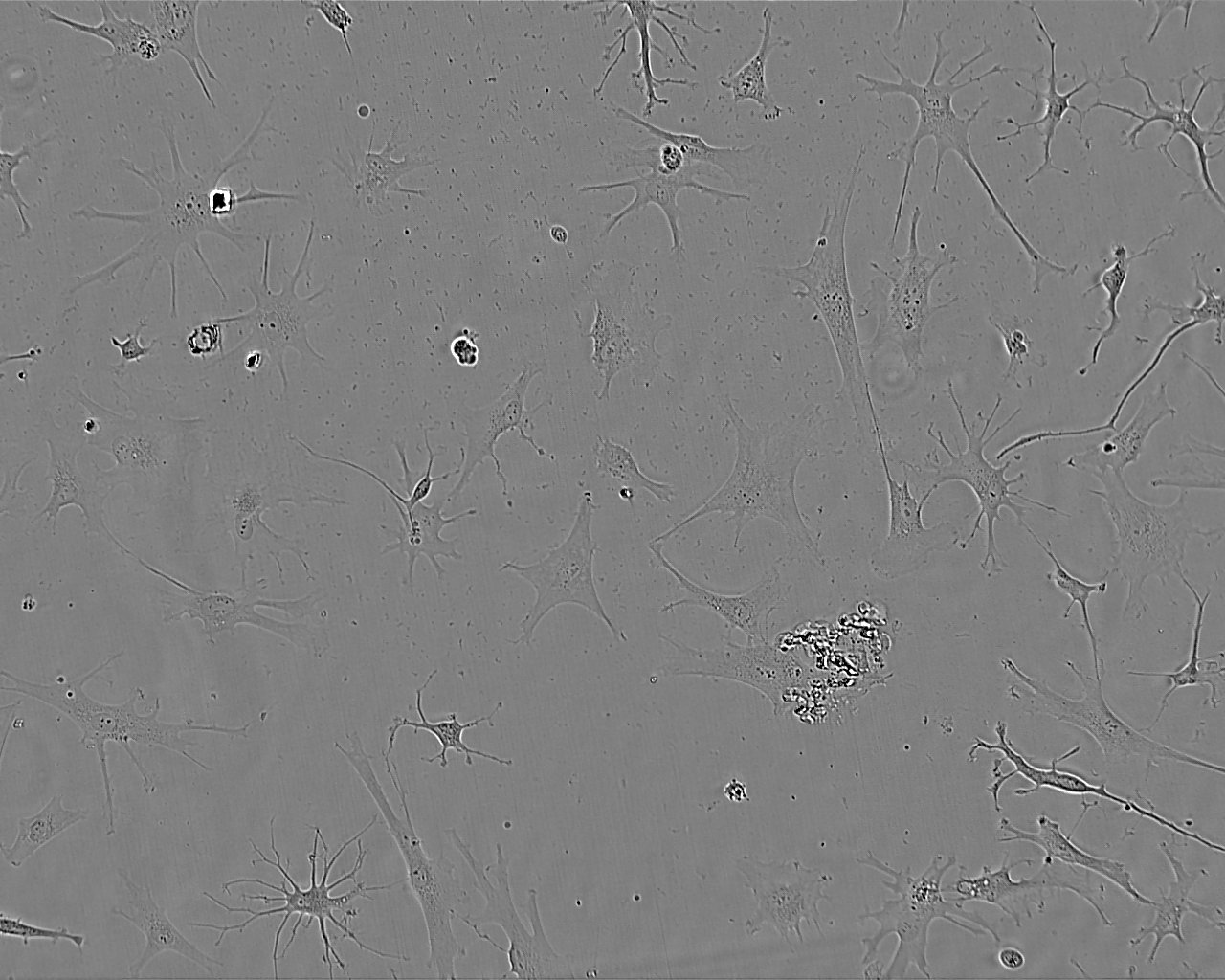

Supplement: Supplementary file 3 — Video microscopy movie 3. 13 frames of the time-lapse microscopy movie (.tif format) corresponding to normal astrocytes when treated with the combination concentration (CPD-1, SAHA) = (2μM, 7μM). (ZIP 18,617 kb) [file 12859_2018_2458_MOESM3_ESM.zip › 1_I18_1_2017y06m27d_08h00m.tif]

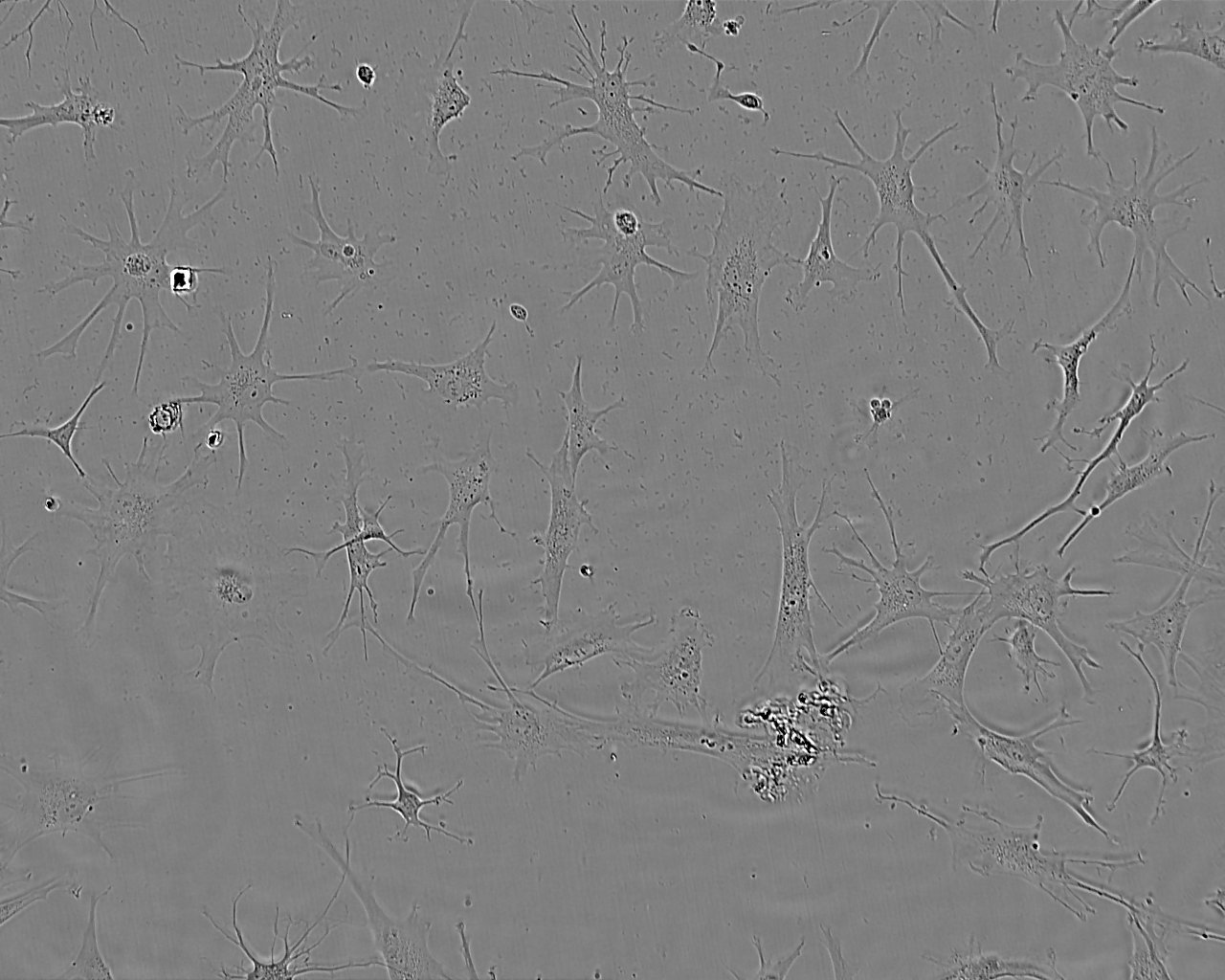

Supplement: Supplementary file 3 — Video microscopy movie 3. 13 frames of the time-lapse microscopy movie (.tif format) corresponding to normal astrocytes when treated with the combination concentration (CPD-1, SAHA) = (2μM, 7μM). (ZIP 18,617 kb) [file 12859_2018_2458_MOESM3_ESM.zip › 1_I18_1_2017y06m27d_14h00m.tif]

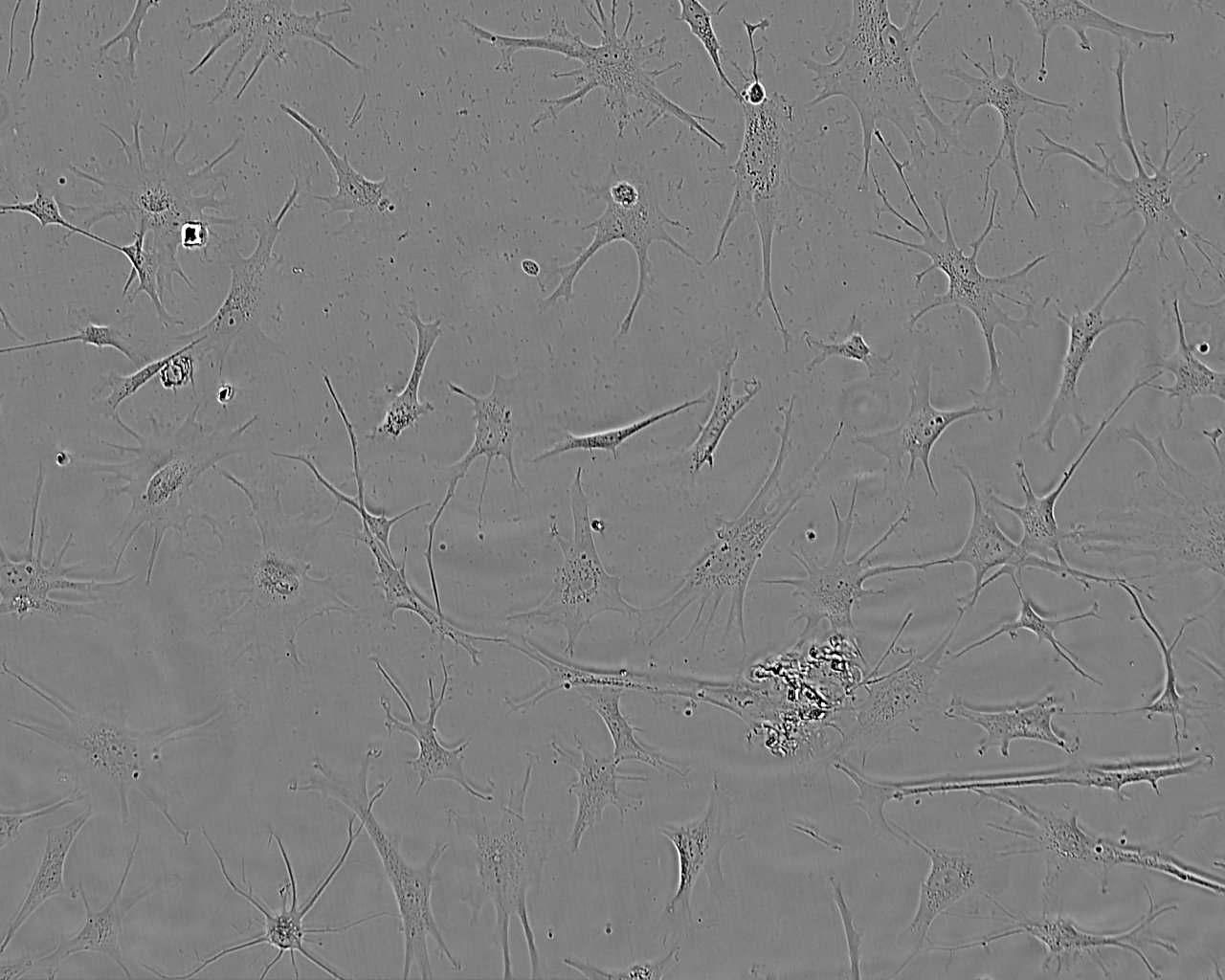

Supplement: Supplementary file 3 — Video microscopy movie 3. 13 frames of the time-lapse microscopy movie (.tif format) corresponding to normal astrocytes when treated with the combination concentration (CPD-1, SAHA) = (2μM, 7μM). (ZIP 18,617 kb) [file 12859_2018_2458_MOESM3_ESM.zip › 1_I18_1_2017y06m27d_20h00m.tif]

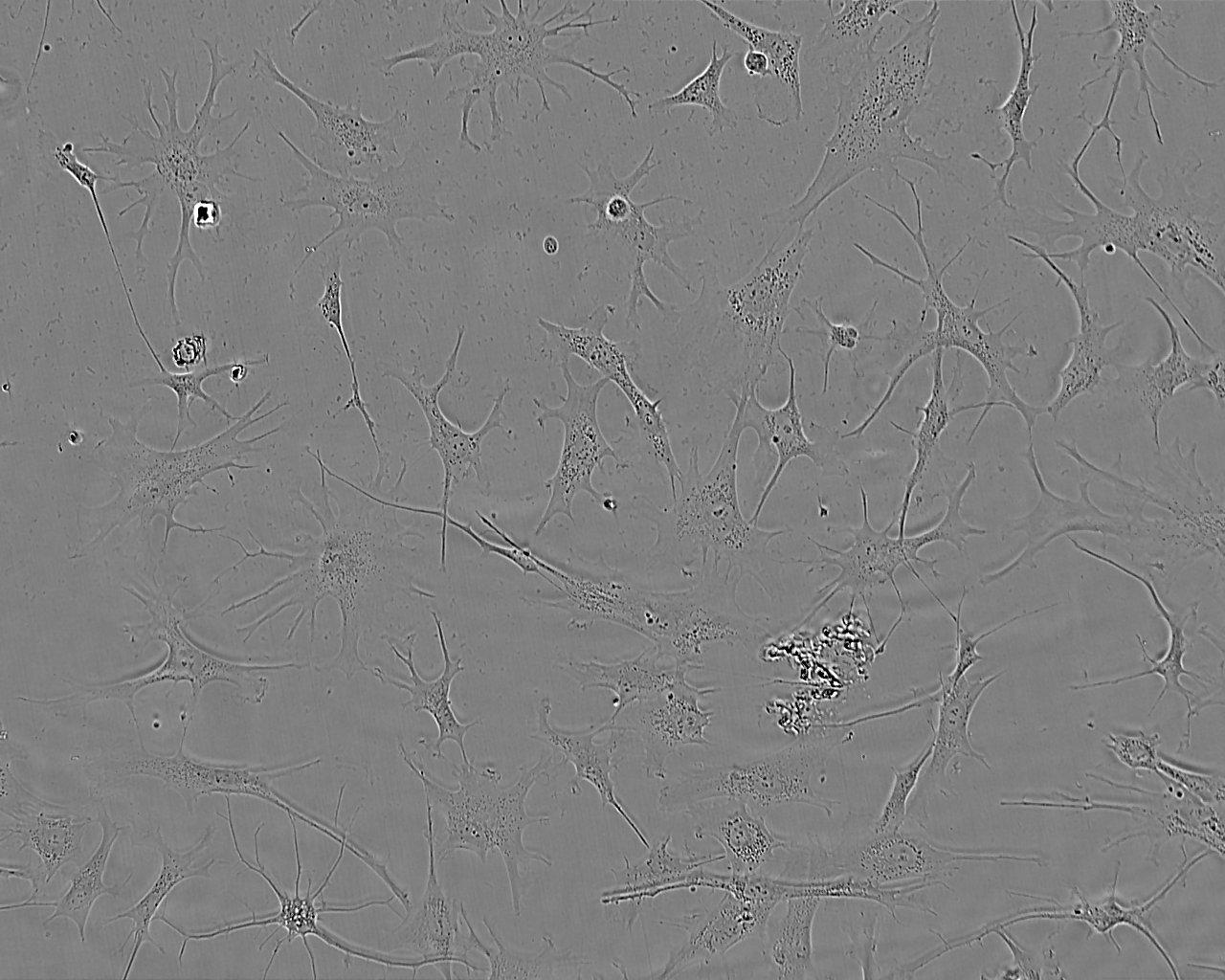

Supplement: Supplementary file 3 — Video microscopy movie 3. 13 frames of the time-lapse microscopy movie (.tif format) corresponding to normal astrocytes when treated with the combination concentration (CPD-1, SAHA) = (2μM, 7μM). (ZIP 18,617 kb) [file 12859_2018_2458_MOESM3_ESM.zip › 1_I18_1_2017y06m28d_02h00m.tif]

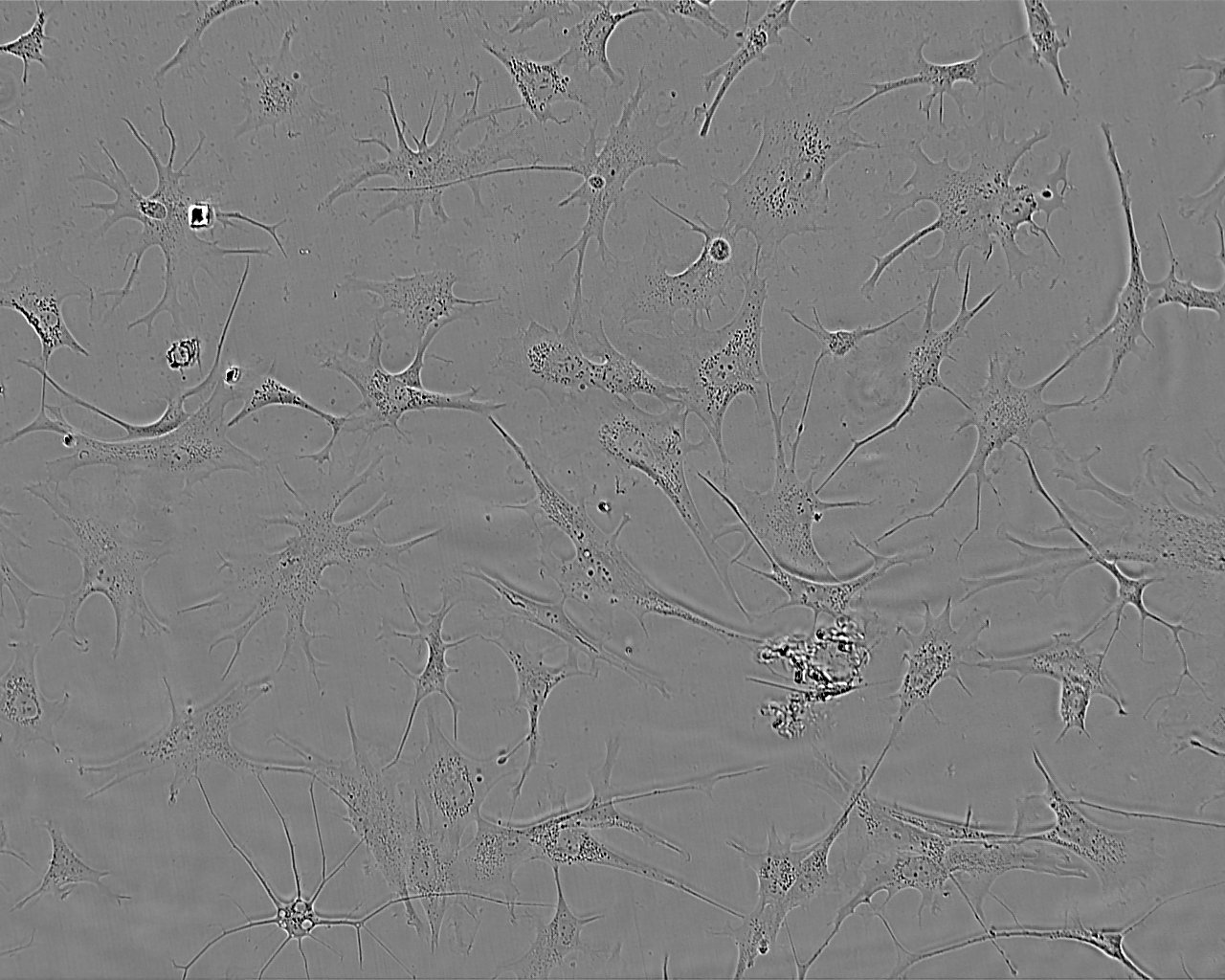

Supplement: Supplementary file 3 — Video microscopy movie 3. 13 frames of the time-lapse microscopy movie (.tif format) corresponding to normal astrocytes when treated with the combination concentration (CPD-1, SAHA) = (2μM, 7μM). (ZIP 18,617 kb) [file 12859_2018_2458_MOESM3_ESM.zip › 1_I18_1_2017y06m28d_08h00m.tif]

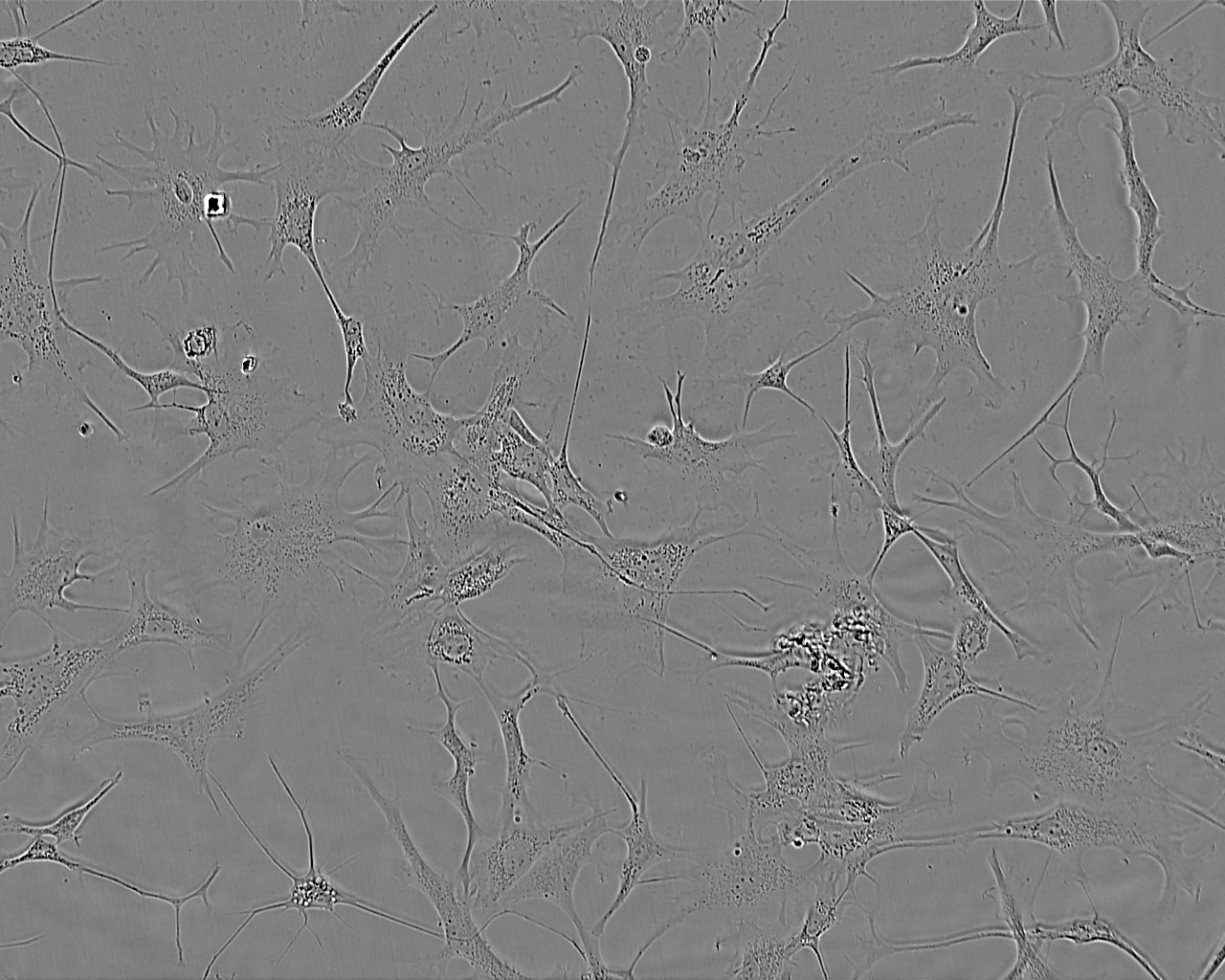

Supplement: Supplementary file 3 — Video microscopy movie 3. 13 frames of the time-lapse microscopy movie (.tif format) corresponding to normal astrocytes when treated with the combination concentration (CPD-1, SAHA) = (2μM, 7μM). (ZIP 18,617 kb) [file 12859_2018_2458_MOESM3_ESM.zip › 1_I18_1_2017y06m28d_14h00m.tif]

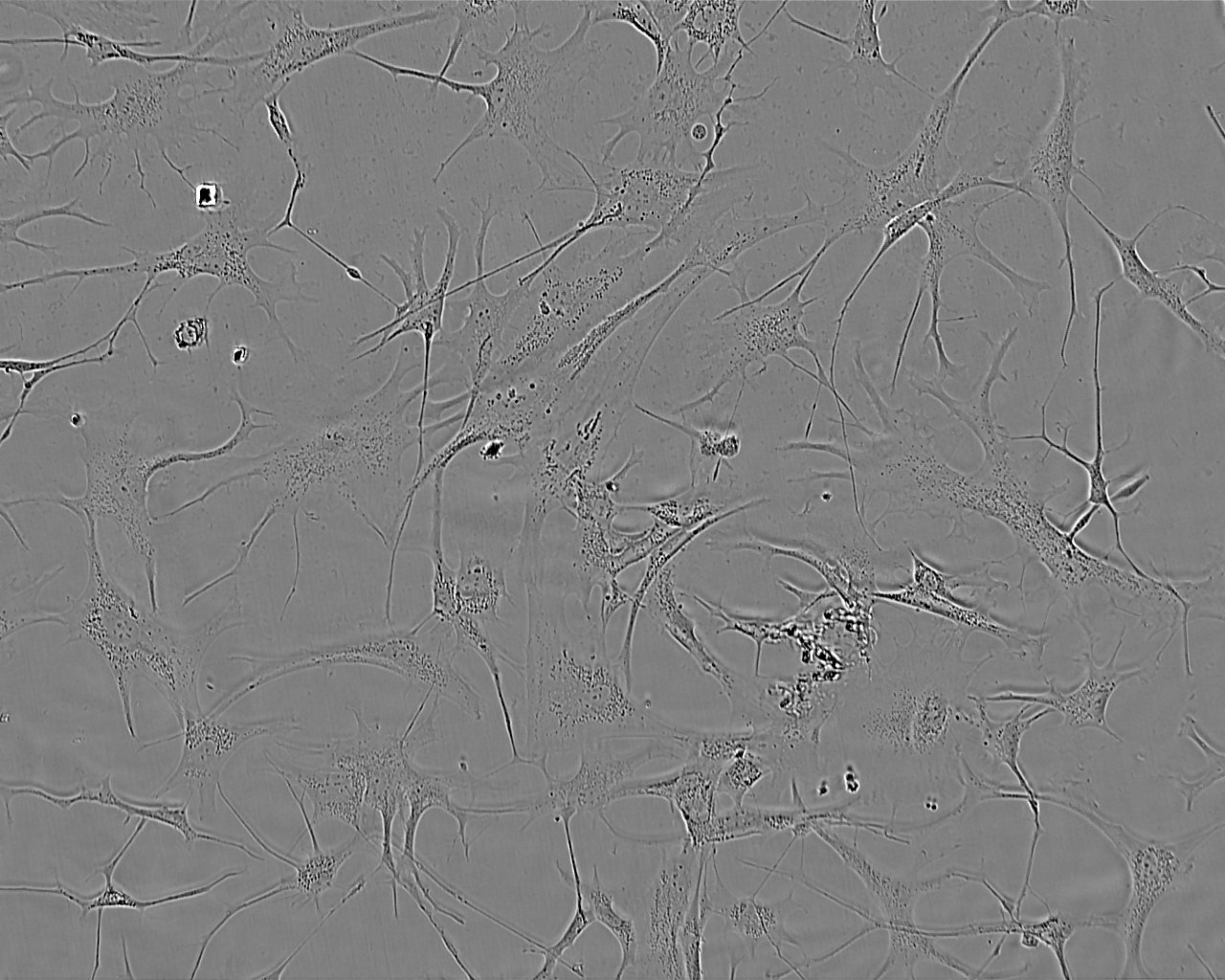

Supplement: Supplementary file 3 — Video microscopy movie 3. 13 frames of the time-lapse microscopy movie (.tif format) corresponding to normal astrocytes when treated with the combination concentration (CPD-1, SAHA) = (2μM, 7μM). (ZIP 18,617 kb) [file 12859_2018_2458_MOESM3_ESM.zip › 1_I18_1_2017y06m28d_20h00m.tif]

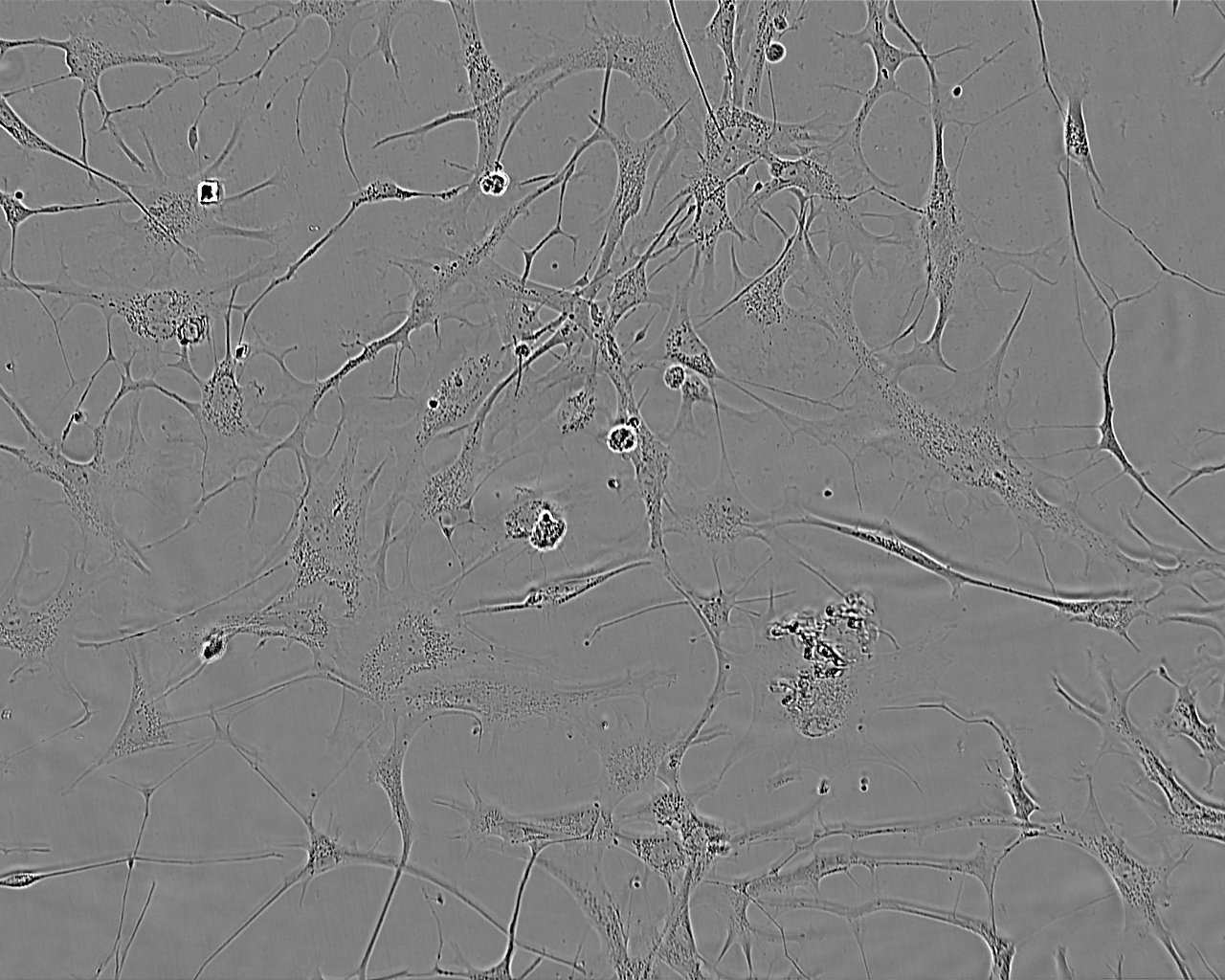

Supplement: Supplementary file 3 — Video microscopy movie 3. 13 frames of the time-lapse microscopy movie (.tif format) corresponding to normal astrocytes when treated with the combination concentration (CPD-1, SAHA) = (2μM, 7μM). (ZIP 18,617 kb) [file 12859_2018_2458_MOESM3_ESM.zip › 1_I18_1_2017y06m29d_02h00m.tif]

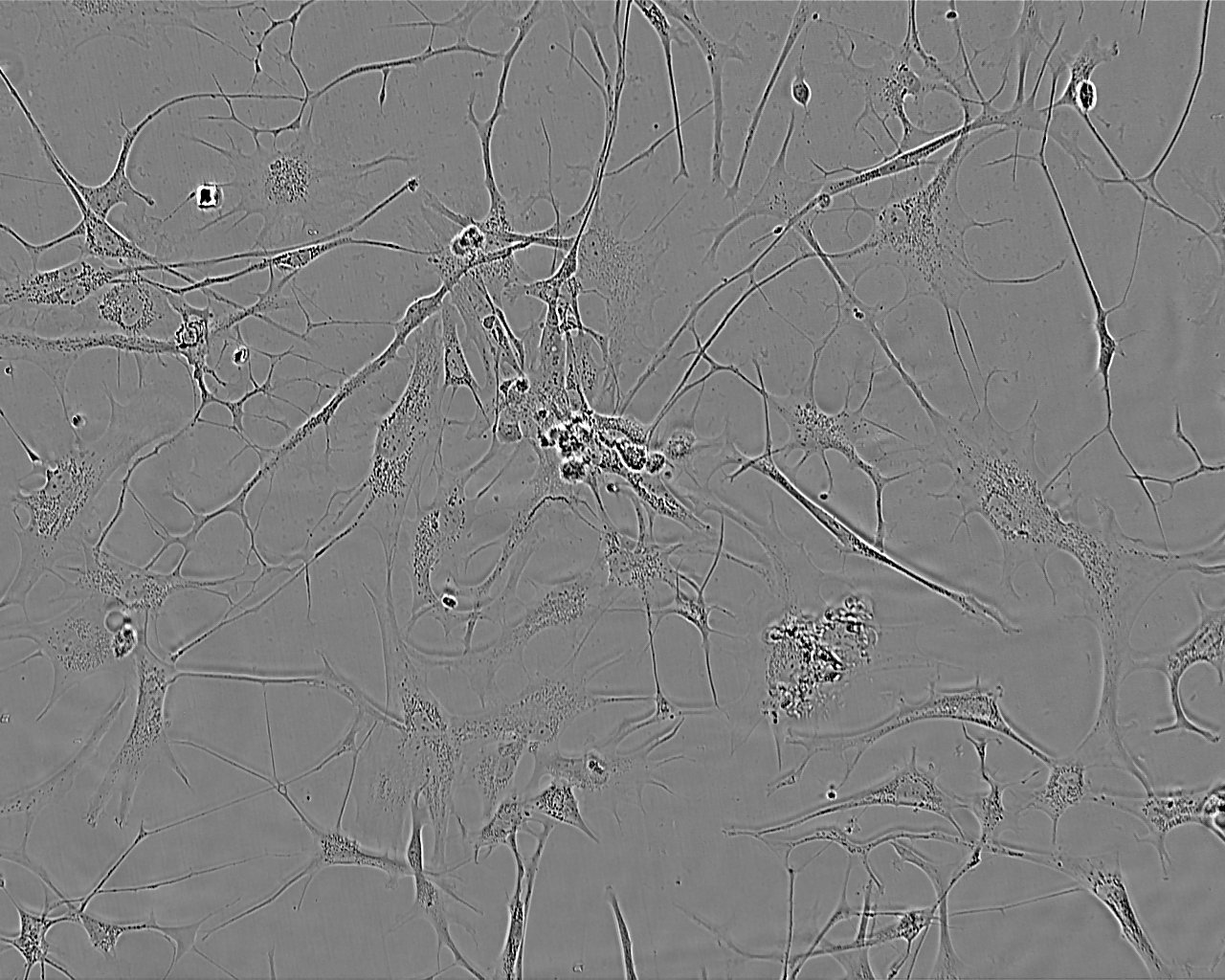

Supplement: Supplementary file 3 — Video microscopy movie 3. 13 frames of the time-lapse microscopy movie (.tif format) corresponding to normal astrocytes when treated with the combination concentration (CPD-1, SAHA) = (2μM, 7μM). (ZIP 18,617 kb) [file 12859_2018_2458_MOESM3_ESM.zip › 1_I18_1_2017y06m29d_08h00m.tif]

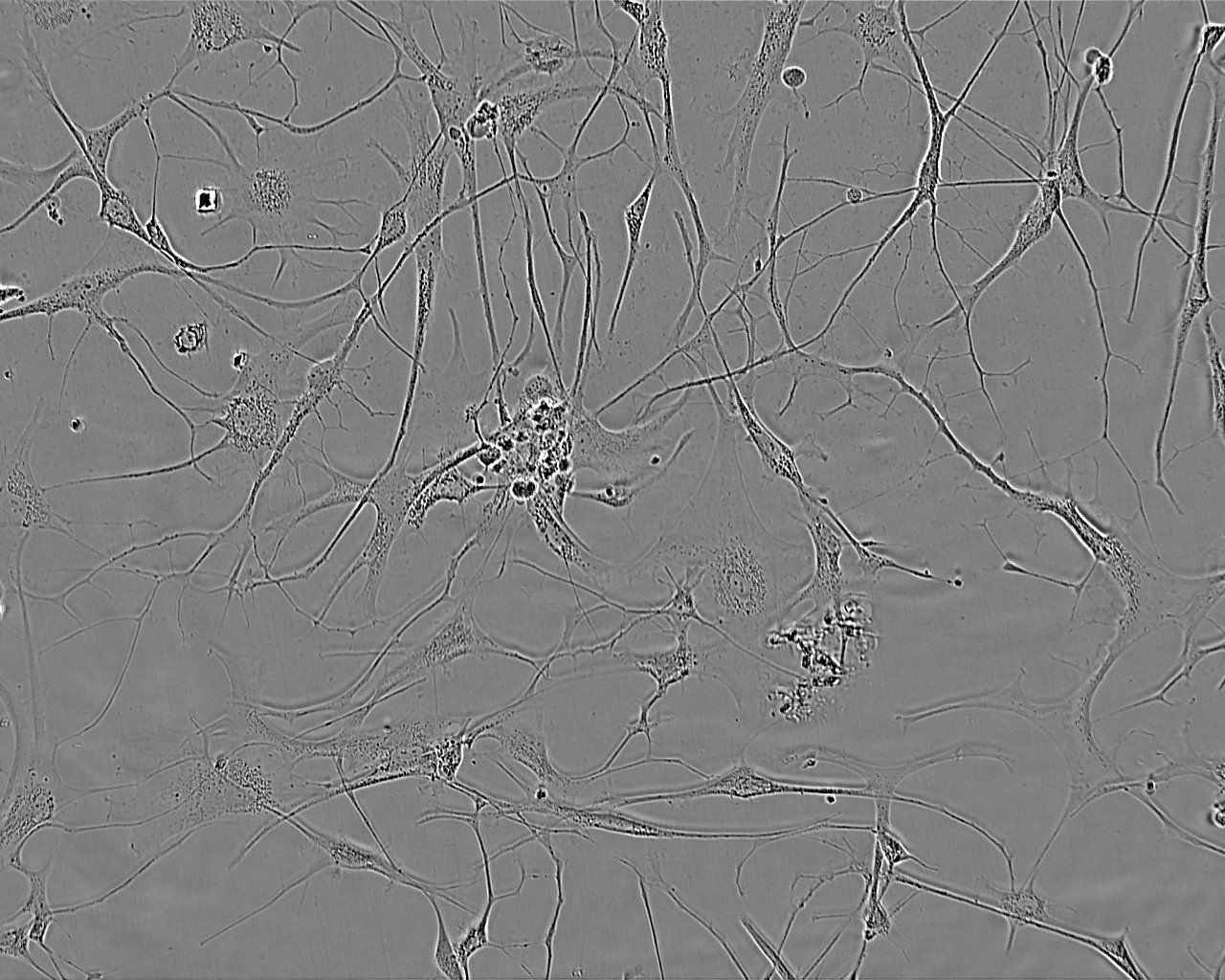

Supplement: Supplementary file 3 — Video microscopy movie 3. 13 frames of the time-lapse microscopy movie (.tif format) corresponding to normal astrocytes when treated with the combination concentration (CPD-1, SAHA) = (2μM, 7μM). (ZIP 18,617 kb) [file 12859_2018_2458_MOESM3_ESM.zip › 1_I18_1_2017y06m29d_14h00m.tif]

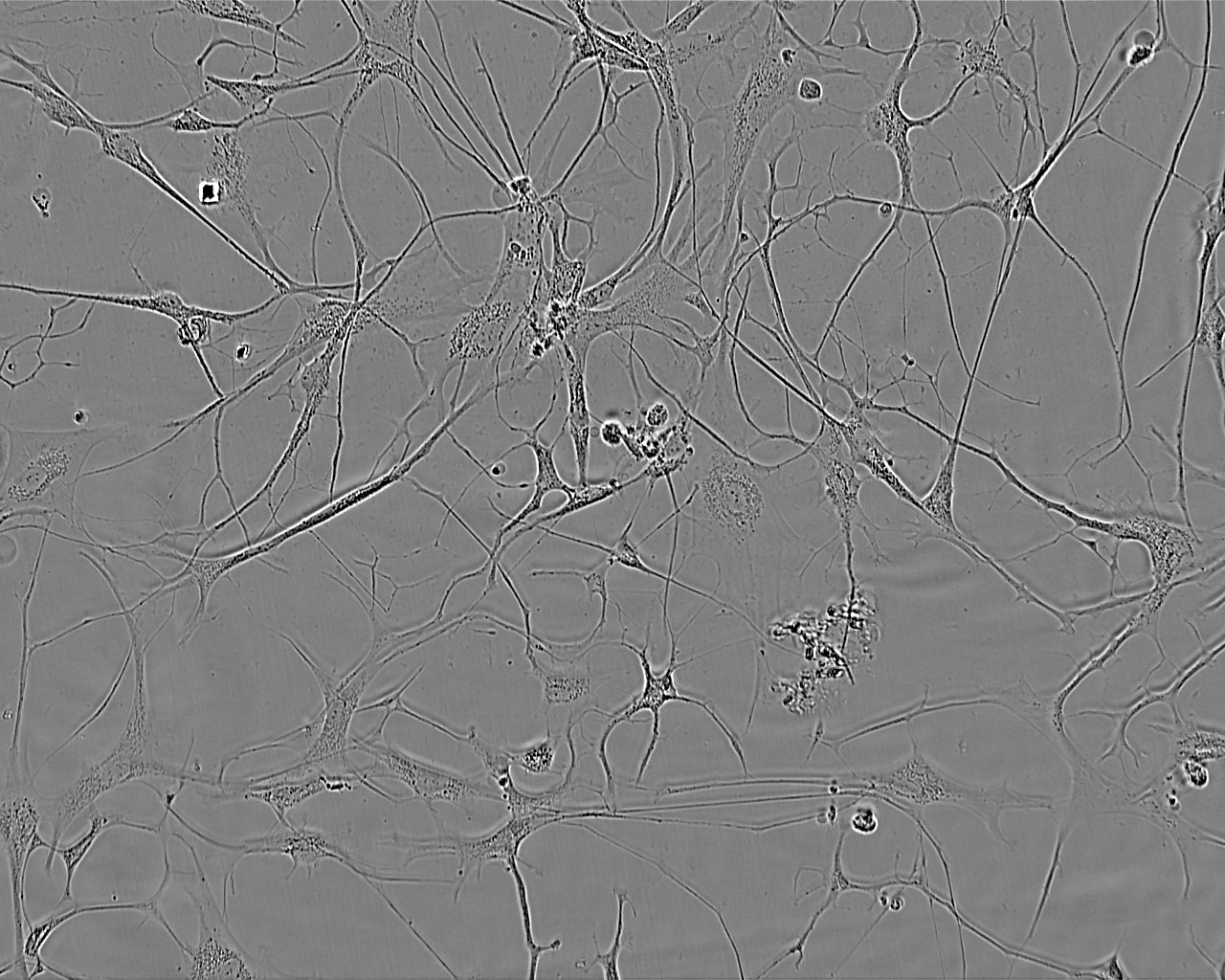

Supplement: Supplementary file 3 — Video microscopy movie 3. 13 frames of the time-lapse microscopy movie (.tif format) corresponding to normal astrocytes when treated with the combination concentration (CPD-1, SAHA) = (2μM, 7μM). (ZIP 18,617 kb) [file 12859_2018_2458_MOESM3_ESM.zip › 1_I18_1_2017y06m29d_20h00m.tif]

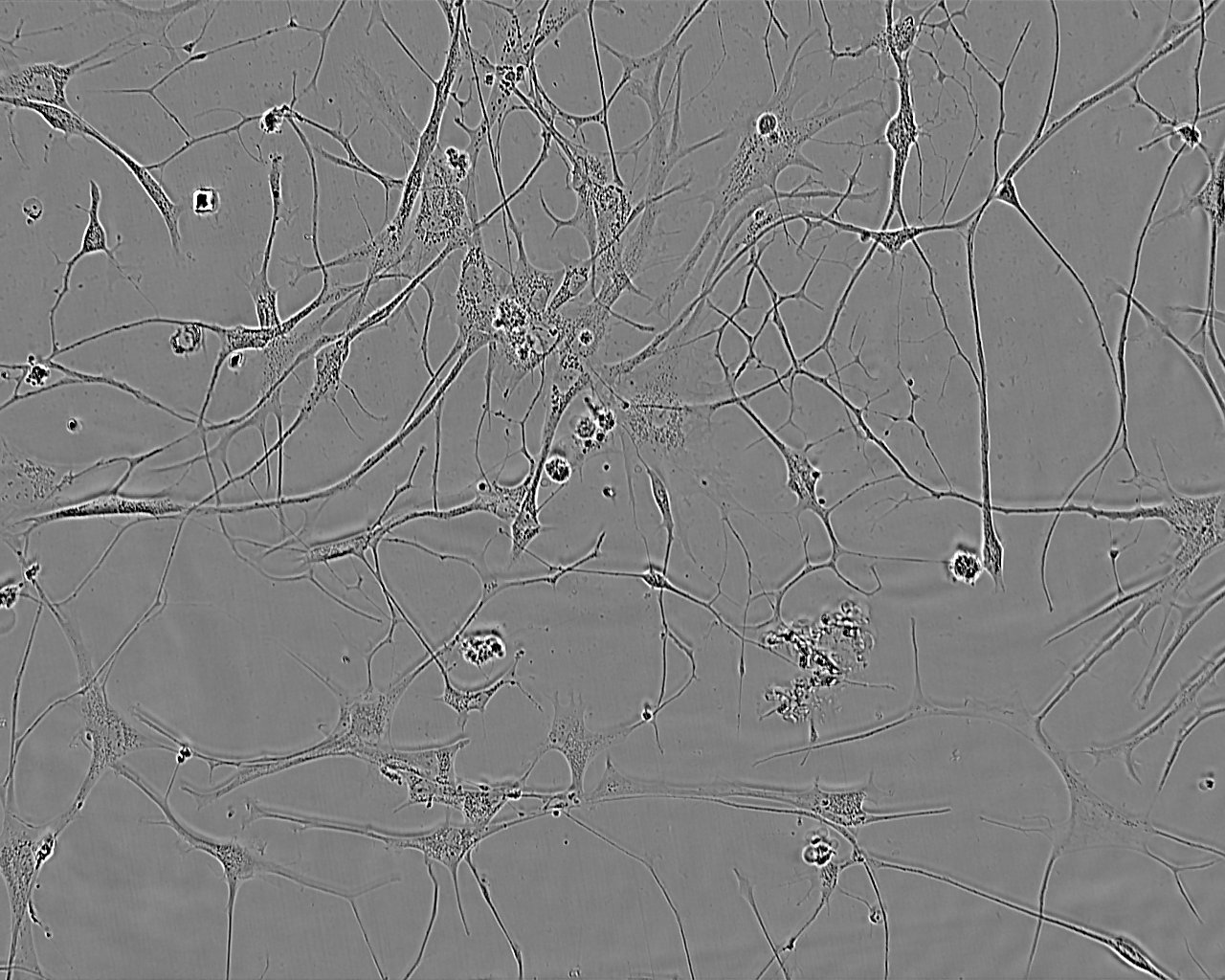

Supplement: Supplementary file 3 — Video microscopy movie 3. 13 frames of the time-lapse microscopy movie (.tif format) corresponding to normal astrocytes when treated with the combination concentration (CPD-1, SAHA) = (2μM, 7μM). (ZIP 18,617 kb) [file 12859_2018_2458_MOESM3_ESM.zip › 1_I18_1_2017y06m30d_02h00m.tif]

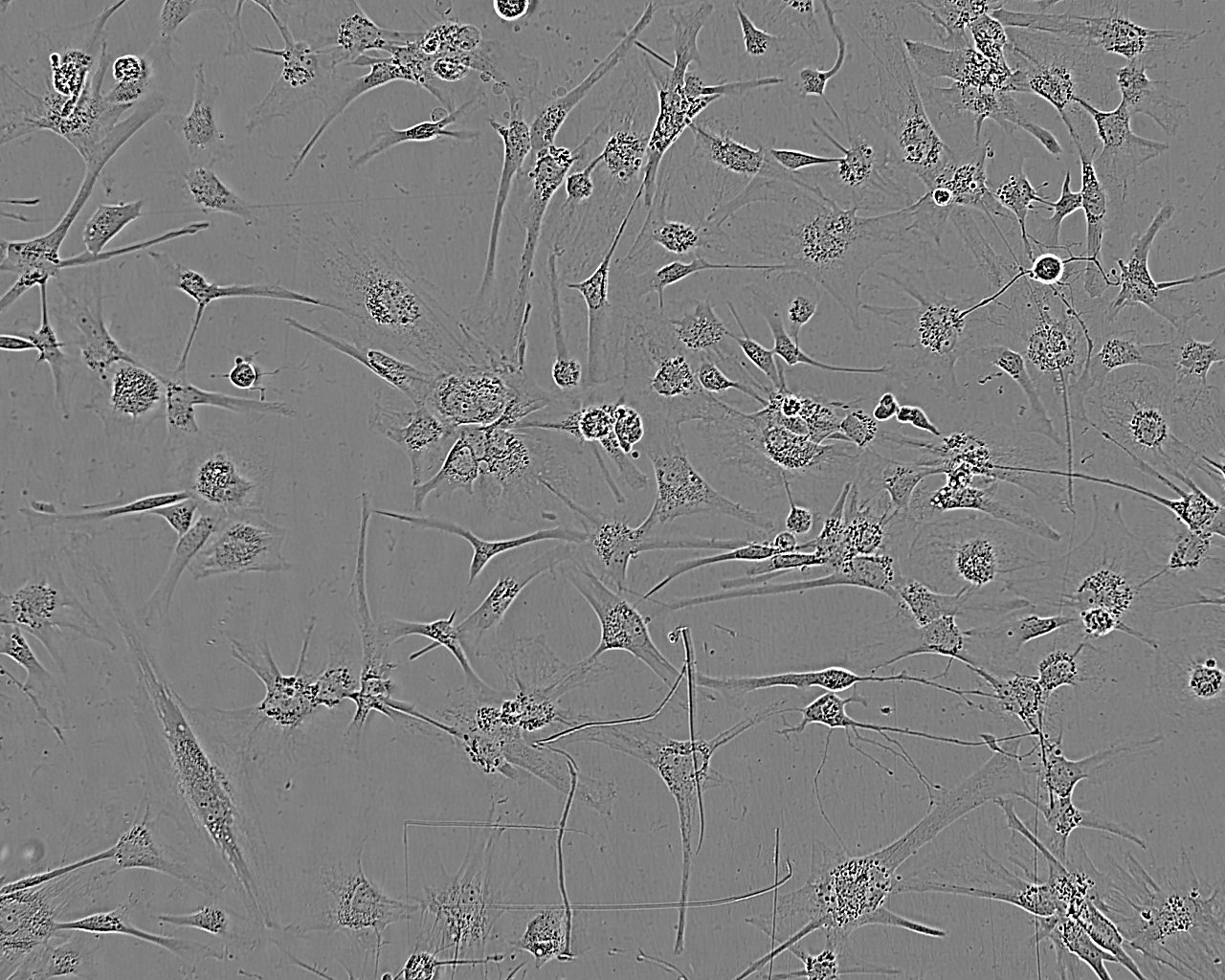

Supplement: Supplementary file 4 — Video microscopy movie 4. 13 frames of the time-lapse microscopy movie (.tif format) corresponding to the resistant GIC clone U3065−c475 when treated with 2μM of CPD-2 alone. (ZIP 20,155 kb) [file 12859_2018_2458_MOESM4_ESM.zip › 1_H23_1_2017y06m30d_06h12m.tif]

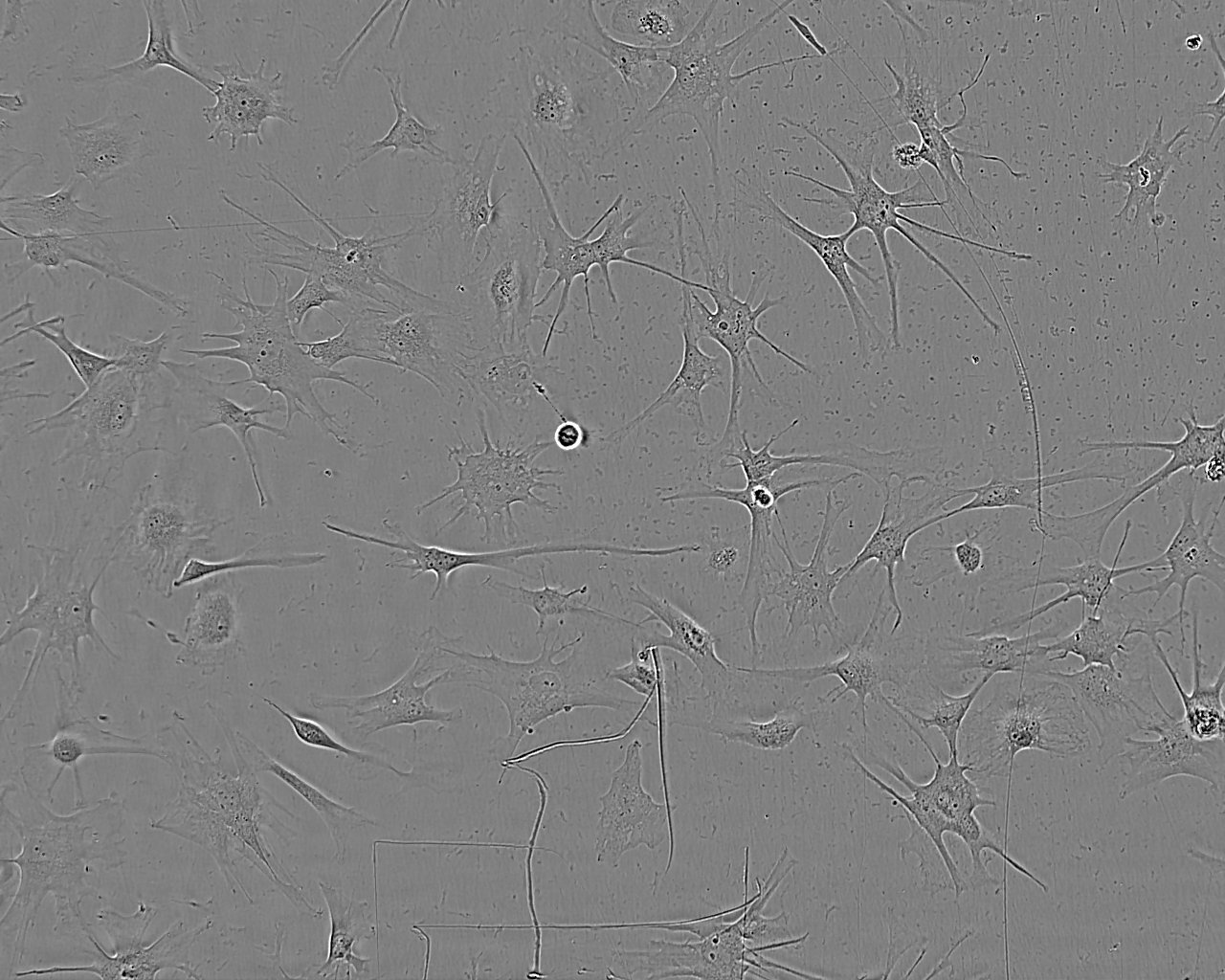

Supplement: Supplementary file 4 — Video microscopy movie 4. 13 frames of the time-lapse microscopy movie (.tif format) corresponding to the resistant GIC clone U3065−c475 when treated with 2μM of CPD-2 alone. (ZIP 20,155 kb) [file 12859_2018_2458_MOESM4_ESM.zip › 1_H23_1_2017y06m27d_07h12m.tif]

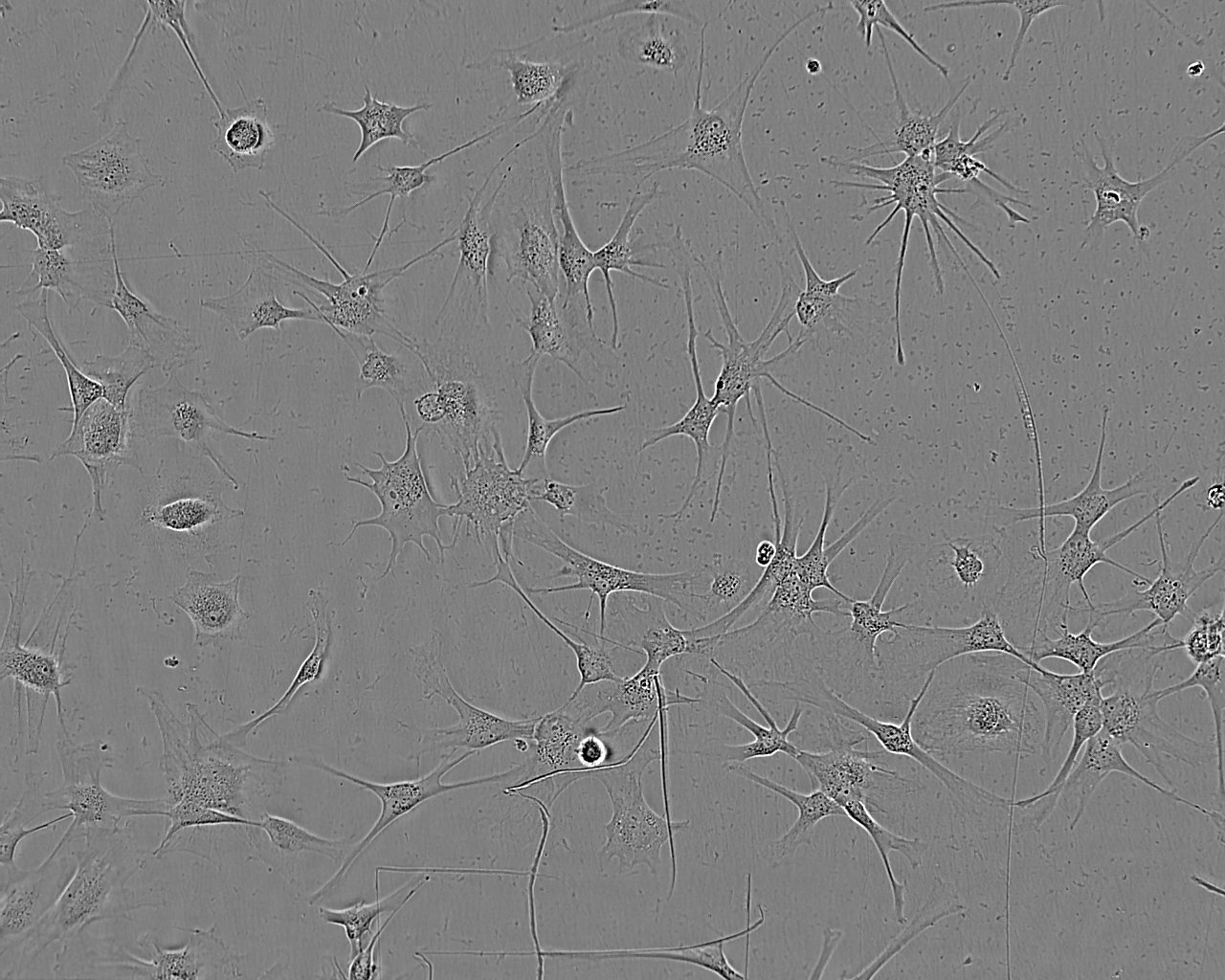

Supplement: Supplementary file 4 — Video microscopy movie 4. 13 frames of the time-lapse microscopy movie (.tif format) corresponding to the resistant GIC clone U3065−c475 when treated with 2μM of CPD-2 alone. (ZIP 20,155 kb) [file 12859_2018_2458_MOESM4_ESM.zip › 1_H23_1_2017y06m27d_13h12m.tif]

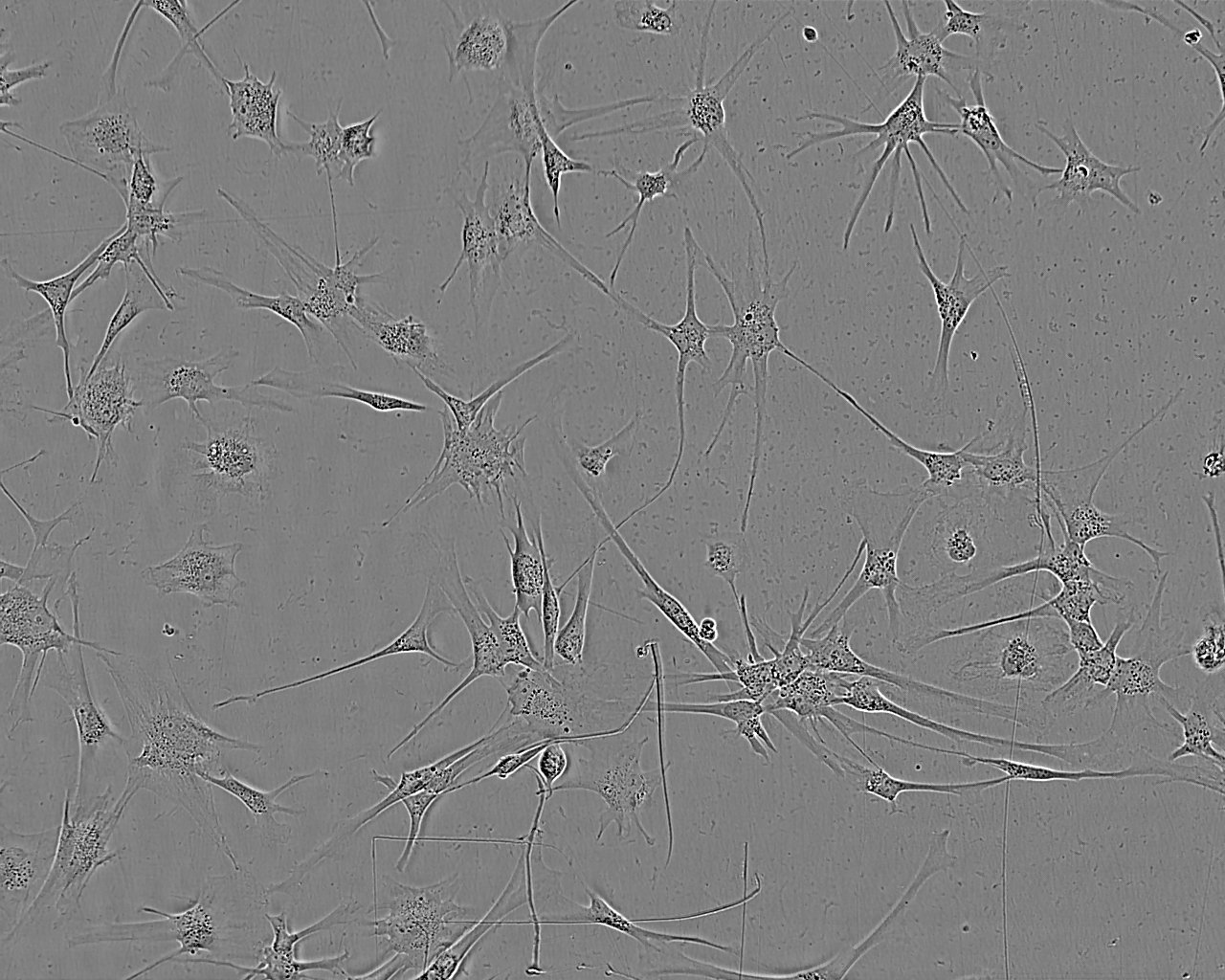

Supplement: Supplementary file 4 — Video microscopy movie 4. 13 frames of the time-lapse microscopy movie (.tif format) corresponding to the resistant GIC clone U3065−c475 when treated with 2μM of CPD-2 alone. (ZIP 20,155 kb) [file 12859_2018_2458_MOESM4_ESM.zip › 1_H23_1_2017y06m27d_19h12m.tif]

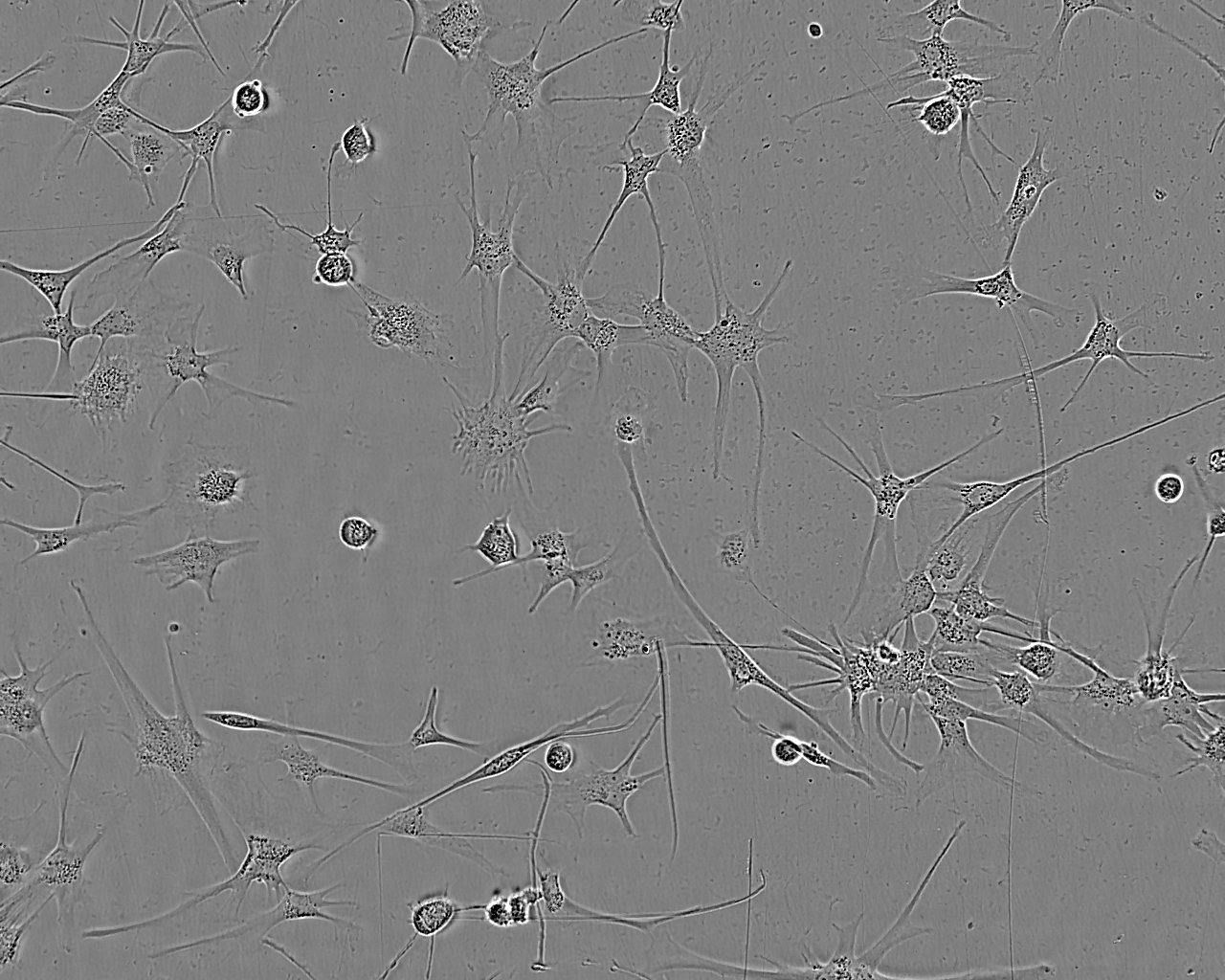

Supplement: Supplementary file 4 — Video microscopy movie 4. 13 frames of the time-lapse microscopy movie (.tif format) corresponding to the resistant GIC clone U3065−c475 when treated with 2μM of CPD-2 alone. (ZIP 20,155 kb) [file 12859_2018_2458_MOESM4_ESM.zip › 1_H23_1_2017y06m28d_01h12m.tif]

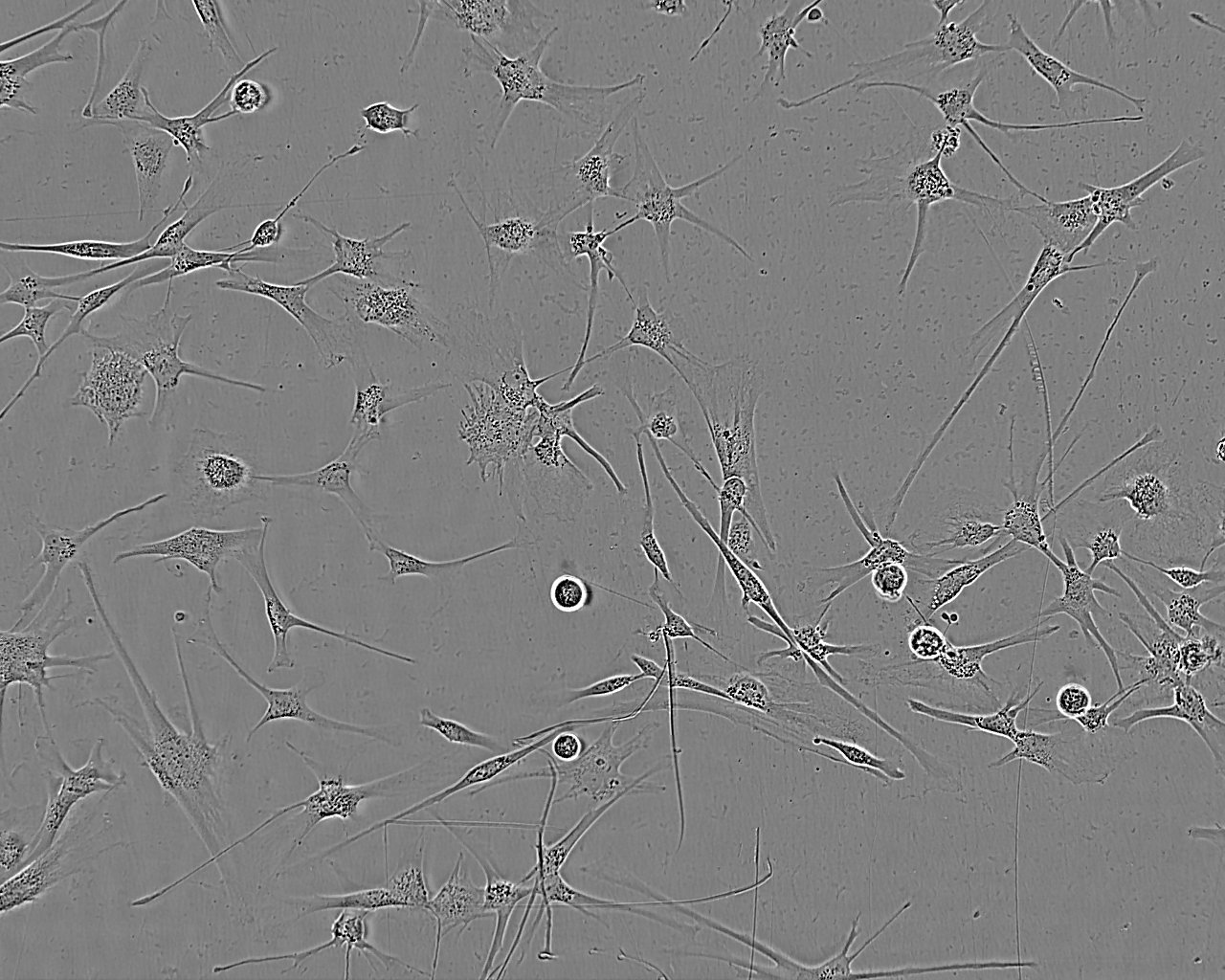

Supplement: Supplementary file 4 — Video microscopy movie 4. 13 frames of the time-lapse microscopy movie (.tif format) corresponding to the resistant GIC clone U3065−c475 when treated with 2μM of CPD-2 alone. (ZIP 20,155 kb) [file 12859_2018_2458_MOESM4_ESM.zip › 1_H23_1_2017y06m28d_07h12m.tif]

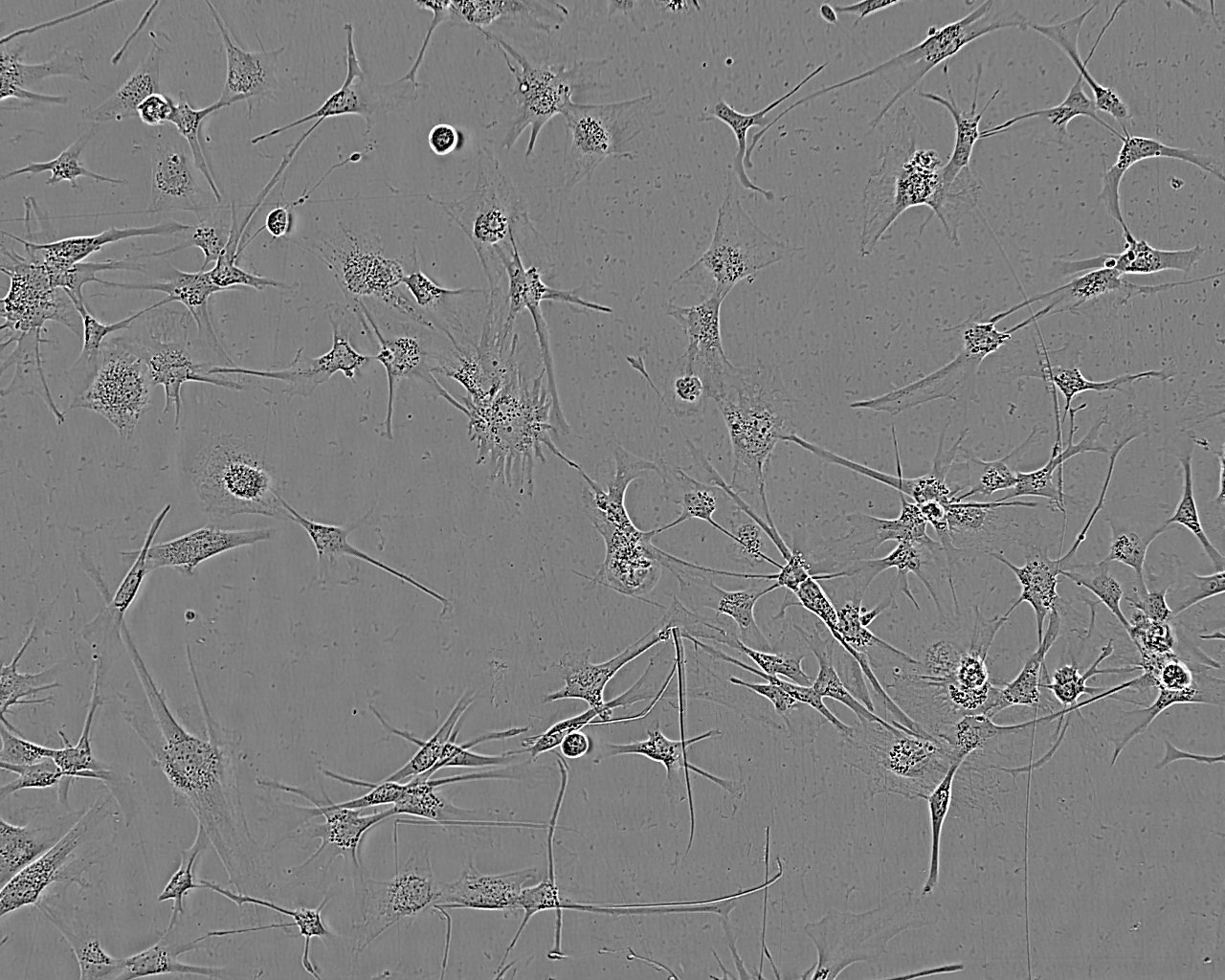

Supplement: Supplementary file 4 — Video microscopy movie 4. 13 frames of the time-lapse microscopy movie (.tif format) corresponding to the resistant GIC clone U3065−c475 when treated with 2μM of CPD-2 alone. (ZIP 20,155 kb) [file 12859_2018_2458_MOESM4_ESM.zip › 1_H23_1_2017y06m28d_13h12m.tif]

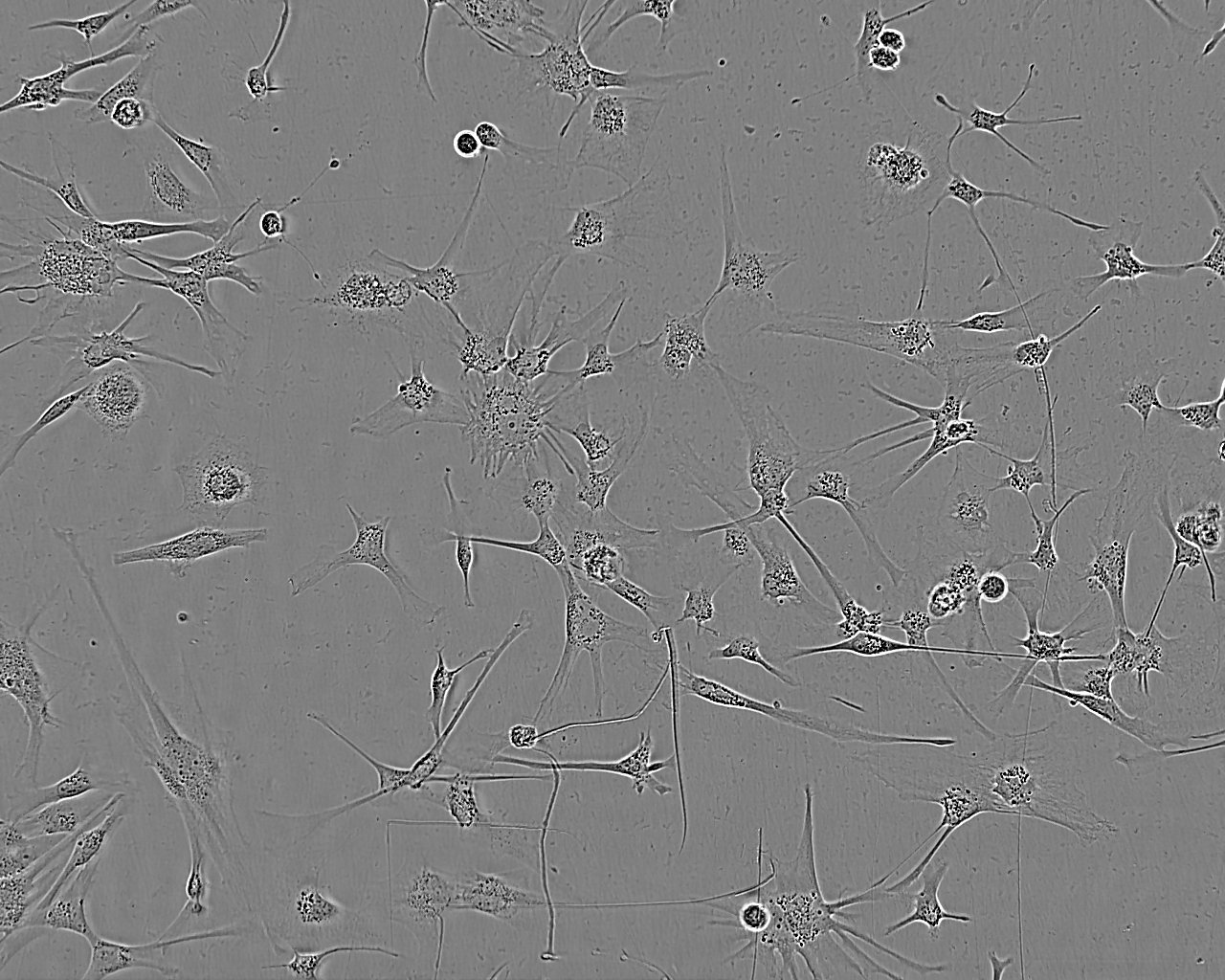

Supplement: Supplementary file 4 — Video microscopy movie 4. 13 frames of the time-lapse microscopy movie (.tif format) corresponding to the resistant GIC clone U3065−c475 when treated with 2μM of CPD-2 alone. (ZIP 20,155 kb) [file 12859_2018_2458_MOESM4_ESM.zip › 1_H23_1_2017y06m28d_19h12m.tif]

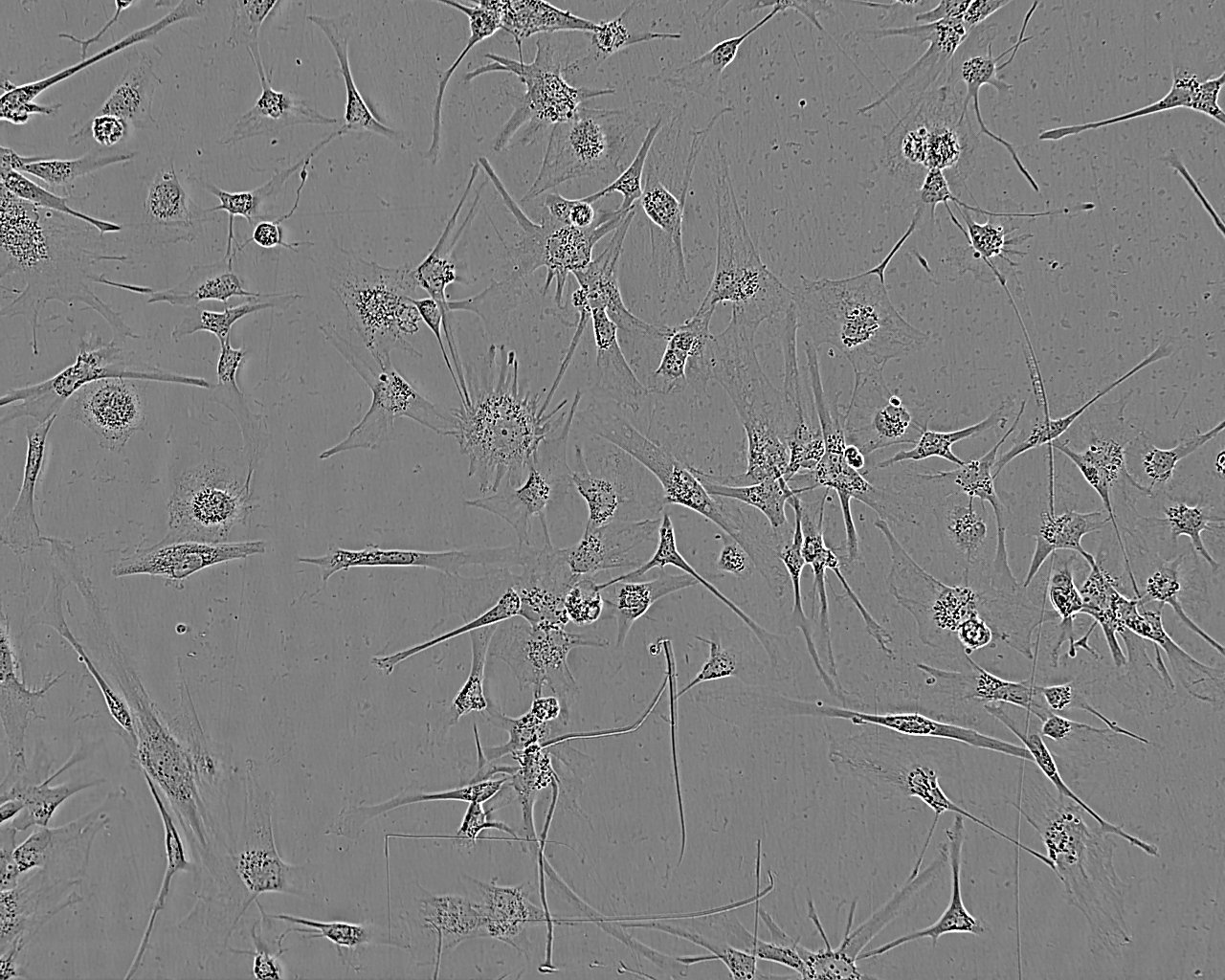

Supplement: Supplementary file 4 — Video microscopy movie 4. 13 frames of the time-lapse microscopy movie (.tif format) corresponding to the resistant GIC clone U3065−c475 when treated with 2μM of CPD-2 alone. (ZIP 20,155 kb) [file 12859_2018_2458_MOESM4_ESM.zip › 1_H23_1_2017y06m29d_01h12m.tif]

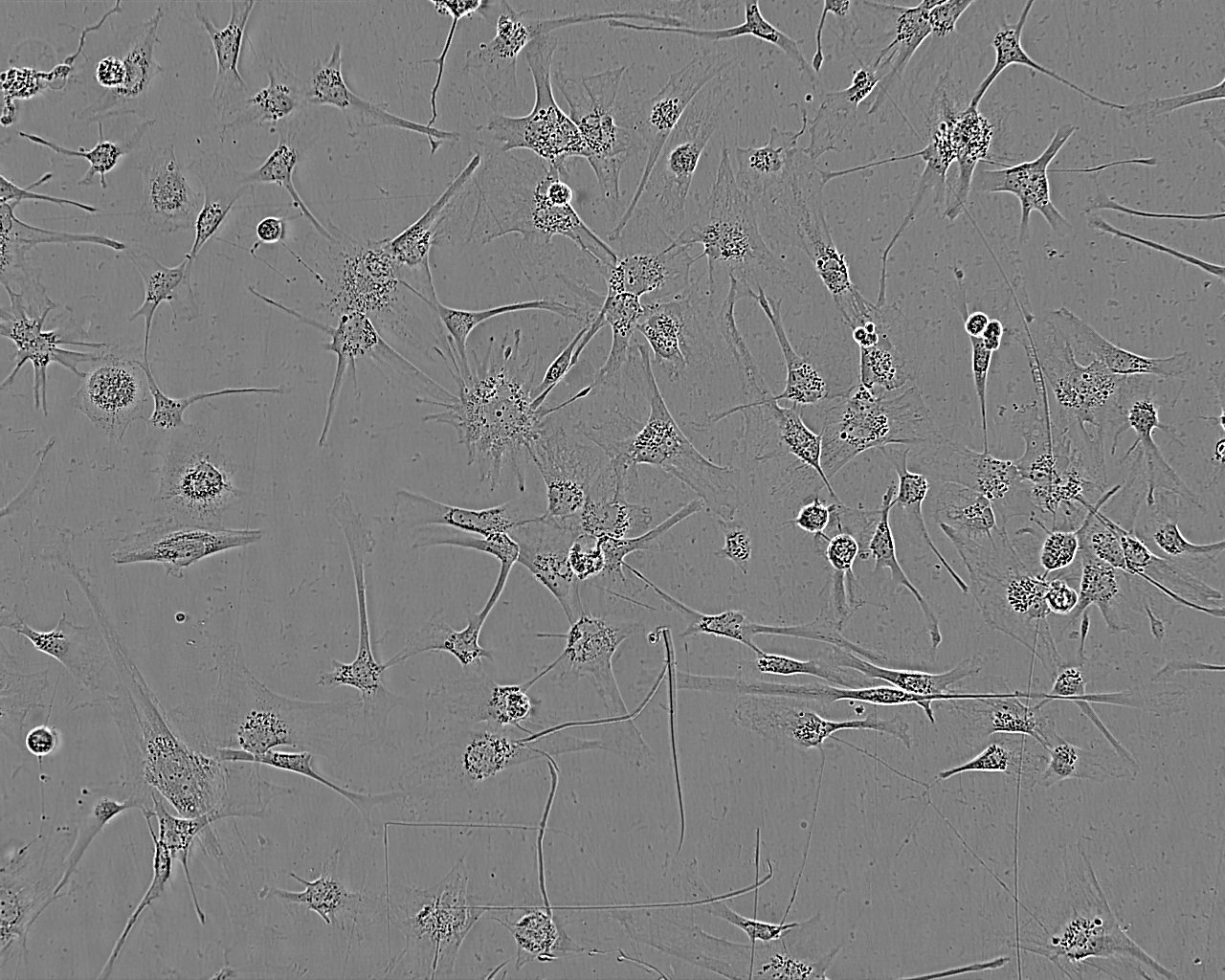

Supplement: Supplementary file 4 — Video microscopy movie 4. 13 frames of the time-lapse microscopy movie (.tif format) corresponding to the resistant GIC clone U3065−c475 when treated with 2μM of CPD-2 alone. (ZIP 20,155 kb) [file 12859_2018_2458_MOESM4_ESM.zip › 1_H23_1_2017y06m29d_07h12m.tif]

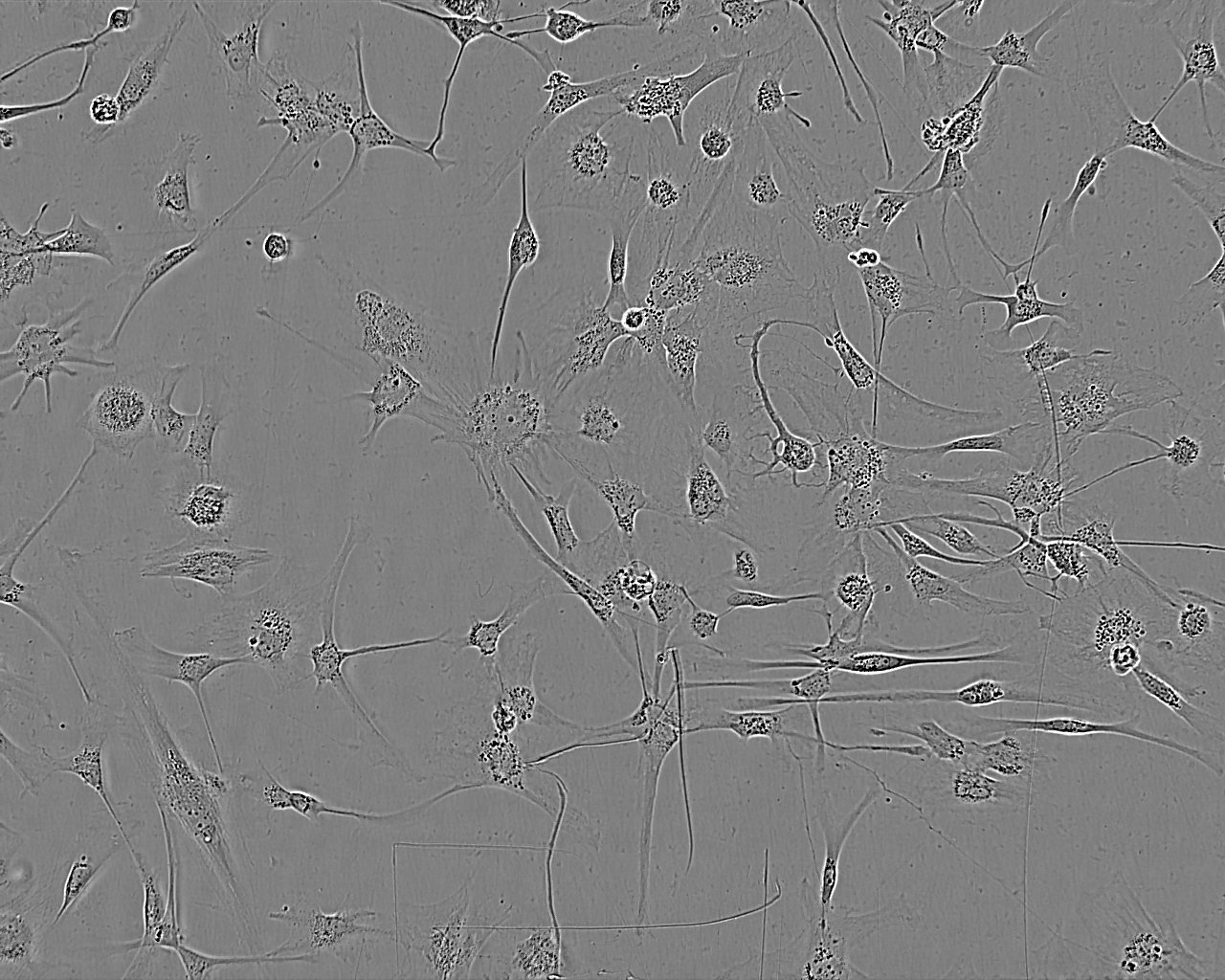

Supplement: Supplementary file 4 — Video microscopy movie 4. 13 frames of the time-lapse microscopy movie (.tif format) corresponding to the resistant GIC clone U3065−c475 when treated with 2μM of CPD-2 alone. (ZIP 20,155 kb) [file 12859_2018_2458_MOESM4_ESM.zip › 1_H23_1_2017y06m29d_13h12m.tif]

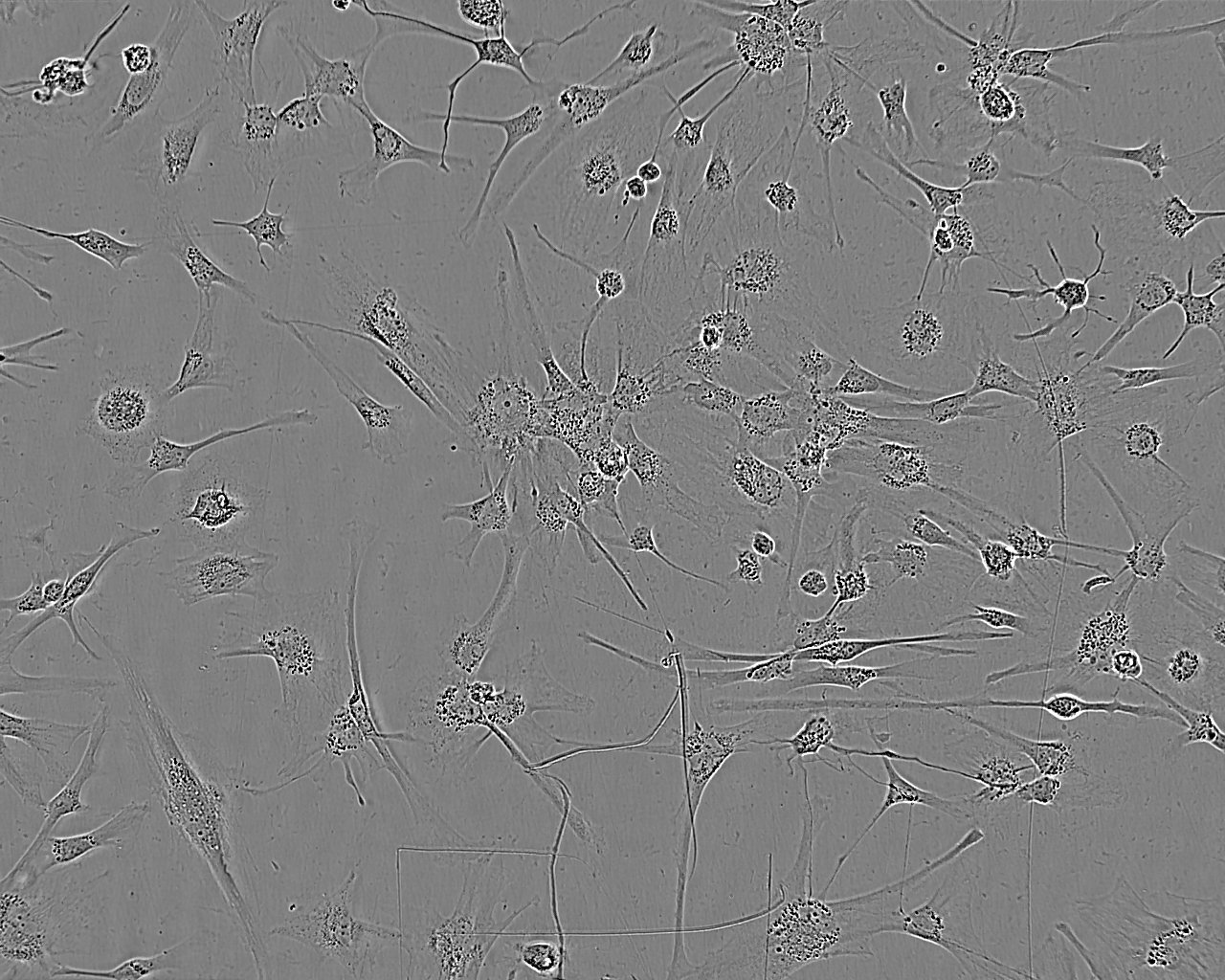

Supplement: Supplementary file 4 — Video microscopy movie 4. 13 frames of the time-lapse microscopy movie (.tif format) corresponding to the resistant GIC clone U3065−c475 when treated with 2μM of CPD-2 alone. (ZIP 20,155 kb) [file 12859_2018_2458_MOESM4_ESM.zip › 1_H23_1_2017y06m29d_19h12m.tif]

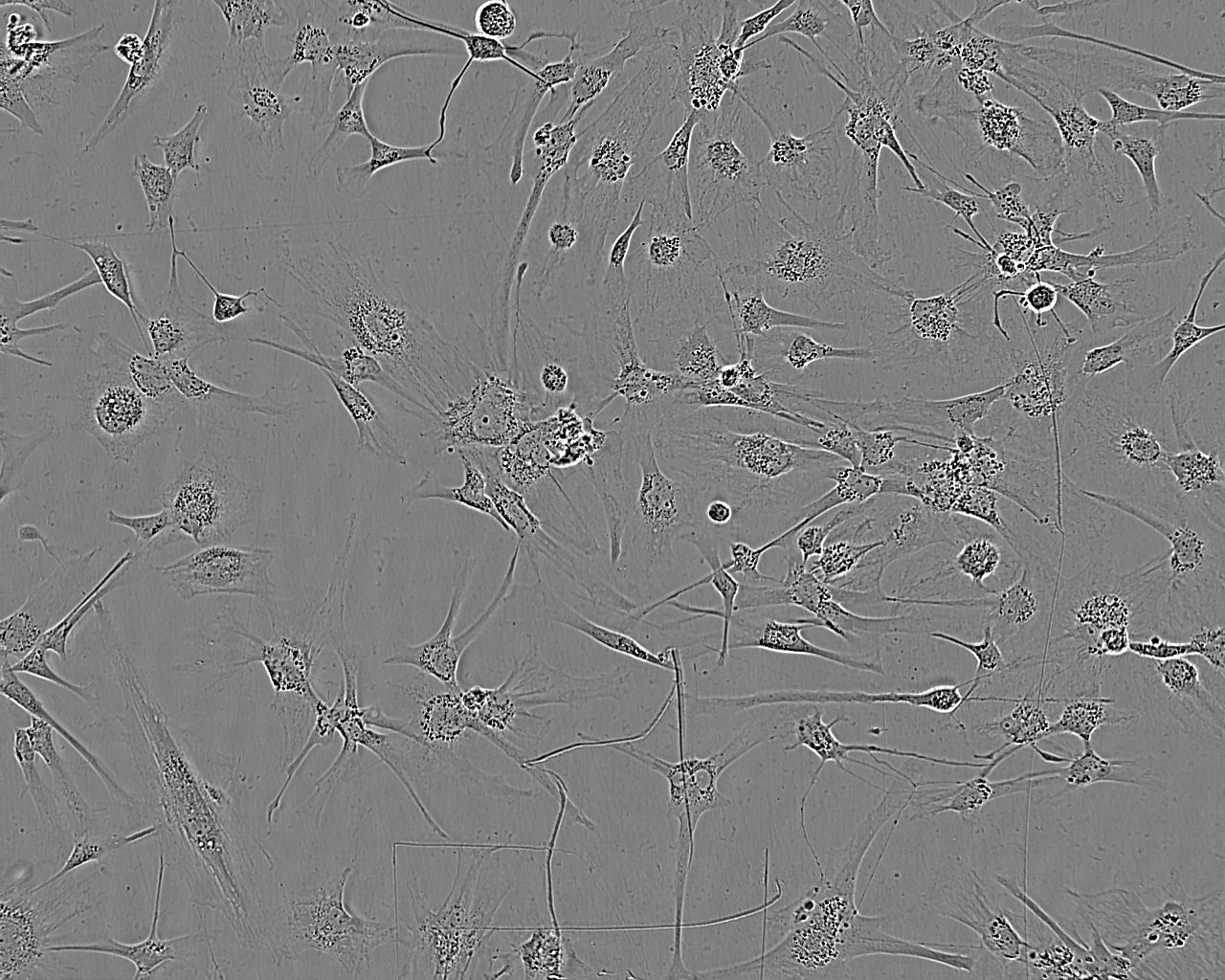

Supplement: Supplementary file 4 — Video microscopy movie 4. 13 frames of the time-lapse microscopy movie (.tif format) corresponding to the resistant GIC clone U3065−c475 when treated with 2μM of CPD-2 alone. (ZIP 20,155 kb) [file 12859_2018_2458_MOESM4_ESM.zip › 1_H23_1_2017y06m30d_01h12m.tif]

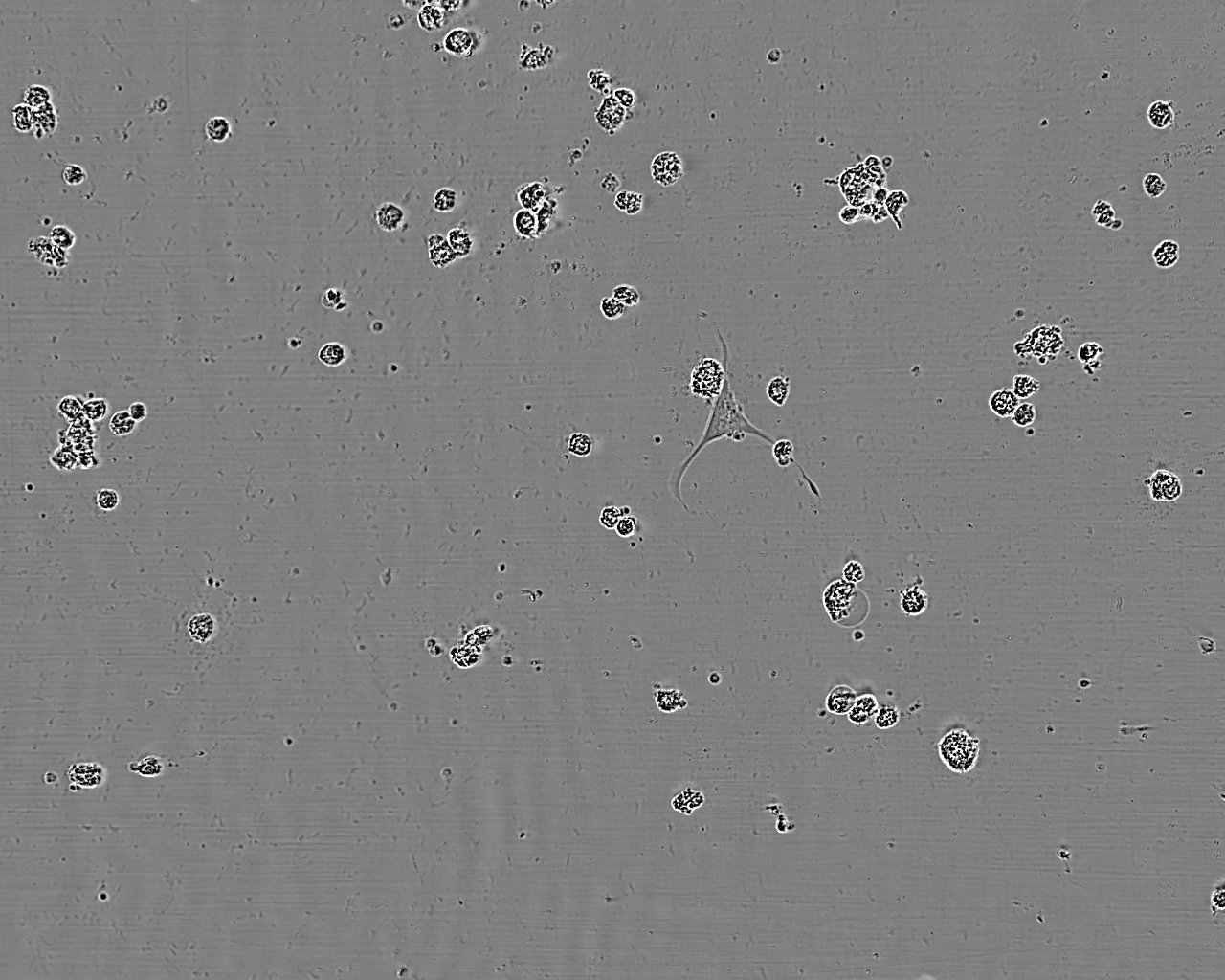

Supplement: Supplementary file 5 — Video microscopy movie 5. 13 frames of the time-lapse microscopy movie (.tif format) corresponding to the resistant GIC clone U3065−c475 when treated with the combination concentration (CPD-1, SAHA) = (2μM, 7μM). (ZIP 17,362 kb) [file 12859_2018_2458_MOESM5_ESM.zip › 1_I18_1_2017y06m30d_06h12m.tif]

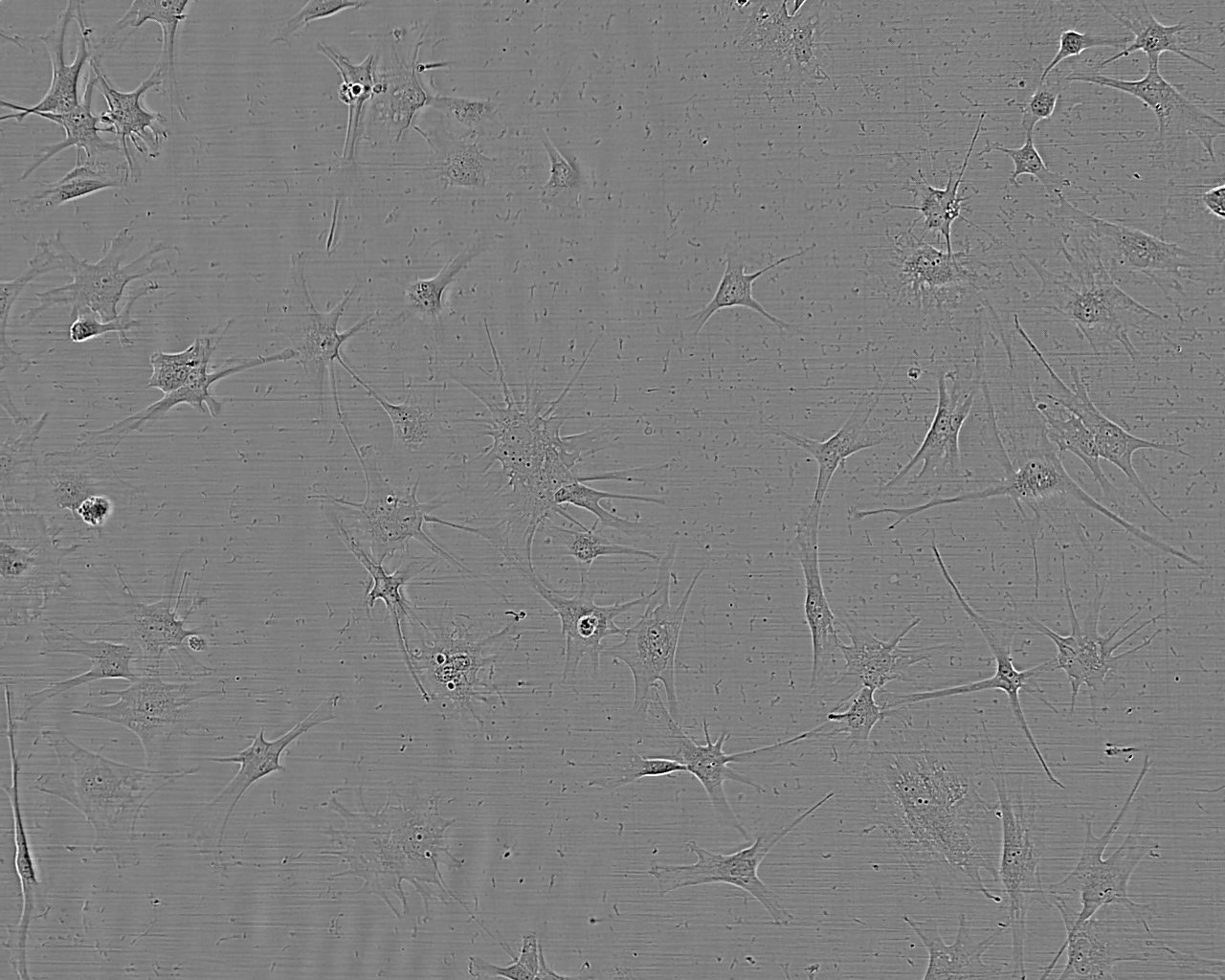

Supplement: Supplementary file 5 — Video microscopy movie 5. 13 frames of the time-lapse microscopy movie (.tif format) corresponding to the resistant GIC clone U3065−c475 when treated with the combination concentration (CPD-1, SAHA) = (2μM, 7μM). (ZIP 17,362 kb) [file 12859_2018_2458_MOESM5_ESM.zip › 1_I18_1_2017y06m27d_07h12m.tif]

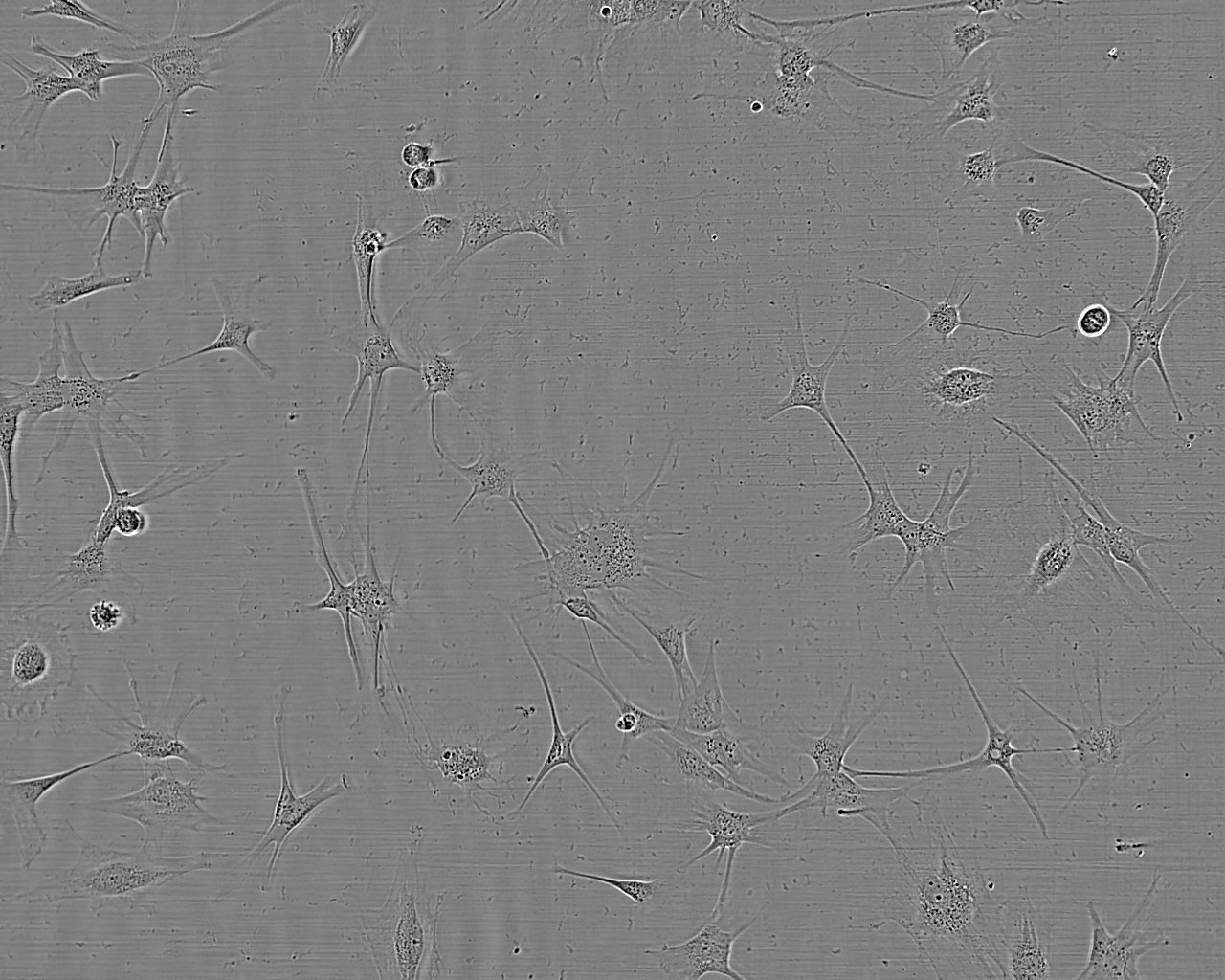

Supplement: Supplementary file 5 — Video microscopy movie 5. 13 frames of the time-lapse microscopy movie (.tif format) corresponding to the resistant GIC clone U3065−c475 when treated with the combination concentration (CPD-1, SAHA) = (2μM, 7μM). (ZIP 17,362 kb) [file 12859_2018_2458_MOESM5_ESM.zip › 1_I18_1_2017y06m27d_13h12m.tif]

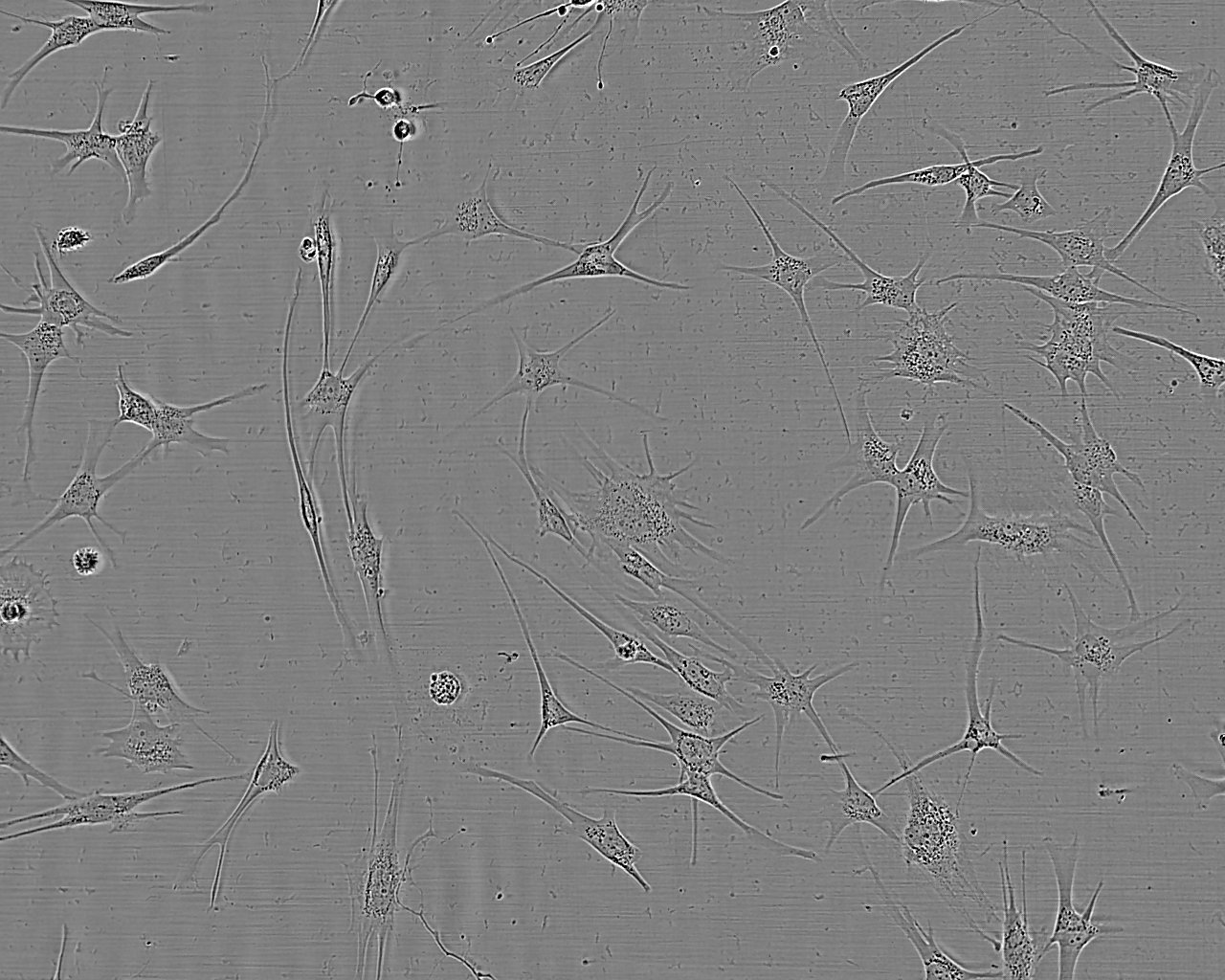

Supplement: Supplementary file 5 — Video microscopy movie 5. 13 frames of the time-lapse microscopy movie (.tif format) corresponding to the resistant GIC clone U3065−c475 when treated with the combination concentration (CPD-1, SAHA) = (2μM, 7μM). (ZIP 17,362 kb) [file 12859_2018_2458_MOESM5_ESM.zip › 1_I18_1_2017y06m27d_19h12m.tif]

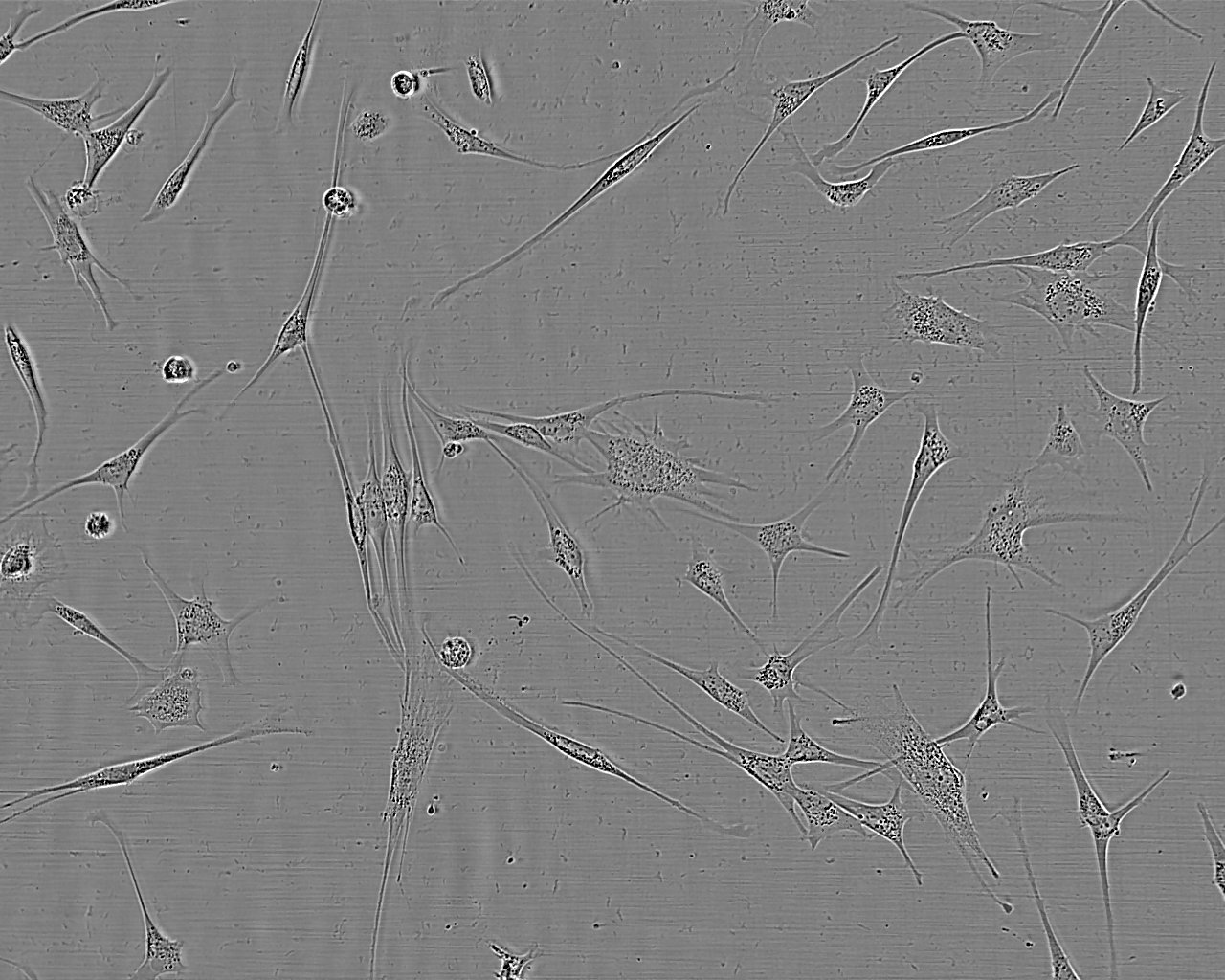

Supplement: Supplementary file 5 — Video microscopy movie 5. 13 frames of the time-lapse microscopy movie (.tif format) corresponding to the resistant GIC clone U3065−c475 when treated with the combination concentration (CPD-1, SAHA) = (2μM, 7μM). (ZIP 17,362 kb) [file 12859_2018_2458_MOESM5_ESM.zip › 1_I18_1_2017y06m28d_01h12m.tif]

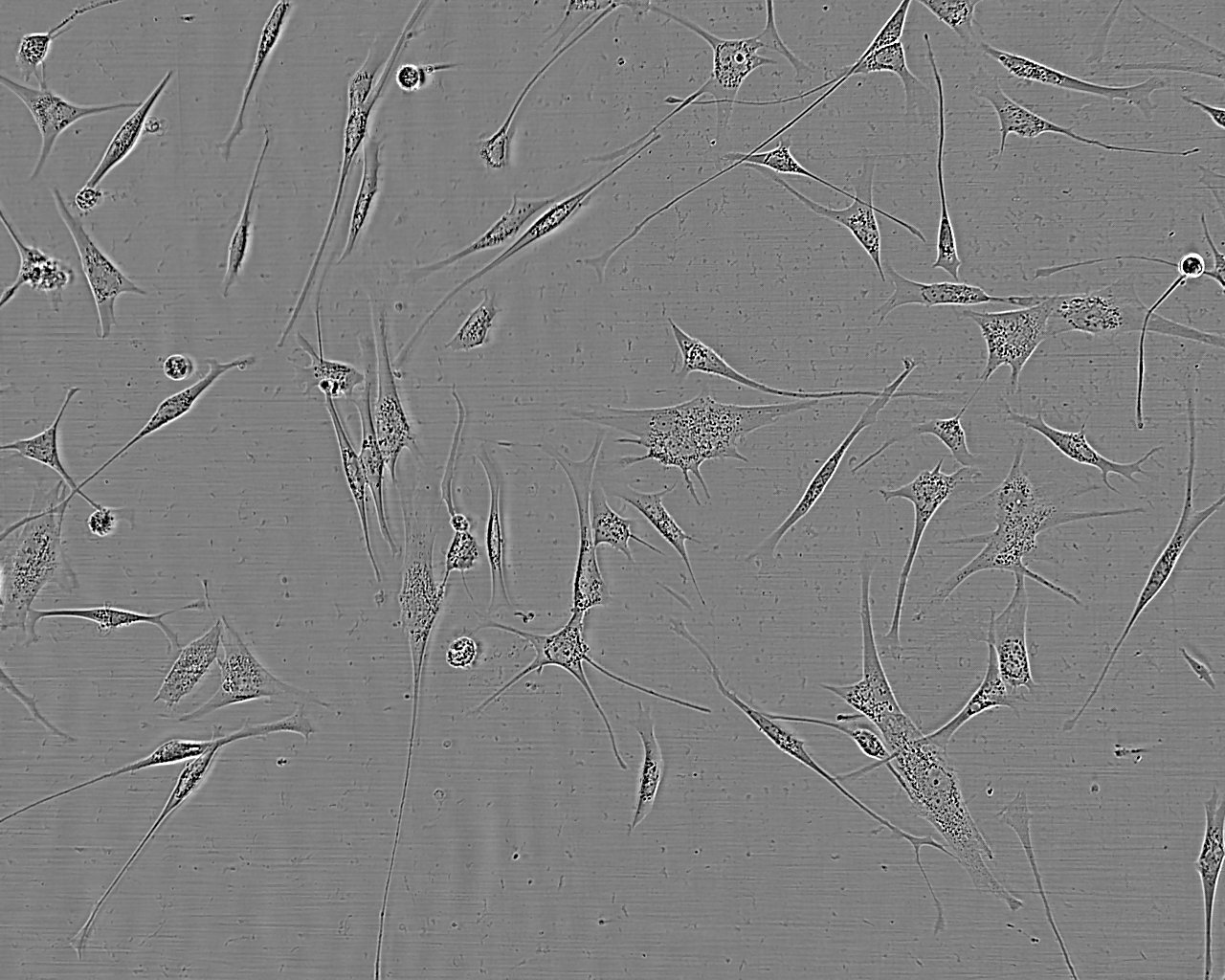

Supplement: Supplementary file 5 — Video microscopy movie 5. 13 frames of the time-lapse microscopy movie (.tif format) corresponding to the resistant GIC clone U3065−c475 when treated with the combination concentration (CPD-1, SAHA) = (2μM, 7μM). (ZIP 17,362 kb) [file 12859_2018_2458_MOESM5_ESM.zip › 1_I18_1_2017y06m28d_07h12m.tif]

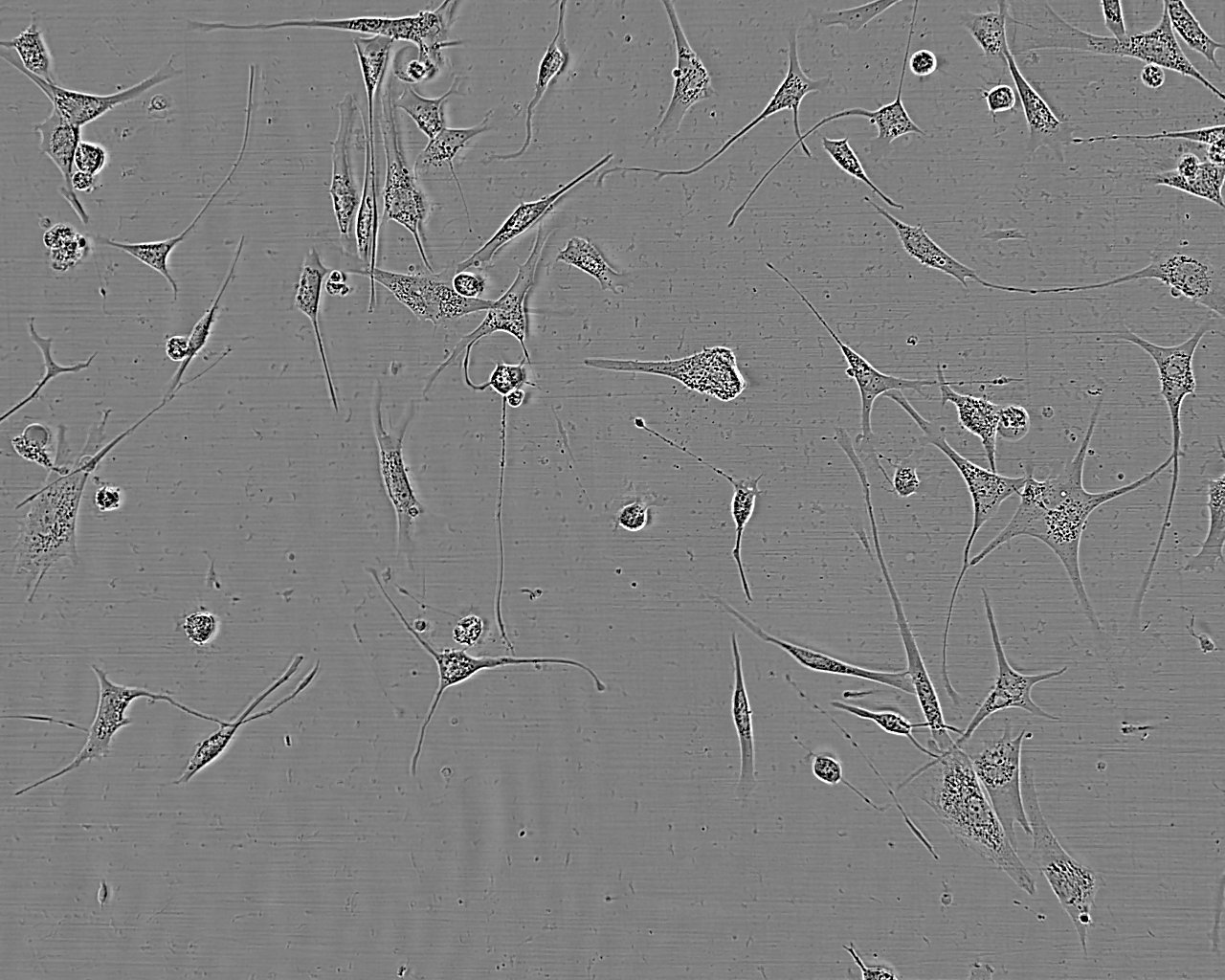

Supplement: Supplementary file 5 — Video microscopy movie 5. 13 frames of the time-lapse microscopy movie (.tif format) corresponding to the resistant GIC clone U3065−c475 when treated with the combination concentration (CPD-1, SAHA) = (2μM, 7μM). (ZIP 17,362 kb) [file 12859_2018_2458_MOESM5_ESM.zip › 1_I18_1_2017y06m28d_13h12m.tif]

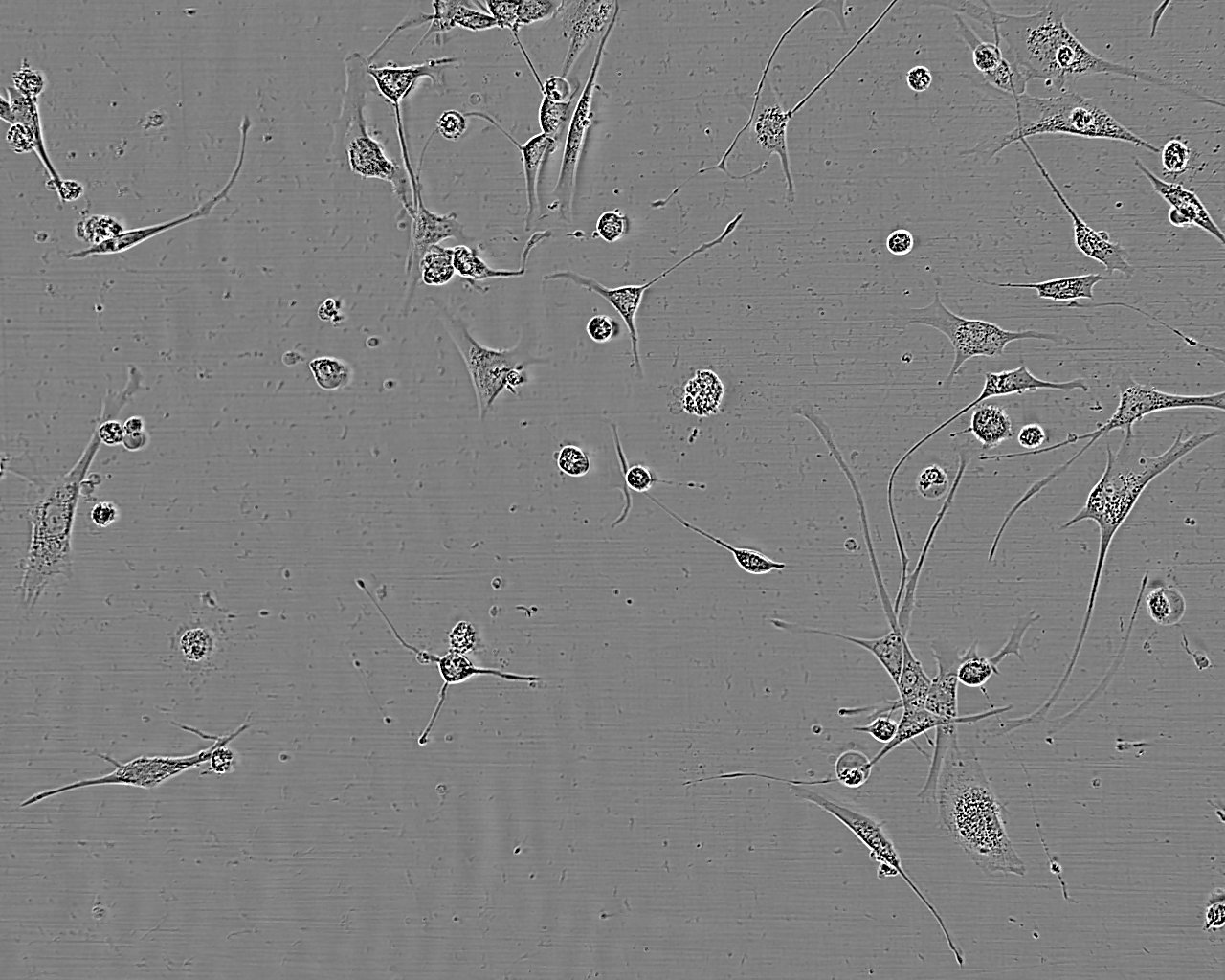

Supplement: Supplementary file 5 — Video microscopy movie 5. 13 frames of the time-lapse microscopy movie (.tif format) corresponding to the resistant GIC clone U3065−c475 when treated with the combination concentration (CPD-1, SAHA) = (2μM, 7μM). (ZIP 17,362 kb) [file 12859_2018_2458_MOESM5_ESM.zip › 1_I18_1_2017y06m28d_19h12m.tif]

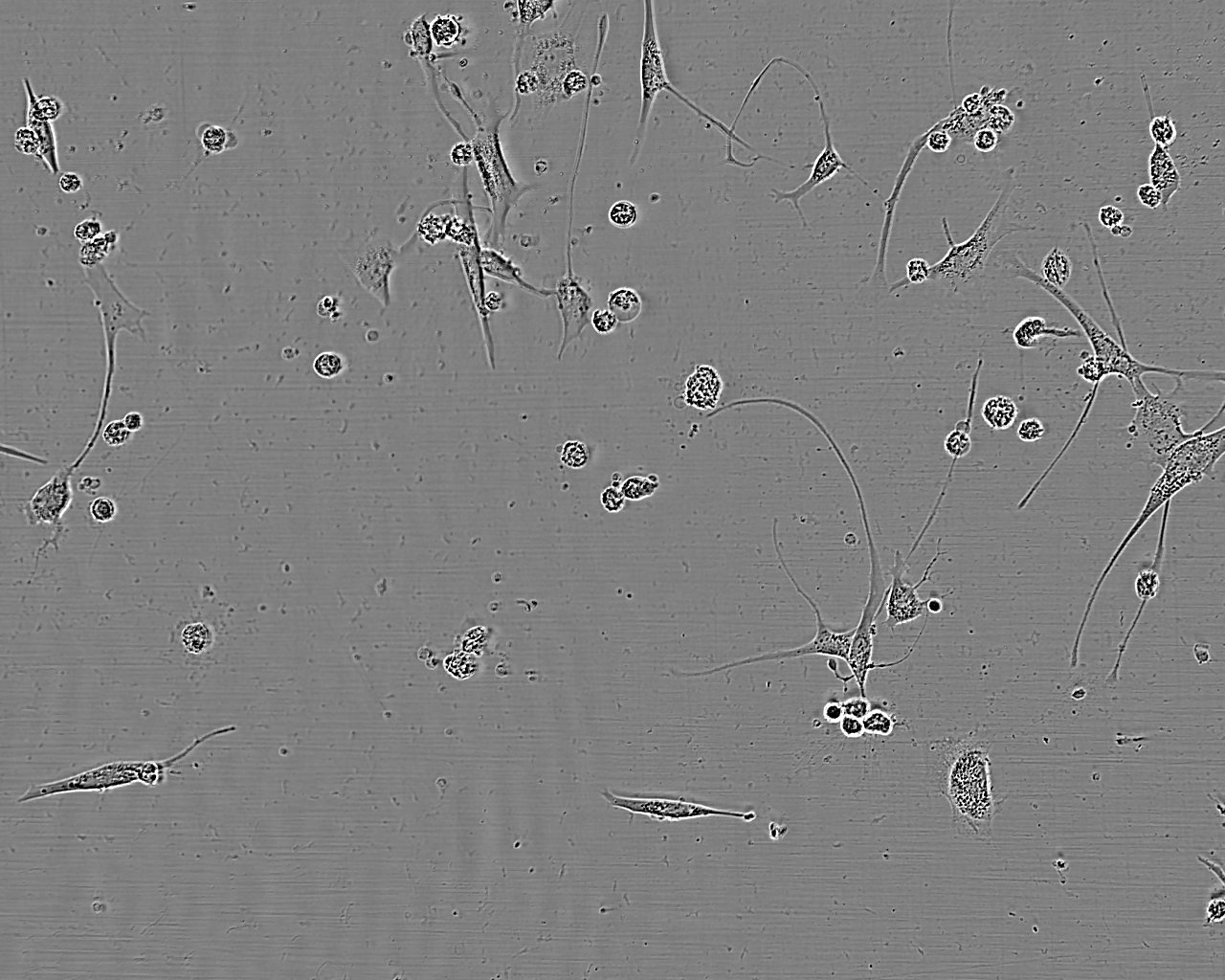

Supplement: Supplementary file 5 — Video microscopy movie 5. 13 frames of the time-lapse microscopy movie (.tif format) corresponding to the resistant GIC clone U3065−c475 when treated with the combination concentration (CPD-1, SAHA) = (2μM, 7μM). (ZIP 17,362 kb) [file 12859_2018_2458_MOESM5_ESM.zip › 1_I18_1_2017y06m29d_01h12m.tif]

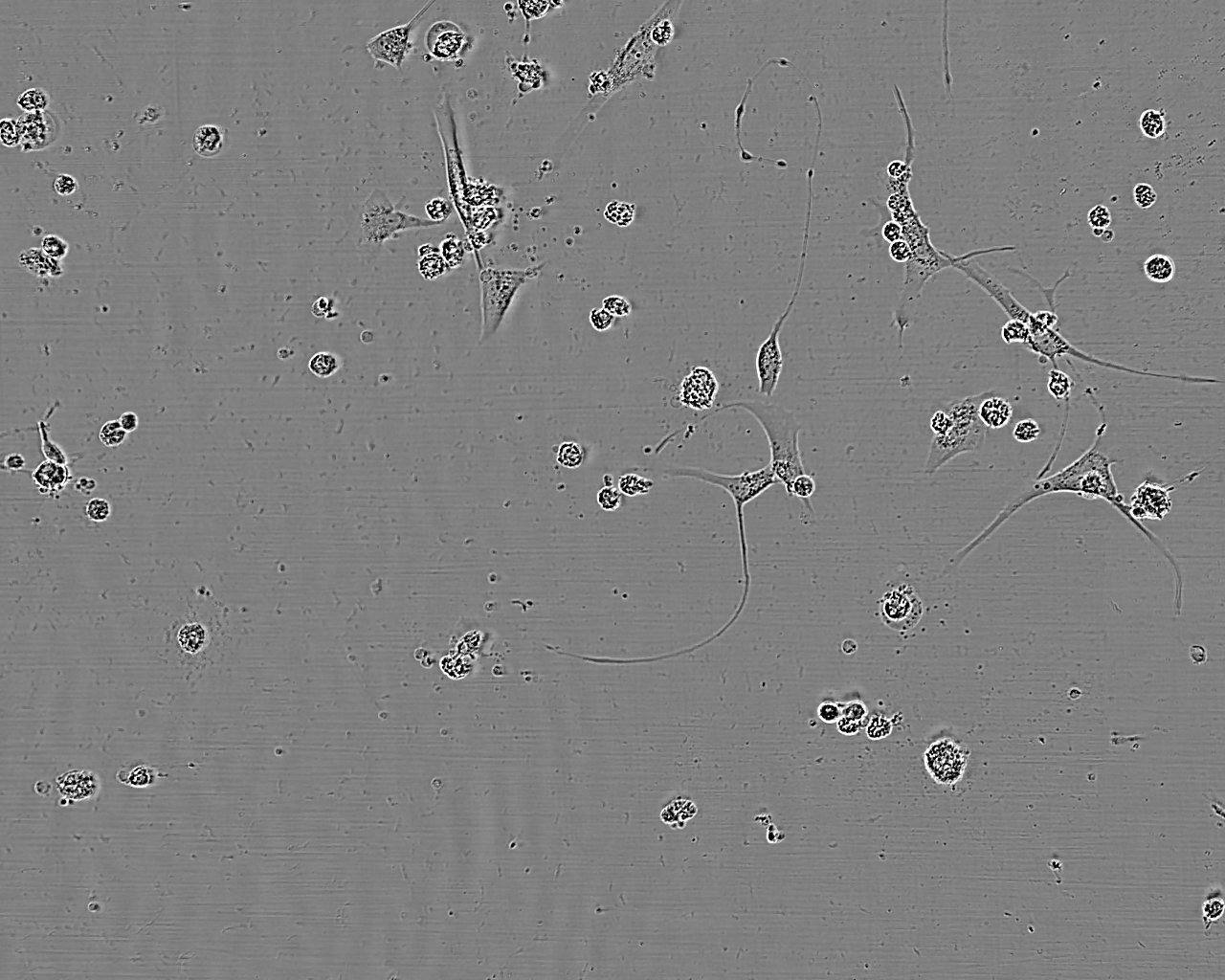

Supplement: Supplementary file 5 — Video microscopy movie 5. 13 frames of the time-lapse microscopy movie (.tif format) corresponding to the resistant GIC clone U3065−c475 when treated with the combination concentration (CPD-1, SAHA) = (2μM, 7μM). (ZIP 17,362 kb) [file 12859_2018_2458_MOESM5_ESM.zip › 1_I18_1_2017y06m29d_07h12m.tif]

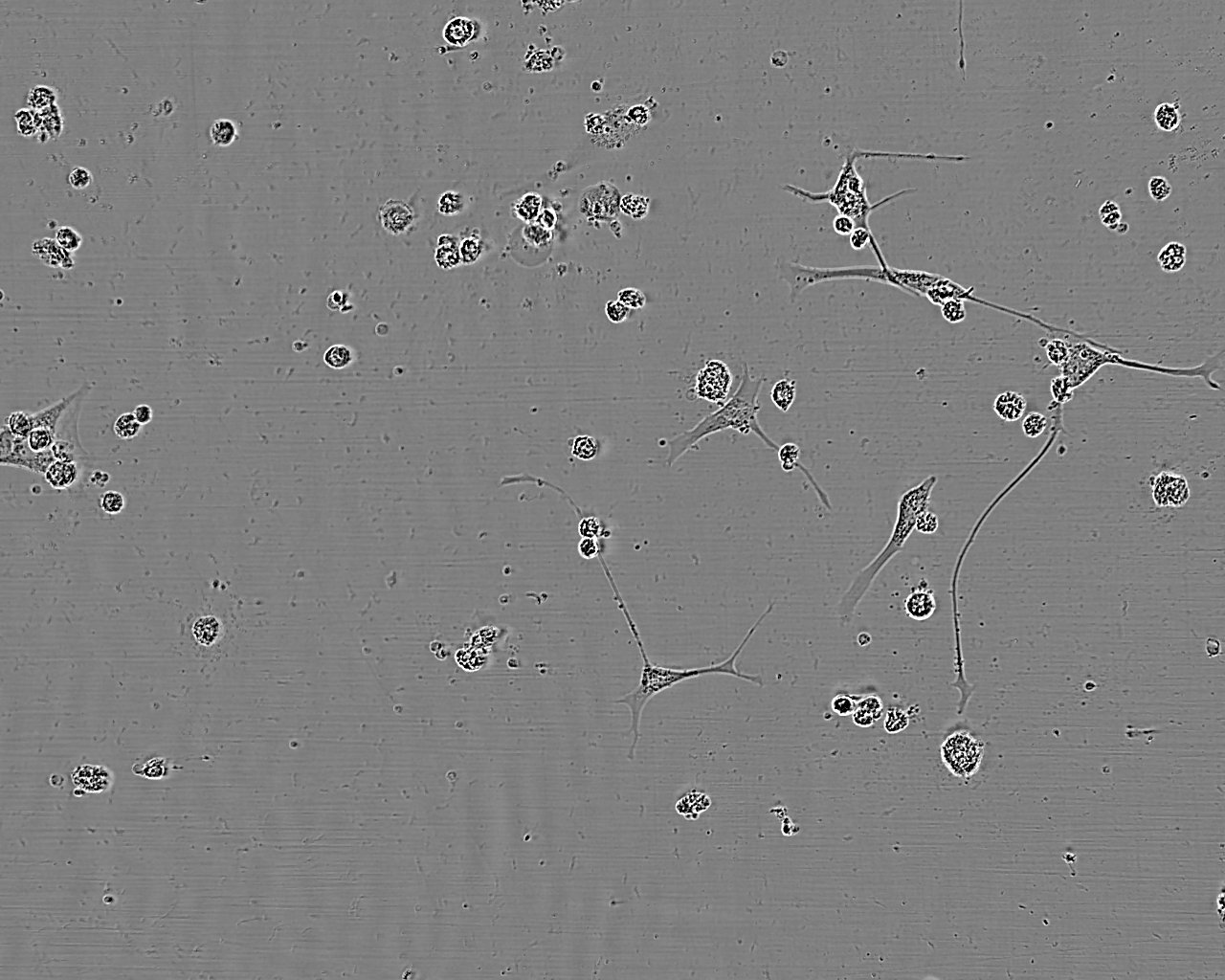

Supplement: Supplementary file 5 — Video microscopy movie 5. 13 frames of the time-lapse microscopy movie (.tif format) corresponding to the resistant GIC clone U3065−c475 when treated with the combination concentration (CPD-1, SAHA) = (2μM, 7μM). (ZIP 17,362 kb) [file 12859_2018_2458_MOESM5_ESM.zip › 1_I18_1_2017y06m29d_13h12m.tif]

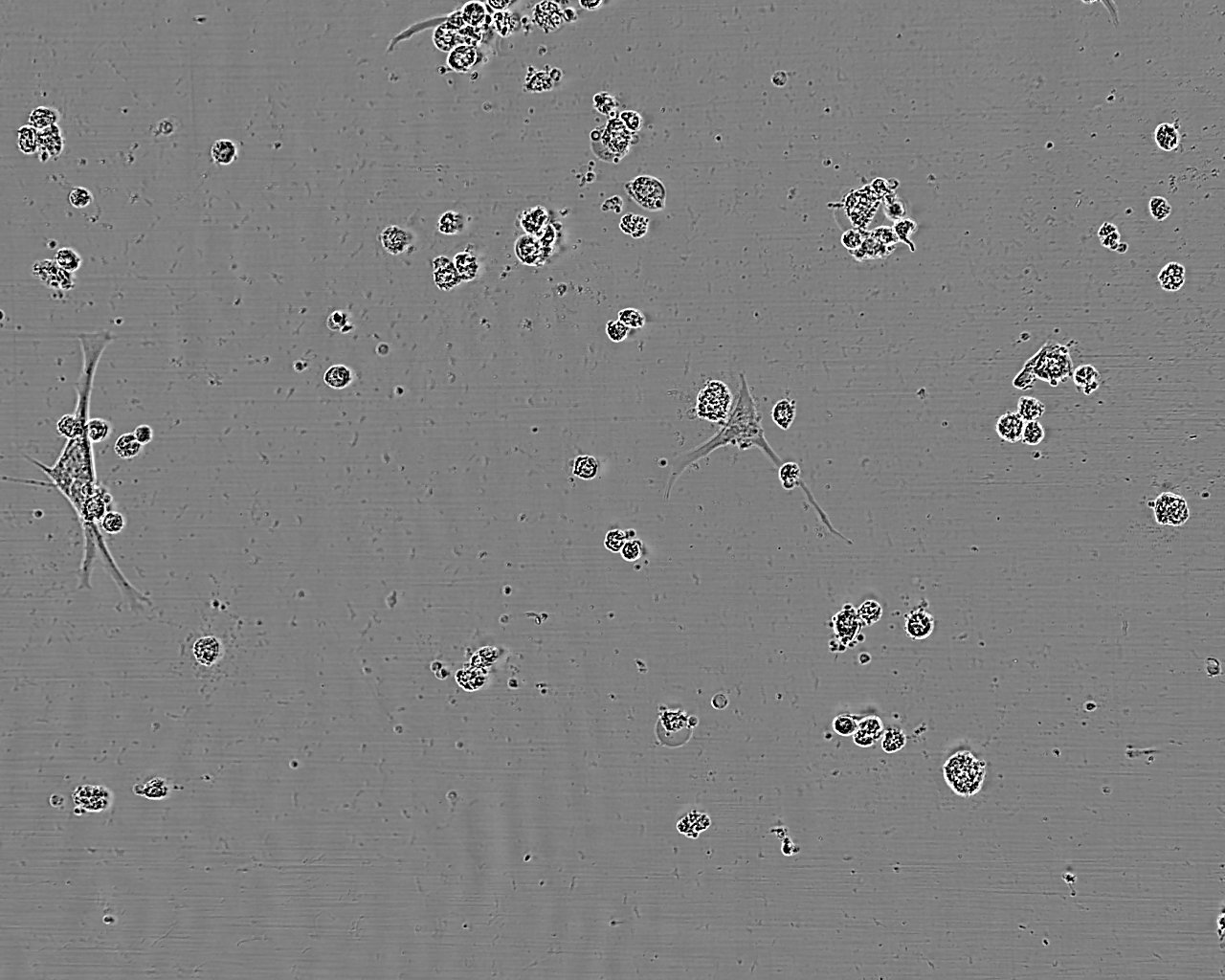

Supplement: Supplementary file 5 — Video microscopy movie 5. 13 frames of the time-lapse microscopy movie (.tif format) corresponding to the resistant GIC clone U3065−c475 when treated with the combination concentration (CPD-1, SAHA) = (2μM, 7μM). (ZIP 17,362 kb) [file 12859_2018_2458_MOESM5_ESM.zip › 1_I18_1_2017y06m29d_19h12m.tif]

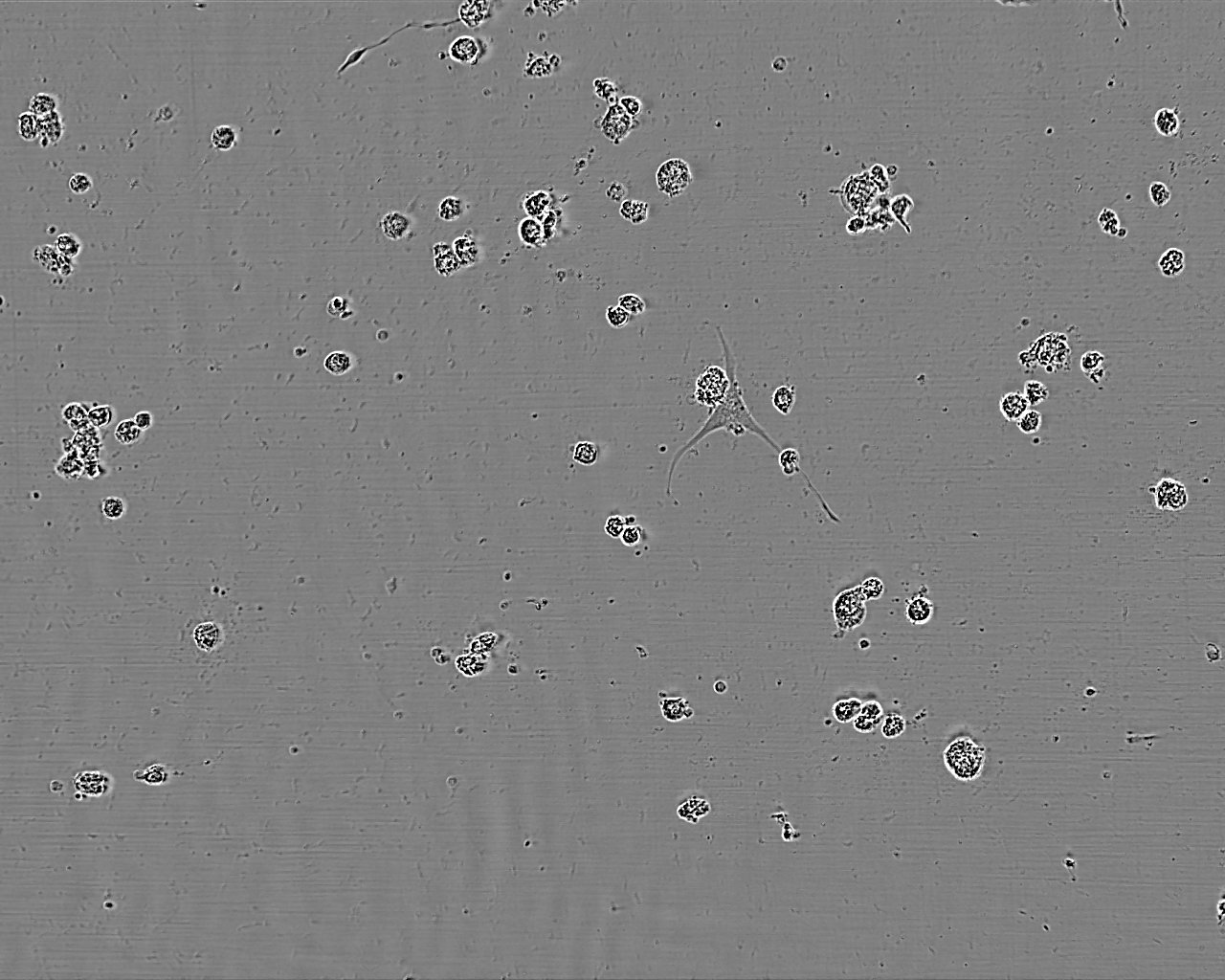

Supplement: Supplementary file 5 — Video microscopy movie 5. 13 frames of the time-lapse microscopy movie (.tif format) corresponding to the resistant GIC clone U3065−c475 when treated with the combination concentration (CPD-1, SAHA) = (2μM, 7μM). (ZIP 17,362 kb) [file 12859_2018_2458_MOESM5_ESM.zip › 1_I18_1_2017y06m30d_01h12m.tif]

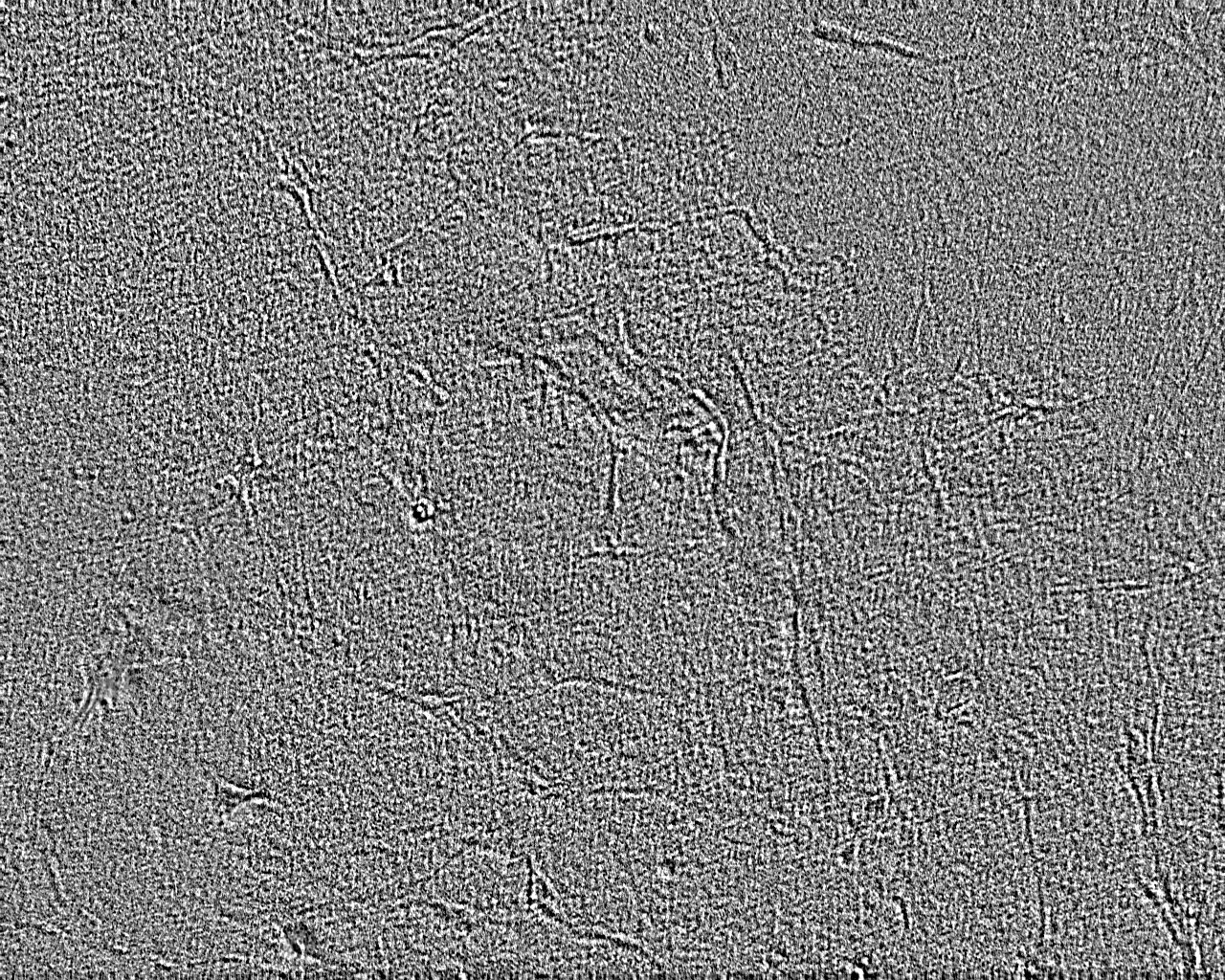

Supplement: Supplementary file 6 — Video microscopy movie 6. Time-lapse microscopy movie (.tif format) showing effects consistent with a bacterial contamination from time point 6 onwards. (ZIP 23,423 kb) [file 12859_2018_2458_MOESM6_ESM.zip › 1_M20_1_2017y06m30d_07h00m.tif]

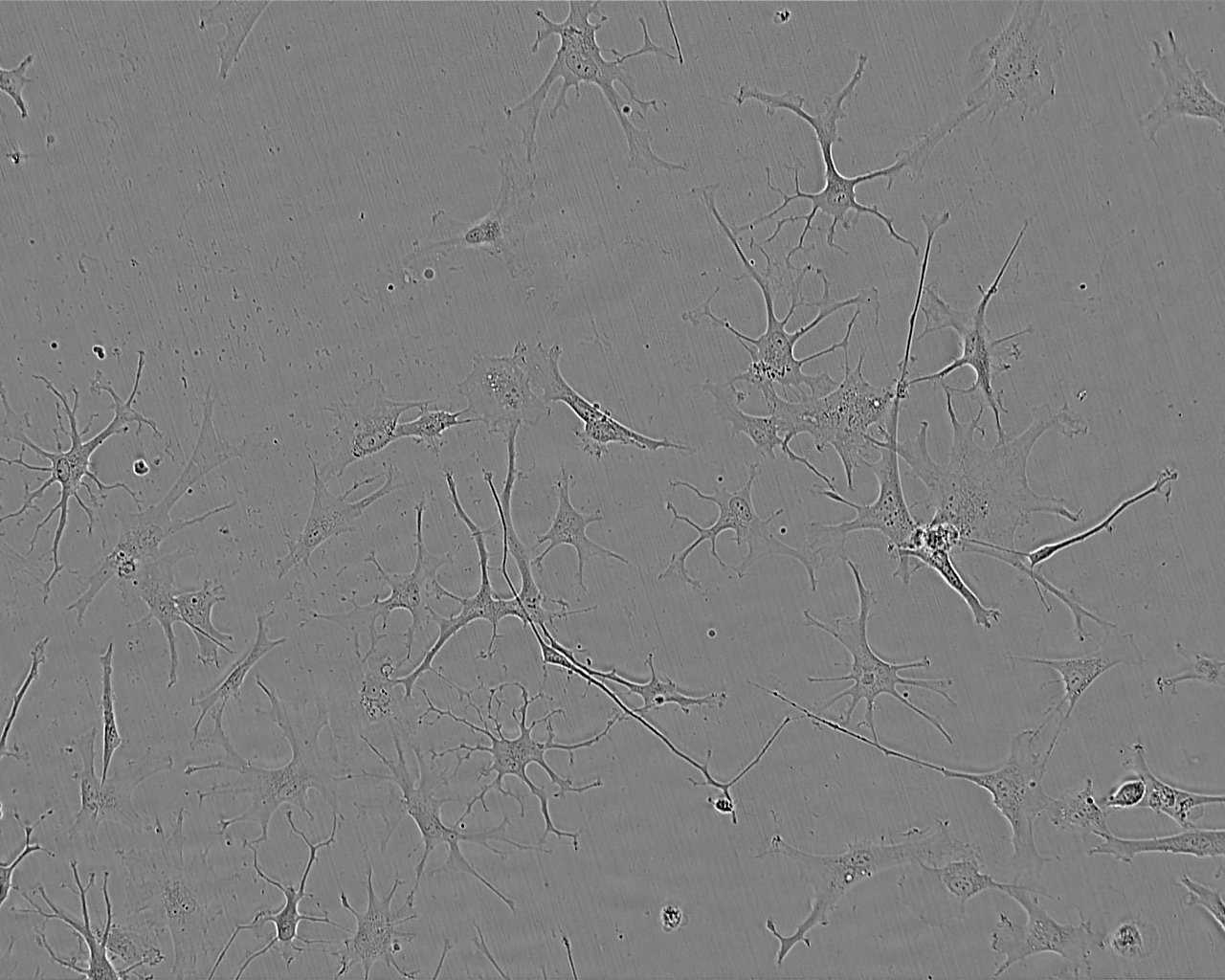

Supplement: Supplementary file 6 — Video microscopy movie 6. Time-lapse microscopy movie (.tif format) showing effects consistent with a bacterial contamination from time point 6 onwards. (ZIP 23,423 kb) [file 12859_2018_2458_MOESM6_ESM.zip › 1_M20_1_2017y06m27d_08h00m.tif]

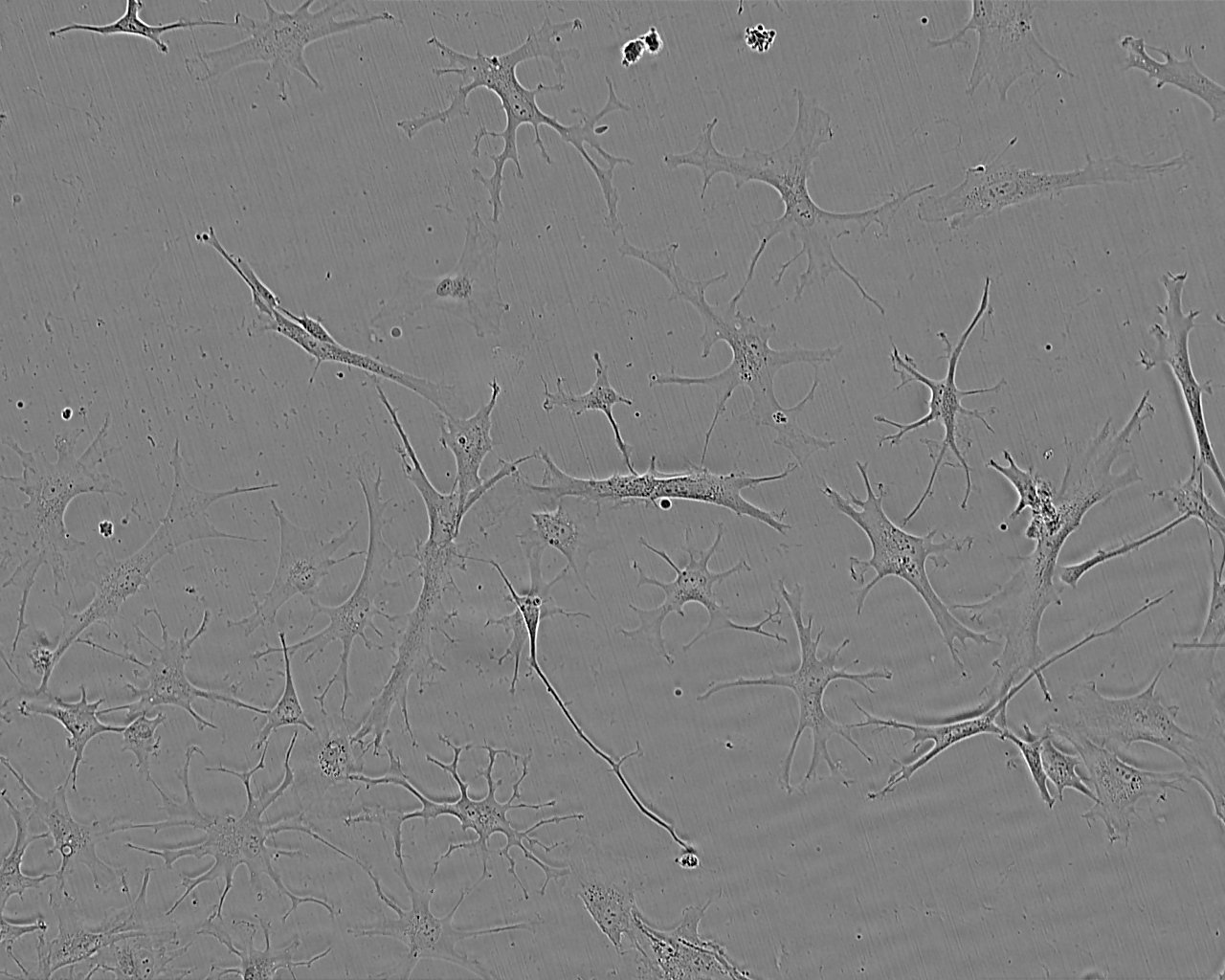

Supplement: Supplementary file 6 — Video microscopy movie 6. Time-lapse microscopy movie (.tif format) showing effects consistent with a bacterial contamination from time point 6 onwards. (ZIP 23,423 kb) [file 12859_2018_2458_MOESM6_ESM.zip › 1_M20_1_2017y06m27d_14h00m.tif]

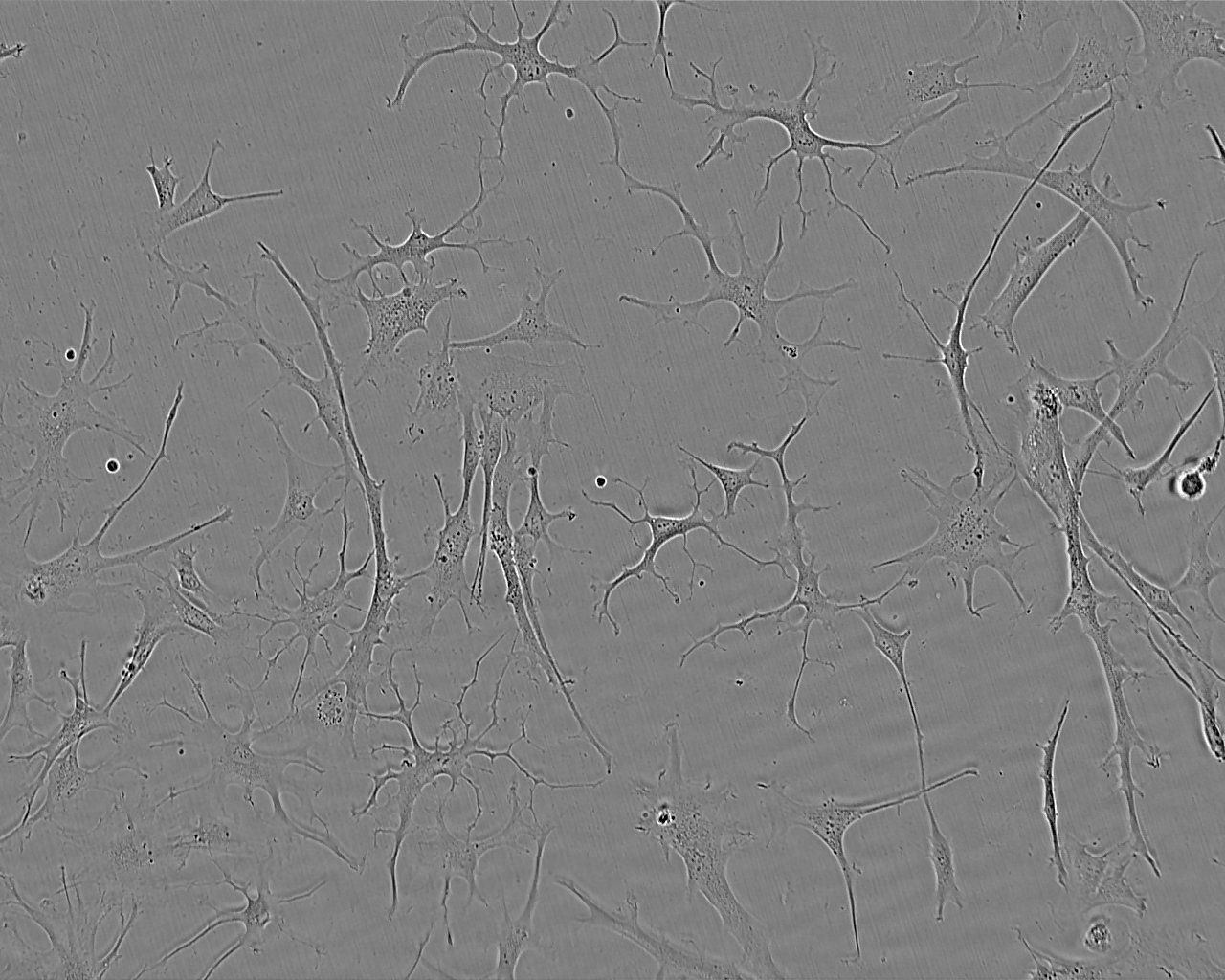

Supplement: Supplementary file 6 — Video microscopy movie 6. Time-lapse microscopy movie (.tif format) showing effects consistent with a bacterial contamination from time point 6 onwards. (ZIP 23,423 kb) [file 12859_2018_2458_MOESM6_ESM.zip › 1_M20_1_2017y06m27d_20h00m.tif]

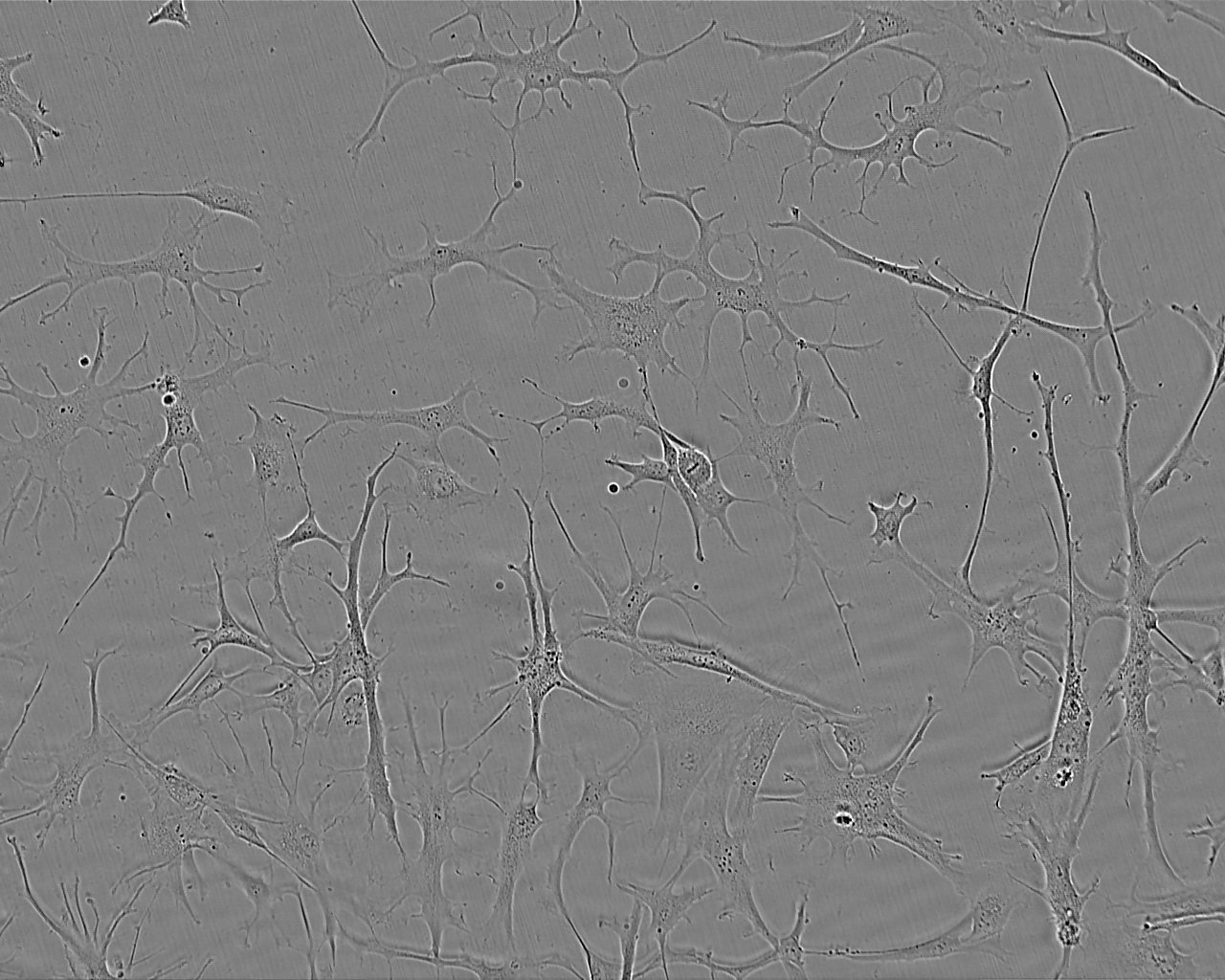

Supplement: Supplementary file 6 — Video microscopy movie 6. Time-lapse microscopy movie (.tif format) showing effects consistent with a bacterial contamination from time point 6 onwards. (ZIP 23,423 kb) [file 12859_2018_2458_MOESM6_ESM.zip › 1_M20_1_2017y06m28d_02h00m.tif]

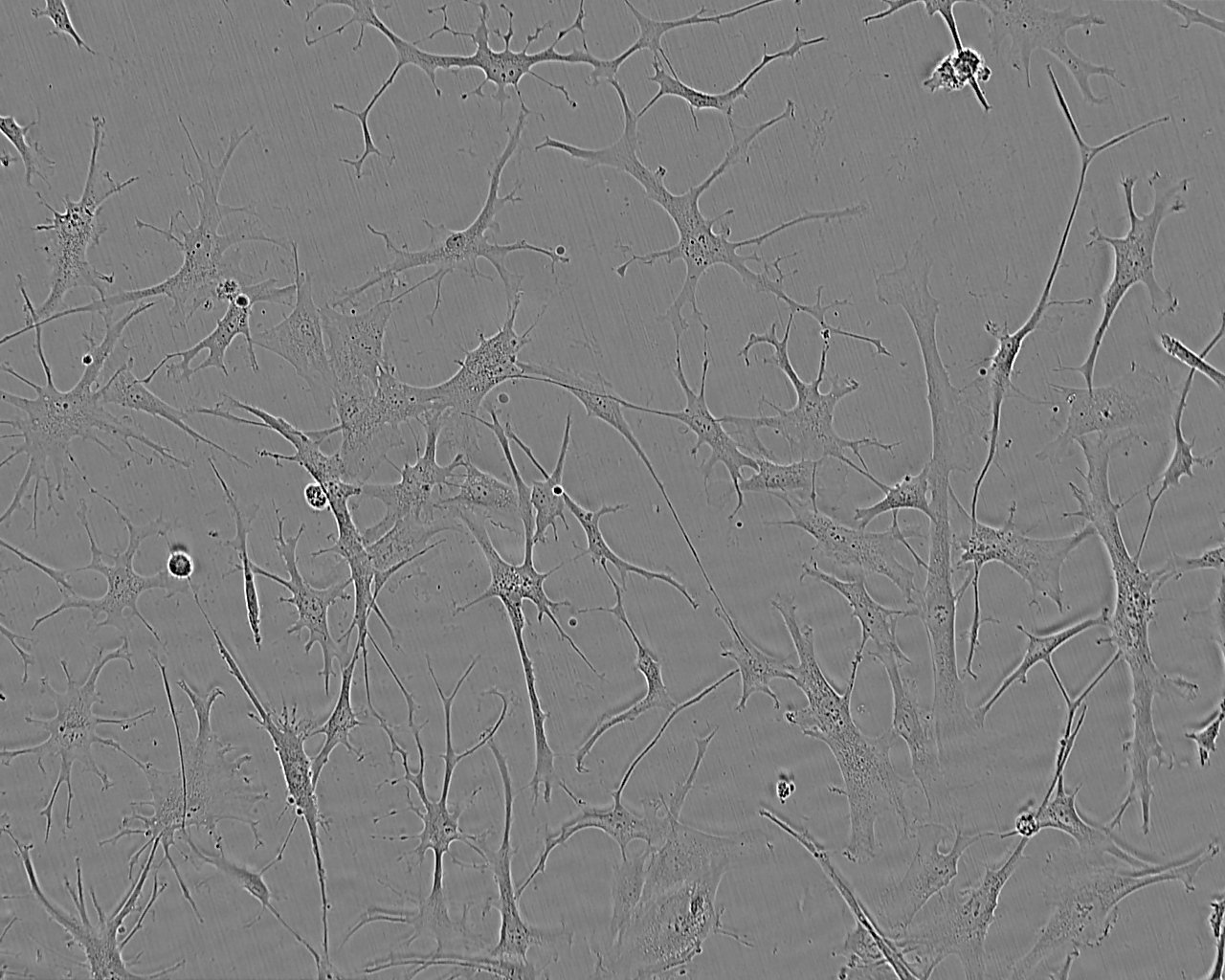

Supplement: Supplementary file 6 — Video microscopy movie 6. Time-lapse microscopy movie (.tif format) showing effects consistent with a bacterial contamination from time point 6 onwards. (ZIP 23,423 kb) [file 12859_2018_2458_MOESM6_ESM.zip › 1_M20_1_2017y06m28d_08h00m.tif]

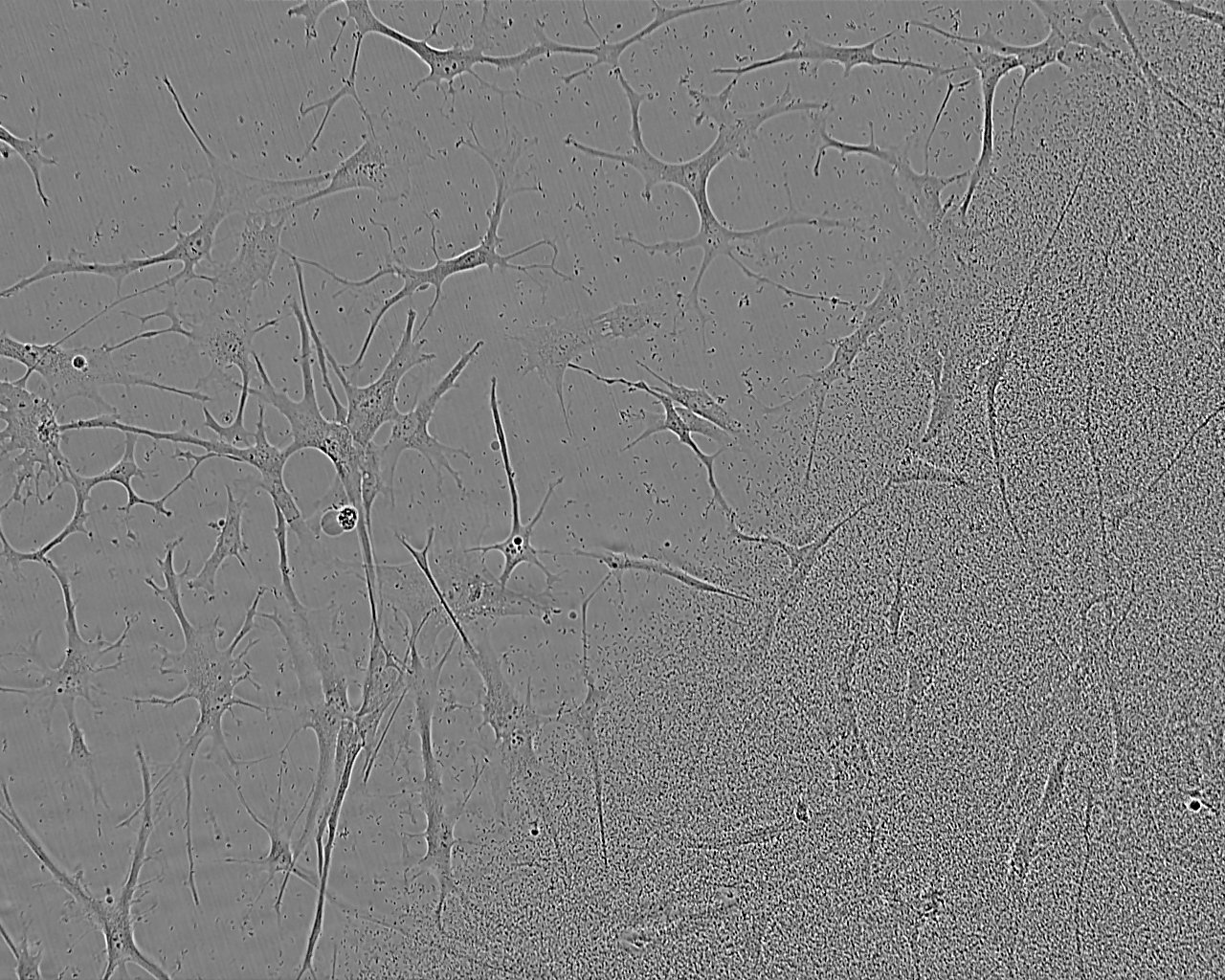

Supplement: Supplementary file 6 — Video microscopy movie 6. Time-lapse microscopy movie (.tif format) showing effects consistent with a bacterial contamination from time point 6 onwards. (ZIP 23,423 kb) [file 12859_2018_2458_MOESM6_ESM.zip › 1_M20_1_2017y06m28d_14h00m.tif]

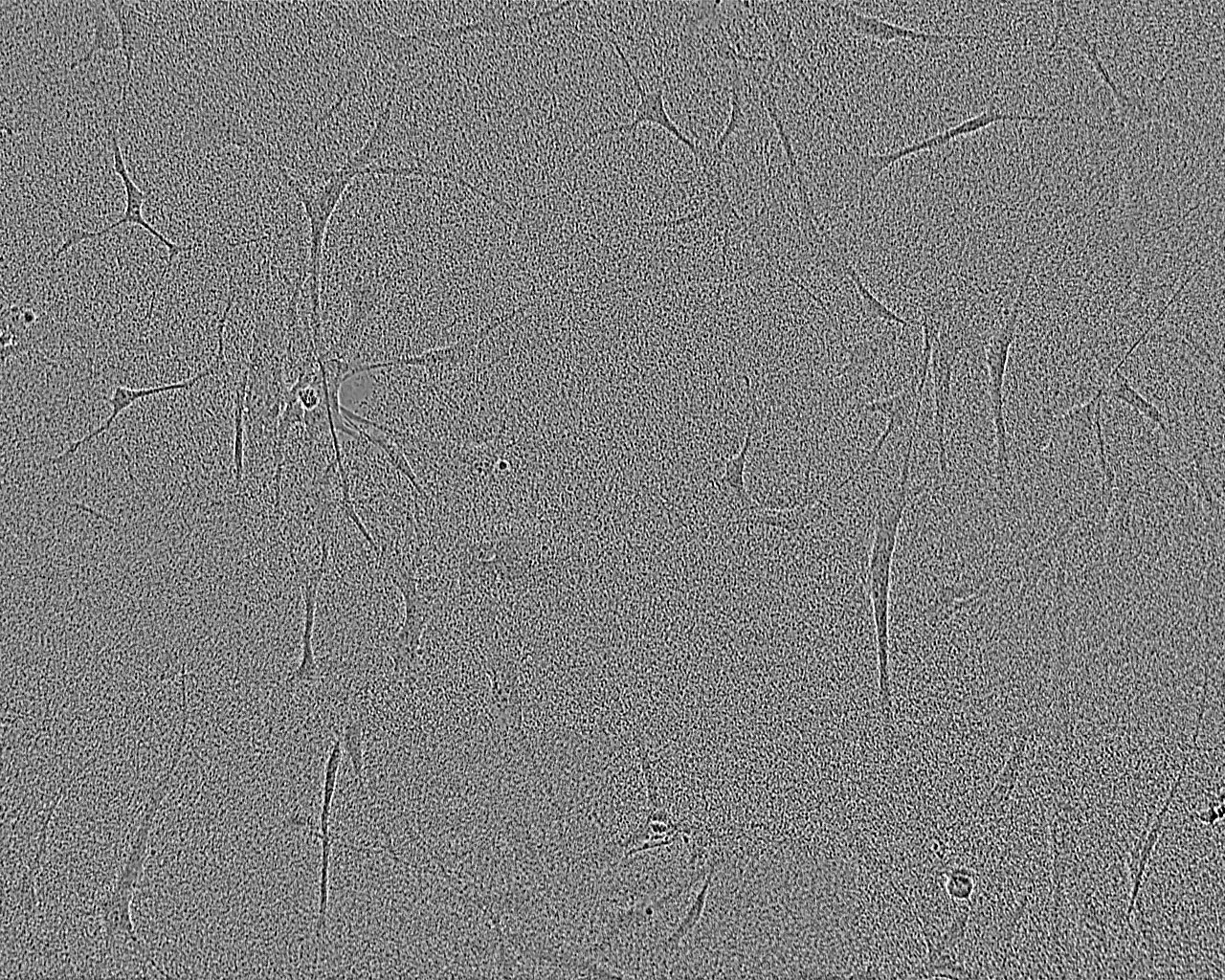

Supplement: Supplementary file 6 — Video microscopy movie 6. Time-lapse microscopy movie (.tif format) showing effects consistent with a bacterial contamination from time point 6 onwards. (ZIP 23,423 kb) [file 12859_2018_2458_MOESM6_ESM.zip › 1_M20_1_2017y06m28d_20h00m.tif]

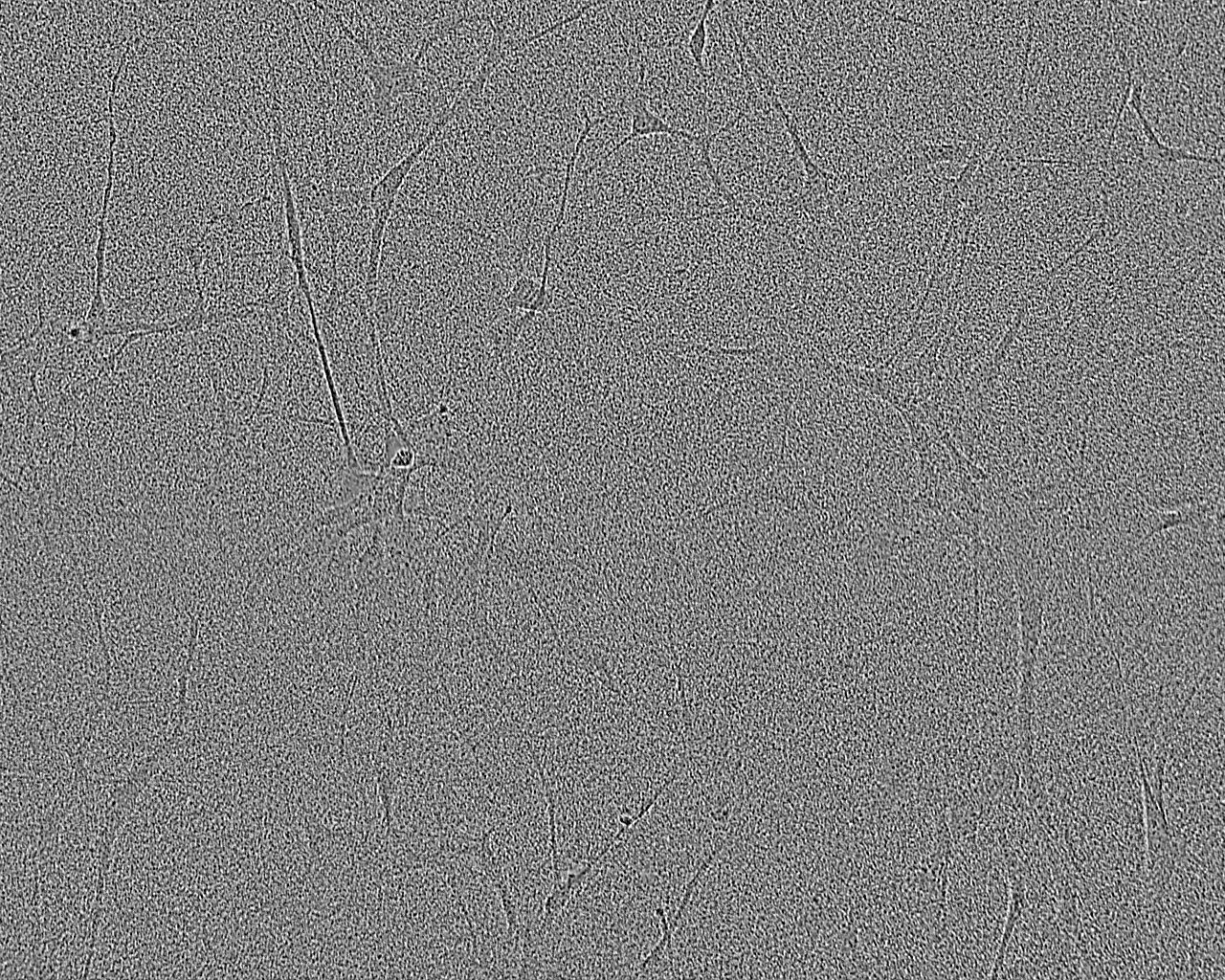

Supplement: Supplementary file 6 — Video microscopy movie 6. Time-lapse microscopy movie (.tif format) showing effects consistent with a bacterial contamination from time point 6 onwards. (ZIP 23,423 kb) [file 12859_2018_2458_MOESM6_ESM.zip › 1_M20_1_2017y06m29d_02h00m.tif]

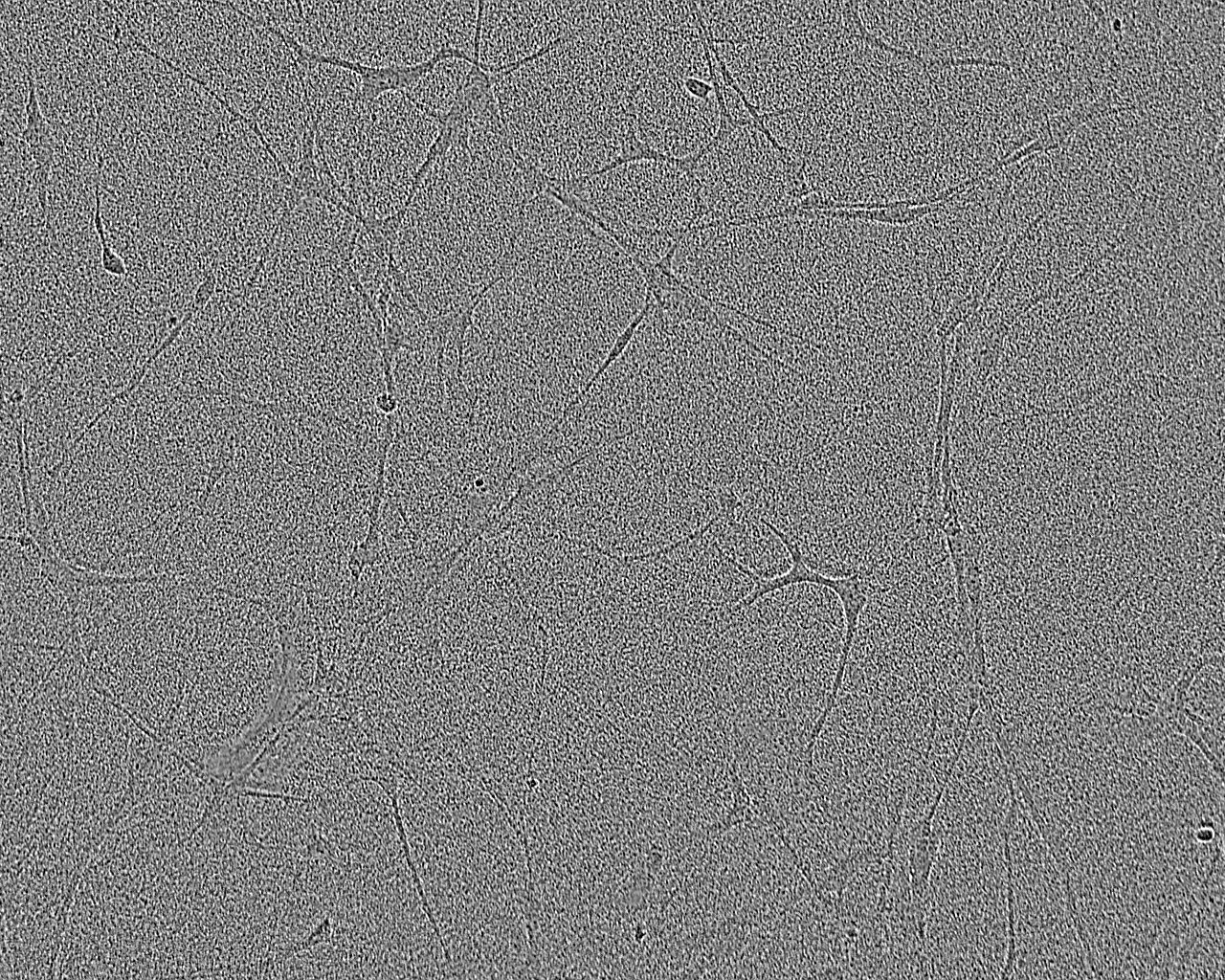

Supplement: Supplementary file 6 — Video microscopy movie 6. Time-lapse microscopy movie (.tif format) showing effects consistent with a bacterial contamination from time point 6 onwards. (ZIP 23,423 kb) [file 12859_2018_2458_MOESM6_ESM.zip › 1_M20_1_2017y06m29d_08h00m.tif]

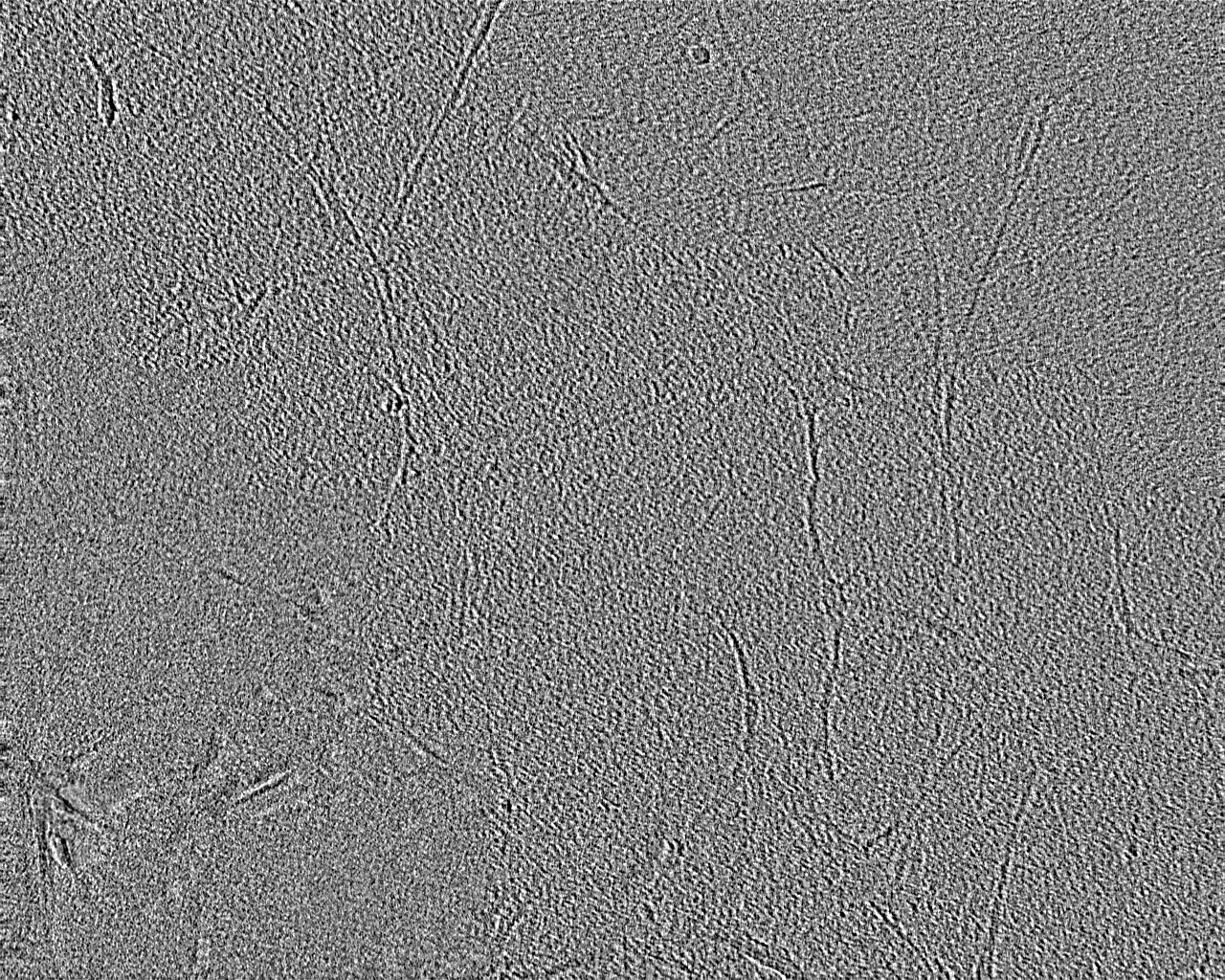

Supplement: Supplementary file 6 — Video microscopy movie 6. Time-lapse microscopy movie (.tif format) showing effects consistent with a bacterial contamination from time point 6 onwards. (ZIP 23,423 kb) [file 12859_2018_2458_MOESM6_ESM.zip › 1_M20_1_2017y06m29d_14h00m.tif]

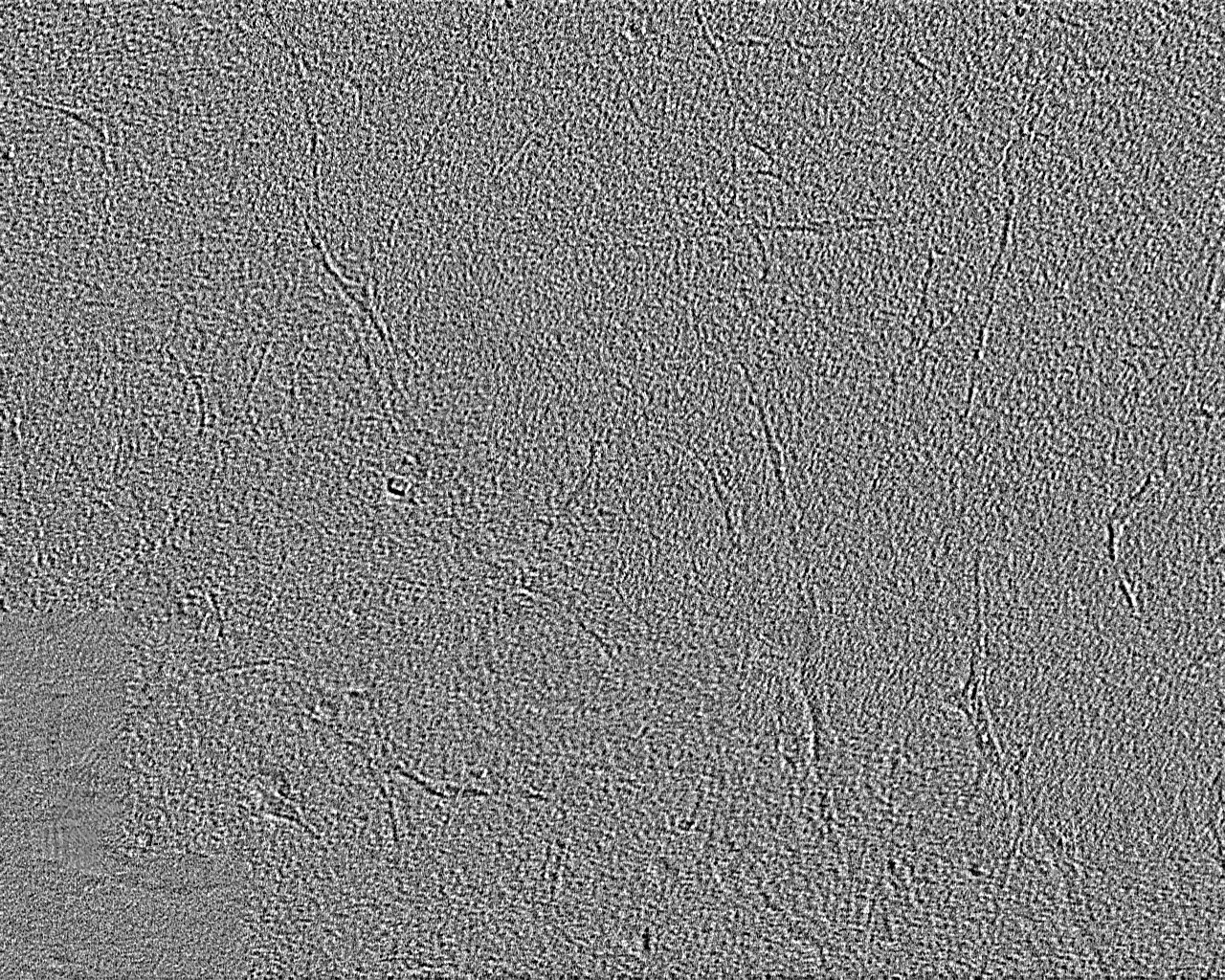

Supplement: Supplementary file 6 — Video microscopy movie 6. Time-lapse microscopy movie (.tif format) showing effects consistent with a bacterial contamination from time point 6 onwards. (ZIP 23,423 kb) [file 12859_2018_2458_MOESM6_ESM.zip › 1_M20_1_2017y06m29d_20h00m.tif]

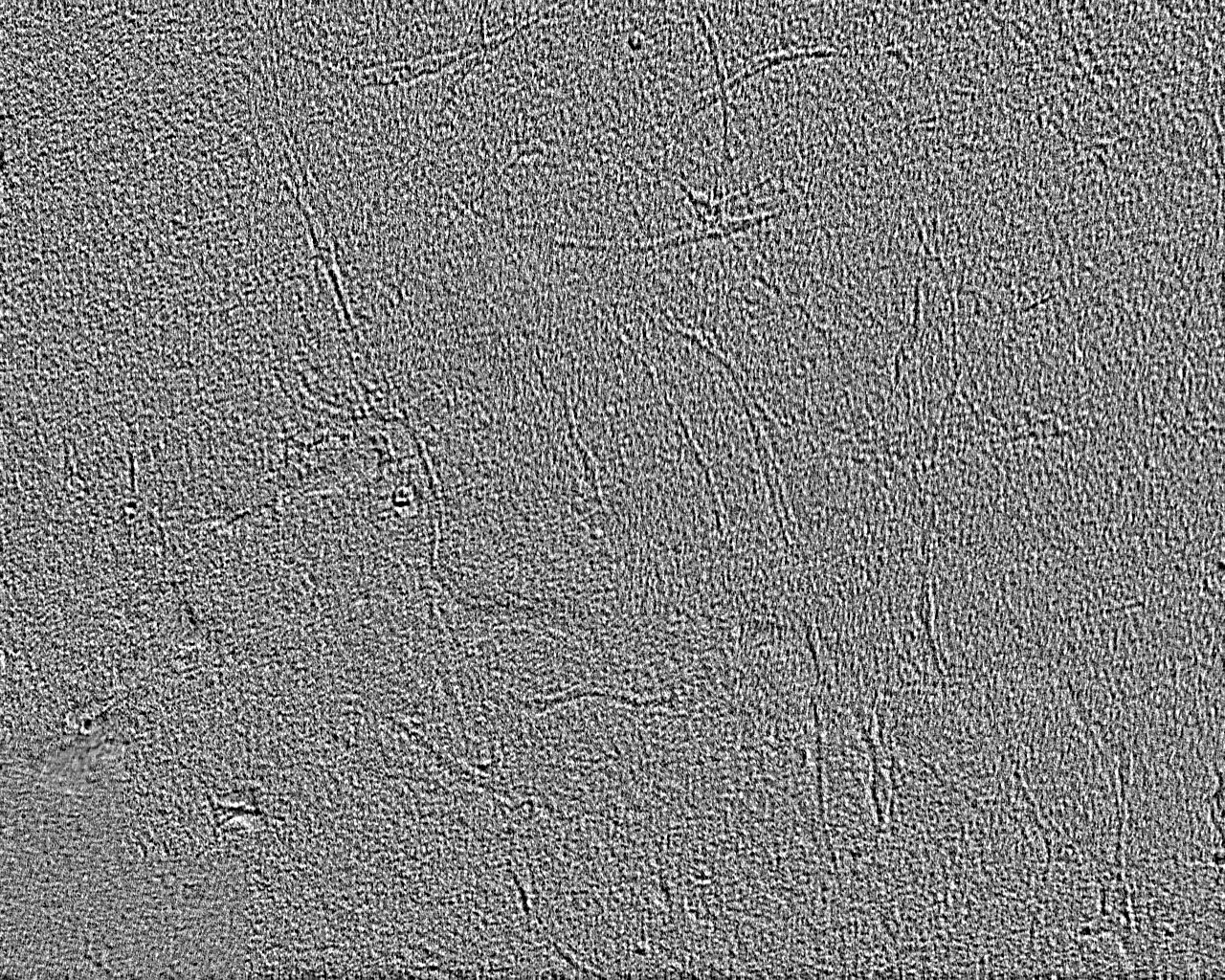

Supplement: Supplementary file 6 — Video microscopy movie 6. Time-lapse microscopy movie (.tif format) showing effects consistent with a bacterial contamination from time point 6 onwards. (ZIP 23,423 kb) [file 12859_2018_2458_MOESM6_ESM.zip › 1_M20_1_2017y06m30d_02h00m.tif]

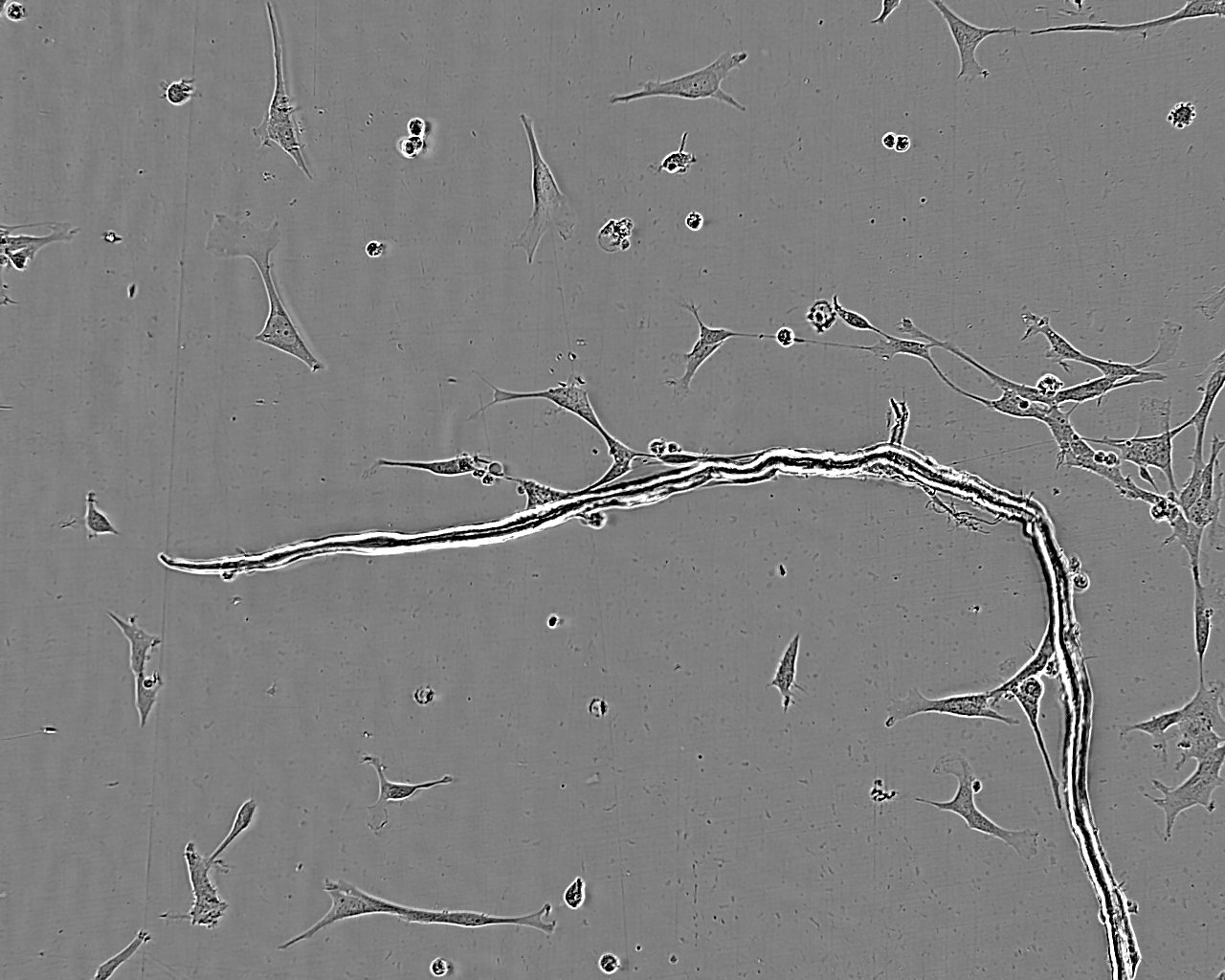

Supplement: Supplementary file 7 — Video microscopy movie 7. Time-lapse microscopy movie (.tif format) detected as an outlier and subsequently removed from all processing steps, during image quality control. (ZIP 18,130 kb) [file 12859_2018_2458_MOESM7_ESM.zip › 1_O21_1_2017y06m29d_18h48m.tif]

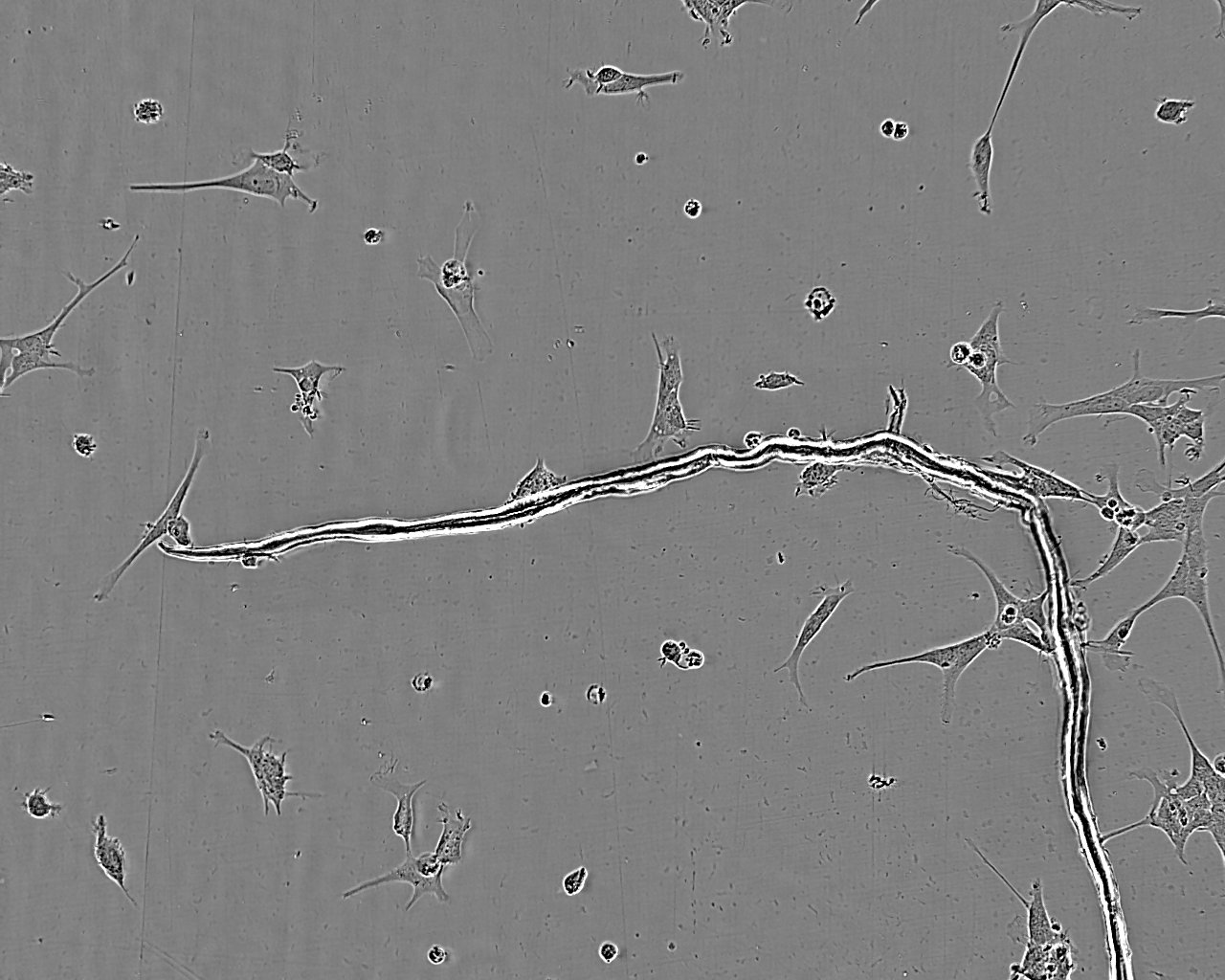

Supplement: Supplementary file 7 — Video microscopy movie 7. Time-lapse microscopy movie (.tif format) detected as an outlier and subsequently removed from all processing steps, during image quality control. (ZIP 18,130 kb) [file 12859_2018_2458_MOESM7_ESM.zip › 1_O21_1_2017y06m30d_00h48m.tif]

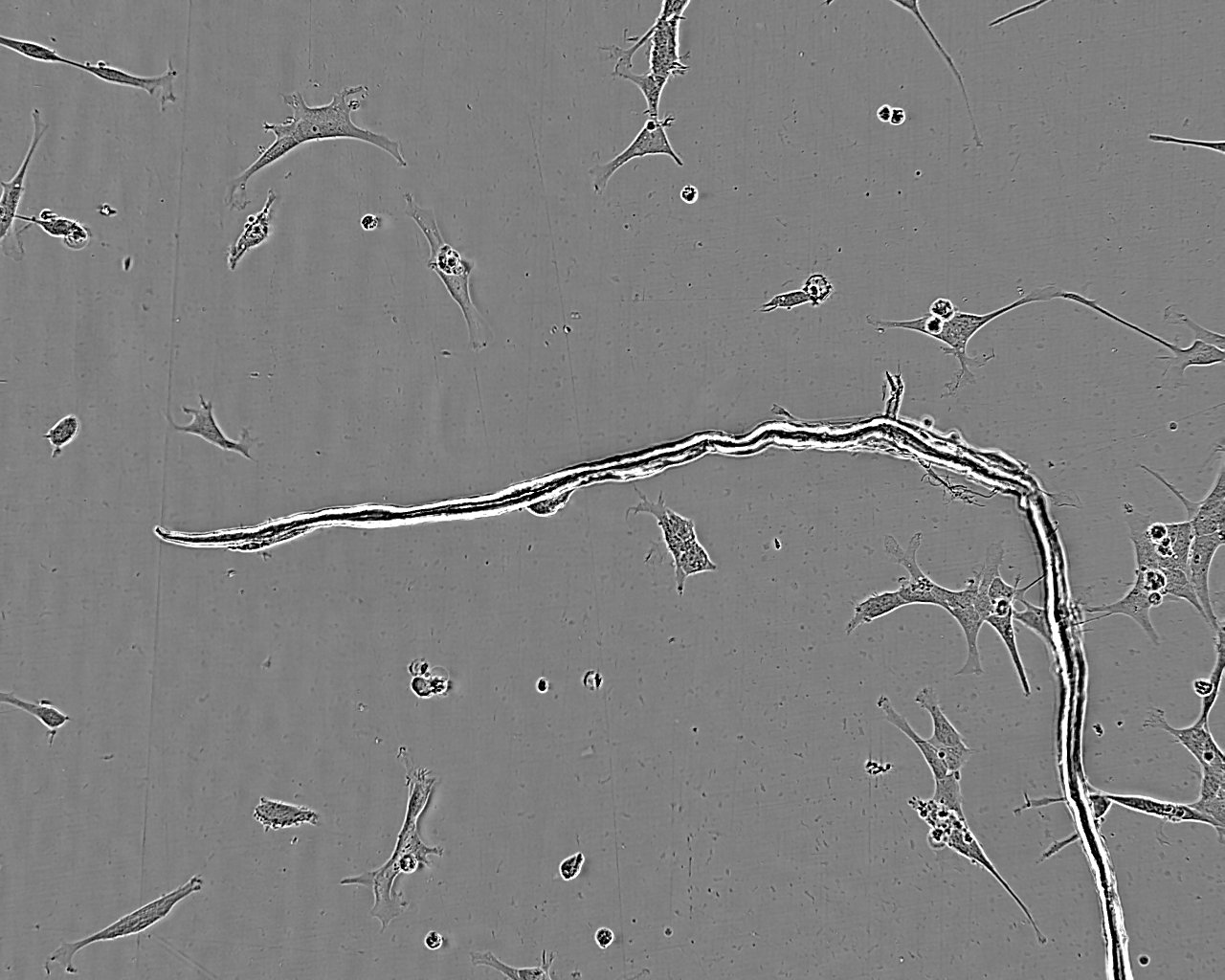

Supplement: Supplementary file 7 — Video microscopy movie 7. Time-lapse microscopy movie (.tif format) detected as an outlier and subsequently removed from all processing steps, during image quality control. (ZIP 18,130 kb) [file 12859_2018_2458_MOESM7_ESM.zip › 1_O21_1_2017y06m30d_05h48m.tif]

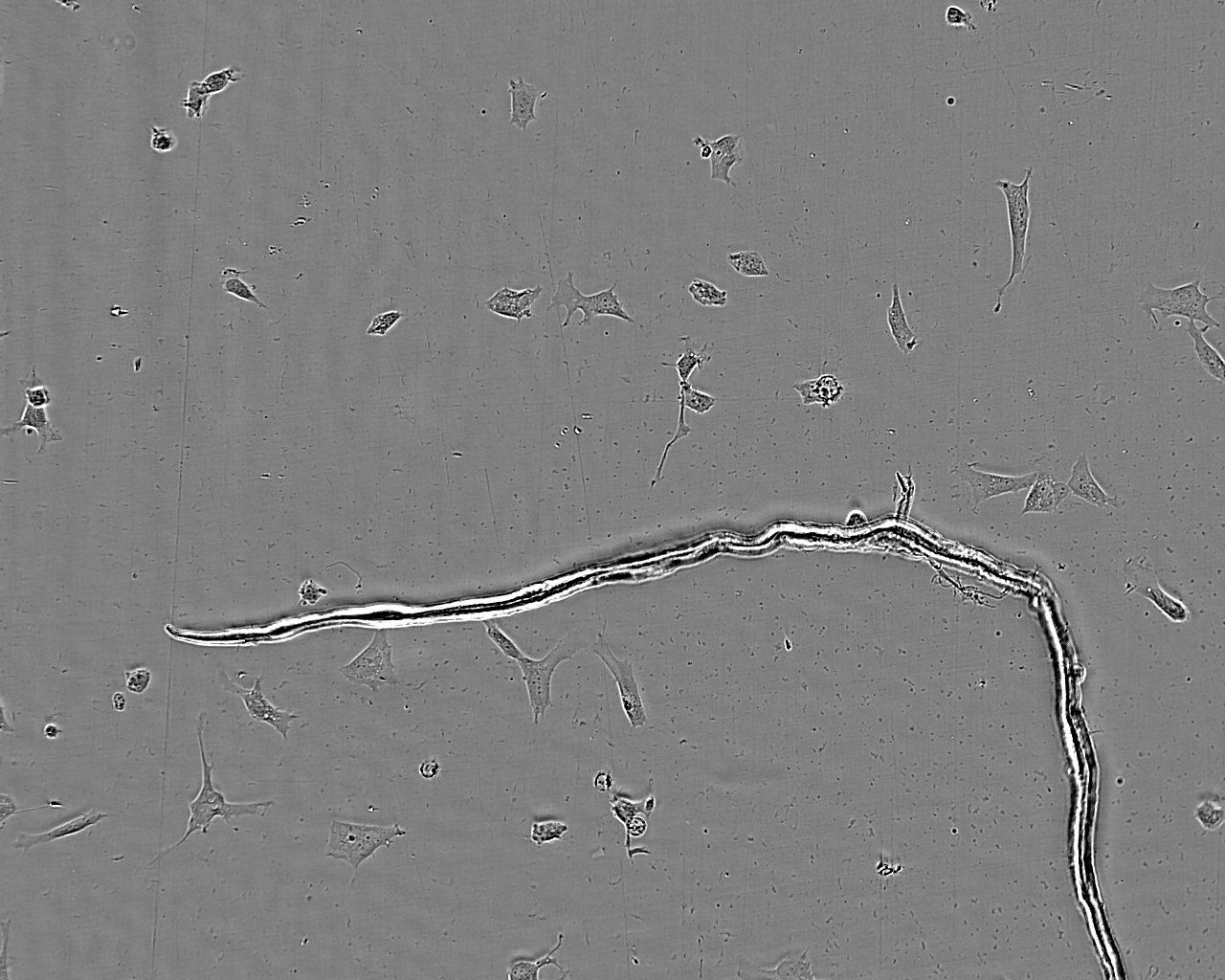

Supplement: Supplementary file 7 — Video microscopy movie 7. Time-lapse microscopy movie (.tif format) detected as an outlier and subsequently removed from all processing steps, during image quality control. (ZIP 18,130 kb) [file 12859_2018_2458_MOESM7_ESM.zip › 1_O21_1_2017y06m26d_18h48m.tif]

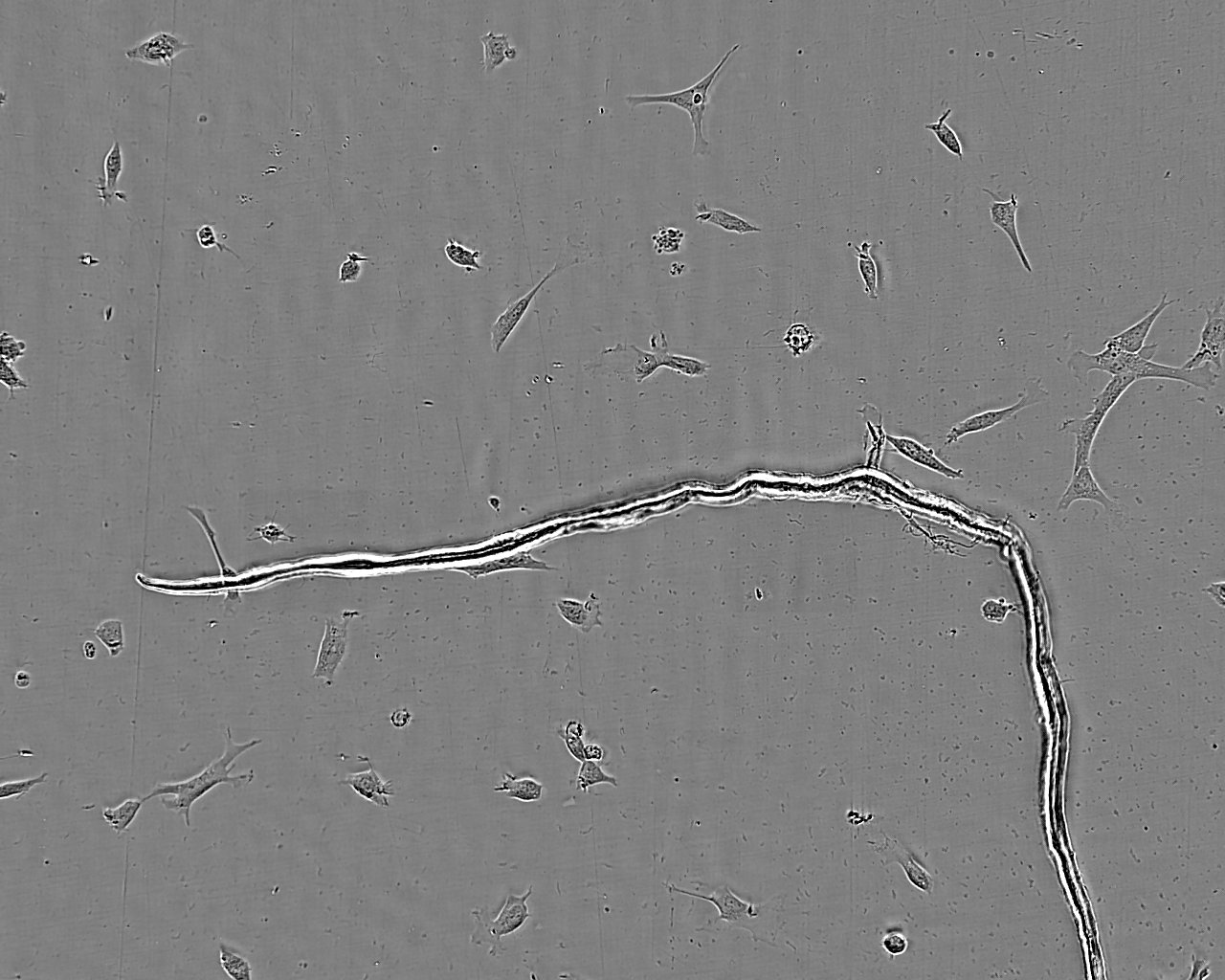

Supplement: Supplementary file 7 — Video microscopy movie 7. Time-lapse microscopy movie (.tif format) detected as an outlier and subsequently removed from all processing steps, during image quality control. (ZIP 18,130 kb) [file 12859_2018_2458_MOESM7_ESM.zip › 1_O21_1_2017y06m27d_00h48m.tif]

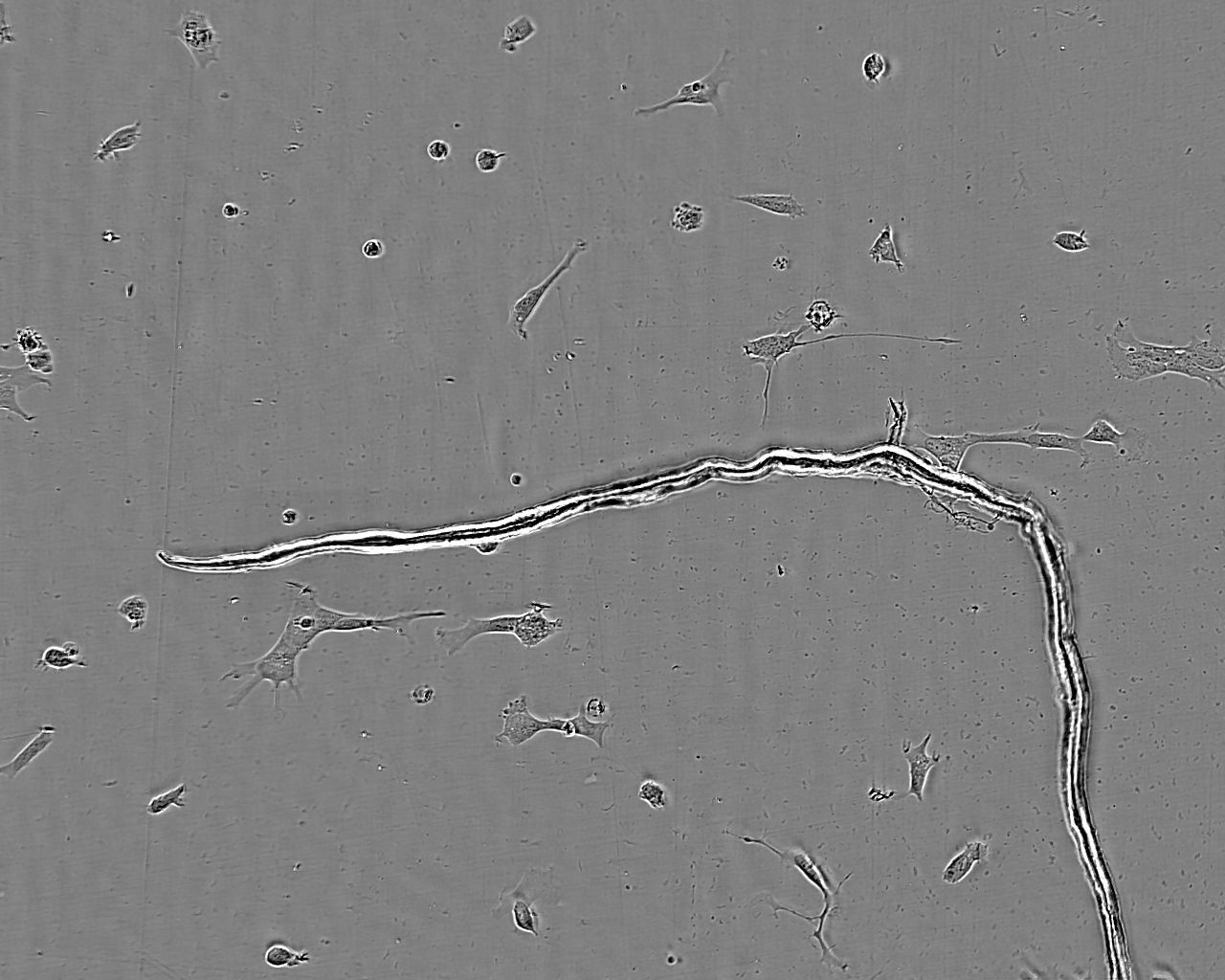

Supplement: Supplementary file 7 — Video microscopy movie 7. Time-lapse microscopy movie (.tif format) detected as an outlier and subsequently removed from all processing steps, during image quality control. (ZIP 18,130 kb) [file 12859_2018_2458_MOESM7_ESM.zip › 1_O21_1_2017y06m27d_06h48m.tif]

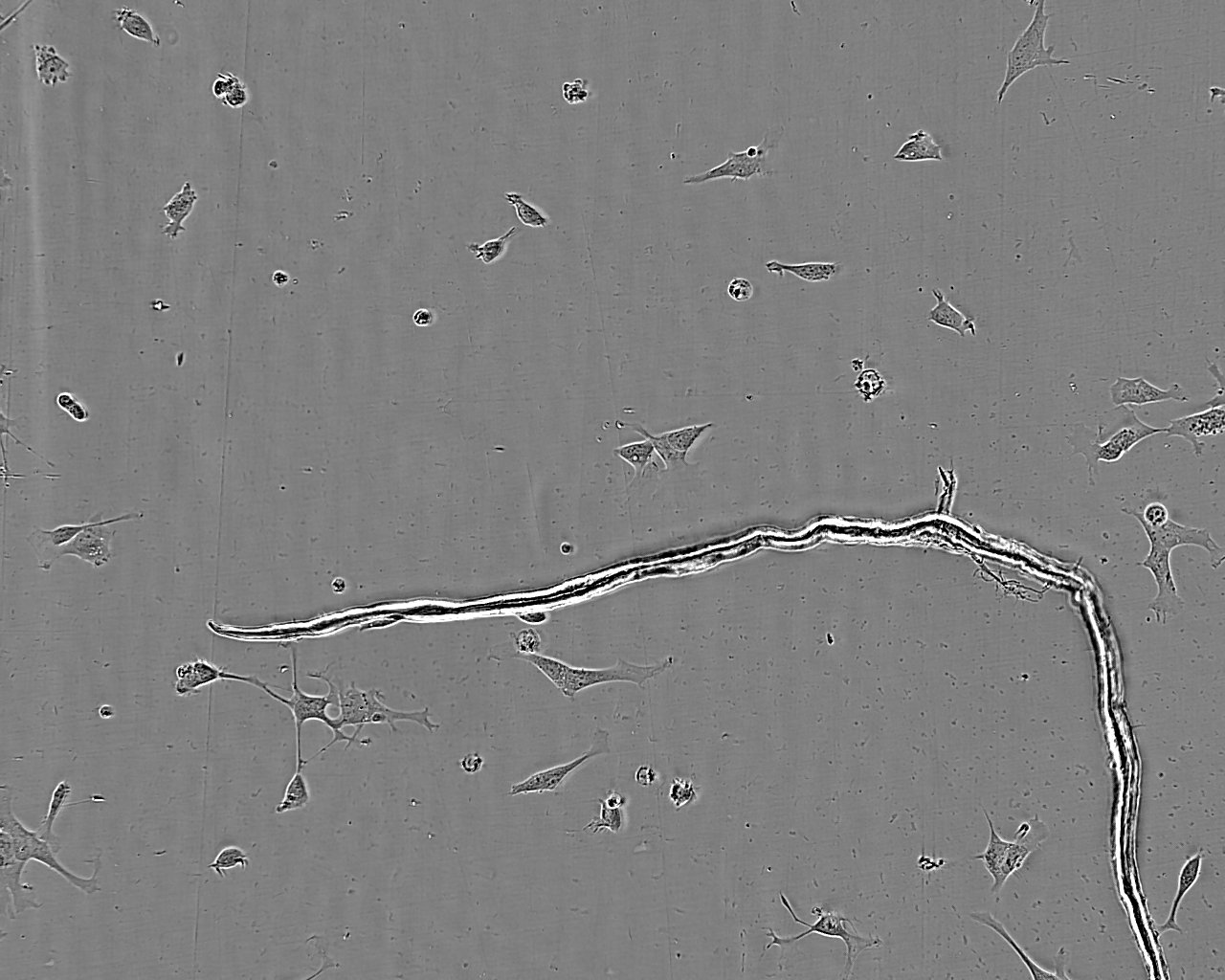

Supplement: Supplementary file 7 — Video microscopy movie 7. Time-lapse microscopy movie (.tif format) detected as an outlier and subsequently removed from all processing steps, during image quality control. (ZIP 18,130 kb) [file 12859_2018_2458_MOESM7_ESM.zip › 1_O21_1_2017y06m27d_12h48m.tif]

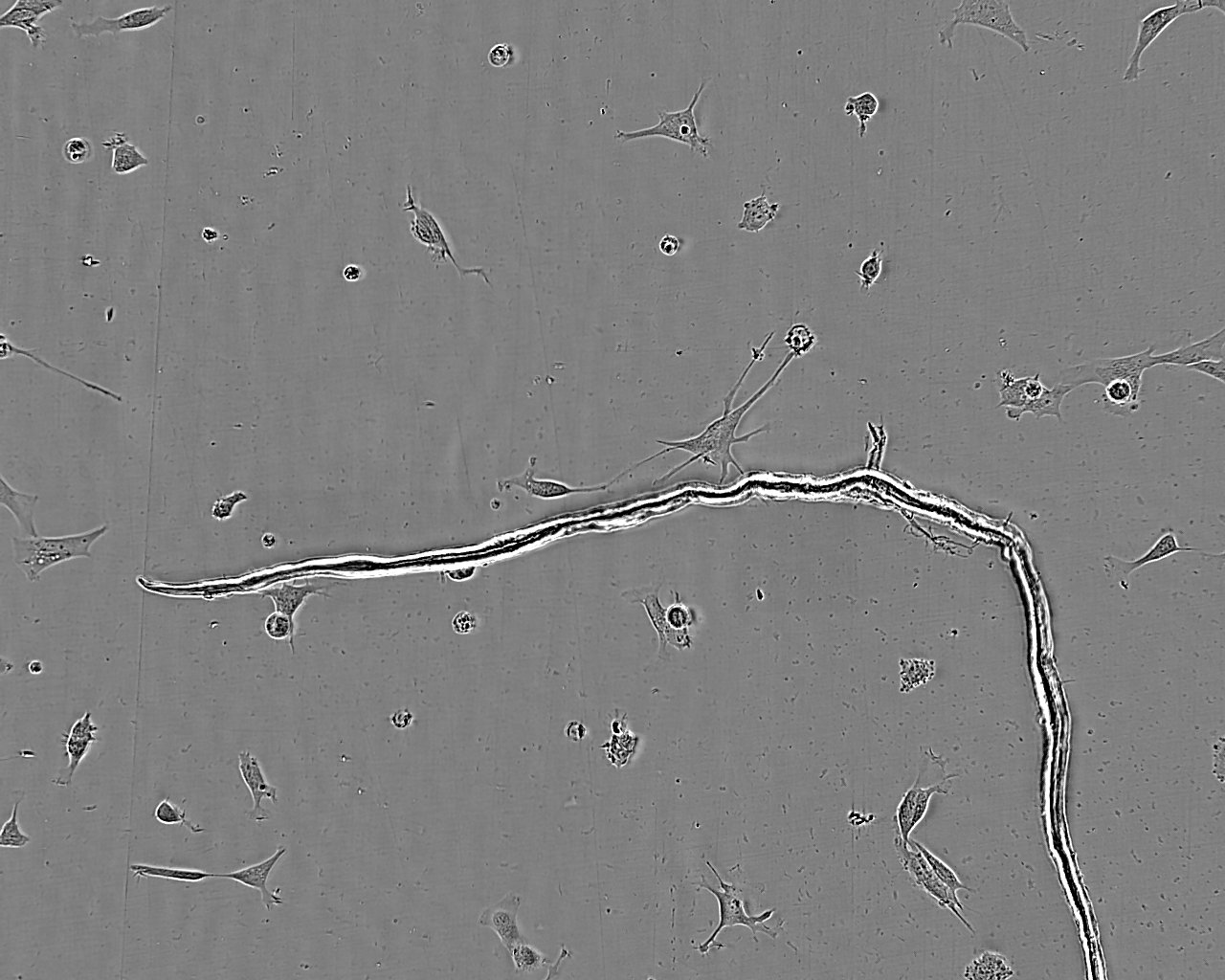

Supplement: Supplementary file 7 — Video microscopy movie 7. Time-lapse microscopy movie (.tif format) detected as an outlier and subsequently removed from all processing steps, during image quality control. (ZIP 18,130 kb) [file 12859_2018_2458_MOESM7_ESM.zip › 1_O21_1_2017y06m27d_18h48m.tif]

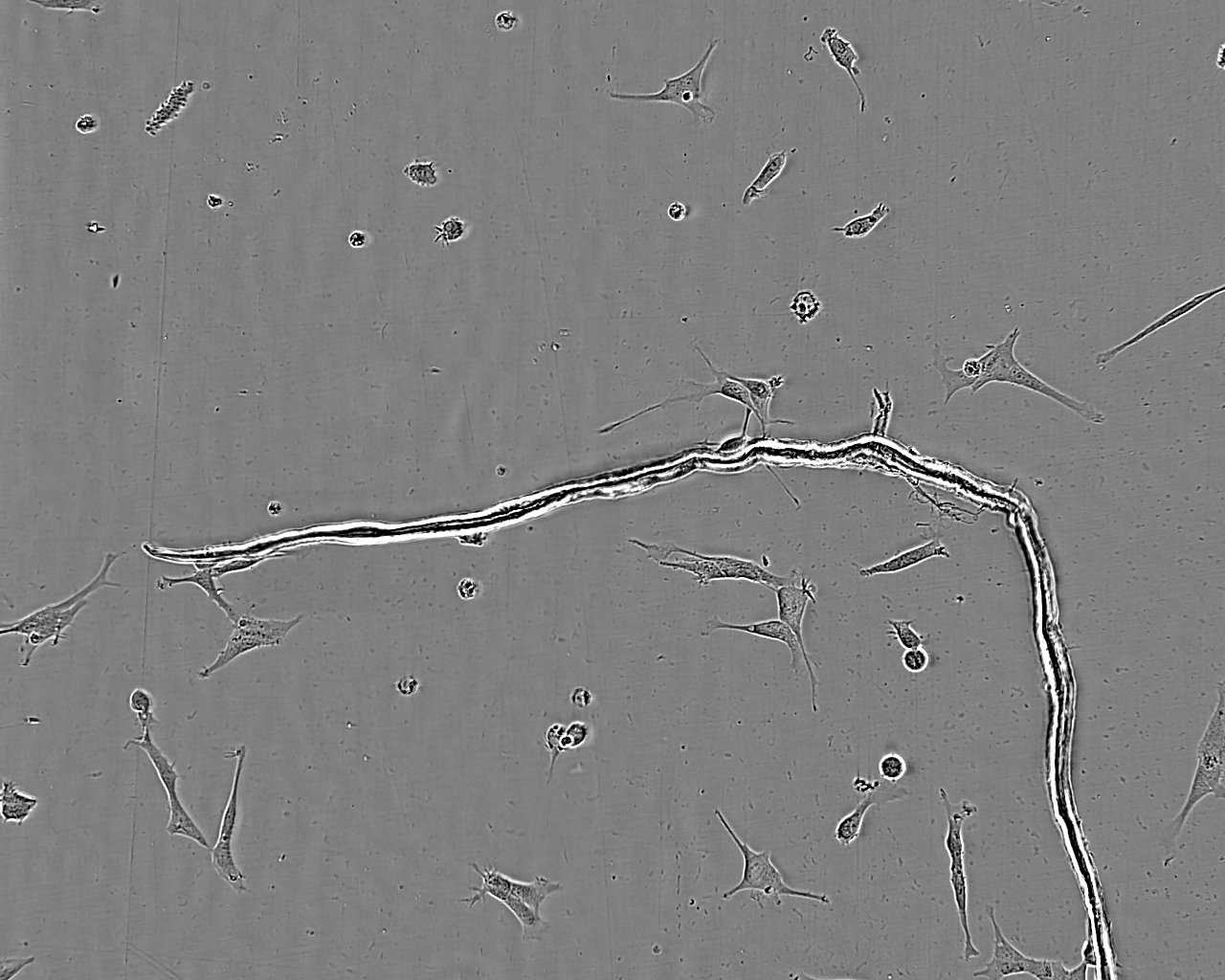

Supplement: Supplementary file 7 — Video microscopy movie 7. Time-lapse microscopy movie (.tif format) detected as an outlier and subsequently removed from all processing steps, during image quality control. (ZIP 18,130 kb) [file 12859_2018_2458_MOESM7_ESM.zip › 1_O21_1_2017y06m28d_00h48m.tif]

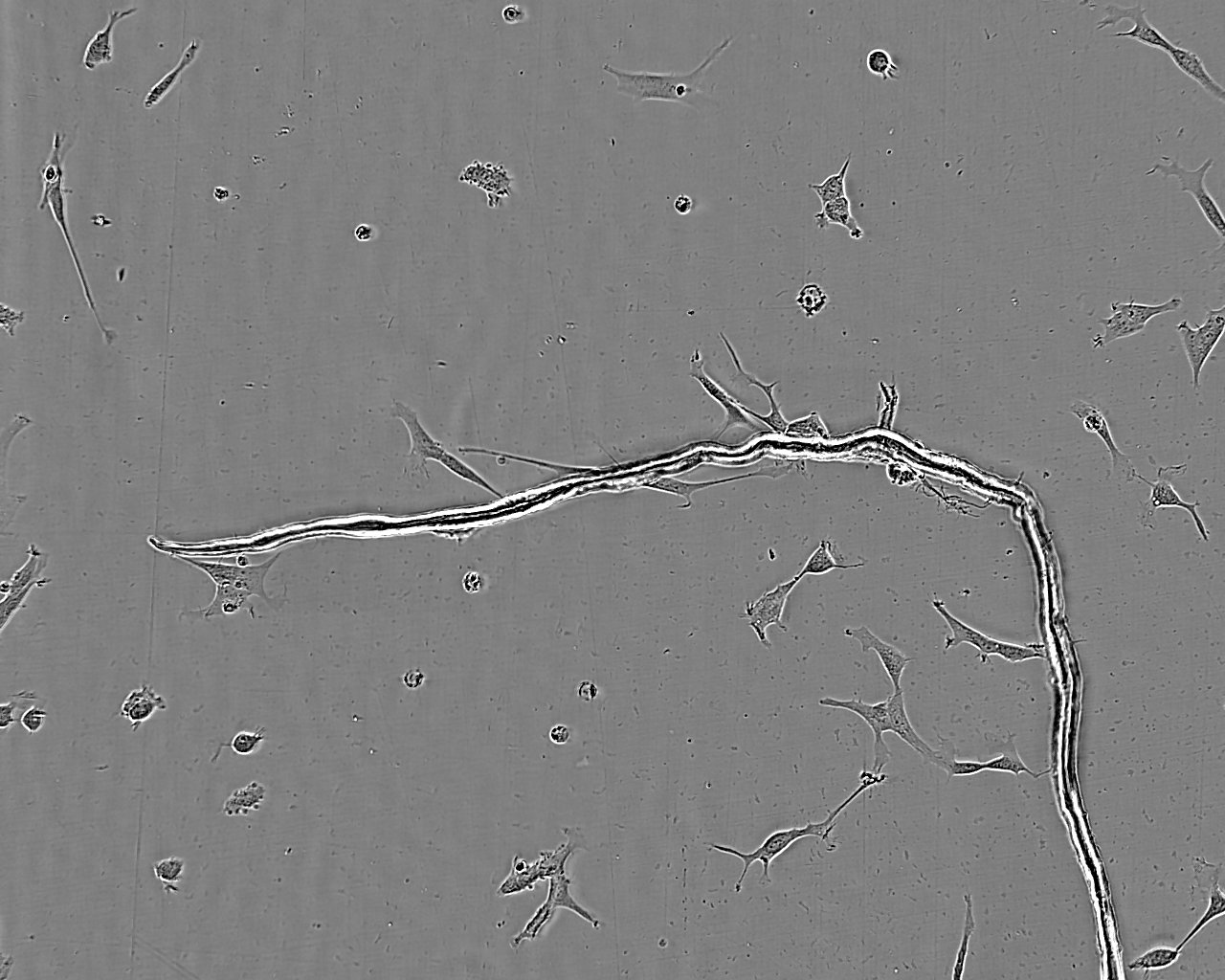

Supplement: Supplementary file 7 — Video microscopy movie 7. Time-lapse microscopy movie (.tif format) detected as an outlier and subsequently removed from all processing steps, during image quality control. (ZIP 18,130 kb) [file 12859_2018_2458_MOESM7_ESM.zip › 1_O21_1_2017y06m28d_06h48m.tif]

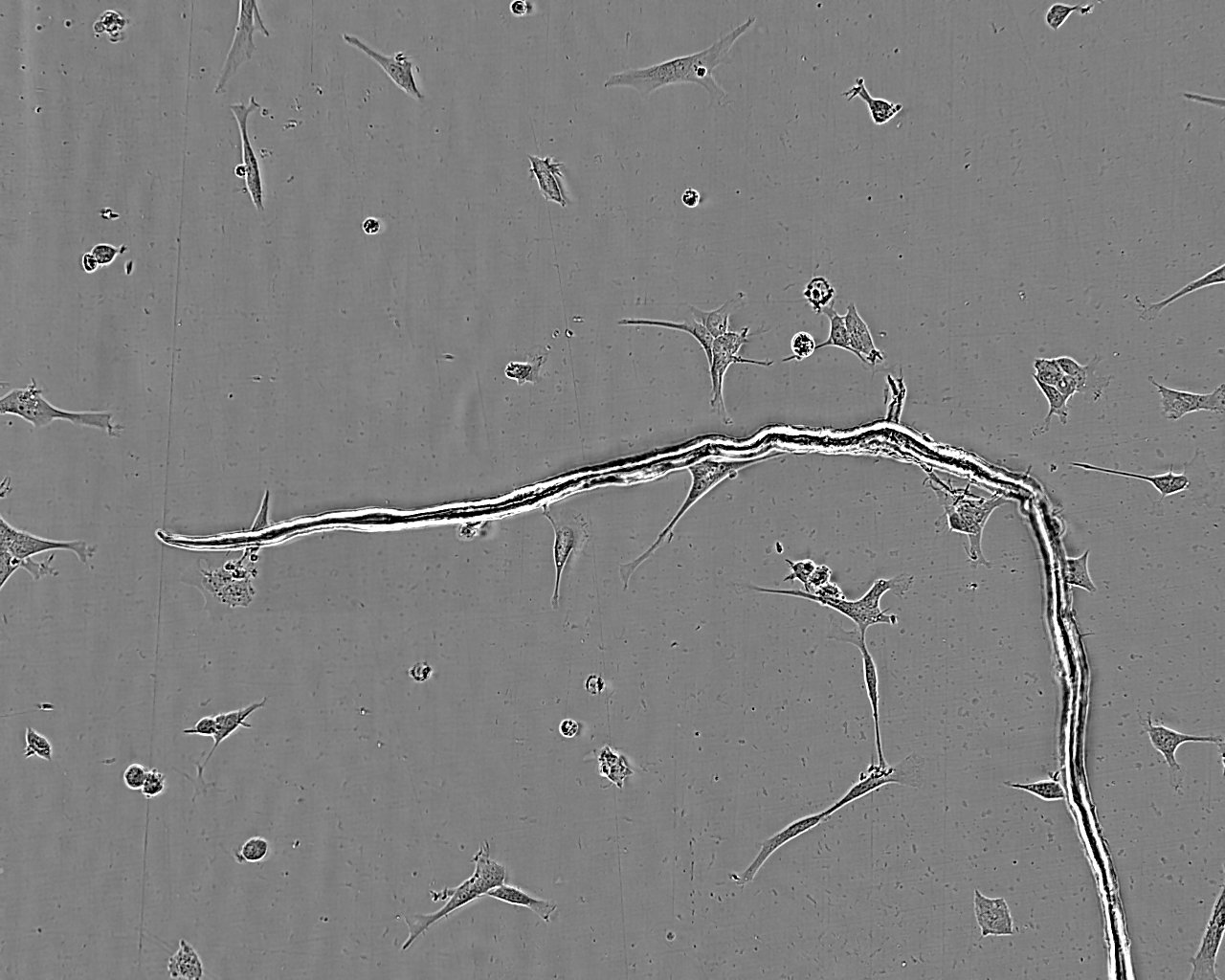

Supplement: Supplementary file 7 — Video microscopy movie 7. Time-lapse microscopy movie (.tif format) detected as an outlier and subsequently removed from all processing steps, during image quality control. (ZIP 18,130 kb) [file 12859_2018_2458_MOESM7_ESM.zip › 1_O21_1_2017y06m28d_12h48m.tif]

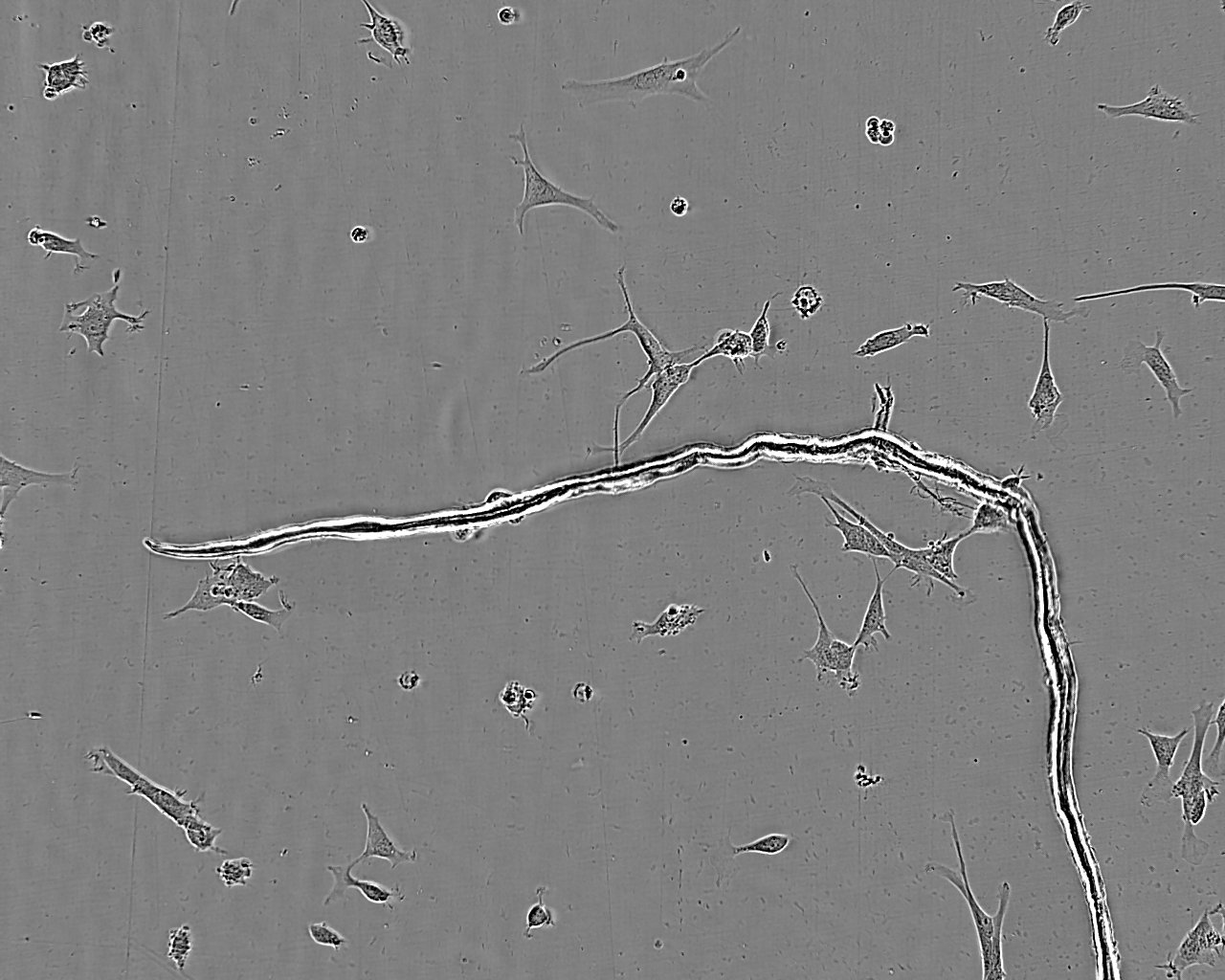

Supplement: Supplementary file 7 — Video microscopy movie 7. Time-lapse microscopy movie (.tif format) detected as an outlier and subsequently removed from all processing steps, during image quality control. (ZIP 18,130 kb) [file 12859_2018_2458_MOESM7_ESM.zip › 1_O21_1_2017y06m28d_18h48m.tif]

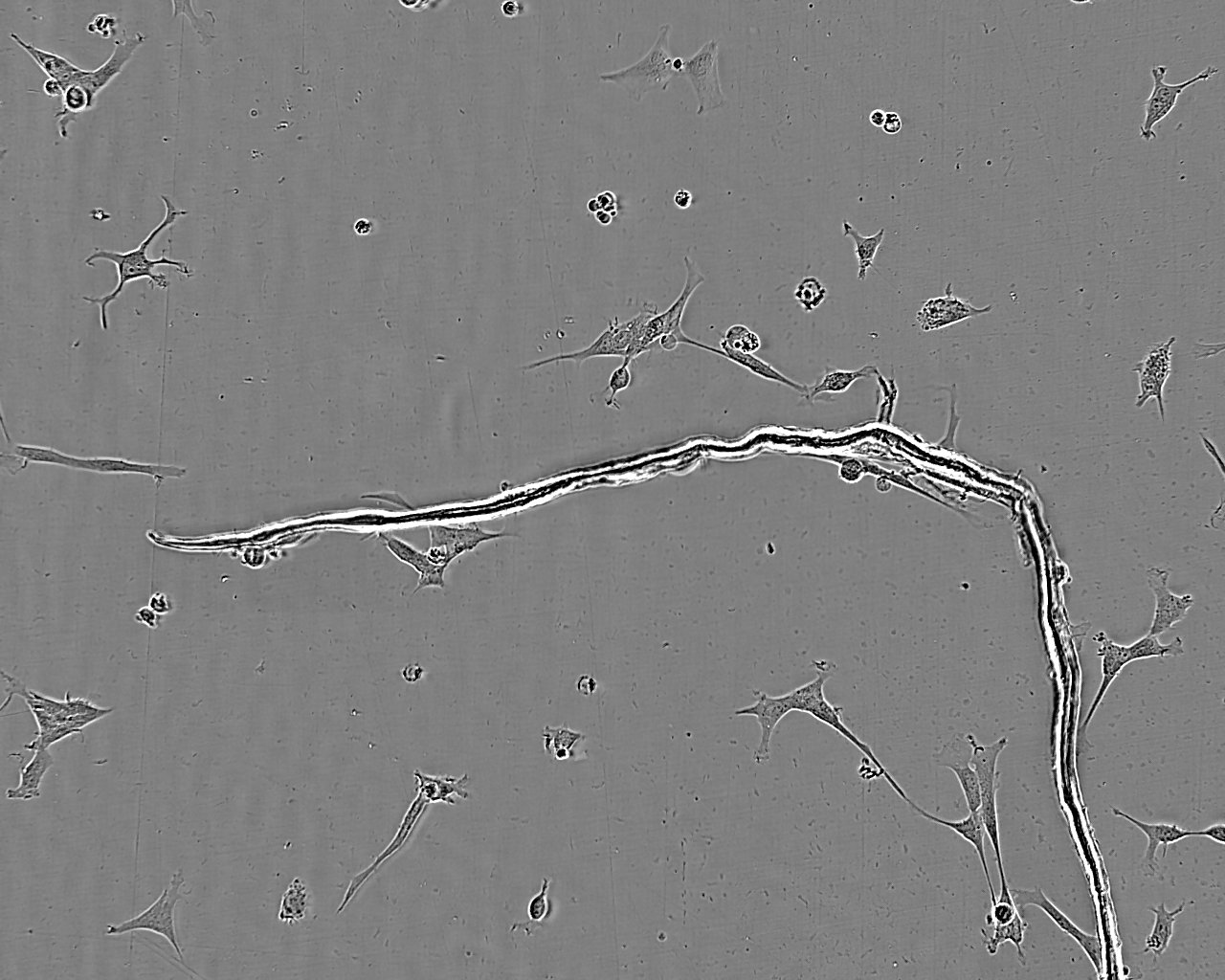

Supplement: Supplementary file 7 — Video microscopy movie 7. Time-lapse microscopy movie (.tif format) detected as an outlier and subsequently removed from all processing steps, during image quality control. (ZIP 18,130 kb) [file 12859_2018_2458_MOESM7_ESM.zip › 1_O21_1_2017y06m29d_00h48m.tif]

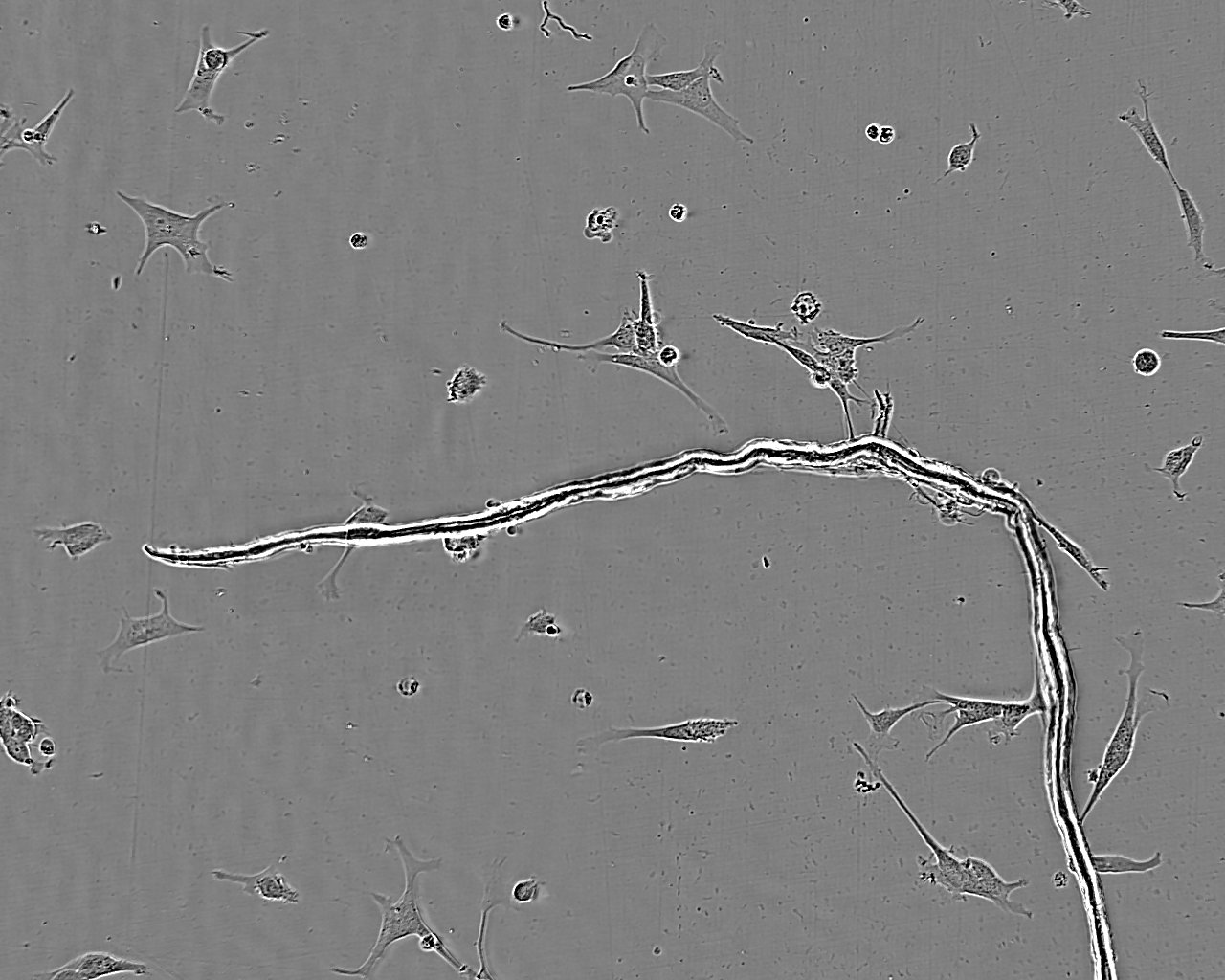

Supplement: Supplementary file 7 — Video microscopy movie 7. Time-lapse microscopy movie (.tif format) detected as an outlier and subsequently removed from all processing steps, during image quality control. (ZIP 18,130 kb) [file 12859_2018_2458_MOESM7_ESM.zip › 1_O21_1_2017y06m29d_06h48m.tif]

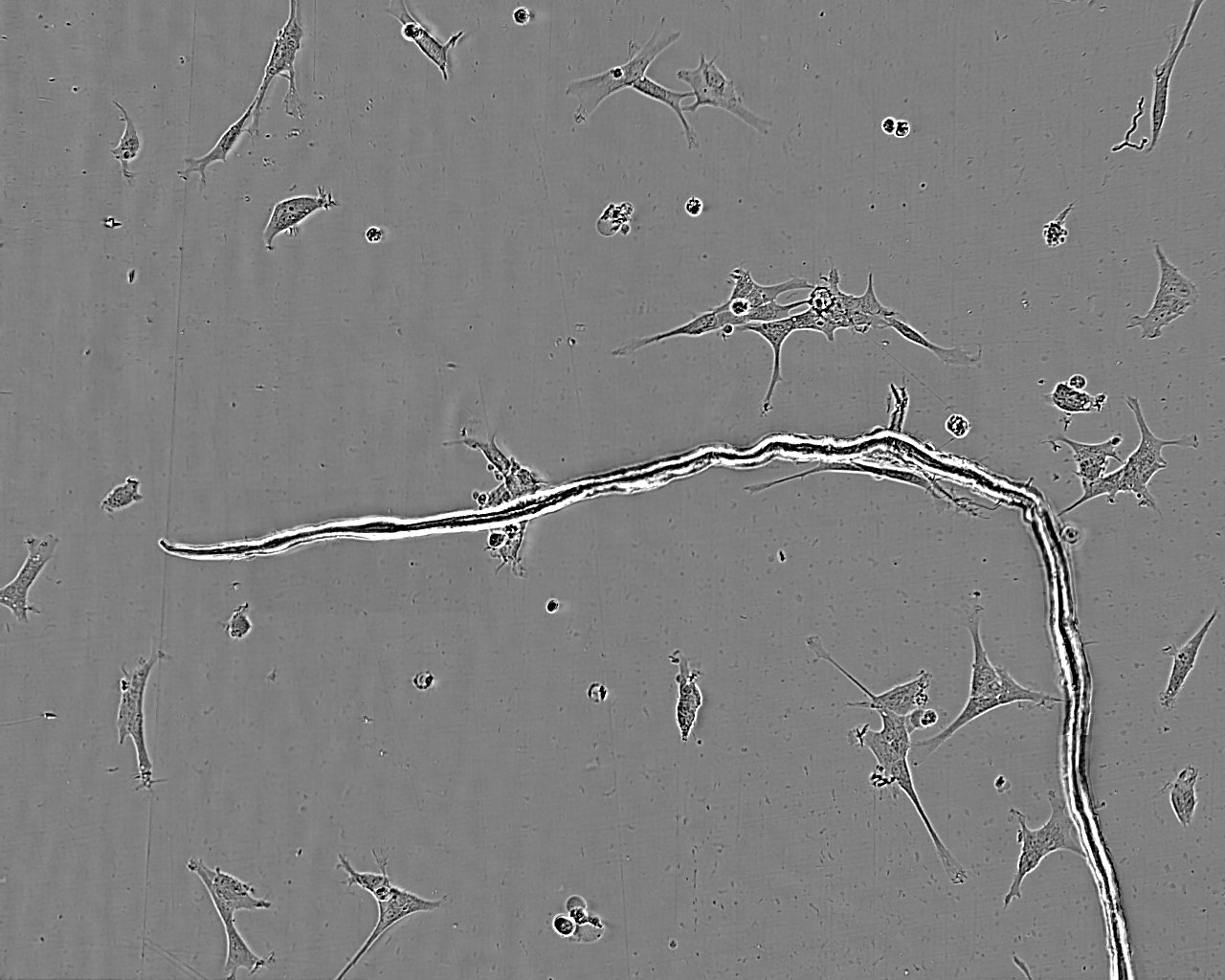

Supplement: Supplementary file 7 — Video microscopy movie 7. Time-lapse microscopy movie (.tif format) detected as an outlier and subsequently removed from all processing steps, during image quality control. (ZIP 18,130 kb) [file 12859_2018_2458_MOESM7_ESM.zip › 1_O21_1_2017y06m29d_12h48m.tif]
