# Supplementary material for: COMBImage: a modular parallel processing framework for pairwise drug combination analysis that quantifies temporal changes in label-free video microscopy movies
Source: BMC Bioinformatics. 2018 Nov 26;19:453. doi: 10.1186/s12859-018-2458-x (PMC6257977; doi:10.1186/s12859-018-2458-x)
Supplement: Supplementary file 8 — Supplementary Information. Single document (.pdf format) with extensive text, additional figures and tables. (ZIP 1466 kb) [file 12859_2018_2458_MOESM8_ESM.pdf]

Supplementary Information

COMBImage: A modular parallel processing framework for pairwise drug combination analysis that quantifies temporal changes in label-free video microscopy movies

Efthymia Chantzi*[1], Malin Jarvius[1,2], Mia Niklasson[3], Anna Segerman[3], and Mats G. Gustafsson[1]

[1]Department of Medical Sciences, Cancer Pharmacology and Computational Medicine, Uppsala University, Uppsala, Sweden
[2]SciLifeLab Drug Discovery and Development, *In Vitro* and Systems Pharmacology Facility, Uppsala University, Uppsala, Sweden
[3]Department of Immunology, Genetics and Pathology, Rudbeck Laboratory, Uppsala University, Uppsala, Sweden

Contents

1 Conventional and Scaled Bliss Synergy Analysis **1**
1.1 Examples 2

2 Conventional and Refined Therapeutic Synergy Analysis **3**
2.1 Examples 3

3 Hierarchical histograms for a particular parameter pair **5**

4 Flowchart Descriptions **5**
4.1 COMBO-V 5
4.2 COMBO-M 6
4.3 COMBO-C 7

5 Supplementary Results **8**
5.1 Cell Viability Analysis & Visualization 8
5.2 Bliss Synergy Analysis and Visualization 11
5.3 Therapeutic Synergy Analysis & Visualization 15
5.4 COMBO-M PHHC Analyses 16

List of Figures

1 COMBO-V (Cell viability analysis for ACS) 8
2 COMBO-V (Cell viability analysis for $U3065 - c271$) 9
3 COMBO-V (Cell viability analysis for $U3065 - c475$) 10
4 COMBO-V (Bliss synergy analysis for ACS) 11

*corresponding author: efthymia.chantzi@medsci.uu.se

5 COMBO-V (Bliss synergy analysis for $U3065 - c271$) 12
6 COMBO-V (Bliss synergy analysis for $U3065 - c475$) 13
7 COMBO-V(Conventional therapeutic synergy analysis) 15
8 COMBO-M PHHC 16
9 COMBO-M Checkerboard Style Screens 17
10 COMBO-C Checkerboard Style Screens 18

List of Tables

1 Pixel histogram hierarchy extraction - Example 5

1 Conventional and Scaled Bliss Synergy Analysis

The framework provides assessment of the pairwise combinatorial effects by calculating their deviation from the Bliss independence model, which is based on the assumption that the combined drugs act independently of each other. This results in the conventional Bliss index, calculated for a particular combination concentration (c_1, c_2), as:

$$B(c_1, c_2) = S_1(c_1) \cdot S_2(c_2) - S_{12}(c_1, c_2) \tag{1}$$

Here, $S_1(c_1) \cdot S_2(c_2)$ denotes the predicted effect according to the Bliss independence model, while $S_{12}(c_1, c_2)$ denotes the actual/observed effect of the combination treatment. The pipeline also provides a novel scaled version of the Bliss index, here denoted B_S and defined as:

$$\begin{aligned} B_S(c_1, c_2) &= B(c_1, c_2) \cdot [1 - min\{S_1(c_1) \cdot S_2(c_2), S_{12}(c_1, c_2)\}] \\ &= \begin{cases} B(c_1, c_2) \cdot [1 - S_{12}(c_1, c_2)] & \text{if } B(c_1, c_2) > 0 \\ 0 & \text{if } B(c_1, c_2) = 0 \\ B(c_1, c_2) \cdot [1 - S_1(c_1) \cdot S_2(c_2)] & \text{if } B(c_1, c_2) < 0 \end{cases} \end{aligned} \tag{2}$$

Here, $min\{S_1(c_1) \cdot S_2(c_2), S_{12}(c_1, c_2)\}$ denotes the minimum value among the predicted and actual survival values, $S_1(c_1) \cdot S_2(c_2)$ and $S_{12}(c_1, c_2)$, respectively. Thus, in the case of synergy, B_S suppresses cases associated with high survival values for the combination evaluated, while in the case of antagonism it suppresses cases associated with high survival values for the single drugs of the combination studied. B_S is introduced as a simple way to rank the drug combinations in a practically more interesting way. This is because the conventional Bliss index B may be relatively large for cases where the combination studied does not cause outstanding effects.

For drugs causing growth inhibition and/or cell killing, the range of $B_S(c_1, c_2)$ spans from -1 to 1 indicating maximal antagonism and synergy, respectively. More precisely:

- If $B_S(c_1, c_2) > 0$, then the combination effect is synergistic, since $B(c_1, c_2) > 0$ and thus, $S_{12}(c_1, c_2) < S_1(c_1) \cdot S_2(c_2)$. Notably, the maximum value $B_S(c_1, c_2) = 1$ is reached, when $B(c_1, c_2) = 1$ and consequently, $S_1(c_1) = S_2(c_2) = 1$, $S_{12}(c_1, c_2) = 0$.
- If $B_S(c_1, c_2) = 0$, then the Bliss independence assumption holds, since $B(c_1, c_2) = 0$ and thus, $S_{12}(c_1, c_2) = S_1(c_1) \cdot S_2(c_2)$, meaning that the drugs act independently of each other.
- If $B_S(c_1, c_2) < 0$, then the combination effect is antagonistic, since $B(c_1, c_2) < 0$ and thus, $S_{12}(c_1, c_2) > S_1(c_1) \cdot S_2(c_2)$. Notably, the minimum value $B_S(c_1, c_2) = -1$ is reached, when $B(c_1, c_2) = -1$ and consequently, $S_1(c_1) = S_2(c_2) = 0$, $S_{12}(c_1, c_2) = -1$.

Concrete examples showing the benefits of employing B_S instead of B, when ranking experimental observations from CA studies, are following.

1.1 Examples

Here, we provide concrete examples that illustrate the importance of adopting the scaled Bliss index B_S instead of the plain Bliss index B.

Example 1 - Elucidating Bliss Synergy

Let us assume that we have the following two cases:

(a) $S_1(c_1) \cdot S_2(c_2) = 1$, $S_{12}(c_1, c_2) = 0.7$

(b) $S_1(c_1) \cdot S_2(c_2) = 0.7$, $S_{12}(c_1, c_2) = 0.4$

In both cases, there is identical Bliss synergy, as indicated by the Bliss index B:

(a) $B(c_1, c_2) = 1 - 0.7 = 0.3$

(b) $B(c_1, c_2) = 0.7 - 0.4 = 0.3$

However, the Bliss synergy seems to be elucidated with respect to the actual effect, when calculating the scaled Bliss index B_S:

(a) $B_S(c_1, c_2) = 0.3 \cdot (1 - 0.7) = 0.3 \cdot 0.3 = 0.09$

(b) $B_S(c_1, c_2) = 0.3 \cdot (1 - 0.4) = 0.3 \cdot 0.6 = 0.18$

Example 2 - Elucidating Bliss Antagonism

Let us assume that we have the following two cases:

(a) $S_1(c_1) \cdot S_2(c_2) = 0.04$, $S_{12}(c_1, c_2) = 0.5$

(b) $S_1(c_1) \cdot S_2(c_2) = 0.34$, $S_{12}(c_1, c_2) = 0.8$

In both cases, there is identical Bliss antagonism, as indicated by the Bliss index B:

(a) $B(c_1, c_2) = 0.04 - 0.5 = -0.46$

(b) $B(c_1, c_2) = 0.34 - 0.8 = -0.46$

However, the Bliss antagonism seems to be elucidated with respect to the predicted effect, when calculating the scaled Bliss index B_S:

(a) $B_S(c_1, c_2) = -0.46 \cdot (1 - 0.04) = -0.46 \cdot 0.96 = -0.44$

(b) $B_S(c_1, c_2) = -0.46 \cdot (1 - 0.34) = -0.46 \cdot 0.66 = -0.3$

Given the aforementioned examples and following the discussion above, it is obvious that the scaled Bliss index B_S may be very useful, as it incorporates the plain and conventional Bliss index B with a sorting/filtering perspective, which may be very helpful and even crucial, especially in large-scale experiments, where plenty of drug pairs in large concentration grids are evaluated. It should not be perceived as a replacement of the conventional Bliss index, but rather as a powerful complement, which not only does it inherit all the established properties, but it also enables ranking/sorting of the results.

2 Conventional and Refined Therapeutic Synergy Analysis

The framework also includes a refined form of the therapeutic index:

$$T(c_1, c_2) = S_r(c_1, c_2) - S_t(c_1, c_2) \tag{3}$$

where $S_r(c_1, c_2)$ and $S_t(c_1, c_2)$ denote the survival index values for the reference and target cell models, respectively. The new refined index is defined as:

$$\begin{aligned} T_{RW}(c_1, c_2) &= T(c_1, c_2) \cdot max\{S_r(c_1, c_2), S_t(c_1, c_2)\} \\ &= \begin{cases} T(c_1, c_2) \cdot S_r(c_1, c_2) & \text{if } T(c_1, c_2) > 0 \\ 0 & \text{if } T(c_1, c_2) = 0 \\ T(c_1, c_2) \cdot S_t(c_1, c_2) & \text{if } T(c_1, c_2) < 0. \end{cases} \end{aligned} \tag{4}$$

Here $max\{S_r(c_1, c_2), S_t(c_1, c_2)\}$ denotes the maximum value among the two survival index values $S_r(c_1, c_2)$ and $S_t(c_1, c_2)$. T_{RW} is referred to as the reference weighted therapeutic index because it is designed to suppress synergistic cases associated with considerable adverse effects (AEs) in the reference cell model and antagonistic cases, where there are not considerable effects on the target cells. The range of T_{RW} ranges from -1 to 1 indicating maximal therapeutic antagonism and synergy, respectively. More specifically:

- If $T_{RW}(c_1, c_2) > 0$, then the combination effect shows therapeutic synergy, since $T(c_1, c_2) > 0$ and thus, $S_r(c_1, c_2) > S_t(c_1, c_2)$. Notably, if $T_{RW}(c_1, c_2) = 1$, then there is maximal therapeutic synergy, since $T(c_1, c_2) = 1$ and thus, $S_r(t_1, t_2) = 1$, $S_t(c_1, c_2) = 0$. This corresponds to the right endpoint of the aforementioned interval $[-1, 1]$, indicating the ideal case, where all reference cells have survived, whereas all target cells have been killed.
- If $T_{RW}(c_1, c_2) = 0$, then the combination effect does not show any therapeutic window, since $S_r(c_1, c_2) = S_t(c_1, c_2)$. This corresponds to the middle value of the aforementioned interval $[-1, 1]$, which indicates the neutral case.
- If $T_{RW}(c_1, c_2) < 0$, then the combination effect shows therapeutic antagonism, since $T(c_1, c_2) < 0$ and thus, $S_r(c_1, c_2) < S_t(c_1, c_2)$. Notably, if $T_{RW}(c_1, c_2) = -1$, then there is maximal therapeutic antagonism, since $T(c_1, c_2) = -1$ and thus, $S_r(c_1, c_2) = 0$, $S_t(c_1, c_2) = 1$. This corresponds to the left endpoint of the aforementioned interval $[-1, 1]$, indicating the worst case, where all target cells have survived, whereas all reference cells have been killed.

Concrete examples showing the benefits of employing T_{RW} instead of T, when ranking experimental observations from CA studies, are following.

2.1 Examples

Here, we provide concrete examples that illustrate the importance of adopting the reference weighted therapeutic index T_{RW} instead of the plain therapeutic index T.

Example 1 - Elucidating Therapeutic Synergy

Let us assume that we have the following two cases:

(a) $S_r(c_1) = 1$, $S_t(c_1, c_2) = 0.7$

(b) $S_r(c_1) = 0.3$, $S_t(c_1, c_2) = 0$

In both cases, there is identical therapeutic synergy, according to the therapeutic index T:

(a) $T(c_1, c_2) = 1 - 0.7 = 0.3$

(b) $T(c_1, c_2) = 0.3 - 0 = 0.3$

However, the therapeutic synergy seems to be elucidated with respect to the effect on the reference cells, only when calculating the reference weighted therapeutic index T_{RW}:

(a) $T_{RW}(c_1, c_2) = 0.3 \cdot 1 = 0.3$

(b) $T_{RW}(c_1, c_2) = 0.3 \cdot 0.3 = 0.09$

Thus, the case when only 30% of the reference cells survive results in a much lower weighted index compared to when the reference cells are unaffected.

Example 2 - Elucidating Therapeutic Antagonism

Let us assume that we have the following two cases:

(a) $S_r(c_1) = 0.7$, $S_t(c_1, c_2) = 1$

(b) $S_r(c_1) = 0$, $S_t(c_1, c_2) = 0.3$

In both cases, there is identical therapeutic antagonism, according to the therapeutic index T:

(a) $T(c_1, c_2) = 0.7 - 1 = -0.3$

(b) $T(c_1, c_2) = 0 - 0.3 = -0.3$

However, the therapeutic antagonism seems to be elucidated with respect to the effect on the target cells, only when calculating the reference weighted therapeutic index T_{RW}:

(a) $T_{RW}(c_1, c_2) = -0.3 \cdot 1 = -0.3$

(b) $T_{RW}(c_1, c_2) = -0.3 \cdot 0.3 = -0.09$

Thus, the case when only 30% of the target cells survive results in a much smaller weighted index compared to when all target cells survive. In other words, there is greater weighted antagonism when the target cells survive.

Given the aforementioned examples and following the discussion above, it is obvious that the reference weighted therapeutic index T_{RW} may be very useful, as it incorporates the

plain and conventional therapeutic index T with a sorting/filtering perspective, which is crucial, especially in large-scale experiments, where plenty of drug pairs in large concentration grids are evaluated. It should not be perceived as a replacement of the conventional therapeutic index, but rather as a powerful complement, which not only does it inherit all the established properties, but it also enables ranking/sorting of the results.

3 Hierarchical histograms for a particular parameter pair

The original dimensions of an image were 1024×1280, while the corresponding number of bins was set to 128, meaning 2 intensity levels per bin (as the images were recorded using 256 grayscale levels). Notably, this number (i.e. number of bins for the original resolution) is user-defined during the initiation of the framework. Table ST1 demonstrates how the pixel histogram hierarchy extraction is performed by using the parameter pair $(r, b) = (\frac{1}{4}, \frac{1}{8})$. This means that the resolution and the corresponding number of bins will be 4 and 8 times smaller at each hierarchical level, respectively:

	**Hierarchical Levels**		
	#1	#2	#3
**resolution**	1024×1280	256×320	64×80
**bins**	128	16	2

Table ST1: Resolution and number of bins for the 3 hierarchical levels of the parameter pair $(r, b) = (\frac{1}{4}, \frac{1}{8})$.

4 Flowchart Descriptions

4.1 COMBO-V

The flowchart of COMBO-V is described in detail below by referring to all individual modules as numbered in the main figure (fig. 2):

(1) The user selects interactively (i) microplate reader file (compatibility with FLUOstar® Omega and CLARIOstar®) and (ii) .xlsx specification file, which contains information about the cell models, plate barcodes, drugs and corresponding concentrations.

(2) The toolbox is built on custom in-house 384-well experimental layouts that allow 15, 20 or 21 and 4 or 8 drug pairs in 4×4 and 6×6 concentration grids, respectively (see table 2, main text). In this step, the microplate reader file is parsed and the raw fluorescence values are automatically assigned to the custom plate layout.

(3) FMCA-based cell viability analysis is performed for all plates included in the specification file from step (1).

(4) Conventional and scaled Bliss synergy analysis for all plates included in the specification file from step (1).

(5) Conventional and refined therapeutic synergy analysis for all plates included in the specification file from step (1). This option is not set by default, since it pre-requires an identical experimental setup for the reference cells. In such cases, the user, when asked, has to enter separately the plate barcodes with target and reference cells.

(6) Results from steps (3), (4) and/or (5) in a checkerboard format, as heatmaps in EPS file format under \Results, a directory which is automatically created inside the folder that contains the executable file for COMBO-V.

(7) Extraction of all results from steps (3) and (4) in CSV file format under \Results, a directory which is automatically created inside the folder that contains the executable file for COMBO-V.

Exact instructions for running COMBO-V are provided as a readme text file, inside the folder that contains the executable file. The execution of COMBO-V is fully automated with appropriate user menus, console printouts and progress bars.

4.2 COMBO-M

The flowchart of COMBO-M is described in detail below by referring to all individual modules as numbered in the main figure (fig. 4):

(1) The user selects interactively the target directory, where the images are stored. It can be either a local or external (e.g., external hard drive) directory. A backup directory (\BU) is automatically created under the target directory, where all images are copied. All the results are saved under \PHHC, an automatically created directory under the target directory.

(2) Image quality control. The user has to insert the number of untreated frames per movie, which are automatically transferred into \untreated, where they are separately processed. When the quality control is completed, the detected outliers are removed from the target directory. The detected outliers are saved in TXT and EPS file format under \BU. Foreground segmentation is also executed at this step and the necessary results for the main analysis are saved in a text file named foreground_segmentation.txt under \BU.

(3) The TEM for all experimental wells are extracted, using MapReduce, as hierarchical pixel histograms only on the foreground, for a particular pair (r, b) of the employed 2×2 parameter grid and all decreasing time intervals. The first time interval includes all available time points, the second one all but the first and so on, until only the last time point.

(4) The DTEM for untreated and treated experimental wells are quantified.

(5) The calculated DTEM are ranked with respect to the user-defined i^{th} percentile ("null" threshold) of the "null" distribution.

(6) The results are saved in TXT file format under \PHHC. There are two different text files; (i) only values of the morphologically interesting wells in decreasing order and (ii) values of all experimental wells in decreasing order.

(7) The user selects interactively the .xlsx specification file of the experiment, which contains information about the cell models, barcodes, drugs and corresponding concentrations.

(8) The toolbox is built on custom in-house 384-well experimental layouts that allow 15, 20 or 21 and 4 or 8 drug pairs in 4×4 and 6×6 non-zero concentration grids, respectively (see table 2, main text). In this step, the relative differences $\widetilde{d}$ from the ith user-defined "null" threshold are automatically assigned to the plate layout.

(9) Results in checkerboard format, as heatmaps in EPS file format under \PHHC inside the target directory from step (1). In particular, two different EPS files are generated per experimental plate; (i) actual values depicted in heatmaps and (ii) well names depicted in heatmaps, so that the user can easily locate the corresponding movies of interest.

Exact instructions for running COMBO-M are provided as a readme text file, inside the folder that contains the executable file. As shown in main figure 4, COMBO-M consists of two main modules (i) AQDTEM, which is the automated quantification of the differences in time evolving morphologies (DTEM) (ii) VISUALIZATION, which produces high quality custom EPS graphics. The execution of COMBO-M is fully automated with appropriate user menus and console printouts.

4.3 COMBO-C

The flowchart of COMBO-C is described in detail below by referring to all individual modules as numbered in the main figure (fig. 9):

(1) The user selects interactively the target directory, where the images of the experiment are stored. The target directory can be either local or external (e.g., external hard drive). All the results are saved under \Confluence, an automatically created directory under the target directory.

(2) Image quality control. The user has to insert the number of untreated frames per movie, which are automatically transferred into \untreated, where they are separately processed. When the quality control is completed, the detected outliers are removed from the target directory. The detected outliers are saved in TXT and EPS file format under \BU. Foreground segmentation is also executed at this step and the necessary results for the main analysis are saved in a text file named foreground_segmentation.txt under \BU.

(3) Automated quantification of confluence (AQC) employing the MapReduce programming model.

(4) The user selects interactively the .xlsx specification file of the experiment, which contains information about the cell models, barcodes, drugs and corresponding concentrations. Examples are distributed with the standalone application upon request.

(5) The toolbox is built on custom in-house 384-well experimental layouts that allow 15, 20 or 21 and 4 or 8 drug pairs in 4×4 and 6×6 non-zero concentration grids, respectively (see table 2, main text). In this step, the raw confluence values from step (3) are automatically assigned to the custom plate layout.

(6) The raw confluence values are annotated with respect to the specification file from step (5) and saved according to the custom plate layout in CSV file format under \Confluence.

(7) The changes in confluence over time with respect to the first time point are quantified across the whole image library.

(8) Global visualization of results as checkerboard style screens in the form of growth curves in EPS file format under \Confluence.

Exact instructions and for running COMBO-C are provided as a readme text file inside the folder that contains the executable file. As shown in main figure 9, the framework consists of three main modules; (i) AQC, which is the automated quantification of confluence, (ii) RESULTS, which extracts and stores the aforementioned values in CSV file format according to the custom experimental layout and (iii) VISUALIZATION, which produces high-resolution graphics as checkerboard growth screens in EPS file format. The module VISUALIZATION can also be executed independently, provided that the module RESULTS has already been executed. The execution of COMBO-C is fully automated with appropriate user menus and console printouts.

5 Supplementary Results

5.1 Cell Viability Analysis & Visualization

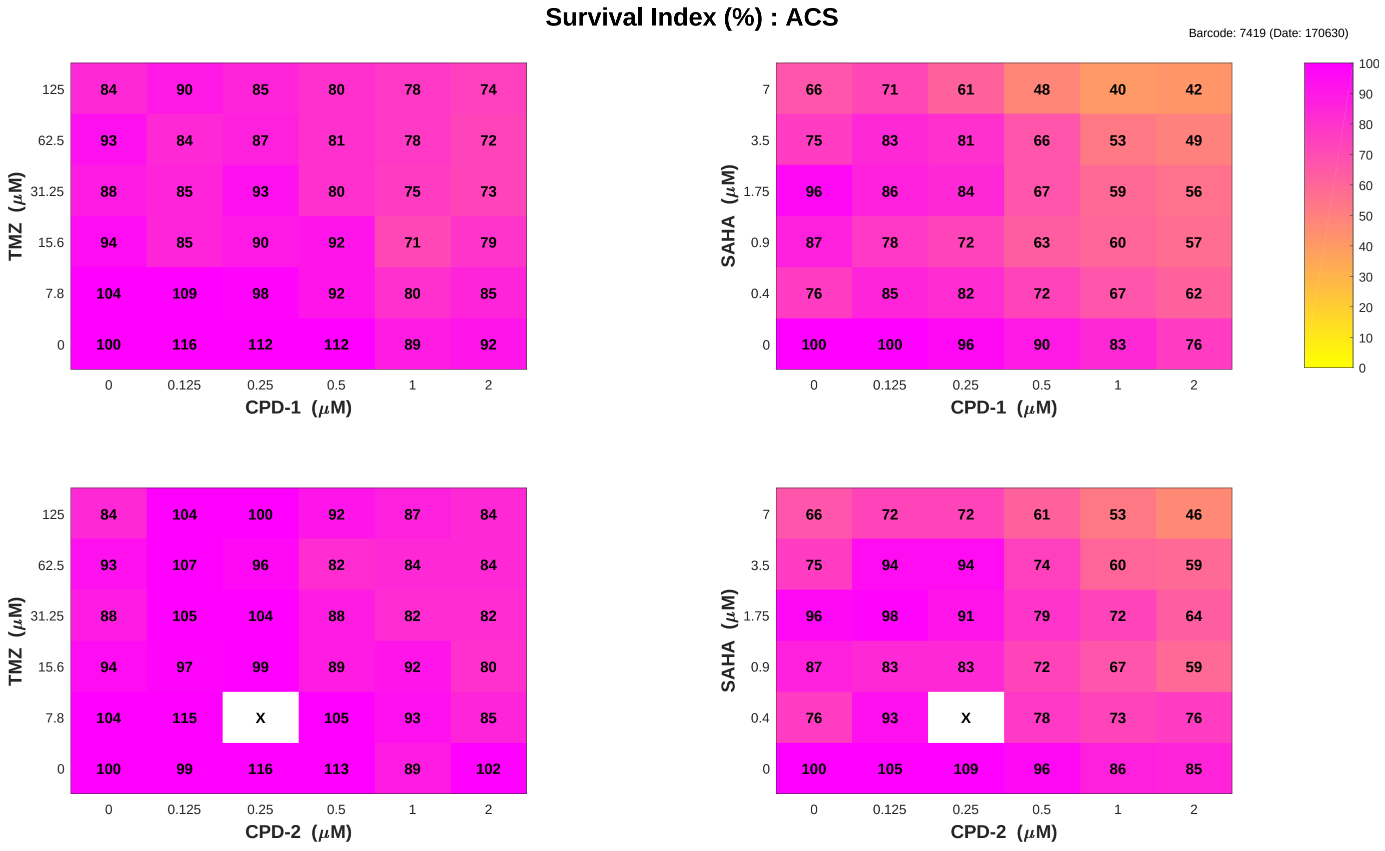

Figure S1: COMBO-V Checkerboard Style Screens. FMCA-based cell viability analysis for astrocytes (ACS). On the bottom left corner of each heatmap, the median survival index of all untreated wells is shown. The color of each combination concentration patch represents the survival index S (%), from purple showing full cell survival (100%) to yellow showing zero cell survival (0%). White patches annotated with "X" are related to survival index values with more than 30% standard deviation between the intra-plate replicates, which have been subsequently excluded.

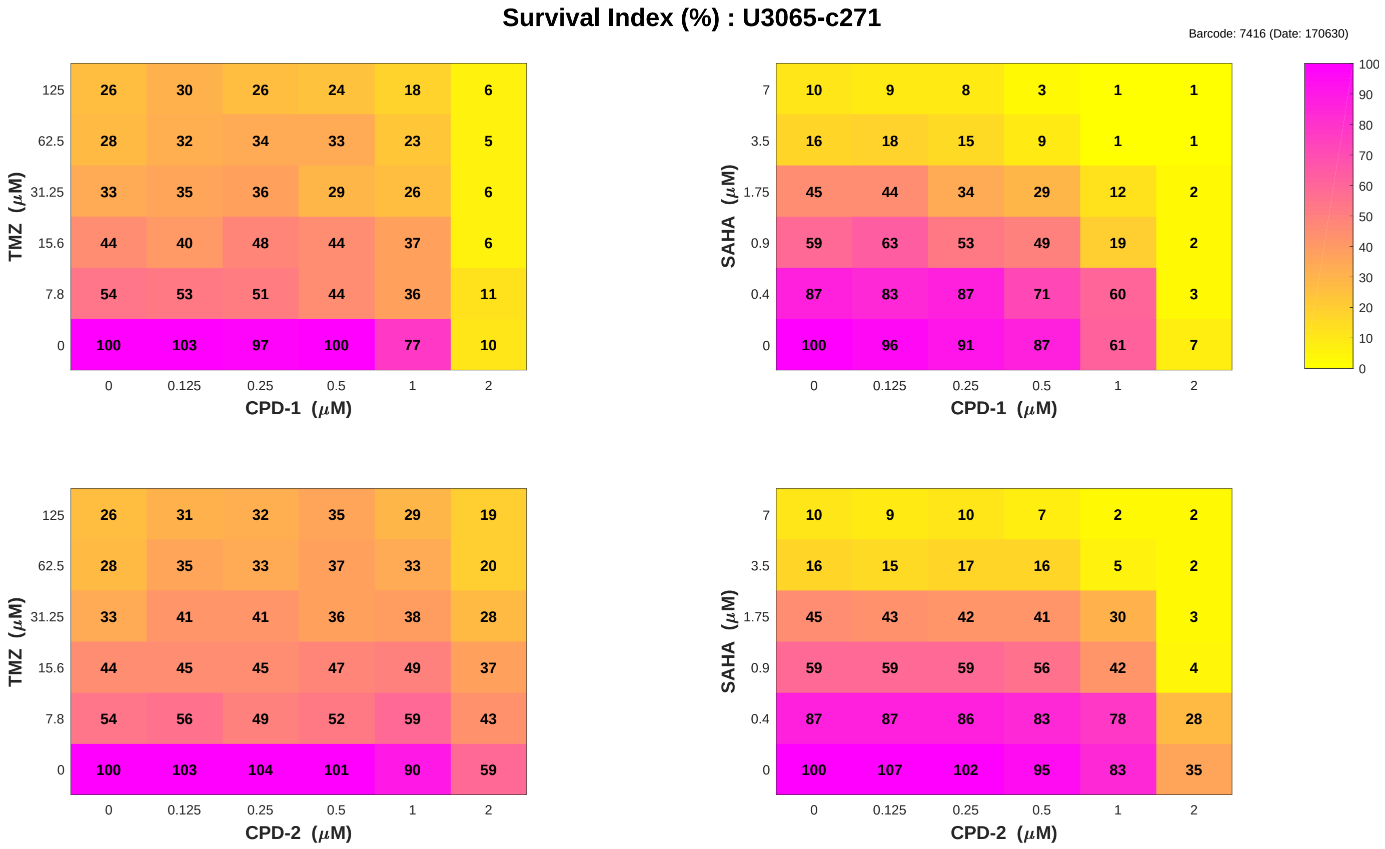

Figure S2: COMBO-V Checkerboard Style Screens. FMCA-based cell viability analysis for the sensitive GIC clone ($U3065 - c271$). On the bottom left corner of each heatmap, the median survival index of all untreated wells is shown. The color of each combination concentration patch represents the survival index S (%), from purple showing full cell survival (100%) to yellow showing zero cell survival (0%).

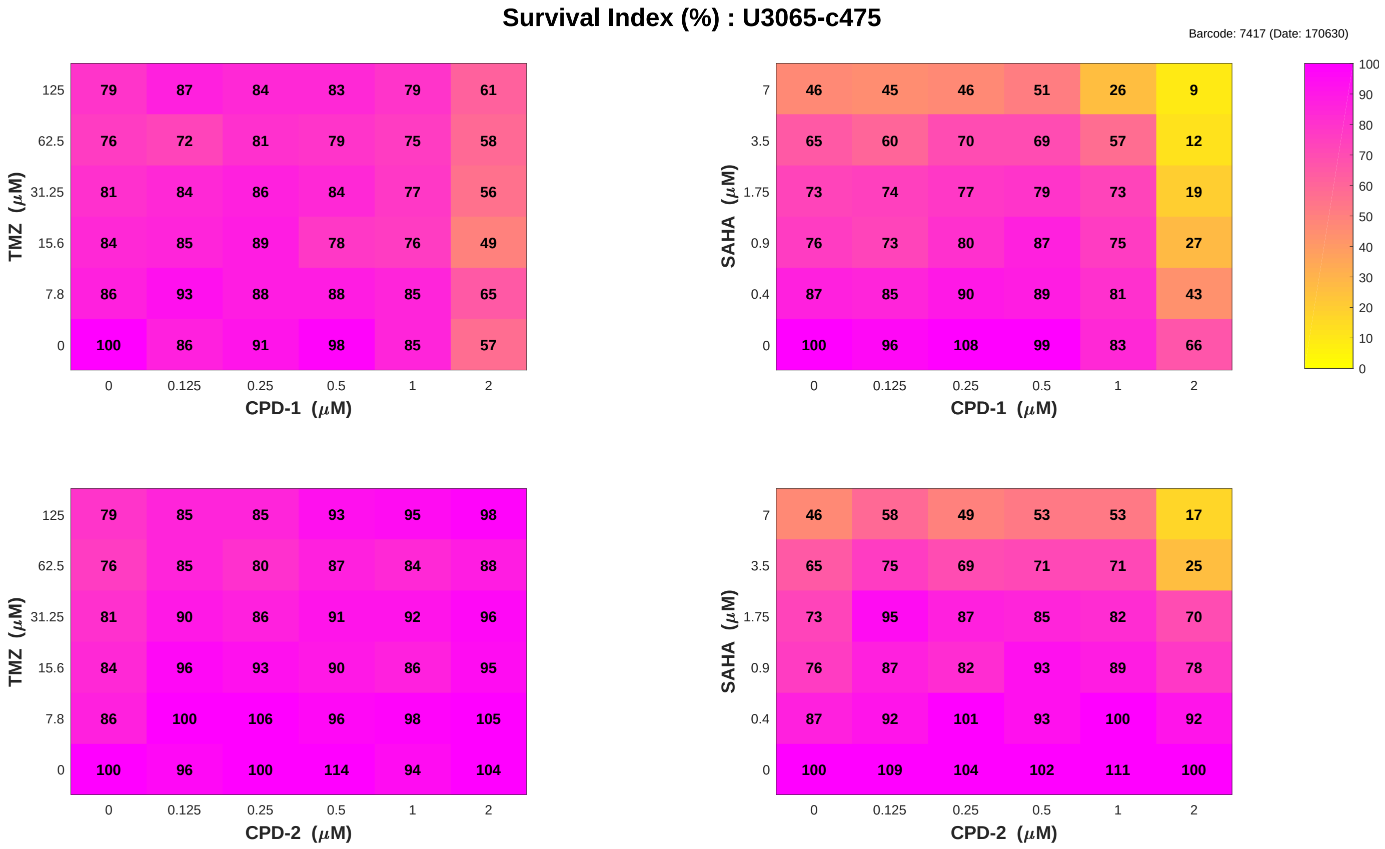

Figure S3: COMBO-V Checkerboard Style Screens. FMCA-based cell viability analysis for the resistant GIC clone ($U3065-c475$). On the bottom left corner of each heatmap, the median survival index of all untreated wells is shown. The color of each combination concentration patch represents the survival index S (%), from purple showing full cell survival (100%) to yellow showing zero cell survival (0%).

Starting with the ACS (fig. S1), they remained unaffected when CPD-1 and CPD-2 were combined with TMZ, but they became relatively affected, when CPD-1 and CPD-2 were combined with SAHA, especially at higher concentrations. The survival index values for $U3065-c271$ (fig. S2) were quite low already when TMZ and SAHA were used alone and almost zero at the highest concentrations of the drug pairs, as well as when CPD-1 was used alone at the highest concentration of 2μM. As for $U3065-c475$ (fig. S3), the survival index dropped substantially when either CPD-1 or CPD-2 at the highest concentration was combined with the partner drug SAHA.

5.2 Bliss Synergy Analysis and Visualization

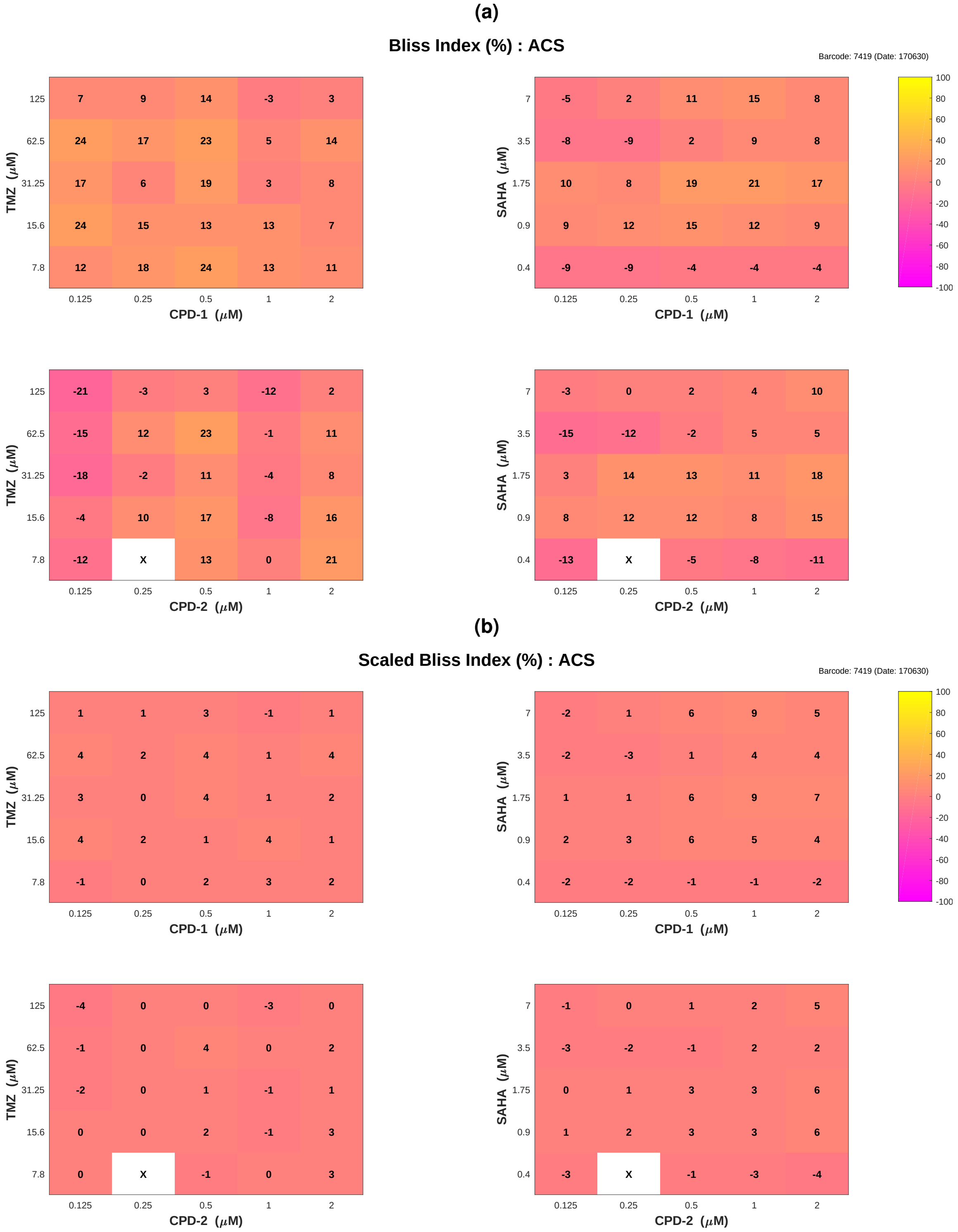

Figure S4: COMBO-V Checkerboard Style Screens. Bliss synergy analyses for astrocytes (ACS): **(a)** conventional; **(b)** scaled. The color of each combination concentration patch represents the conventional and scaled Bliss indices, B and B_S (%) respectively, from purple showing maximal Bliss antagonism (-100%) to yellow showing maximal Bliss synergy (100%). White patches annotated with "X" are related to survival index values with more than 30% standard deviation between the intra-plate replicates, which have been subsequently excluded.

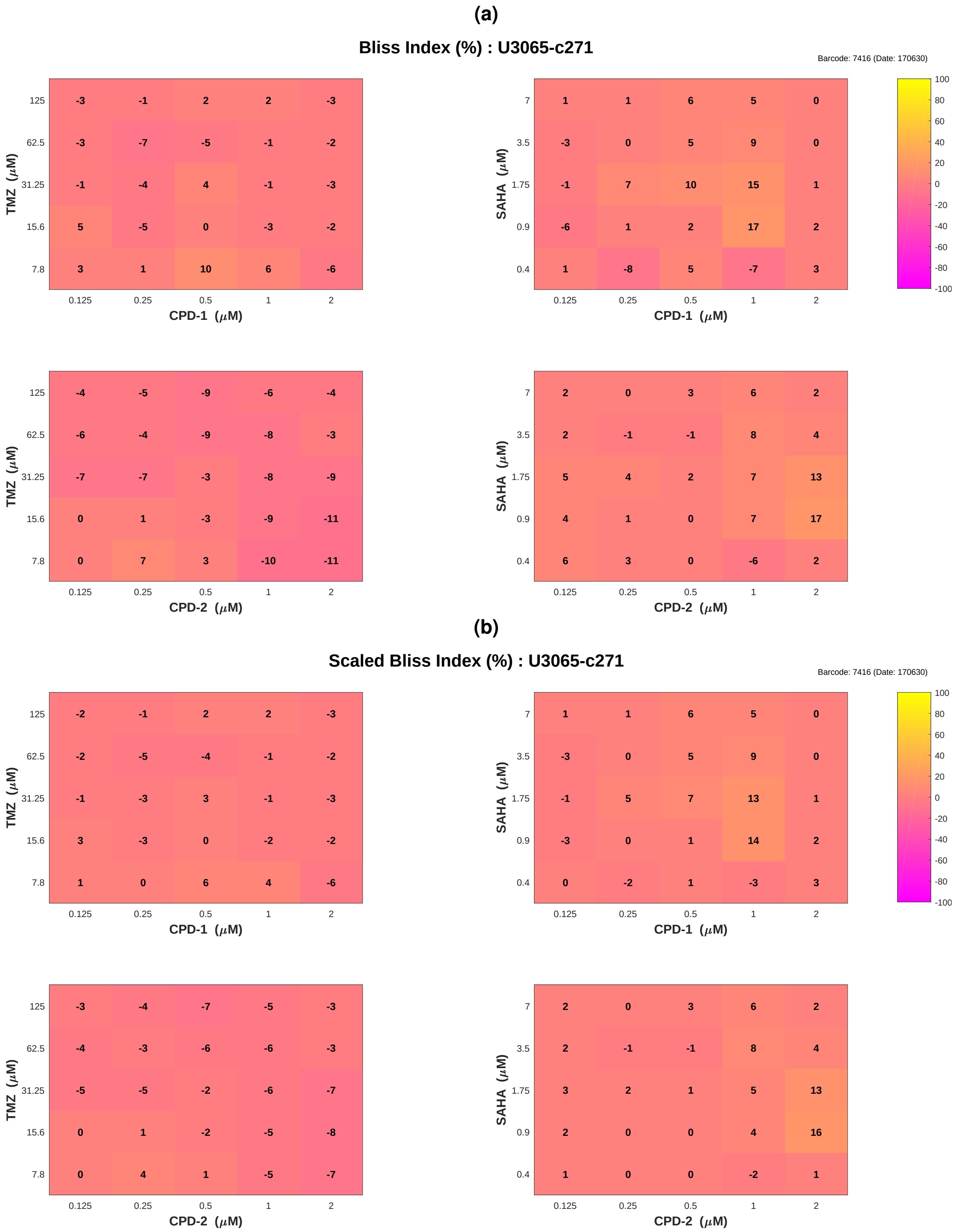

Figure S5: COMBO-V Checkerboard Style Screens. Bliss synergy analyses for the sensitive GIC clone ($U3065 - c271$): **(a)** conventional; **(b)** scaled. The color of each combination concentration patch represents the conventional and scaled Bliss indices, B and B_S (%) respectively, from purple showing maximal Bliss antagonism (-100%) to yellow showing maximal Bliss synergy (100%).

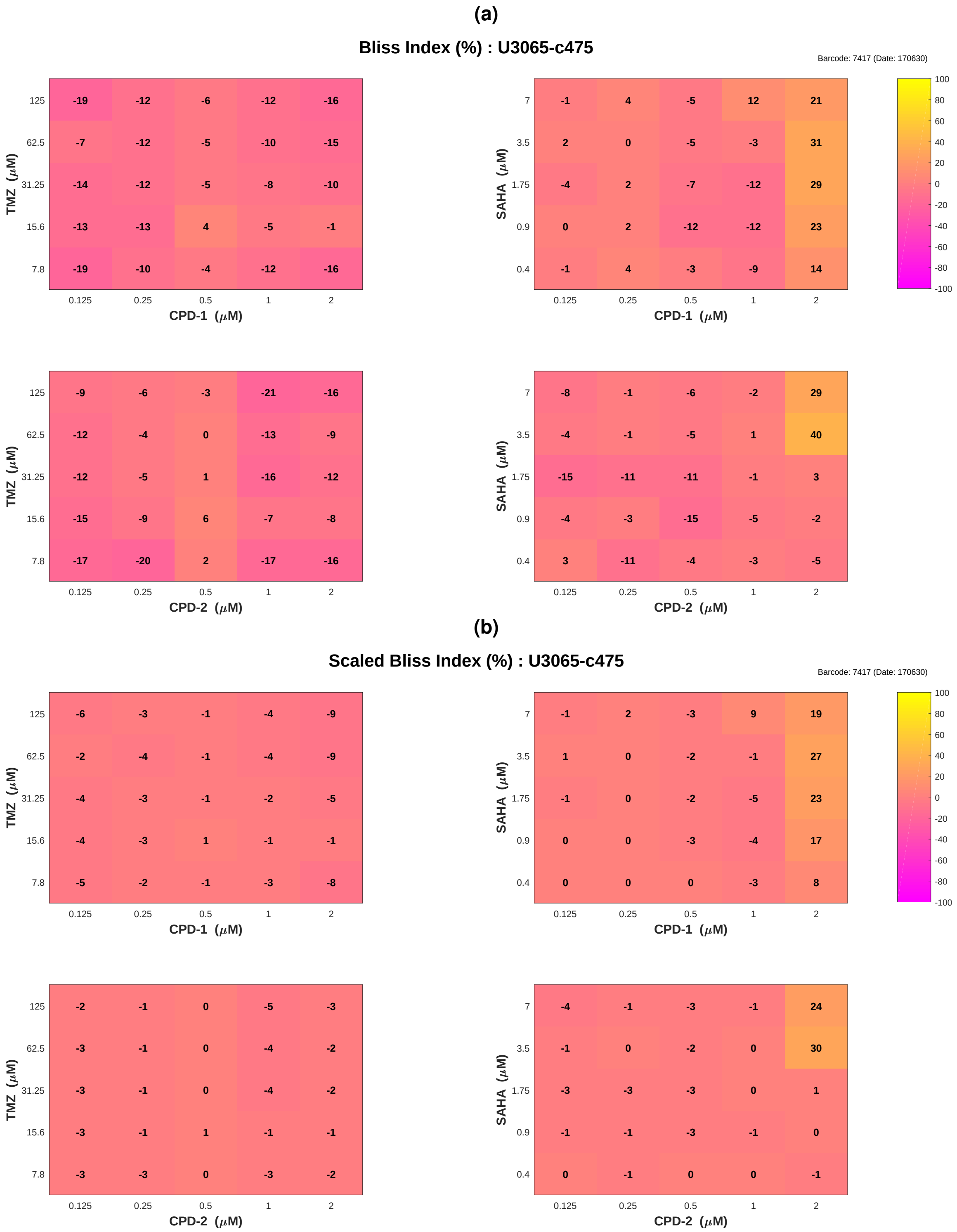

Figure S6: COMBO-V Checkerboard Style Screens. Bliss synergy analyses for the resistant GIC clone ($U3065 - c475$): **(a)** conventional; **(b)** scaled. The color of each combination concentration patch represents the conventional and scaled Bliss indices, B and B_S (%) respectively, from purple showing maximal Bliss antagonism (-100%) to yellow showing maximal Bliss synergy (100%).

Heatmaps from conventional and scaled/refined Bliss synergy analyses enables a direct comparison between these two as well as a global overview of the observed combination effects. In all three cases (fig. S4-S6), the ranked/sorted results given by the scaled

Bliss index were less misleading compared to the unsorted (conventional) Bliss index. For instance, in S4a, there were quite many synergistic combination concentrations among the four drug pairs, which were successfully suppressed in S4b, since they were all associated with high actual survival values (fig. S1). Thus, focusing only on the heatmaps from the scaled Bliss synergy analysis, neutral combination effects were observed overall for the ACS (fig. S4b), limited synergistic combination concentrations were found for $U3065 - c271$ (S5b), while apparent Bliss synergies were detected for $U3065-c475$ (fig. S6b), when CPD-1 and CPD-2 were combined with SAHA at the highest concentrations. Notably, regarding the drug pair (CPD-1, SAHA), Bliss synergistic effects were observed across the whole concentration range for SAHA.

5.3 Therapeutic Synergy Analysis & Visualization

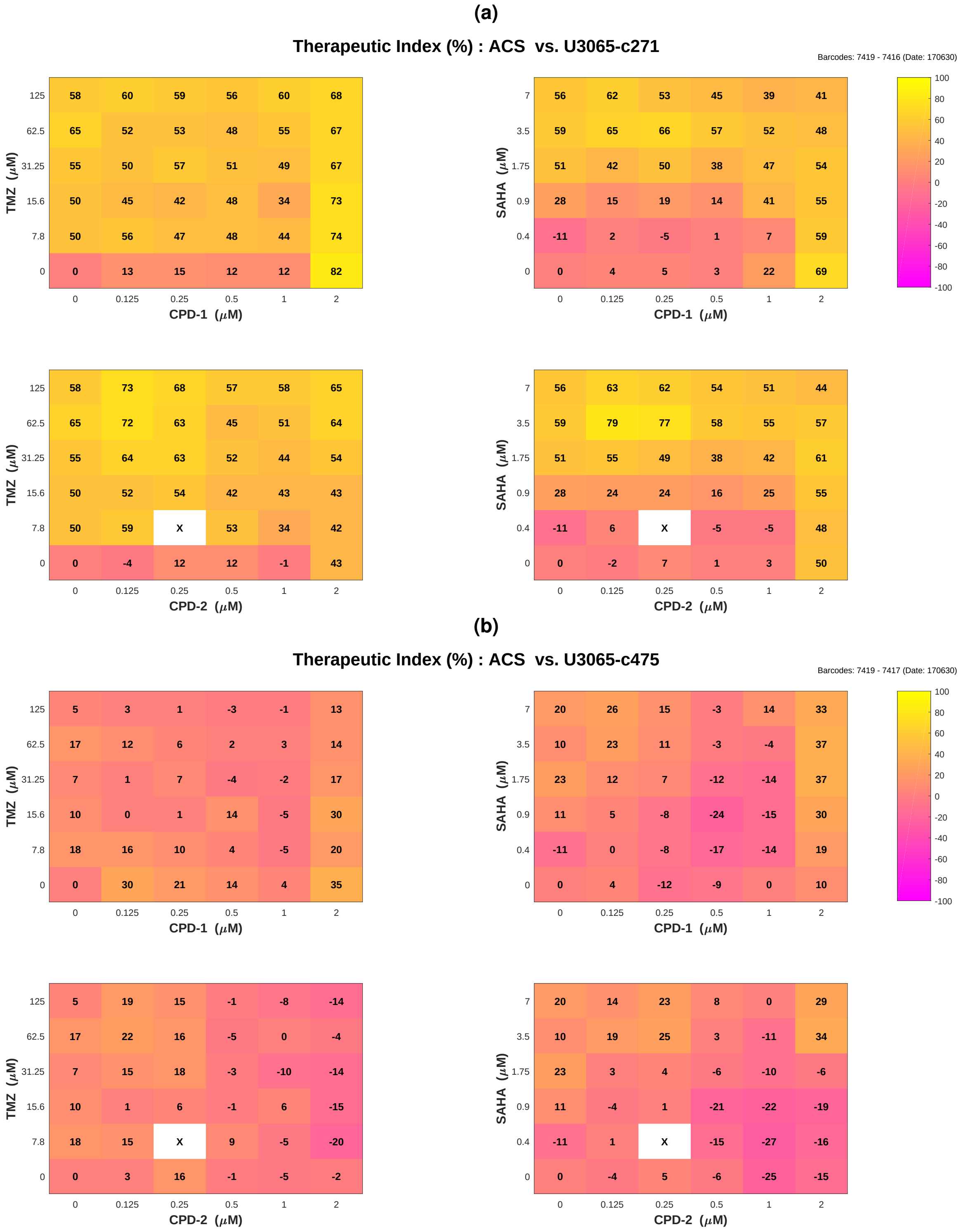

Figure S7: COMBO-V Checkerboard Style Screens. Conventional therapeutic synergy analysis: **(a)** astrocytes (ACS) vs. sensitive GIC clone ($U3065-c271$); **(b)** astrocytes (ACS) vs. resistant GIC clone ($U3065-c475$). The color of each combination concentration patch represents the conventional therapeutic index, T (%), from purple showing maximal therapeutic antagonism (-100%) to yellow showing maximal therapeutic synergy (100%). White patches annotated with "X" are related to survival index values with more than 30% standard deviation between the intra-plate replicates, which have been subsequently excluded.

5.4 COMBO-M PHHC Analyses

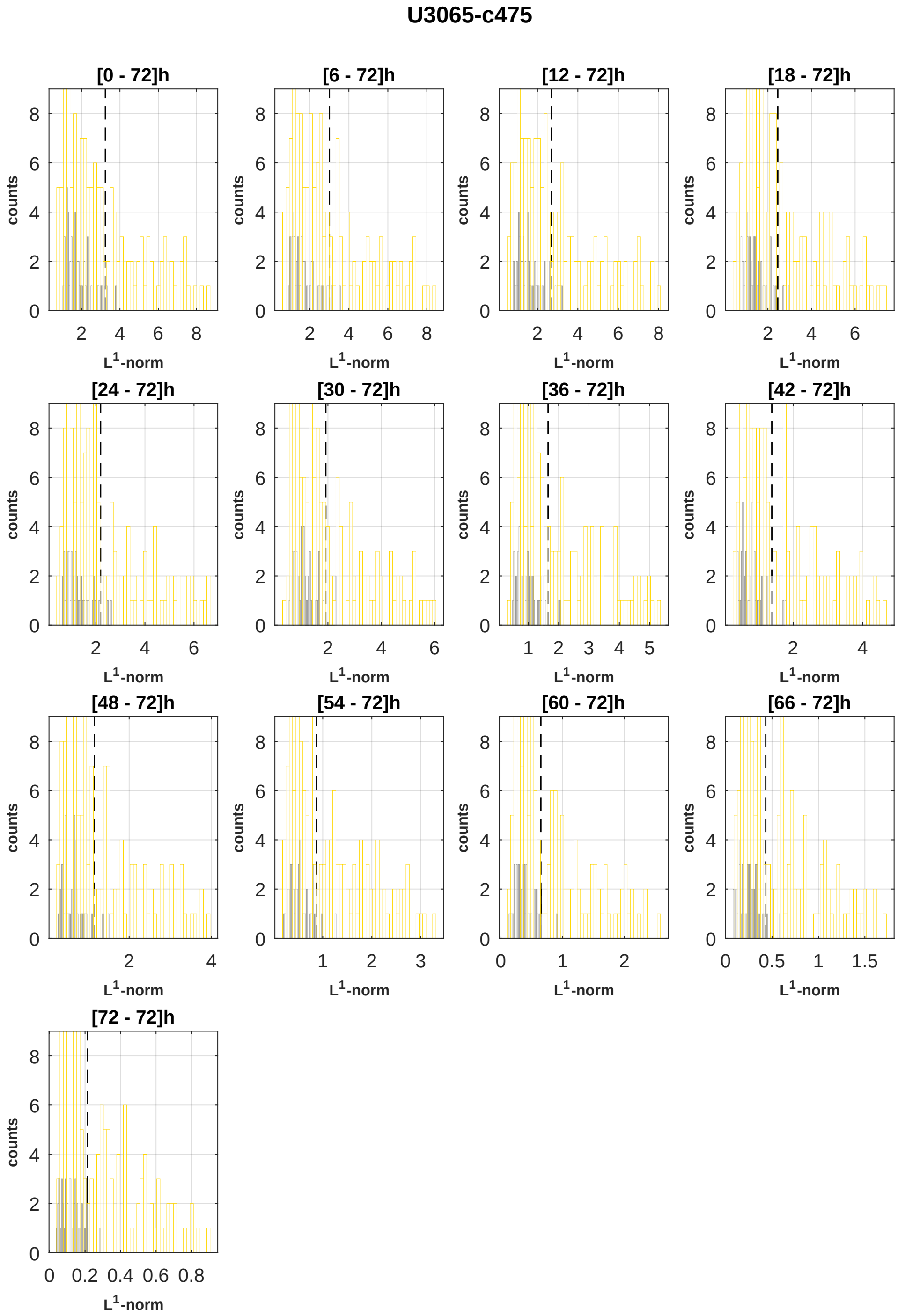

Figure S8: COMBO-M PHHC analyses. Distribution of L^1-norms for the resistant GIC clone ($U3065 - c475$) using 13 decreasing time intervals for the parameter pair $(r, b) = \left(\frac{1}{4}, \frac{1}{2}\right)$. The black and yellow bars correspond to untreated and treated wells, respectively. The black dotted line corresponds to the 95th percentile of the "null" distribution, above which all detections lie.

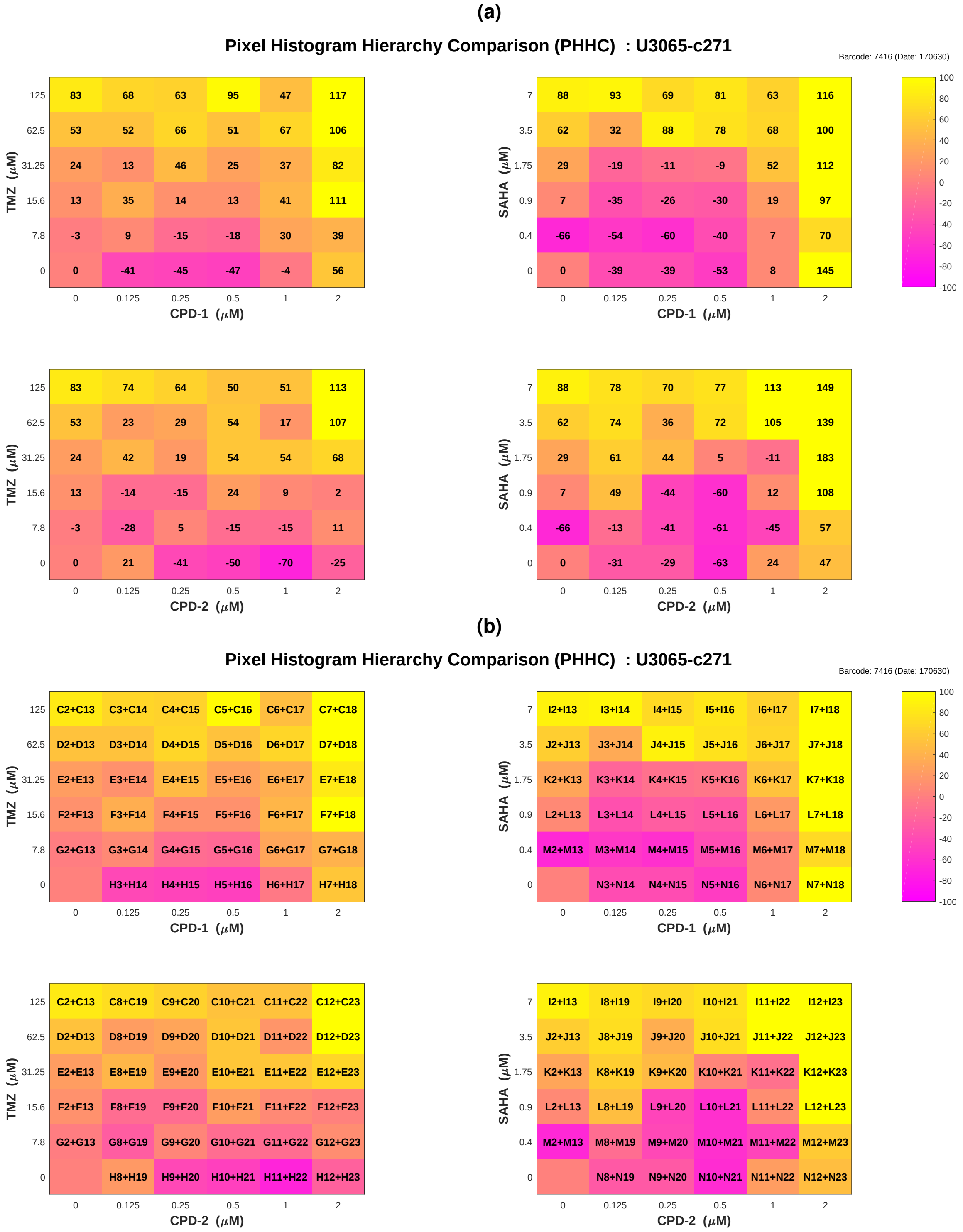

Figure S9: COMBO-M Checkerboard Style Screens. PHHC analysis for the sensitive GIC clone ($U3065-c271$) using the optimum parameter pair $(r^*, b^*) = \left(\frac{1}{2}, \frac{1}{2}\right)$ for the time interval $[60h, 72h]$: **(a)** actual quantified values; **(b)** plate map for movie retrieval. The color of each combination concentration patch represents the relative difference (%) from the top 5% of the corresponding natural/untreated morphological effects, from purple being -100% to yellow being 100%.

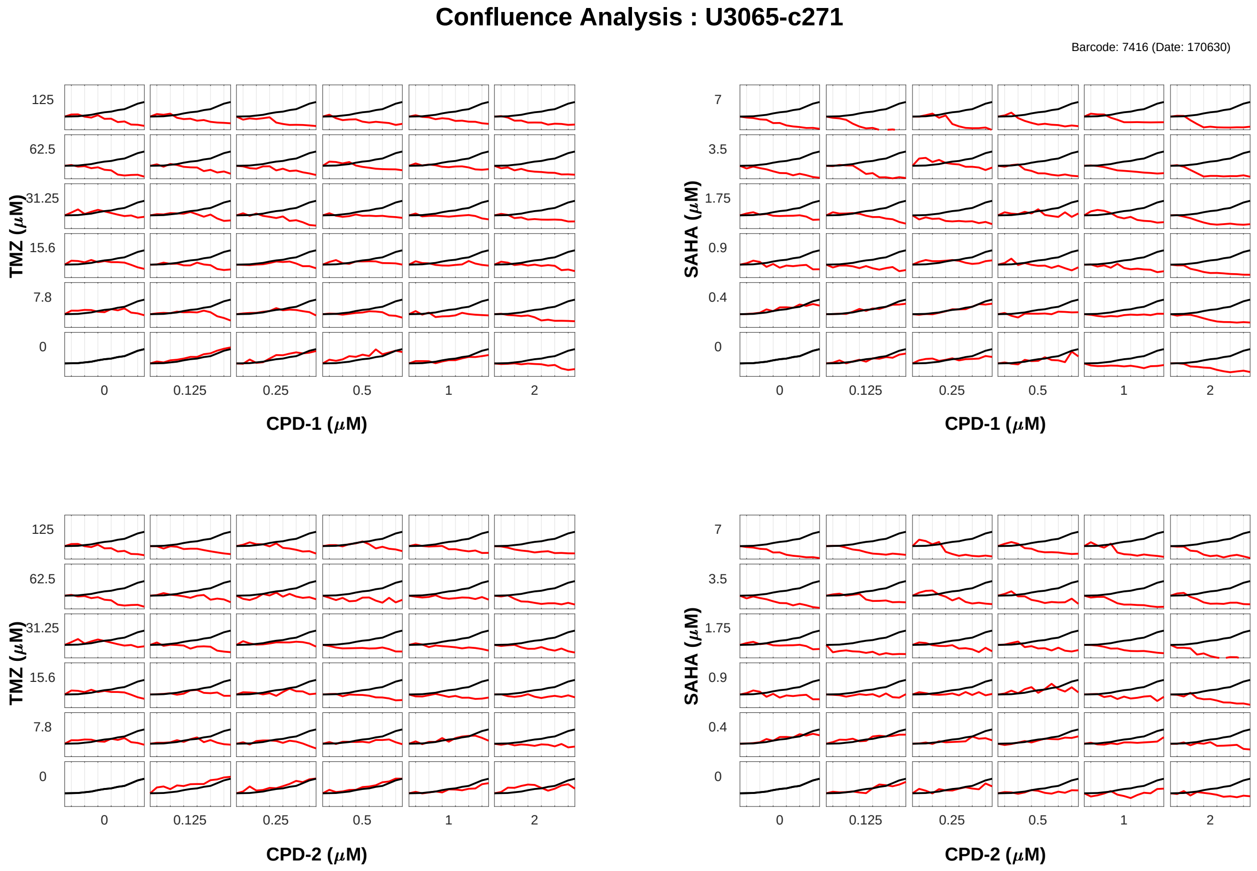

Figure S10: COMBO-C Checkerboard Style Screens. Quantification of changes in confluence for the sensitive GIC clone ($U3065 - c271$). The median growth curve of all untreated wells (black) is shown alone in the lower left subplot of each drug pair, as well as together with the growth curves of treated (red) cells. All growth curves are expressed with respect to the first time point.

The confluence for $U3065-c271$ (fig. S10) dropped substantially from early on ($t_4 = 18h$) among all 4 different drug pairs and concentrations. Notably, apparent decreases were already observed, when the cells were only treated with either high concentrations of the partner drugs TMZ and SAHA or the highest concentrations of CPD-1 and CPD-2. Corresponding cell viability and PHHC analyses resulted in very low survival values (fig. S2) and noticeable morphological changes (fig. S9), respectively.
